# Hydroxy-directed fluorination of remote unactivated C(sp^3^)–H bonds: a new age of diastereoselective radical fluorination†

**DOI:** 10.1039/d2sc01907h

**Published:** 2022-05-30

**Authors:** Stefan Andrew Harry, Michael Richard Xiang, Eric Holt, Andrea Zhu, Fereshte Ghorbani, Dhaval Patel, Thomas Lectka

**Affiliations:** Department of Chemistry, Johns Hopkins University 3400 N. Charles St. Baltimore MD 21218 USA lectka@jhu.edu

## Abstract

We report a photochemically induced, hydroxy-directed fluorination that addresses the prevailing challenge of high diastereoselectivity in this burgeoning field. Numerous simple and complex motifs showcase a spectrum of regio- and stereochemical outcomes based on the configuration of the hydroxy group. Notable examples include a long-sought switch in the selectivity of the refractory sclareolide core, an override of benzylic fluorination, and a rare case of 3,3′-difluorination. Furthermore, calculations illuminate a low barrier transition state for fluorination, supporting our notion that alcohols are engaged in coordinated reagent direction. A hydrogen bonding interaction between the innate hydroxy directing group and fluorine is also highlighted for several substrates with ^19^F–^1^H HOESY experiments, calculations, and more.

## Abstract 2

We report a photochemical, hydroxy-directed fluorination that addresses the prevailing challenge of high diastereoselectivity. Numerous motifs showcase a range of regio- and stereochemical outcomes based on the configuration of the hydroxy group.

## Graphical abstract

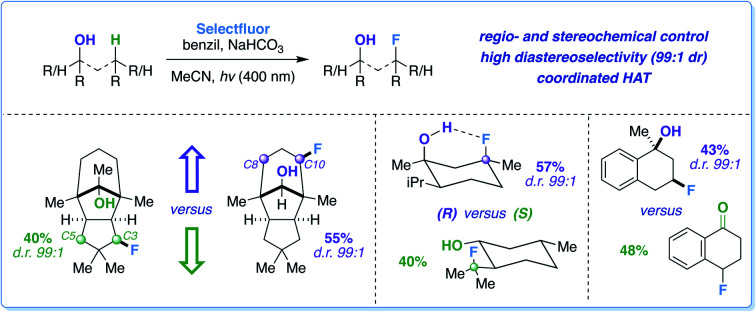

The hydroxy (OH) group is treasured and versatile in chemistry and biology.^1^ Its ubiquity in nature and broad spectrum of chemical properties make it an attractive source as a potential directing group.^2^ The exploitation of the mild Lewis basicity exhibited by alcohols has afforded several elegant pathways for selective functionalization (*e.g.*, Sharpless epoxidation,^3^ homogeneous hydrogenation,^4^ cross-coupling reactions,^5^ among others^6^). Recently, we reported a photochemically promoted carbonyl-directed aliphatic fluorination, and most notably, established the key role that C–H⋯O hydrogen bonds play in the success of the reaction.^7^ Our detailed mechanistic investigations prompt us to postulate that other Lewis basic functional groups (such as –OH) can direct fluorination in highly complementary ways.^8^ In this communication, we report a hydroxy-directed aliphatic fluorination method that exhibits unique directing properties and greatly expands the domain of radical fluorination into the less established realm governing high diastereoselectivity.^9^

Our first inclination that functional groups other than carbonyls may influence fluorination regiochemical outcomes was obtained while screening substrates for our published ketone-directed radical-based method (Scheme 1).^8*a*^ In this example, we surmised that oxidation of the tertiary hydroxy group on substrate 1 cannot occur and would demonstrate functional group tolerance (directing to C11, compound 2). Surprisingly, the two major regioisomers (products 3 and 4) are derivatized by Selectfluor (SF) on C12 and C16 – indicative of the freely rotating hydroxyl directing fluorination. Without an obvious explanation of how these groups could be involved in dictating regiochemistry, we continued the mechanistic study of carbonyl-directed fluorination (Scheme 2A). We established that the regioselective coordinated hydrogen atom abstraction occurs by hydrogen bonding between a strategically placed carbonyl and Selectfluor radical dication (SRD).^7^ However, we noted that the subsequent radical fluorination is not diastereoselective due to the locally planar nature of carbonyl groups. Thus, we posed the question: are there other directing groups that can provide both regio- and diastereoselectivity? Such a group would optimally be attached to a sp^3^ hybridized carbon; thus the “three dimensional” hydroxy carbon logically comes to mind as an attractive choice, and Scheme 1 illustrates the first positive hint.

**Scheme 1:**
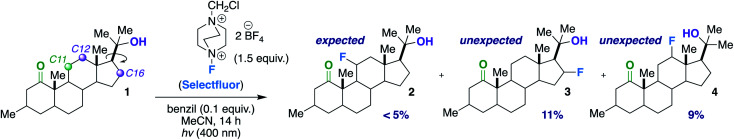
Observed products for the fluorination of compound 1.

**Scheme 2:**
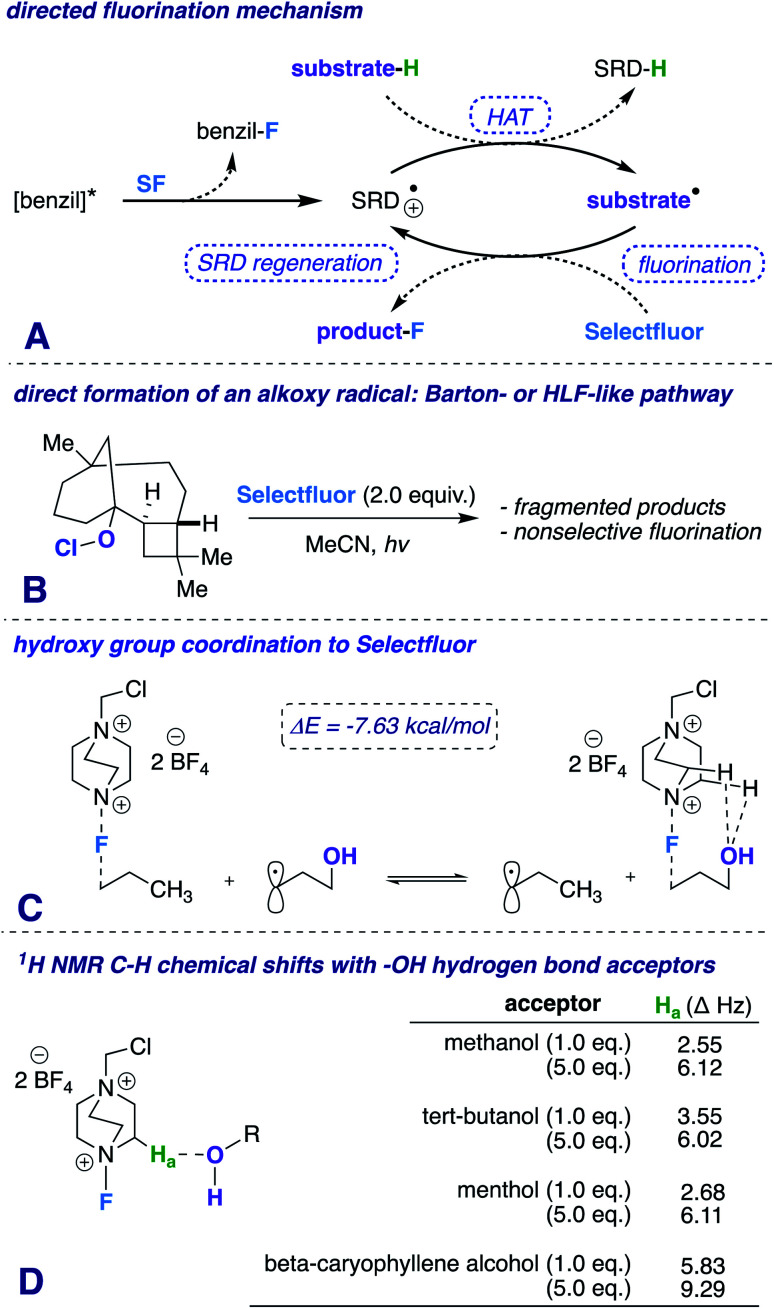
(A) Proposed mechanism, (B) β-caryophyllene alcohol hypochlorite derivative synthetic probe, (C) isodesmic relation of transition states showing the general importance of the hydroxy group to reactivity (ωB97xd/6-31+G*), and (D) ^1^H NMR experiment with Selectfluor and various additives at different concentrations.

We began our detailed study with a simple substrate that contains a tertiary hydroxyl group. Alcohol 5 was synthesized stereoselectively by the reaction of 3-methylcyclohexanone, FeCl_3_, and 4-chlorophenylmagnesium bromide;^10^ the 4-chlorophenyl substituent allows for an uncomplicated product identification and isolation (aromatic chromophore). We sought to determine optimal reaction conditions by examination of numerous photosensitizers, bases, solvents, and light sources (Table 1). To our satisfaction, fluorination not only provides the intended regioisomer but only a single diastereomer is formed (compound 6). A photosensitizer screen shows that benzil (in MeCN solvent) generally affords the highest yield (83%).^7^ Although we utilize cool blue LEDs (sharp cutoff *ca.* 400 nm), CFLs (small amount of UVB (280–315 nm) and UVA (315–400 nm)) are useable as well.^11^ A mild base additive was also found to neutralize adventitious HF and improve yields in the substrates indicated (Table 2). Control substrates, such as methylcyclohexane, afford low or no yields of product mixtures under identical conditions.

**Table 1:** Screening for reaction conditions^a^

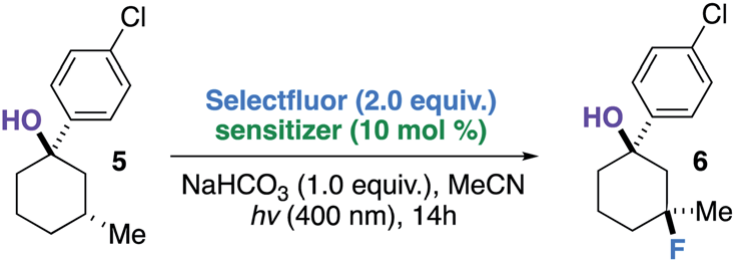		
Entry	Sensitizer	^19^F yield
1	None	0%
**2**	**Benzil**	**83%**
3	Benzil, no base	63%
4	Benzil, K_2_CO_3_	68%
5	Benzil, CFL light source	75%
6	5-Dibenzosuberenone	15%
7	4,4′-Difluorobenzil	63%
8	9,10-Phenantherenequinone	71%
9	Perylene	8%
10	Methyl benzoylformate	42%

a Unless stated otherwise: substrate (0.25 mmol, 1.0 equiv.), Selectfluor (0.50 mmol, 2.0 equiv.), NaHCO_3_ (0.25 mmol, 1.0 equiv.), and sensitizer (0.025 mmol, 10 mol%) were dissolved in MeCN (4.0 mL) and irradiated with cool white LEDs for 14 h.

**Table 2:** Substrate scope^a^

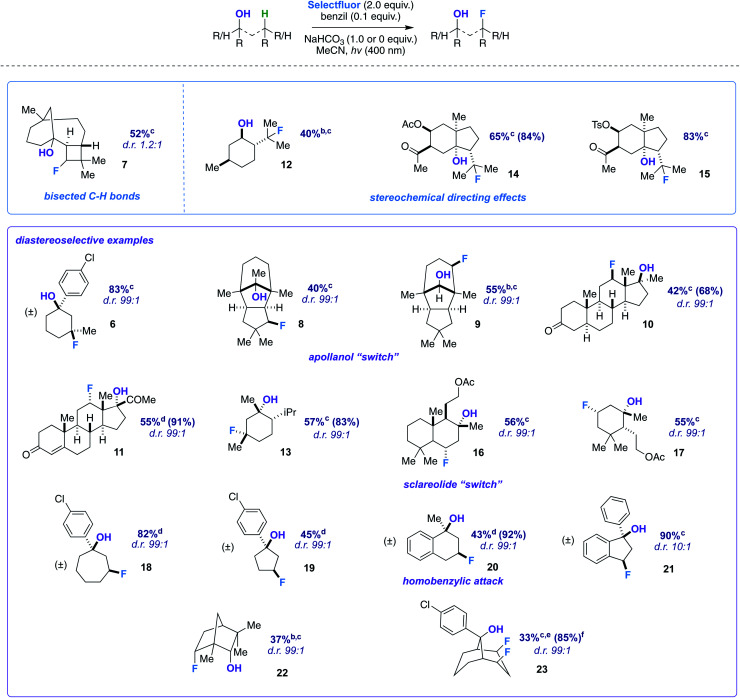

a Unless otherwise specified, the substrate (0.25 mmol, 1.0 equiv.), Selectfluor (0.50 mmol, 2.0 equiv.), NaHCO_3_ (0.25 mmol, 1.0 equiv. or 0.0 equiv.), and benzil (0.025 mmol 10 mol%) were stirred in MeCN (4.0 mL) and irradiated with cool white LEDs for 14 h. Yields were determined by integration of ^19^F NMR signals relative to an internal standard and confirmed by isolation of products through column chromatography on silica gel. Yields based on recovered starting material in parentheses. Major diastereomer (with respect to C–F bond) depicted where known.

b 1.2 equiv. of Selectfluor used.

c 1.0 equiv. of NaHCO_3_.

d 0.0 equiv. of NaHCO_3_.

e 3.0 equiv. of Selectfluor used.

f Including the monofluoride (approx. 11%) with starting material.

The screening concurrently buttresses our claim that hydroxy-directed fluorination is proceeding through a mechanism involving a network of C–H⋯OH hydrogen bonds.^12^ Other N–F reagents (for example, *N*-fluorobenzenesulfonimide and *N*-fluoropyridinium tetrafluoroborate) do not provide the desired fluorinated product 6. The 1,3-diaxial relationship shown in Fig. 1 presents an intramolecular competition: tertiary *vs.* secondary C–H abstraction (O⋯H–C calculated distances: 2.62 and 2.70 Å at B3LYP 6-311++G**, respectively). The tertiary fluoride is the major product in this case.

**Fig. 1:**
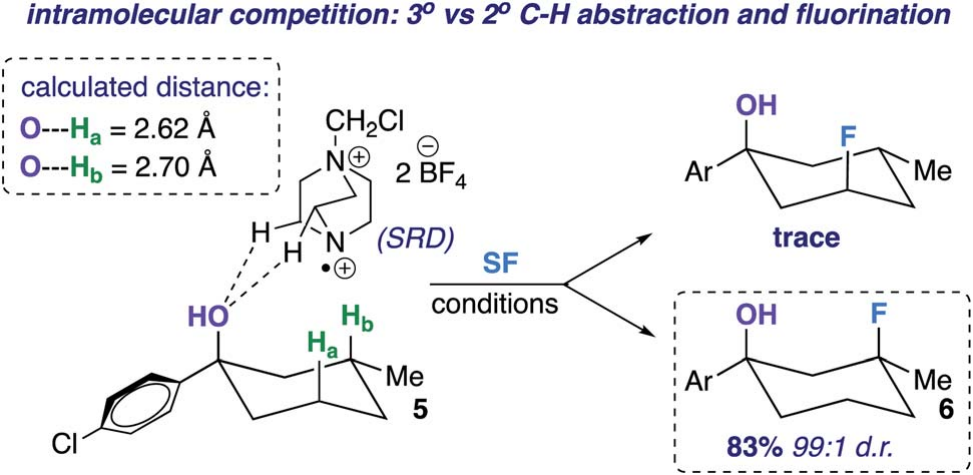
Example of an intramolecular competition (secondary *vs.* tertiary C–H abstraction/fluorination) and calculated C–H⋯O distances of compound 5 (B3LYP/6-311++G**).

With optimized conditions established, we assessed the site-selectivity of the method with a molecule derived from the acid catalyzed cyclization of α-caryophyllene, β-caryophyllene alcohol (commonly used as a fragrance ingredient in cosmetics, soaps, and detergents).^13^ When subjected to fluorination conditions, it targets the strained cyclobutane ring (substrate 7) in 52% yield (Table 2). Based on computational modeling (B3LYP/6-311++G**), the hydroxy group bisects the cyclobutane ring; thus, the diastereomeric ratio is only 1.2 : 1. However, this observation suggests that diastereoselectivity is incumbent upon the relative position of the hydroxy group in space to the carbon radical, and that substrates should be assessed geometrically for suitability. Products 12–15, on the other hand, illustrate that directionality may in turn influence site-selectivity.

Next, we pursued a substrate that supports our notion that hydroxy group orientation can influence both site selectivity and diastereoselectivity favorably. Another unique, rare natural product derived from α-caryophyllene came to mind; 11-apollanol (α-caryophyllene alcohol 9).^14^ The hydroxy group stereochemistry is poised to direct fluorination to either the C8 or C10 positions (compound 9) due to the plane of symmetry (Fig. 3A). Moreover, we synthesized a complementary derivative through PCC oxidation followed by a Grignard reaction, thereby switching directionality of the hydroxy group (Fig. 3A) to target the C3 or C5 positions instead (compound 8). We found the resultant fluorinated products to be what one expects if engaged in coordinated hydrogen atom transfer (HAT) (55% and 40% for molecules 9 and 8) – a change in regiochemistry based on the stereochemistry of the alcohol. Additionally, only a single stereoisomer is produced for both (d.r. 99 : 1) and reinforce this study as a salient example of diastereoselective radical fluorination.

**Fig. 2:**
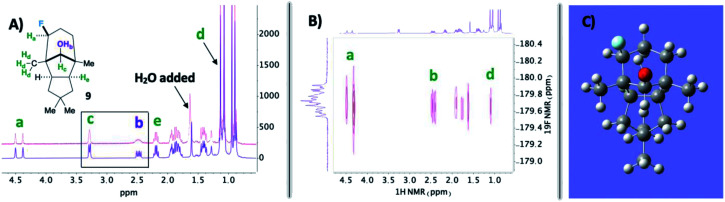
(A) Top spectrum (pink) has broadened peaks due to adventitious H_2_O in solution. (B) Strong interaction observed between the installed fluorine and designated hydroxy proton in the ^19^F–^1^H HOESY NMR spectrum. (C) Calculated structure for compound 9 at B3LYP/6-311++G* revealing the hydroxy proton aiming toward the fluorine.

**Fig. 3:**
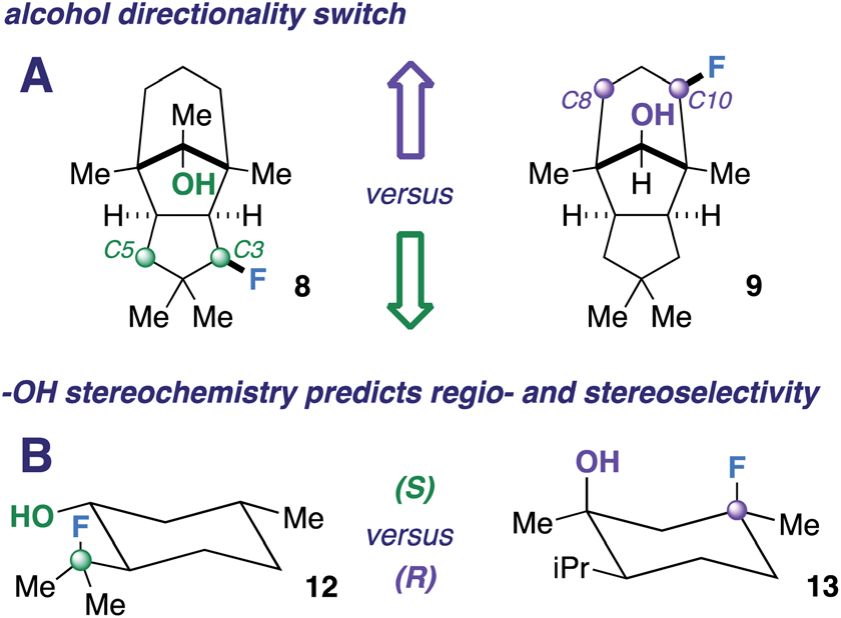
Examples of hydroxy group stereochemical switches.

In the midst of characterizing compound 9, we uncovered a noteworthy hydrogen bonding interaction. Firstly, our plan was to identify the –OH peak within the ^1^H NMR spectrum and determine if there is a through-space interaction with fluorine in the ^19^F–^1^H HOESY NMR spectrum (ultimately aiding in assigning the stereochemistry of the fluorine).^15^ At first glance, no peaks were immediately discernible as the –OH; however, when a stoichiometric amount of H_2_O is added, it becomes apparent that the –OH group and geminal proton to the hydroxy peaks broaden by rapid proton exchange (Fig. 2A). Upon closer examination of the dry ^1^H NMR spectrum, the –OH peak appears to be a sharp doublet of doublets: one bond coupling to the geminal C–H proton of 9 Hz and one of the largest reported through-space couplings to fluorine of 20 Hz. The ^19^F–^1^H HOESY spectrum also supports our regio- and stereochemical assignment – a strong interaction between fluorine and H_a_, H_b_, and H_d_, as well as no apparent interaction with H_c_ and H_e_ (Fig. 2B). Consequently, we postulate that intramolecular hydrogen bonding is responsible for the considerable coupling constant. This conclusion is also supported by calculations at B3LYP/6-311++G** (Fig. 2C): the O–H–F angle is given as 140° and F⋯H–O bond distance is 1.97 Å.

Appreciating the complexity and biological significance of steroids,^16^ we derivatized dehydroepiandrosterone to afford fluorinated substrate 10 (42%; d.r. 99 : 1). Computational modeling assisted in verifying that the β-hydroxy group targets the C12 position (B3LYP/6-311++G**); furthermore, the β-fluoro isomer is the major product (validated by NOESY, ^1^H, and ^19^F NMR). Additionally, we subjected 17α-hydroxyprogesterone (endogenous progestogen steroid hormone^17^) to fluorination conditions and found the α-fluoro product (11) as the major diastereomer in 55% yield (99 : 1 d.r.). To investigate further the notion of coordinated fluorination and explanation of the observed stereoisomers (*e.g.*, β-hydroxy/β-fluoro and α-hydroxy/α-fluoro), we calculated a simplified system comparing the fluorination of 1-propyl radical and γ-propanol radical (Scheme 2C). The reaction can be distilled into two key steps: a site-selective HAT, followed by a diastereoselective fluorination reaction. The following isodesmic relation (ωB97xd/6-31+G*, −7.63 kcal mol^−1^) illustrates the stabilizing energetic role that the hydroxy group plays in commanding diastereoselectivity. The transition states represent low barrier processes; a solvent dielectric was necessary to find saddle points.

Additionally, a simple Protein Data Bank (PDB) survey showed numerous intermolecular close contacts between hydroxy groups and H–C–^+^NR_3_ moieties.^18^ What is more, solutions of Selectfluor with various alcohols at different concentrations reveal characteristic H–C–^+^NR_3_ downfield chemical shifts in the ^1^H NMR spectra (Scheme 2D).^19^ Both of these observations buttress the claim of a putative hydrogen bonding interaction between Selectfluor and the hydroxy group.

We theorize that the regioselective HAT step proceeds similarly to the reported carbonyl-directed pathway (Scheme 2A) involving Selectfluor radical cation coordination (considering the likenesses in conditions and aforementioned Lewis basicity logic). Alternatively, one can imagine the reaction proceeding through a Barton^20^ or Hofmann–Löffler–Freytag^21^ style mechanism. To probe this possibility, we employed a β-caryophyllene alcohol hypochlorite derivative to form the alkoxy radical directly, and found that under standard conditions there is complex fragmentation and nonselective fluorination (Scheme 2B). Lastly, we compared the hydroxy *versus* carbonyl group SF coordination computationally. The carbonyl group is preferred to bind to SF through nonclassical C–H⋯O hydrogen bonds preferentially over the hydroxy group, as the following isodesmic relation shows (acetone and *t*-BuOH as models; ωB97xd/6-31+G*, −3.81 kcal mol^−1^), but, once again, rigidity and propinquity are ultimately more important factors in determining directing effects (Scheme 3).

**Scheme 3:**
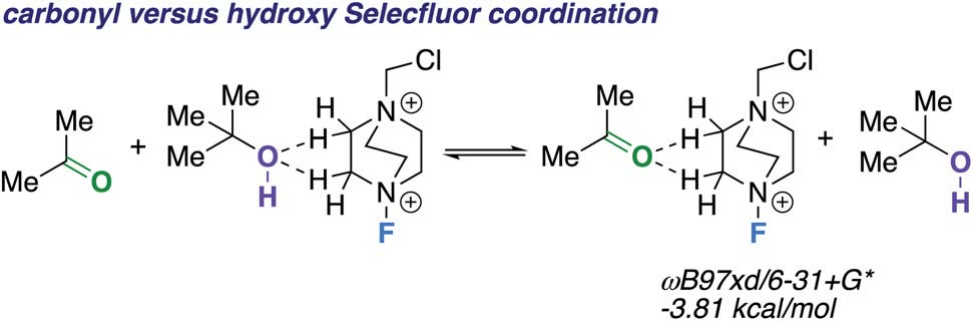
Isodesmic equation comparing carbonyl *versus* hydroxy group Selectfluor coordination.

The tetrahedral nature of hydroxy groups provides unique access to previously unobtainable sites. For example, we compared menthol and an alkylated congener to form products 12 and 13 (Fig. 3B). The hydroxy group in the precursor to 12 is in the equatorial position, mandating the exocyclic isopropyl group as the reactive site (40% yield).^22^ In the precursor to 13, the methyl and isopropyl substituent lock the hydroxy group into the axial position, targeting its endocyclic tertiary site through a 1,3-diaxial relationship to afford fluorinated product in 57% yield (d.r. 99 : 1). In all, the comparison showcases the versatility in directing ability, offering a choice of regio- and stereoselectivity based on the stereochemistry of the hydroxy group. The directing system only necessitates two features based on our results: (1) the hydroxy group must be either secondary or tertiary (primary tends to favor oxidation) and (2) the oxygen atom must be within the range of 2.4–3.2 Å of the targeted secondary or tertiary hydrogen.

Among the several biologically active compounds we screened, caratol derivatives 14 and 15 were found to be attractive candidates that reveal directed fluorination to an exocyclic isopropyl group (Table 2). Comprising *ca.* 40% of carrot seed oil, caratol is its major constituent and has shown allelopathic interactions (*e.g.*, as insecticidal, antifungal, and herbicidal agents^23^).^24^ After extraction, isolation, and derivatization, molecules 14 and 15 are afforded in 65% and 83% yield (Table 2). Acetates and oxidized sulfur-containing functional groups were well tolerated.

Another natural product we modified to a tertiary alcohol was sclareolide, which has a rich history of fluorinating on its C2 and C3 positions due to polar effects, as reported by Tang,^25^ Groves,^9*f*^ Britton,^26^ and others.^27^ The derived alcohol finally overrode this natural tendency and directed to the predicted position in 56% (d.r. 99 : 1) (product 16). Smaller amounts of competitive polar effect fluorination were observed at the C2 and C3 positions, highlighting how challenging a problem the functionalization of the sclareolide core presents.^28,29^

An altered dihydroactinidiolide was found to participate in the fluorination through a 1,3-diaxial guided HAT and fluorination in 55% yield (product 17, d.r. 99 : 1). We next modeled several more substrates that participated in similar 1,3 relationships; however, each exhibited a variation from one another (*e.g.*, ring size or fused aromatic ring). Products 19 and 18 displayed the reaction's capability to direct to the desired positions with an expanded (65%; d.r. 99 : 1) and reduced (45%; d.r. 99 : 1) ring system when compared to the previous 6-membered ring examples. Additionally, we examined a methylated α-tetralone derivative. The desired 3-fluoro product 20 forms in 43% yield (d.r. 99 : 1), overriding benzylic fluorination (Scheme 4).^30^ Under identical conditions α-tetralone provides 4-fluorotetralone in 48% yield. In similar motif, 1-phenylindanol, we intentionally targeted the benzylic position in a 90% and 10 : 1 d.r. (product 21). Unlike the methylated α-tetralone derivative, the geometry of the starting material calculated at B3LYP/6-311++G** shows the hydroxy group is not truly axial and is 4.30 Å from the targeted C–H bond, explaining the dip in diastereoselectivity.

**Scheme 4:**
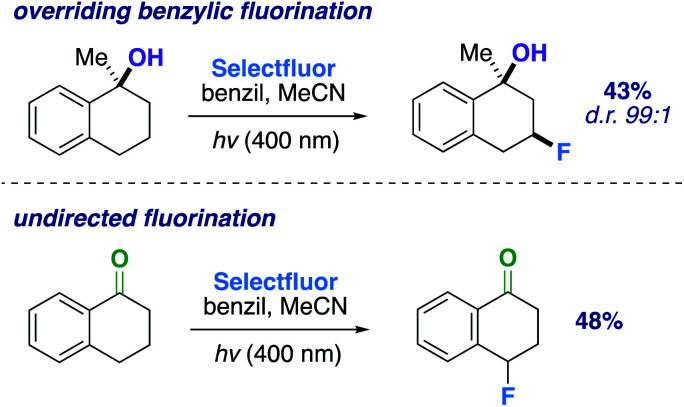
Comparing fluorination outcomes for different functional groups.

Next, we examined an isomer of borneol that is widely used in perfumery, fenchol.^31^ The secondary alcohol displays a diastereoselective fluorination in 38% (d.r. 99 : 1) (product 22). Our last designed motif was ideally constructed to have a doubly-directing effect. Our observations show that a well-positioned hydroxy group not only provides sequential regioselective hydrogen atom abstraction but also displays a powerful demonstration of Selectfluor guidance to afford the *cis*-difluoro product (23) in 33% yield (85% brsm, d.r. 99 : 1). Spectroscopically (^1^H, ^13^C, and ^19^F NMR), the product possesses apparent C_s_ symmetry and showcases close interactions (*e.g.*, diagnostic couplings and chemical shifts). *cis*-Polyfluorocycloalkanes are of intense current interest in materials chemistry, wherein faces of differing polarity can complement one another.^32^

All in all, this photochemical hydroxy-directed fluorination report represents one of the first steps in commanding diastereoselectivity within the field of radical fluorination. An ability to dictate regio- and stereoselectivity is demonstrated in a variety of substrates by simply switching the stereochemistry of the hydroxy group. Computations support the key role of Selectfluor coordination to the key hydroxy group in the fluorination step. Future studies will seek to uncover other compatible Lewis basic functional groups, expanding further the versatility of radical fluorination.

## Data availability

Experimental and computational data is located in the ESI.†

## Author contributions

S. Harry designed probes, synthesized compounds, and provided drafts of the manuscript. M. Xiang, E. Holt, A. Zhu, F. Ghorbani, and D. Patel synthesized compounds for isolation/characterization and assisted in compiling data. T. Lectka supervised the project and reviewed/edited the manuscript.

## Conflicts of interest

The authors declare no competing financial interest.

## Supplementary Material

SC-013-D2SC01907H-s001
