# Supplementary material for: Hydroxy-directed fluorination of remote unactivated C(sp3)–H bonds: a new age of diastereoselective radical fluorination
Source: Chem Sci. 2022 May 30;13(23):7007–13. doi: 10.1039/d2sc01907h (PMC9200124; doi:10.1039/d2sc01907h)
Supplement: SC-013-D2SC01907H-s001 [file SC-013-D2SC01907H-s001.pdf]

Electronic Supplementary Material (ESI) for Chemical Science.
This journal is © The Royal Society of Chemistry 2022

Supporting Information

Hydroxy-Directed Fluorination of Remote Unactivated C(sp^3)-H Bonds: A New Age of Diastereoselective Radical Fluorination

Stefan Andrew Harry, Michael Richard Xiang, Eric Holt, Andrea Zhu, Fereshte Ghorbani, Dhaval Patel, and Thomas Lectka*

Department of Chemistry, Johns Hopkins University, 3400 N. Charles St., Baltimore, MD, 21218, United States

Contents:

General Information, S1
General Fluorination Procedures, S2
Procedure for the NMR Experiment, S2
Starting Material Syntheses and Characterization Data, S2-S24
NMR Spectral Data, S25-S98
Computational Data, S99-S115
Mass Spectral Data, S116-S139
References, S140

General Information

Unless otherwise stated, all reactions were carried out under strictly anhydrous conditions and N_2 atmosphere. All solvents were dried and distilled by standard methods. All ^{1}H spectra were acquired on a 400 MHz NMR spectrometer in CD_3CN or $CDCl_3$, ^{19}F spectra were acquired on a 300 MHz NMR spectrometer in $CDCl_3$, and ^{13}C NMR spectra were acquired on a 400 MHz NMR spectrometer in $CDCl_3$. The ^{1}H and ^{13}C NMR chemical shifts are given in parts per million (δ) with respect to an internal tetramethylsilane (TMS, δ = 0.00 ppm) standard. NMR data are reported in the following format: chemical shift (integration, multiplicity (s = singlet, d = doublet, t = triplet, q = quartet, m = multiplet), coupling constants (Hz)). Spectral data were processed with Bruker software. Photochemical reactions were run in front of a 72-LED work light (Designers Edge L1923). HPLC purification (if necessary) was conducted on a Teledyne Isco CombiFlash EZ Prep system using a Dynamax-60A SiO_2 column and HPLC grade EtOAc and hexanes. The Gaussian '09 package was used for all calculations.[1]

General Fluorination Procedure

Selectfluor (177 mg, 0.50 mmol), benzil (5.0 mg, 0.025 mmol), $NaHCO_3$ (21 mg, 0.25 mmol), and the substrate (0.25 mmol) were added to an oven-dried μω vial equipped with a stir bar; the vial was then sealed with a cap with a septum using a crimper and evacuated/refilled with N_2 multiple times. Anhydrous CH_3CN (4 mL) was added, and the reaction mixture was irradiated with a cool white LED work light while stirring. After 14 h, a 0.3 mL aliquot was taken for ^{19}F NMR yield determination, and the rest of the reaction mixture was transferred to a separatory funnel, diluted with H_2O, and extracted into CH_2Cl_2. The combined organic layers were washed with H_2O and brine, then dried with $MgSO_4$, filtered through Celite, and concentrated. The crude reaction mixture was purified via gradient column chromatography on silica gel eluting with EtOAc and hexanes.

Procedure for the NMR Experiment

Selectfluor (20 mg, 0.056 mmol, 1.0 equiv.) was dissolved in 0.6 mL CD3CN in a small glass vial. The hydrogen bond acceptor (1.0 or 5.0 equiv.) was added to the vial and the contents were mixed before being transferred into an NMR tube. The ^1H NMR spectrum was obtained.

Starting Material Syntheses and Characterization

(1S,3S)- and (1*R*,3*R*)-1-(4-chlorophenyl)-3-methylcyclohexan-1-ol[2]

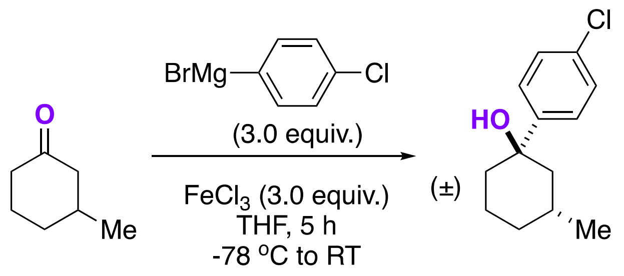

To a flame-dried three-neck round-bottom equipped with a stir bar under N_2 were added $FeCl_3$ (4.37 g, 27.0 mmol) and a suspension of 3-methylcyclohexanone (1.00 g, 8.93 mmol) in THF (30.0 mL). The reaction mixture was cooled to -78 °C and 1M 4-chlorophenylmagnesium bromide in Et_2O (27.0 mL, 27.0 mmol) was added. The reaction mixture was slowly warmed to RT over 5 h. The reaction mixture was quenched with 1M HCl and extracted into CH_2Cl_2 repeatedly. The combined organic layers were dried with $MgSO_4$, filtered through Celite, and concentrated. The crude residue was purified via gradient column chromatography to provide (1S,3S)- and (1R,3R)-1-(4-chlorophenyl)-3-methylcyclohexan-1-ol (1.70 g, 85%).

White solid. ^1H NMR (400 MHz, $CDCl_3$): δ 7.44-7.40 (m, 2H), 7.31-7.27 (m, 2H), 1.95-1.82 (m, 1H), 1.80-1.65 (m, 7H), 1.43-1.33 (m, 1H), 0.98-0.91 (m, 4H). $^{13}C\{^1H\}$ NMR (100 MHz, $CDCl_3$):

δ 148.3, 132.4, 128.3, 126.1, 73.7, 47.7, 38.5, 34.30, 28.3, 22.6, 22.0. FTMS (ESI) m/z $C_{13}H_{17}OCl$: calc 224.0968, observed 224.0964.

(2*R*,5*S*)-4,4,8-trimethyltricyclo[6.3.1.0^{2,5}]dodecan-1-ol

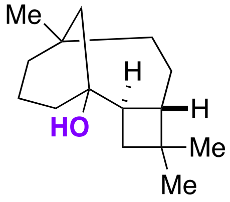

Obtained from Prof. Alex Nickon's (JHU) chemical reserves.

Spectral data matches what is reported in the literature.[3]

(3a*R*,4*R*,8*S*,8a*R*,9*R*)-2,2,4,8,9-pentamethyldecahydro-4,8-methanoazulen-9-ol[4, 5]

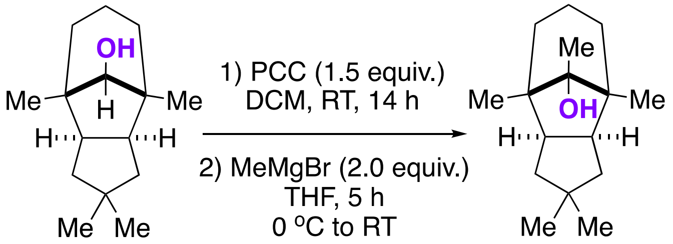

To a flame-dried three-neck round-bottom equipped with a stir bar under N_2 were added apollan-ol (2.00 g, 9.01 mmol), DCM (25.0 mL), and PCC (2.90 g, 13.5 mmol). The reaction mixture was stirred at RT for 14 h. The reaction mixture was quenched with H_2O and extracted into CH_2Cl_2 repeatedly. The combined organic layers were dried with $MgSO_4$, filtered through Celite, and concentrated.

To a flame-dried three-neck round-bottom equipped with a stir bar under N_2 were added crude mixture from the previous reaction (1.98 g, 9.01 mmol) and THF (30.0 mL). The reaction mixture was cooled to 0 °C and 1M methylmagnesium bromide in Et_2O (18.0 mL, 18.0 mmol) was added. The reaction mixture was slowly warmed to RT over 5 h. The reaction mixture was quenched with 1M HCl and extracted into CH_2Cl_2 repeatedly. The combined organic layers were dried with $MgSO_4$, filtered through Celite, and concentrated. The crude residue was purified via gradient column chromatography to provide (3a*R*,4*R*,8*S*,8a*R*,9*R*)-2,2,4,8,9-pentamethyldecahydro-4,8-methanoazulen-9-ol (1.66 g, 78% over two steps).

White solid. ^1H NMR (400 MHz, $CDCl_3$): δ 2.30-2.26 (m, 2H), 1.78-1.44 (m, 6H), 1.40-1.32 (m, 1H), 1.29 -1.24 (m, 2H), 1.21-1.15 (m, 2H), 1.03 (d, J = 1.9 Hz, 6H), 0.91 (s, 3H), 0.88 (s, 6H).

$^{13}C\{^{1}H\}$ NMR (100 MHz, $CDCl_3$): δ 86.1, 50.8, 46.2, 43.9, 40.6, 38.9, 29.4, 26.9, 18.39, 18.35, 16.9. FTMS (ESI) m/z $C_{16}H_{28}O$: calc 236.2140, observed 219.2112 (corresponds to loss of -OH).

(3a*R*,4*S*,8*R*,8a*S*,9*r*)-2,2,4,8-tetramethyldecahydro-4,8-methanoazulen-9-ol

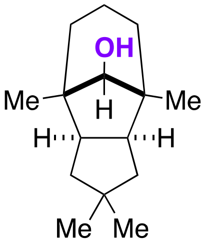

Obtained from Prof. Alex Nickon's chemical reserves.

White solid. ^{1}H NMR (400 MHz, $CDCl_3$): δ 3.26 (d, *J* = 4.8 Hz, 1H), 2.21-2.14 (m, 2H), 1.56-1.34 (m, 6H), 1.22 (d, *J* = 5.3 Hz, 1H), 1.07-0.98 (m, 7H), 0.89 (s, 3H), 0.85 (s, 6H). $^{13}C\{^{1}H\}$ NMR (100 MHz, $CDCl_3$): δ 79.2, 46.7, 43.9, 41.7, 39.6, 31.8, 29.0, 25.5, 21.0, 18.8. FTMS (ESI) m/z $C_{15}H_{26}O$: calc 222.1984, observed 222.1985.

(5*S*,8*R*,9*S*,10*S*,13*S*,14*S*,17*S*)-17-hydroxy-10,13,17-trimethylhexadecahydro-3*H*-cyclopenta[*a*]phenanthren-3-one[5, 6, 7]

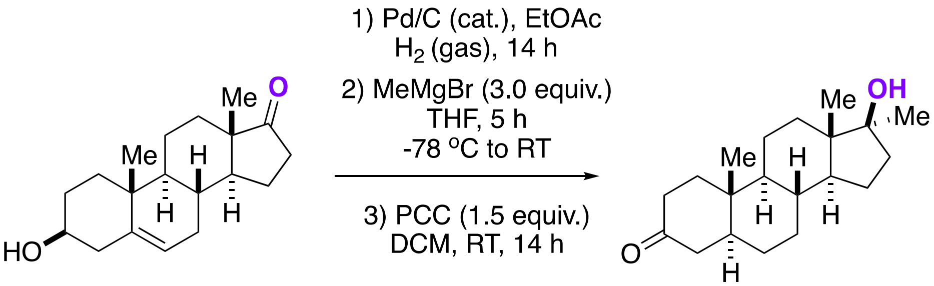

A balloon filled with hydrogen was placed over a round-bottom flask containing a solution of DHEA (5.00 g, 17.4 mmol), and 10% Pd/C (184 mg), and EtOAc (100 mL). The reaction was then stirred at RT for 14 h. The catalyst was removed by filtration through Celite, and the filtrate was concentrated. The crude residue was subjected to the next reaction without purification.

To a flame-dried three-neck round-bottom equipped with a stir bar under N_2 were added crude mixture from the previous reaction (5.03 g, 17.4 mmol) and THF (60.0 mL). The reaction mixture was cooled to -78 °C and 1M methylmagnesium bromide in Et_2O (52.0 mL, 52.0 mmol) was added. The reaction mixture was slowly warmed to RT over 5 h. The reaction mixture was quenched with 1M HCl and extracted into CH_2Cl_2 repeatedly. The combined organic layers were dried with $MgSO_4$, filtered through Celite, and concentrated. The crude residue was purified via gradient

column chromatography to provide (3*S*,8*R*,9*S*,10*S*,13*S*,14*S*,17*S*)-10,13,17-trimethylhexadecahydro-1*H*-cyclopenta[*a*]phenanthrene-3,17-diol (3.72 g, 70% over two steps).

To a flame-dried three-neck round-bottom equipped with a stir bar under N_2 were added (3*S*,8*R*,9*S*,10*S*,13*S*,14*S*,17*S*)-10,13,17-trimethylhexadecahydro-1*H*-cyclopenta[*a*]phenanthrene-3,17-diol (3.72 g, 12.2 mmol), DCM (50.0 mL), and PCC (3.92 g, 18.2 mmol). The reaction mixture was stirred at RT for 14 h. The reaction mixture was quenched with H_2O and extracted into CH_2Cl_2 repeatedly. The combined organic layers were dried with $MgSO_4$, filtered through Celite, and concentrated. The crude residue was purified via gradient column chromatography to provide (8*R*,9*S*,10*S*,13*S*,14*S*,17*S*)-17-hydroxy-10,13,17-trimethylhexadecahydro-3*H* cyclopenta[*a*]phenanthren-3-one (3.14 g, 85%).

White solid. ^{1}H NMR (400 MHz, $CDCl_3$): δ 2.44-2.24 (m, 3H), 2.11-2.00 (m, 2H), 1.85-1.70 (m, 3H), 1.65-1.41 (m, 6H), 1.38-1.15 (m, 10H), 1.03 (s, 3H), 0.94-0.84 (m, 4H), 0.75-0.69 (m, 1H). ^{13}C{^{1}H} NMR (100 MHz, $CDCl_3$): δ 212.1, 81.7, 53.9, 50.6, 46.9, 45.6, 44.8, 39.0, 38.7, 38.2, 36.4, 35.9, 31.7, 31.5, 29.0, 25.9, 23.4, 21.2, 14.1, 11.6. FTMS (ESI) m/z $C_{20}H_{33}O_2$ (M+H$^+$): calc 305.2472, observed 305.2476.

(8R,9S,10R,13S,14S,17R)-17-acetyl-17-hydroxy-10,13-dimethyl-1,2,6,7,8,9,10,11,12,13,14,15,16,17-tetradecahydro-3H-cyclopenta[a]phenanthren-3-one

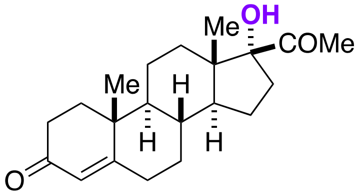

Obtained from Sigma-Aldrich.

Spectral data matches what is reported in literature.[8]

(1*R*,2*S*,5*R*)-2-isopropyl-5-methylcyclohexan-1-ol

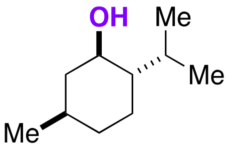

Obtained from Sigma-Aldrich.

Spectral data matches what is reported in literature.[9]

(1*S*,2*S*,5*R*)-2-isopropyl-1,5-dimethylcyclohexan-1-ol[6]

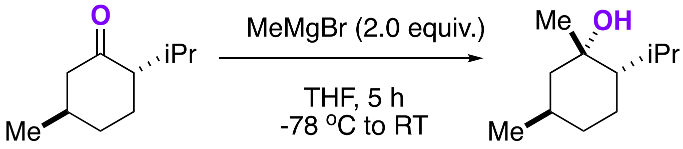

To a flame-dried three-neck round-bottom equipped with a stir bar under N_2 were added menthone (2.00 g, 13.0 mmol) and THF (30.0 mL). The reaction mixture was cooled to -78 °C and 1M methylmagnesium bromide in Et_2O (26.0 mL, 26.0 mmol) was added. The reaction mixture was slowly warmed to RT over 5 h. The reaction mixture was quenched with 1M HCl and extracted into CH_2Cl_2 repeatedly. The combined organic layers were dried with $MgSO_4$, filtered through Celite, and concentrated. The crude residue was purified via gradient column chromatography to provide (1*S*,2*S*,5*R*)-2-isopropyl-1,5-dimethylcyclohexan-1-ol (1.61 g, 73%).

Spectral data matches what is reported in literature.[10]

(1*R*,3a*R*,5*S*,6*R*,7a*S*)-6-acetyl-7a-hydroxy-1-isopropyl-3a-methyloctahydro-1*H*-inden-5-yl acetate[11, 12]

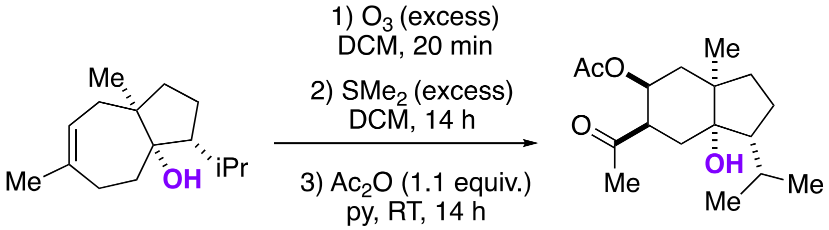

To a flame-dried three-neck round-bottom flask equipped with a stir bar were added caratol (3.00 g, 13.5 mmol) and DCM (25.0 mL). The solution was then cooled to -78 °C, purged with oxygen for 5 minutes, and then a stream of ozone gas was bubbled through the solution for 10 minutes (excess ozone was quenched by bubbling through a saturated aqueous $NaSO_3$). Subsequently, the solution was purged with oxygen for 5 minutes, warmed to RT under N_2, and concentrated. Excess dimethyl sulfide (3.0 mL) was added to the flask and stirred for 14 h. The crude mixture was concentrated and purified via column chromatography on silica gel eluting with EtOAc and hexanes to afford 1-((3*R*,3a*S*,5*R*,6*S*,7a*R*)-3a,6-dihydroxy-3-isopropyl-7a-methyloctahydro-1*H*-inden-5-yl)ethan-1-one (2.50 g, 73%).

To a flame-dried three-neck round-bottom flask equipped with a stir bar under N_2 were added 1-((3*R*,3a*S*,5*R*,6*S*,7a*R*)-3a,6-dihydroxy-3-isopropyl-7a-methyloctahydro-1*H*-inden-5-yl)ethan-1-one (2.50 g, 9.84 mmol), acetic anhydride (10.0 mL), and pyridine (10.0 mL). The reaction mixture was stirred for 21 h and then diluted with CH_2Cl_2 (20 mL). The organic layer was washed with 1M HCl, saturated aq. $NaHCO_3$, and H_2O. The organic layer was dried over $MgSO_4$, filtered through Celite, and concentrated. The crude residue was purified via gradient column chromatography on silica gel eluting with EtOAc and hexanes to provide (1R,3aR,5S,6R,7aS)-6-acetyl-7a-hydroxy-1-isopropyl-3a-methyloctahydro-1H-inden-5-yl acetate (2.68 g, 92%) as a white solid.

White solid. ^1H NMR (400 MHz, $CDCl_3$): δ 5.17-5.11 (m, 1H), 2.89-2.82 (m, 1H), 2.20-2.15 (m, 4H), 2.05-1.86 (m, 5H), 1.81-1.72 (m, 2H), 1.67-1.44 (m, 5H), 1.43-1.34 (m, 1H), 1.10-1.07 (m, 6H), 0.96 (d, *J* = 6.6 Hz, 3H). $^{13}C\{^1H\}$ NMR (100 MHz, $CDCl_3$): δ 208.4, 170.1, 81.8, 70.6, 53.0, 49.2, 47.8, 40.5, 36.9, 33.7, 29.8, 29.1, 25.7, 23.2, 22.7, 21.2, 19.7. FTMS (ESI) m/z $C_{17}H_{28}O_4$: calc 296.1988, observed 296.1985.

<u>(1*R*,3a*R*,5*S*,6*R*,7a*S*)-6-acetyl-7a-hydroxy-1-isopropyl-3a-methyloctahydro-1*H*-inden-5-yl 4-methylbenzenesulfonate[12, 13]</u>

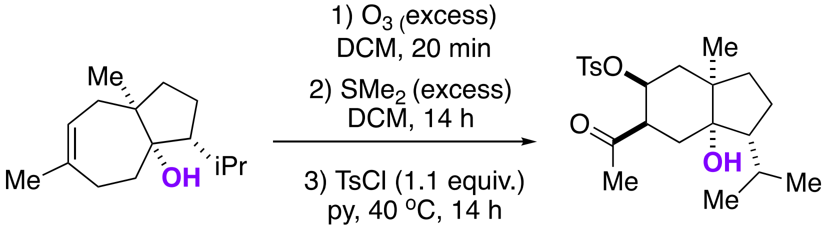

To a flame-dried three-neck round-bottom flask equipped with a stir bar were added caratol (3.00 g, 13.5 mmol) and DCM (25.0 mL). The solution was then cooled to -78 °C, purged with oxygen for 5 minutes, and then a stream of ozone gas was bubbled through the solution for 10 minutes (excess ozone was quenched by bubbling through a saturated aqueous $NaSO_3$). Subsequently, the solution was purged with oxygen for 5 minutes, warmed to RT under N_2, and concentrated. Excess dimethyl sulfide (3.0 mL) was added to the flask and stirred for 14 h. The crude mixture was concentrated and purified via column chromatography on silica gel eluting with EtOAc and hexanes to afford 1-((3*R*,3a*S*,5*R*,6*S*,7a*R*)-3a,6-dihydroxy-3-isopropyl-7a-methyloctahydro-1*H*-inden-5-yl)ethan-1-one (2.50 g, 73%).

To a flame-dried three-neck round-bottom flask equipped with a stir bar under N_2 were added 1-((3*R*,3a*S*,5*R*,6*S*,7a*R*)-3a,6-dihydroxy-3-isopropyl-7a-methyloctahydro-1*H*-inden-5-yl)ethan-1-one (2.50 g, 9.84 mmol), tosyl chloride (2.10 g, 11.0 mmol), and pyridine (10.0 mL). The reaction mixture was stirred for 21 h at 40 °C and then diluted with CH_2Cl_2 (20.0 mL). The organic layer was washed with 1M HCl, saturated aq. $NaHCO_3$, and H_2O. The organic layer was dried over $MgSO_4$, filtered through Celite, and concentrated. The crude residue was purified via gradient column chromatography on silica gel eluting with EtOAc and hexanes to provide (1R,3aR,5S,6R,7aS)-6-acetyl-7a-hydroxy-1-isopropyl-3a-methyloctahydro-1H-inden-5-yl 4-methylbenzenesulfonate (3.73 g, 93%) as a white solid.

White solid. ^1H NMR (400 MHz, $CDCl_3$): δ 7.69 (d, *J* = 8.3 Hz, 2H), 7.30 (d, *J* = 8.0 Hz, 2H), 4.82-4.75 (m, 1H), 2.88 (ddt, *J* = 20.1, 13.6, 6.8 Hz, 1H), 2.41 (s, 3H), 2.07-2.02 (m, 1H), 2.00 (s, 3H), 1.93-1.80 (m, 4H), 1.73-1.41 (m, 6H), 1.36-1.30 (m, 2H), 1.00-0.98 (m, 6H), 0.90 (m, 3H). $^{13}C\{1H\}$ NMR (100 MHz, CDCl3): δ 207.3, 144.8, 133.8, 129.7, 127.8, 81.1, 79.3, 53.5, 52.6, 49.1, 47.9, 41.5, 36.7, 33.6, 29.8, 29.6, 25.6, 23.0, 22.5, 21.7, 19.4. FTMS (ESI) m/z $C_{21}H_{32}O_5S$ $(M+H^+)$: calc 409.2040, observed 409.2046.

2-((1*R*,2*R*,4a*S*,8a*S*)-2-hydroxy-2,5,5,8a-tetramethyldecahydronaphthalen-1-yl)ethyl acetate

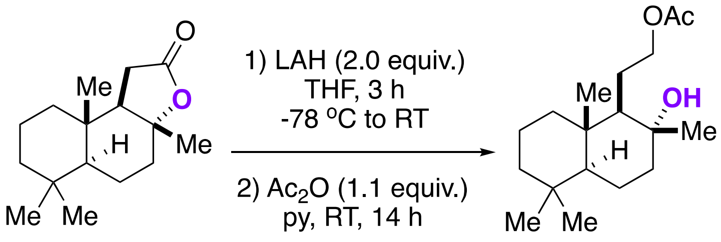

To a flame-dried three-neck round-bottom equipped with a stir bar under N_2 were added (3a*R*,5a*S*,9a*S*,9b*R*)-3a,6,6,9a-tetramethyldecahydronaphtho[2,1-*b*]furan-2(1*H*)-one (3.00 g, 12.0 mmol) and THF (30.0 mL). The reaction mixture was cooled to -78 °C and 1M LAH in THF (24.0 mL, 24.0 mmol) was added dropwise. The reaction mixture was slowly warmed to RT over 3 h. The reaction mixture was quenched with 1M HCl and extracted into CH_2Cl_2 repeatedly. The combined organic layers were dried with $MgSO_4$, filtered through Celite, and concentrated. The crude residue was purified via gradient column chromatography to provide (1*R*,2*R*,4a*S*,8a*S*)-1-(2-hydroxyethyl)-2,5,5,8a-tetramethyldecahydronaphthalen-2-ol (2.80 g, 92%).

To a flame-dried three-neck round-bottom flask equipped with a stir bar under N2 were added (1*R*,2*R*,4a*S*,8a*S*)-1-(2-hydroxyethyl)-2,5,5,8a-tetramethyldecahydronaphthalen-2-ol (2.80 g, 11.0 mmol), acetic anhydride (10.0 mL), and pyridine (10.0 mL). The reaction mixture was stirred for 14 h and then diluted with CH_2Cl_2 (20.0 mL). The organic layer was washed with 1M HCl, saturated aq. $NaHCO_3$, and H_2O. The organic layer was dried over $MgSO_4$, filtered through Celite, and concentrated. The crude residue was purified via gradient column chromatography on silica gel eluting with EtOAc and hexanes to provide 2-((1*R*,2*R*,4a*S*,8a*S*)-2-hydroxy-2,5,5,8a-tetramethyldecahydronaphthalen-1-yl)ethyl acetate (3.10 g, 95%).

White solid. ^{1}H NMR (400 MHz, $CDCl_3$) δ 4.16-4.05 (m, 2H), 2.04 (s, 3H), 1.88 (dt, *J* = 12.2, 3.1 Hz, 1H), 1.78-1.52 (m, 6H), 1.46-1.34 (m, 3H), 1.31-1.20 (m, 1H), 1.17-1.07 (m, 5H), 0.93-0.86 (m, 5H), 0.78 (s, 6H). ^{13}C{^{1}H} NMR (100 MHz, $CDCl_3$): δ 171.2, 73.6, 66.7, 58.1, 56.1, 44.4, 42.0, 39.7, 38.8, 33.5, 33.3, 24.6, 24.0, 21.5, 21.2, 20.5, 18.5, 15.4. FTMS (ESI) m/z $C_{18}H_{32}O_3$: calc 296.2351, observed 279.2319 (corresponds to loss of -OH).

2-((1*R*,2*S*)-2-hydroxy-2,6,6-trimethylcyclohexyl)ethyl acetate[13, 14]

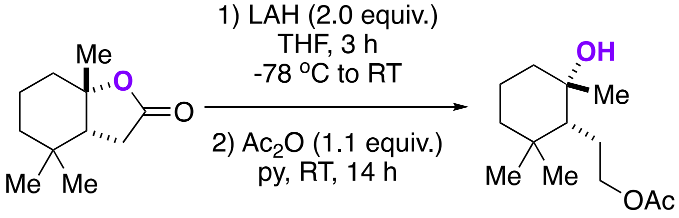

To a flame-dried three-neck round-bottom equipped with a stir bar under N_2 were added (3a*R*,7a*S*)-4,4,7a-trimethylhexahydrobenzofuran-2(3*H*)-one (2.50 g, 13.7 mmol) and THF (30.0 mL). The reaction mixture was cooled to -78 °C and 1M LAH in THF (27.5 mL, 27.5 mmol) was added

dropwise. The reaction mixture was slowly warmed to RT over 3 h. The reaction mixture was quenched with 1M HCl and extracted into CH_2Cl_2 repeatedly. The combined organic layers were dried with $MgSO_4$, filtered through Celite, and concentrated. The crude residue was purified via gradient column chromatography to provide (1*S*,2*R*)-2-(2-hydroxyethyl)-1,3,3-trimethylcyclohexan-1-ol (2.48 g, 97%).

To a flame-dried three-neck round-bottom flask equipped with a stir bar under N_2 were added (1*S*,2*R*)-2-(2-hydroxyethyl)-1,3,3-trimethylcyclohexan-1-ol (2.48 g, 13.3 mmol), acetic anhydride (10.0 mL), and pyridine (10.0 mL). The reaction mixture was stirred for 14 h and then diluted with CH_2Cl_2 (20 mL). The organic layer was washed with 1M HCl, saturated aq. $NaHCO_3$, and H_2O. The organic layer was dried over $MgSO_4$, filtered through Celite, and concentrated. The crude residue was purified via gradient column chromatography on silica gel eluting with EtOAc and hexanes to provide 2-((1*R*,2*S*)-2-hydroxy-2,6,6-trimethylcyclohexyl)ethyl acetate (2.74 g, 90%).

Clear oil. ^{1}H NMR (400 MHz, $CDCl_3$): δ 4.07 (dm, J = 17.9, 2H), 2.05 (s, 3H), 1.89-1.62 (m, 4H), 1.47-1.35 (m, 3H), 1.21-1.13 (m, 4H), 0.97 (s, 4H), 0.90-0.88 (m, 4H). ^{13}C{^{1}H} NMR (100 MHz, $CDCl_3$): δ 170.9, 72.1, 66.1, 49.9, 41.6, 41.0, 34.2, 31.8, 30.6, 24.6, 21.2, 20.8, 18.0. FTMS (ESI) m/z $C_{13}H_{24}O_3$: calc 228.1725, observed 211.1694 (corresponds to loss of -OH).

1-(4-chlorophenyl)cycloheptane-1-ol

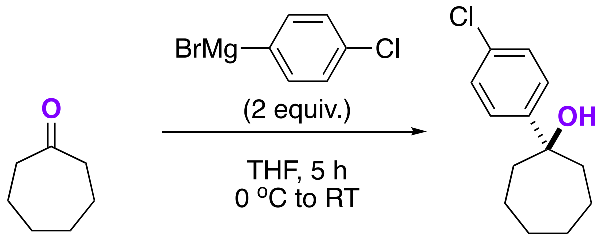

To a flame-dried three-neck round-bottom equipped with a stir bar under N_2 were added a suspension of cycloheptanone (2.00 g, 17.9 mmol) in THF (30.0 mL). The reaction mixture was cooled to 0 °C and 1M 4-chlorophenylmagnesium bromide in Et_2O (35.0 mL, 35.0 mmol) was added. The reaction mixture was slowly warmed to RT over 5 h. The reaction mixture was quenched with 1M HCl and extracted into CH_2Cl_2 repeatedly. The combined organic layers were dried with $MgSO_4$, filtered through Celite, and concentrated. The crude residue was purified via gradient column chromatography to provide 1-(4-chlorophenyl)cycloheptane-1-ol (2.10 g, 53%).

Spectral data matches what is reported in literature.[15]

1-(4-chlorophenyl)cyclopentane-1-ol

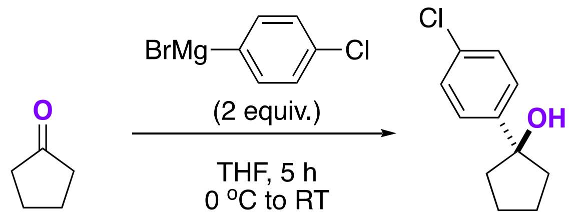

To a flame-dried three-neck round-bottom equipped with a stir bar under N_2 were added a suspension of cyclopentanone (2.00 g, 23.8 mmol) in THF (30.0 mL). The reaction mixture was cooled to 0 °C and 1M 4-chlorophenylmagnesium bromide in Et_2O (45.0 mL, 45.0 mmol) was added. The reaction mixture was slowly warmed to RT over 5 h. The reaction mixture was quenched with 1M HCl and extracted into CH_2Cl_2 repeatedly. The combined organic layers were dried with $MgSO_4$, filtered through Celite, and concentrated. The crude residue was purified via gradient column chromatography to provide 1-(4-chlorophenyl)cyclopentane-1-ol (1.90 g, 41%).

Spectral data matches what is reported in literature. [16]

(S)- and (R)-1-methyl-1,2,3,4-tetrahydronaphthalen-1-ol

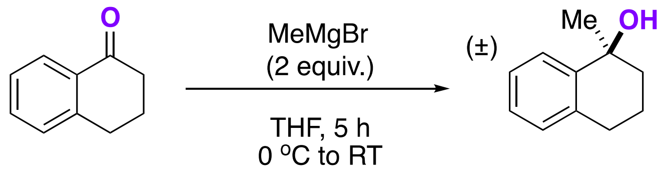

To a flame-dried three-neck round-bottom equipped with a stir bar under N_2 were added a suspension of 1-tetralone (2.00 g, 13.7 mmol) in THF (30.0 mL). The reaction mixture was cooled to 0 °C and 3M methylmagnesium bromide in Et_2O (10.0 mL, 30.0 mmol) was added. The reaction mixture was slowly warmed to RT over 5 h. The reaction mixture was quenched with 1M HCl and extracted into CH_2Cl_2 repeatedly. The combined organic layers were dried with $MgSO_4$, filtered through Celite, and concentrated. The crude residue was purified via gradient column chromatography to provide 1-methyl-1,2,3,4-tetrahydronaphthalen-1-ol (1.55 g, 70%).

Spectral data matches what is reported in literature.[16]

(S)- and (R)-1-phenyl-2,3-dihydro-1H-inden-1-ol

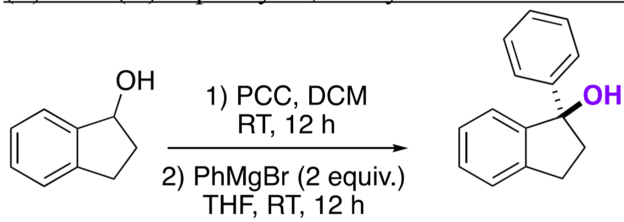

To a flame-dried three-neck round-bottom equipped with a stir bar under N_2 were added indanol (1.20 g, 9.01 mmol), DCM (25.0 mL), and PCC (2.90 g, 13.5 mmol). The reaction mixture was stirred at RT for 14 h. The reaction mixture was then filtered through Celite and concentrated.

To a flame-dried three-neck round-bottom equipped with a stir bar under N_2 were added crude mixture from the previous reaction (1.98 g, 9.01 mmol) and THF (30.0 mL). The reaction mixture was cooled to 0 °C and 1M phenylmagnesium bromide in Et_2O (18.0 mL, 18.0 mmol) was added. The reaction mixture was slowly warmed to RT over 5 h. The reaction mixture was quenched with 1M HCl and extracted into CH_2Cl_2 repeatedly. The combined organic layers were dried with $MgSO_4$, filtered through Celite, and concentrated. The crude residue was purified via gradient column chromatography to provide (S)- and (R)-1-phenyl-2,3-dihydro-1H-inden-1-ol.

Spectral data matches what is reported in literature[17]

Fenchyl alcohol

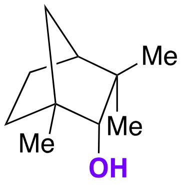

Obtained from Sigma-Aldrich.

Spectral data matches what is reported in literature.[18]

9-(4-chlorophenyl)bicyclo[3.3.1]nonan-9-ol

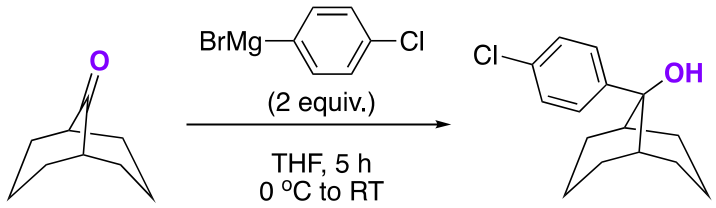

To a flame-dried three-neck round-bottom equipped with a stir bar under N_2 were added a suspension of bicyclo[3.3.1]nonan-9-one (1.00 g, 7.25 mmol) in THF (20.0 mL). The reaction mixture was cooled to 0 °C and 1M 4-chlorophenylmagnesium bromide in Et_2O (15.0 mL, 15.0 mmol) was added. The reaction mixture was slowly warmed to RT over 5 h. The reaction mixture was quenched with 1M HCl and extracted into CH_2Cl_2 repeatedly. The combined organic layers were dried with $MgSO_4$, filtered through Celite, and concentrated. The crude residue was purified via gradient column chromatography to provide 9-(4-chlorophenyl)bicyclo[3.3.1]nonan-9-ol (1.20 g, 66%).

Clear Oil. ^{1}H NMR (400 MHz, $CDCl_3$): δ 7.51 (d, J = 8.70 Hz, 2H) 2.51 (s, 1H), 2.44-2.34 (m, 2H), 2.12-2.07 (m, 2H), 1.99-1.63 (m, 9H), 1.38-1.33 (m, 1H); ^{13}C{1H} NMR (100 MHz, CDCl3):

δ 143.55, 133.00, 128.85, 127.17, 74.02, 35.43, 29.64, 27.18, 20.98, 20.55. FTMS (ESI) m/z $C_{15}H_{19}O$: calc 250.1124, observed 233.1086 (corresponds to loss of -OH).

Product Characterization Data

(1S,3R)- and (1*R*,3*S*)-1-(4-chlorophenyl)-3-fluoro-3-methylcyclohexan-1-ol (compound **6**)

(±)

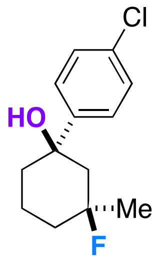

The reaction was run according to the general procedure, and the major diastereomer was isolated. The crude material was subjected to gradient column chromatography on silica gel eluting with hexanes and EtOAc, followed by HPLC purification.

Regiochemical and stereochemical assignments were made on the basis of 1) chemical shift of the ^{19}F NMR signal, indicative of a tertiary fluoride on a cyclohexane ring, 2) disappearance of the diagnostic tertiary proton signal (1.88 ppm) in the ^{1}H NMR spectrum, 3) diagnostic methyl ^{1}H signal shift (1.40 ppm) and coupling constant (J = 22.10 Hz), 4) -OH ^{1}H signal has a chemical shift (3.62 ppm) and coupling (J = 19.1 Hz) indicative of intramolecular hydrogen bonding, 5) absence of a downfield ^{1}H signal with approximately $^{2}J_{HF}$ = 50 Hz coupling suggest no secondary or primary fluorides present, 6) chemical shift and splitting in the ^{19}F NMR spectrum that indicates F_{ax} (for example: the F_{ax} and F_{eq} diastereomers of 1-fluoro-1-methyl-4-tbutylcyclohexane have a chemical shift of -154 ppm and -127 ppm, respectively), and 7) identification of distinguishable peaks (quaternary, C-F, etc.) in the ^{13}C NMR spectrum are in agreement with the regioisomer assignment ($^{2}J_{CF}$- and $^{3}J_{CF}$-coupling and chemical shifts).

^{1}H NMR (400 MHz, $CDCl_3$): 7.45 (m, 2H), 7.31 (m, 2H), 3.62 (d, J = 19.1 Hz, 1H), 2.16-2.01 (m, 3H), 1.90-1.69 (m, 4H), 1.40 (m, 4H). ^{13}C{^{1}H} NMR (100 MHz, $CDCl_3$): δ 146.6, 132.6, 128.4, 126.3, 97.9 (d, J = 141.9 Hz), 73.2, 48.0, 47.8, 37.8, 35.9, 35.7, 28.5, 28.3, 17.5. ^{19}F NMR (282 MHz, $CDCl_3$): δ -145.5 - (-146.0) (m). FTMS (ESI) m/z $C_{13}H_{16}OClF$: calc 242.0874, observed 242.0861.

(2*R*,5*S*)-3-fluoro-4,4,8-trimethyltricyclo[6.3.1.0^{2,5}]dodecan-1-ol (compound **7**)

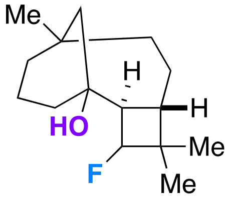

The reaction was run according to the general procedure, and the major diastereomer was isolated. The crude material was subjected to gradient column chromatography on silica gel eluting with hexanes and EtOAc, followed by HPLC purification.

Regiochemical assignment was made on the basis of 1) chemical shift of the ^{19}F NMR signal, indicative of a secondary fluoride on a cyclobutane ring, 2) ^{1}H signals of the adjacent dimethyl group are separate (in comparison to the starting material) and shifted downfield (1.04 ppm), 3) the chemical shift and coupling constant ($^{2}J_{HF}$ = 50.1 Hz) of the ^{1}H signal at 4.38 ppm are indicative of a proton with a geminal fluoride, 4) ^{13}C signal of the carbon attached to fluorine has a $^{1}J_{CF}$ = 225.6 Hz, which is characteristic of a fluorocyclobutane (for example: the $^{1}J_{CF}$ of fluorocyclohexane, fluorocyclopentane, and fluorocyclobutane are 170, 174, and 215 Hz, respectively), and 5) -OH ^{1}H signal (taken in dry $CHCl_3$) at 1.88 ppm is shifted downfield relative to the starting material (indicative of hydrogen bonding), and 6) identification of distinguishable peaks (quaternary, C-F, etc.) in the ^{13}C NMR spectrum are in agreement with the regioisomer assignment ($^{2}J_{CF}$- and $^{3}J_{CF}$-coupling and chemical shifts).

^{1}H NMR (400 MHz, $CDCl_3$): δ 4.38 (dm, J = 50.1 Hz, 1H), 2.17-1.87 (m, 4H), 1.73-1.46 (m, 6H), 1.38-1.14 (m, 5H), 1.02 (s, 3H), 0.98 (s, 3H), 0.92 (s, 3H). ^{13}C{^{1}H} NMR (100 MHz, $CDCl_3$): δ 93.70 (dd, J = 225.6, 3.77 Hz) , 72.0, 71.8, 44.9, 43.19, 43.18, 43.16, 38.22, 38.18, 36.8, 35.32, 35.30, 34.63, 34.55, 34.4, 33.08, 33.06, 32.4, 30.5, 26.4, 26.3, 26.2, 26.1, 21.98, 21.95, 20.8. ^{19}F NMR (282 MHz, $CDCl_3$): δ -190.4 - (-190.8) (m). FTMS (ESI) m/z $C_{15}H_{25}OF$: calc 240.1889, observed 240.1882.

(3a*R*,4*R*,8*S*,8a*R*,9*S*)-1-fluoro-2,2,4,8,9-pentamethyldecahydro-4,8-methanoazulen-9-ol (compound **8**)

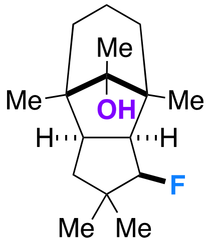

The reaction was run according to the general procedure, and the major diastereomer was isolated. The crude material was subjected to gradient column chromatography on silica gel eluting with hexanes and EtOAc, followed by HPLC purification.

Regiochemical and stereochemical assignments were made on the basis of 1) chemical shift of the ^{19}F NMR signal, indicative of a secondary fluoride on a cyclopentane ring, and coupling (doublet of doublets) agree with the proposed regioisomer, 2) -OH ^{1}H signal (taken in dry $CHCl_3$) at 3.43 ppm is shifted downfield relative to the starting material (indicative of hydrogen bonding), 3) diagnostic tertiary ^{1}H signal (alpha to the designated fluorine) signal (2.58 ppm) is shifted downfield, 4) one of the diagnostic dimethyl ^{1}H signal shift (1.07 ppm) and coupling constant (J = 1.47 Hz), 5) the absence of a strong interaction within the ^{1}H-^{1}H NOESY and ^{19}F-^{1}H HOESY spectrum between the fluorine/geminal proton to fluorine and methyl at the alcohol bridgehead, suggesting the assigned regio- and stereoisomer, and 6) identification of distinguishable peaks (quaternary, C-F, etc.) in the ^{13}C NMR spectrum are in agreement with the regioisomer assignment ($^{2}J_{CF}$- and $^{3}J_{CF}$-coupling and chemical shifts).

^{1}H NMR (400 MHz, $CDCl_3$): δ 4.40 (dm, *J* = 50.4 Hz, 1H), 2.65-2.56 (m, 1H), 2.29-2.20 (m, 1H), 1.95-1.87 (m, 1H), 1.82-1.58 (m, 3H) 1.42-1.28 (m, 4H), 1.06-1.05 (m, 6H) 0.97-0.93 (m, 10H)$^{13}C\{^{1}H\}$ NMR (100 MHz, $CDCl_3$): δ 96.5 (d, *J* = 176.7 Hz), 84.9 (d, *J* = 8.2 Hz), 51.3, 51.2, 51.1, 45.17, 45.16, 43.9, 43.5, 40.6, 35.3, 35.2, 29.3, 26.8, 25.3, 25.1, 18.2, 16.3, 12.23, 12.18. ^{19}F NMR (282 MHz, $CDCl_3$): δ -181.2 (dd, *J* = 50.4, 20.9 Hz). FTMS (ESI) m/z $C_{16}H_{27}OF$: calc 254.2046, observed 235.2056 (corresponds to loss of fluorine).

(3a*S*,4*R*,8*S*,8a*R*,9*R*)-5-fluoro-2,2,4,8-tetramethyldecahydro-4,8-methanoazulen-9-ol (compound **9**)

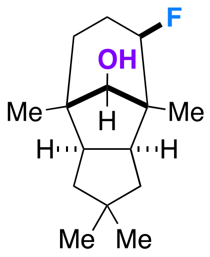

The reaction was run according to the general procedure (with the exception: 1.2 equiv. of Selectfluor used), and the major diastereomer was isolated. The crude material was subjected to gradient column chromatography on silica gel eluting with hexanes and EtOAc, followed by HPLC purification.

Stereochemical and regiochemical assignment reasoning discussed within manuscript.

^{1}H NMR (400 MHz, $CDCl_3$): δ 4.64 (dm, *J* = 50.3 Hz, 1H), 3.44-3.41 (t, *J* = 4.5 Hz, 1H), 2.48 (dd, *J* = 19.54, 9.94 Hz, 1H), 2.23-2.16 (m, 1H), 1.94-1.88 (m, 1H), 1.74-1.59 (m, 2H), 1.45-1.38 (m, 2H), 1.34 (d, *J* = 4.3 Hz, 1H), 1.20-1.11 (m, 1H), 1.07, 1.03 (m, 7H), 0.92-0.87 (m, 7H). $^{13}C\{^{1}H\}$ NMR (100 MHz, $CDCl_3$): δ 95.80, 95.77, 94.08, 94.05, 81.14, 81.11, 81.06, 81.03, 47.2, 46.5, 46.4, 44.1, 43.7, 41.31, 41.30, 40.8, 39.6, 30.8, 30.7, 29.0, 25.7, 25.5, 25.4, 19.84, 19.81, 16.7, 16.6. ^{19}F NMR (282 MHz, $CDCl_3$): δ -194.2 - (-195.2) (m). FTMS (ESI) m/z $C_{15}H_{25}OF$: calc 240.1889, observed 240.1901.

(5*S*,8*R*,9*S*,10*S*,12*R*,13*S*,14*S*,17*S*)-12-fluoro-17-hydroxy-10,13,17-trimethylhexadecahydro-3*H*-cyclopenta[*a*]phenanthren-3-one (compound **10**)

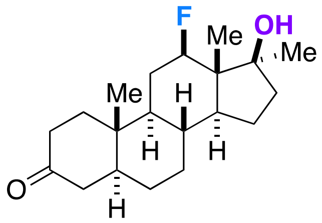

The reaction was run according to the general procedure, and the major diastereomer was isolated. The crude material was subjected to gradient column chromatography on silica gel eluting with hexanes and EtOAc, followed by HPLC purification.

Regiochemical and stereochemical assignments were made on the basis of 1) chemical shift of the ^{19}F NMR signal, indicative of a secondary fluoride on a cyclohexane ring, 2) the C18 methyl ^{1}H signal (0.88 → 0.99 ppm) is shifted downfield relative to the starting material, 3) the chemical shift and coupling constant (doublet of doublet of doublets, J = 50.2, 11.2, 5.0 Hz) of the ^{1}H signal at 4.63 ppm are indicative of a proton with a geminal fluoride and corroborates the designated regioisomer's chemical environment, 4) strong interaction within the ^{1}H-^{1}H NOESY spectrum between the geminal proton to the fluoride and methyl upon the C17 carbon supports evidence for the beta-fluoro configuration, 5) the absence of a strong interaction within the ^{19}F-^{1}H NOESY spectrum between the fluorine and methyl upon the C17 carbon infer the beta-fluoro configuration, and 6) ^{13}C signal associated with F-C; $^{1}J_{CF}$ = 179.2 Hz is indicative of a secondary fluoride on a cyclohexane ring.

^{1}H NMR (400 MHz, $CDCl_3$): δ 4.63 (ddd, J = 50.2, 11.2, 5.0 Hz, 1H), 2.47-2.22 (m, 3H), 2.14-1.87 (m, 4H), 1.77-1.13 (m, 13H), 1.06 (s, 3H), 0.99 (s, 3H), 0.91-0.77 (s, 4H). ^{13}C{^{1}H} NMR (100 MHz, $CDCl_3$): δ 211.4, 95.4 (d, J = 179.2 Hz), 81.6, 52.1, 52.0, 50.3, 50.1, 49.3, 49.2, 46.6, 44.6, 38.6, 38.1, 35.9, 35.3, 30.9, 28.8, 27.9, 27.7, 26.3, 23.3, 11.6, 9.1. ^{19}F NMR (282 MHz, $CDCl_3$): δ -183.5 (d, J = 50.3 Hz). FTMS (ESI) m/z $C_{20}H_{32}O_2F$ (M+H$^+$): calc 323.2378, observed 323.2381.

(8R,9S,10R,12S,13S,14S,17R)-17-acetyl-12-fluoro-17-hydroxy-10,13-dimethyl 1,2,6,7,8,9,10,11,12,13,14,15,16,17-tetradecahydro-3H-cyclopenta[a]phenanthren-3-one (compound **11**)

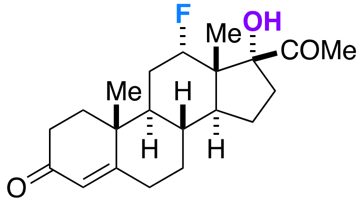

The reaction was run according to the general procedure (with the exception: 0.0 equiv. of $NaHCO_3$ used), and the major diastereomer was isolated. The crude material was subjected to gradient column chromatography on silica gel eluting with hexanes and EtOAc, followed by HPLC purification.

Regiochemical and stereochemical assignments were made on the basis of 1) chemical shift of the ^{19}F NMR signal, indicative of a secondary fluoride on a cyclohexane ring, 2) -OH ^{1}H signal (3.12 ppm) is shifted downfield relative to the starting material (indicative of hydrogen bonding, thus alpha-fluoro configuration), 3) ^{1}H signal with the coupling constant (doublet of doublet of doublets, J = 49.52, 11.09, 5.16 Hz) at 4.96 ppm are indicative of a proton with a geminal fluoride and corroborates the designated regioisomer's chemical environment, 4) the absence of a strong interaction within the ^{1}H-^{1}H NOESY spectrum between the geminal proton to the fluoride and -OH infers the alpha-fluoro configuration, and 5) identification of distinguishable peaks (quaternary, C-F, etc.) in the ^{13}C NMR spectrum are in agreement with the regioisomer assignment ($^{2}J_{CF}$- and $^{3}J_{CF}$-coupling and chemical shifts).

^{1}H NMR (400 MHz, $CDCl_3$): δ 5.75 (s, 1H), 4.96 (ddd, J = 49.52, 11.09, 5.16 Hz, 1H), 3.12 (s, 1H), 2.77-2.66 (m, 1H), 2.48-2.28 (m, 7H), 2.05-1.85 (m, 4H), 1.76-1.67 (m, 2H), 1.61 (s, 3H), 1.59-1.48 (m, 3H), 1.21 (s, 3H), 1.12-1.01 (m, 2H), 0.88 (s, 3H). $^{13}C\{^{1}H\}$ NMR (100 MHz, $CDCl_3$): δ 211.87, 199.37, 169.97, 169.96, 124.29, 92.04, 90.27, 89.00, 88.99, 53.21, 53.04, 51.65, 51.56, 48.74, 48.68, 38.40, 38.39, 35.64, 35.20, 34.28, 34.27, 33.80, 32.62, 31.24, 31.22, 27.17. 26.98, 26.87, 26.82, 23.49, 23.47, 17.23, 9.79, 9.75; ^{19}F NMR (282 MHz, $CDCl_3$): δ -176.37 – (-176.55) (m). FTMS (ESI) m/z $C_{13}H_{23}O_3F$ (M+H$^+$): calc 349.2179, observed 349.2163.

<u>(1*R*,2*R*,5*R*)-2-(2-fluoropropan-2-yl)-5-methylcyclohexan-1-ol</u> (compound **12**)

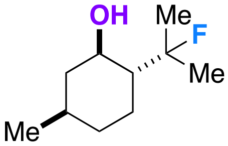

The reaction was run according to the general procedure (with the exception: 1.2 equiv. of Selectfluor used), and the major diastereomer was isolated. The crude material was subjected to gradient column chromatography on silica gel eluting with hexanes and EtOAc, followed by HPLC purification.

Spectral data matches what is reported in literature.[19]

<u>(1*S*,2*S*,5*S*)-5-fluoro-2-isopropyl-1,5-dimethylcyclohexan-1-ol</u> (compound **13**)

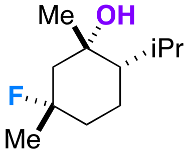

The reaction was run according to the general procedure, and the major diastereomer was isolated. The crude material was subjected to gradient column chromatography on silica gel eluting with hexanes and EtOAc, followed by HPLC purification.

Regiochemical and stereochemical assignments were made on the basis of 1) the chemical shift of the ^{19}F NMR signal, highly indicative of an axial tertiary fluoride on a cyclohexane ring, 2) disappearance of the diagnostic tertiary proton signal (1.4 ppm) in the ^{1}H NMR spectrum, 3) absence of a downfield ^{1}H signal with approximately 50 Hz coupling suggests no secondary or primary fluorides present, 4) diagnostic isopropyl ^{1}H signals are still present at the approximately 0.93 ppm methyl, 5) methyl ^{1}H signal alpha to the fluoride has a drastic shift (compared to the starting material) to 1.51 ppm and coupling constant of J = 23.3 Hz, and 6) identification of distinguishable peaks (quaternary, C-F, etc.) in the ^{13}C NMR spectrum are in agreement with the regioisomer assignment ($^{2}J_{CF}$- and $^{3}J_{CF}$-coupling and chemical shifts).

^{1}H NMR (400 MHz, $CDCl_3$): δ 2.14-2.07 (m, 1H), 1.95-1.92 (m, 1H), 1.87 -1.73(m, 2H), 1.54-1.43 (m, 6H), 1.29 (s, 3H), 1.13 (dm, J = 11.2, 1H), 0.96 (dm, J = 7.0 Hz, 3H), 0.90 (dm, J = 6.9 Hz, 3H), 0.85 (s, 1H). $^{13}C\{^{1}H\}$ NMR (100 MHz, $CDCl_3$): δ 96.1 (d, J = 167.0 Hz), 74.2 (d, J =

13.1 Hz), 51.9, 51.8, 50.6, 38.3 (d, J = 20.4 Hz), 30.5, 26.5, 26.3, 26.0, 24.3, 19.7 (d, J = 10.7 Hz), 18.7. ^{19}F NMR (282 MHz, $CDCl_3$): δ -118.8 - (-119.3) (m). FTMS (ESI) m/z: 188.1576 calc, observed 172.16 (corresponds to loss of –OH + H^+).

(1*S*,3a*R*,5*S*,6*R*,7a*S*)-6-acetyl-1-(2-fluoropropan-2-yl)-7a-hydroxy-3a-methyloctahydro-1*H*-inden-5-yl acetate (compound **14**)

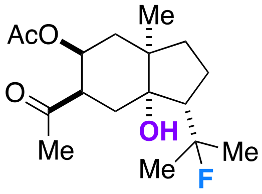

The reaction was run according to the general procedure, and the major diastereomer was isolated. The crude material was subjected to gradient column chromatography on silica gel eluting with hexanes and EtOAc, followed by HPLC purification.

Regiochemical assignment was made on the basis of 1) the chemical shift of the ^{19}F NMR signal, indicative of a tertiary fluoride, 2) ^{19}F signal coupling (dqd, J = 44.0, 21.9, 11.2 Hz) agrees with the chemical environment of the regioisomer, 3) absence of a downfield ^{1}H signal with approximately 50 Hz coupling implies no secondary or primary fluorides are present, 4) diagnostic ^{1}H methyl signal shift from isopropyl group (1.56 ppm) and coupling constant (J = 22.04 Hz) for the isopropyl group, and 5) identification of distinguishable peaks (quaternary, C-F, etc.) in the ^{13}C NMR spectrum are in agreement with the regioisomer assignment ($^{2}J_{CF}$- and $^{3}J_{CF}$-coupling and chemical shifts).

^{1}H NMR (400 MHz, $CDCl_3$): δ 5.16 (td, J = 11.4, 4.7 Hz, 1H), 2.84-2.77 (m, 1H), 2.56-2.45 (m, 1H), 2.30 (dd, J = 13.9, 3.7 Hz, 1H), 2.17-2.12 (m, 4H), 1.97 (s, 3H), 1.95-1.80 (m, 2H), 1.72-1.63 (m, 2H), 1.58-1.50 (m, 6H), 1.45-1.37 (m, 3H), 1.11 (s, 3H). ^{13}C{^{1}H} NMR (100 MHz, $CDCl_3$): δ 208.2, 170.1, 99.8 (d, J = 164.3 Hz), 82.0, 70.3, 53.3, 50.0, 49.8, 48.0, 40.2, 36.8, 33.93, 33.90, 28.8, 28.3, 28.1, 27.0, 26.7, 22.9, 22.8, 21.2, 19.5. ^{19}F NMR (282 MHz, $CDCl_3$): -142.0 (dqd, J = 44.0, 21.9, 11.2 Hz). FTMS (ESI) m/z $C_{17}H_{27}O_4F$ (M+H^+): calc 315.1900, observed 315.1966.

(1*S*,3a*R*,5*S*,6*R*,7a*S*)-6-acetyl-1-(2-fluoropropan-2-yl)-7a-hydroxy-3a-methyloctahydro-1*H*-inden-5-yl 4-methylbenzenesulfonate (compound **15**)

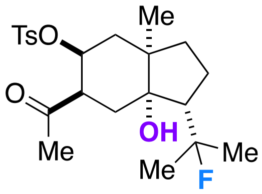

The reaction was run according to the general procedure, and the major diastereomer was isolated. The crude material was subjected to gradient column chromatography on silica gel eluting with hexanes and EtOAc, followed by HPLC purification.

Regiochemical assignment was made on the basis of 1) the chemical shift (142.5 ppm) of the ^{19}F NMR signal, indicative of a tertiary fluoride, 2) ^{19}F signal coupling (dqd, J = 55.7, 33.7, 11.4 Hz) agrees with the chemical environment of the regioisomer, 3) absence of a downfield ^{1}H signal with approximately 50 Hz coupling implies no secondary or primary fluorides are present, 4) diagnostic methyl ^{1}H signal shift (1.53 and 1.48 ppm) and coupling constant (J = 21.9 Hz) for the fluoro-isopropyl group, and 6) identification of distinguishable peaks (quaternary, C-F, etc.) in the ^{13}C NMR spectrum are in agreement with the regioisomer assignment ($^{2}J_{CF}$- and $^{3}J_{CF}$-coupling and chemical shifts).

^{1}H NMR (400 MHz, $CDCl_3$) δ 7.73 (d, J = 8.3 Hz, 2H), 7.33 (d, J = 8.2 Hz, 2H), 4.87-4.81 (m, 1H), 2.90-2.83 (m, 1H), 2.48- 2.38 (m, 4H), 2.22 (dd, J = 14.1, 3.9 Hz, 1H), 2.10-1.99 (m, 5H), 1.92-1.75 (m, 2H), 1.71-1.54 (m, 5H), 1.48-1.42 (m, 5H), 1.03 (s, 3H). ^{13}C{1H} NMR (100 MHz, $CDCl_3$): δ 207.1, 144.9, 133.8, 129.8, 128.0, 99.7 (d, J = 164.7 Hz), 81.5, 79.0, 52.9, 50.2, 50.0, 48.1, 41.4, 36.7, 34.0, 29.6, 28.4, 28.1, 26.8, 26.5, 22.9, 22.8, 21.9, 19.4. ^{19}F NMR (282 MHz, $CDCl_3$): δ -142.5 (dqd, J = 55.7, 33.7, 11.4 Hz). FTMS (ESI) m/z $C_{21}H_{31}O_5SF$ (M+H$^+$): calc 427.1950, observed 427.1950.

2-((1*R*,2*R*,8a*R*)-4-fluoro-2-hydroxy-2,5,5,8a-tetramethyldecahydronaphthalen-1-yl)ethylacetate (compound **16**)

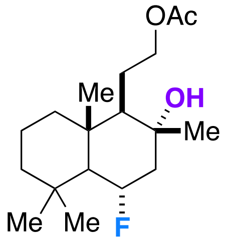

The reaction was run according to the general procedure, and the major diastereomer was isolated. The crude material was subjected to gradient column chromatography on silica gel eluting with hexanes and EtOAc, followed by HPLC purification.

Regiochemical and stereochemical assignments were made on the basis of 1) chemical shift in the ^{19}F NMR signal, indicative of a secondary fluoride on a cyclohexane ring, 2) the chemical shift and coupling constant (J = 55.7 Hz) of the ^{1}H signal at 4.49 ppm are indicative of a proton with a geminal fluoride, 3) the -OH ^{1}H signal shifts downfield (2.15 ppm) relative to the starting material and has a 5.7 Hz coupling constant (indicative of the alpha-fluoro configuration hydrogen bonding), 4) strong interaction within the ^{19}F-^{1}H HOESY spectrum between the -OH and fluoride infers the alpha-fluoro configuration, 5) interaction within the ^{1}H-^{1}H NOESY spectrum between the geminal proton to the fluorine and the methyl attached to the same carbon as the hydroxy group in the 3 position, 6) absence of a through-space coupling to the two methyl groups on the cyclohexane core infers the alpha-flouro configuration, and 7) identification of distinguishable peaks (quaternary, C-F, etc.) in the ^{13}C NMR spectrum are in agreement with the regioisomer assignment ($^{2}J_{CF}$- and $^{3}J_{CF}$-coupling and chemical shifts).

^{1}H NMR (400 MHz, $CDCl_3$): δ 4.49 (dm, J = 55.7 Hz, 1H), 4.19-4.05 (m, 2H), 2.15 (dm, J = 6.8 Hz, 1H), 2.09-2.01 (m, 4H), 1.84-1.75 (m, 1H), 1.69-1.60 (m, 3H), 1.54-1.34 (m, 5H), 1.24-1.15

(m, 1H), 1.13-1.12 (m, 3H), 0.98-0.90 (m, 1H), 0.87 (s, 3H), 0.80 (m, 6H).$^{13}C\{^1H\}$ NMR (100 MHz, $CDCl_3$): 171.3, 96.2 (d, J = 171.2 Hz), 73.6, 73.4, 66.5, 52.9, 47.12, 47.11, 41.9, 39.4, 38.4, 33.1, 32.9, 25.5, 25.3, 24.0, 21.49, 21.48, 21.3, 21.2, 21.1, 18.5, 14.94, 14.92. ^{19}F NMR (282 MHz, $CDCl_3$): δ -189.3 - (-189.7) (m). FTMS (ESI) m/z $C_{18}H_{31}O_3F$: calc 314.2257, observed 298.2303 (corresponds to loss of –OH + H^+).

2-((1*R*,2*S*)-4-fluoro-2-hydroxy-2,6,6-trimethylcyclohexyl)ethyl acetate (compound **17**)

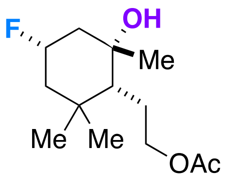

The reaction was run according to the general procedure, and the major diastereomer was isolated. The crude material was subjected to gradient column chromatography on silica gel eluting with hexanes and EtOAc, followed by HPLC purification.

Regiochemical and stereochemical assignments were made on the basis of 1) chemical shift in the ^{19}F NMR signal, indicative of a secondary fluoride on a cyclohexane ring, 2) the chemical shift and coupling constant (J = 49.4 Hz) of the ^1H signal at 4.91 ppm are indicative of a proton with a geminal fluoride, 3) absence of a strong interaction between -OH and geminal proton to the fluorine in the ^1H-^1H NOESY, as well as interaction between the geminal proton and the methyl geminal to the alcohol, suggest the indicated diastereomer, 4) the ^1H signal at 4.91 ppm (doublet of triplets of triplets) agrees with the proposed regioisomer, and 5) identification of distinguishable peaks (quaternary, C-F, etc.) in the ^{13}C NMR spectrum are in agreement with the regioisomer assignment ($^2J_{CF}$- and $^3J_{CF}$-coupling and chemical shifts).

^1H NMR (400 MHz, $CDCl_3$): δ 4.91 (dtt, J = 49.4, 11.5, 4.5 Hz, 1H), 4.18-3.98 (m, 2H), 2.22-2.12 (m, 1H), 2.05 (s, 3H), 2.01-1.92 (m, 1H), 1.86-1.59 (m, 2H), 1.40-1.28 (m, 4H), 1.03-1.01 (m, 4H), 0.98 (s, 4H), 0.95-0.92 (m. 1H).$^{13}C\{^1H\}$ NMR (100 MHz, $CDCl_3$): δ 171.2, 88.1 (d, J = 167.1), 75.0, 74.9, 65.9, 49.6, 49.6, 47.7, 47.5, 47.4, 47.3, 36.6, 36.4, 32.1, 31.3, 27.1, 24.3, 22.2, 21.2. ^{19}F NMR (282 MHz, $CDCl_3$): δ -180.7 (dm, J = 49.4 Hz). FTMS (ESI) m/z $C_{13}H_{23}O_3F$: calc 246.1631, observed 229.1602 (corresponds to loss of -OH).

(1R,3S)- and (1S,3R)-1-(4-chlorophenyl)-3-fluorocycloheptan-1-ol (compound **18**)

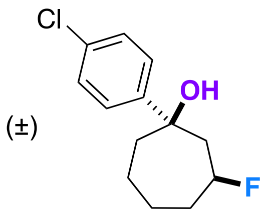

The reaction was run according to the general procedure (with the exception: 0.0 equiv. of $NaHCO_3$ used), and the major diastereomer was isolated. The crude material was subjected to

gradient column chromatography on silica gel eluting with hexanes and EtOAc, followed by HPLC purification.

Regiochemical and stereochemical assignments were made on the basis of 1) chemical shift in the ^{19}F NMR signal, indicative of a secondary fluoride on a cycloheptane ring, 2) ^{1}H signal coupling constants (doublet of doublet of doublets, J = 44.66, 13.07, 2.17 Hz) at 4.86 ppm agrees with the chemical environment of the regioisomer, 3) through-space coupling between the -OH and F (J = 4.05 Hz) for the ^{1}H peak at 2.56 ppm and a very strong interaction between -OH and -F in the ^{19}F-^{1}H HOESY spectrum (suggestive of a through-space coupling and intramolecular hydrogen bonding -OH---F-), 4) computational modeling at B3LYP 6-311++G** show that only the 3-beta-fluoro has the geometrical possibility for intramolecular hydrogen bonding, and a strong interaction within the ^{19}F-^{1}H HOESY spectrum is observed, 5) ^{1}H signal alpha to the alcohol shifted downfield (2.26 ppm), and 6) identification of distinguishable peaks (quaternary, C-F, etc.) in the ^{13}C NMR spectrum are in agreement with the regioisomer assignment ($^{2}J_{CF}$- and $^{3}J_{CF}$-coupling and chemical shifts).

^{1}H NMR (400 MHz, $CDCl_3$): δ 7.47-7.45 (m, 2H), 7.37-7.35 (m, 2H), 4.86 (ddd, J = 44.66, 13.07, 2.17 Hz, 1H), 2.57 (d, J = 4.05 Hz, 1H), 2.31-2.22 (m, 1H), 1.99-1.76 (m, 5H), 1.74-1.48 (m, 4H). ^{13}C{^{1}H} NMR (100 MHz, $CDCl_3$): δ 145.91 (d, J = 1.52 Hz), 132.76, 128.43, 126.18, 96.81 (d, J = 172.29 Hz), 76.66 (d, J = 18.89 Hz), 39.09 (d, J = 4.87 Hz), 27.94 (d, J = 20.86 Hz), 26.58, 21.51 (d, J = 13.63 Hz), 19.97; ^{19}F NMR (282 MHz, $CDCl_3$): δ -176.85 – (-177.03) (m). FTMS (ESI) m/z $C_{13}H_{16}OF$: calc 242.0874, observed 225.0832 (corresponds to loss of -OH).

(1R,3S)- and (1S,3R)-1-(4-chlorophenyl)-3-fluorocyclopentan-1-ol (compound **19**)

(±)

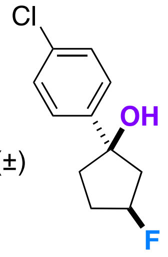

The reaction was run according to the general procedure (with the exception: 0.0 equiv. of $NaHCO_3$ used), and the major diastereomer was isolated. The crude material was subjected to gradient column chromatography on silica gel eluting with hexanes and EtOAc, followed by HPLC purification.

Regiochemical and stereochemical assignments were made on the basis of 1) chemical shift in the ^{19}F NMR signal, indicative of a secondary fluoride on a cycloheptane ring, 2) ^{1}H signal at 4.80 ppm has a diagnostic geminal fluoride coupling ($^{2}J_{HF}$ = 51.82 Hz), 3) absence of a strong interaction between the geminal proton to fluorine and the -OH within the ^{1}H-^{1}H NOESY spectrum suggests the cis fluoro/hydroxy conformation, 4) ^{1}H signal alpha to the alcohol shifted downfield (2.50 ppm), suggestive of intramolecular hydrogen bonding, and 5) identification of distinguishable peaks (quaternary, C-F, etc.) in the ^{13}C NMR spectrum are in agreement with the regioisomer assignment ($^{2}J_{CF}$- and $^{3}J_{CF}$-coupling and chemical shifts).

^{1}H NMR (400 MHz, $CDCl_3$): δ 7.50-7.46 (m, 2H), 7.36-7.33 (m, 2H), 4.80 (dm, J = 51.82Hz, 1H), 2.47-2.26 (m, 2H), 2.10-1.94 (m, 4H). $^{13}C\{^{1}H\}$ NMR (100 MHz, $CDCl_3$): δ 140.14, 133.71, 128.39, 128.14 (d, J = 2.20 Hz), 99.28 (d, J = 180.35 Hz), 83.37 (d, J = 23.47). 36.02 (d, J = 1.10 Hz), 30.74 (d, J = 21.63 Hz), 20.65. ^{19}F NMR (282 MHz, $CDCl_3$): δ -173.90 – (-174.23) (m). FTMS (ESI) m/z $C_{11}H_{12}OF$: calc 214.0561, observed 179.0428 (corresponds to loss of (-OH and -F) plus H^{+}).

(1S,3S)- and (1R,3R)-3-fluoro-1-methyl-1,2,3,4-tetrahydronaphthalen-1-ol (compound **20**)

(±)

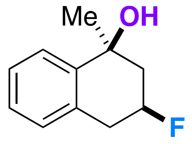

The reaction was run according to the general procedure (with the exception: 0.0 equiv. of $NaHCO_3$ used), and the major diastereomer was isolated. The crude material was subjected to gradient column chromatography on silica gel eluting with hexanes and EtOAc, followed by HPLC purification.

Regiochemical and stereochemical assignments were made on the basis of 1) chemical shift in the ^{19}F NMR signal, indicative of a secondary fluoride on a cyclohexane ring, 2) ^{1}H signal coupling constants (doublet of doublet of doublets, J = 49.85, 10.16, 3.40 Hz,) agrees with the chemical environment of the regioisomer, 3) absence of a strong interaction between -OH and geminal proton to the fluorine in the ^{1}H-^{1}H NOESY spectrum suggests the cis fluoro/hydroxy conformation, 4) benzylic ^{1}H signals shifted downfield to 2.95 ppm, and 5) identification of distinguishable peaks (quaternary, C-F, etc.) in the ^{13}C NMR spectrum are in agreement with the regioisomer assignment ($^{2}J_{CF}$- and $^{3}J_{CF}$-coupling and chemical shifts).

^{1}H NMR (400 MHz, $CDCl_3$): δ 7.62-7.60 (m, 1H), 7.24-7.19 (m, 2H), 7.10-7.08 (m, 1H), 4.80 (ddd, J = 49.85, 10.16, 3.40 Hz, 1H), 3.05-2.80 (m, 2H), 2.31-2.09 (m, 2H), 2.07 (s, 1H), 1.58 (d, J = 2.57 Hz, 3H). $^{13}C\{^{1}H\}$ NMR (100 MHz, $CDCl_3$): δ 140.48, 134.22, 128.31, 127.66, 126.85, 126.37, 96.84, 95.06, 72.95, 72.75, 26.50, 26.40, 25.70, 25.51, 25.34, 25.28. ^{19}F NMR (282 MHz, $CDCl_3$): □ -191.36 – (-191.57) (m). FTMS (ESI) m/z $C_{11}H_{13}OF$: calc 180.0950, observed 163.0910 (corresponds to loss of -OH).

(1S, 3S)- and (1R,3R)-3-fluoro-1-phenyl-2,3-dihydro-1H-inden-1-ol (compound **21**)

(±)

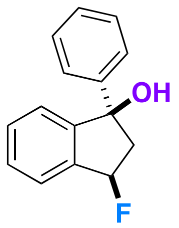

The reaction was run according to the general procedure, and the major diastereomer was isolated. The crude material was subjected to gradient column chromatography on silica gel eluting with hexanes and EtOAc, followed by HPLC purification.

Regiochemical and stereochemical assignments were made on the basis of 1) chemical shift in the ^{19}F NMR signal, indicative of a secondary fluoride, 2) ^{1}H signal at 5.15 ppm has a diagnostic geminal fluoride coupling ($^{2}J_{HF}$ = 54.2 Hz), 3) diagnostic -OH in the starting material (2.13 ppm, broad singlet) shifts downfield (3.21 ppm, doublet, J = 3.2 Hz), 4) very strong interaction between -OH and -F in the ^{19}F-^{1}H HOESY spectrum (suggestive of a through-space coupling and intramolecular hydrogen bonding -OH---F-), and 5) identification of distinguishable peaks (quaternary, C-F, etc.) in the ^{13}C NMR spectrum are in agreement with the regioisomer assignment ($^{2}J_{CF}$- and $^{3}J_{CF}$-coupling and chemical shifts).

^{1}H NMR (400 MHz, $CDCl_3$): δ 7.40-7.28 (m, 7H), 7.23-7.20 (m, 2H), 5.15 (dm, J = 54.2 Hz, 1H), 3.21, (d, J = 3.80 Hz, 1H), 3.17-3.12 (m, 2H). ^{13}C{^{1}H} NMR (100 MHz, $CDCl_3$): δ 144.19. 141.59, 141,57, 139.37, 139.36, 129.23, 128.36, 128.01, 127.93 126.55, 126.54, 125.28, 125.07, 101.37, 99.50, 85.19, 85.02, 35.99, 35.76. ^{19}F NMR (282 MHz, $CDCl_3$): □ -186.21 - -186.51 (m). TOF MS(Cl) m/z $C_{11}H_{13}OF$: calc 228.0950, observed 211.0596 (corresponds to loss of -OH).

<u>(1S,2S,4R,6R)-6-fluoro-1,3,3-trimethylbicyclo[2.2.1]heptan-2-ol</u> (compound **22**)

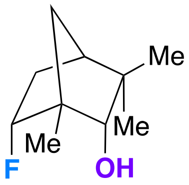

The reaction was run according to the general procedure (with the exception: 1.2 equiv. of Selectfluor used), and the major diastereomer was isolated. The crude material was subjected to gradient column chromatography on silica gel eluting with hexanes and EtOAc, followed by HPLC purification.

Regiochemical and stereochemical assignments were made on the basis of 1) chemical shift in the ^{19}F NMR signal, indicative of a secondary fluoride, 2) ^{1}H signal at 4.83 ppm has a diagnostic geminal fluoride coupling ($^{2}J_{HF}$ = 56.4 Hz), 3) diagnostic -OH in the starting material (1.36 ppm, broad singlet) shifts downfield (1.78 ppm, doublet, J = 3.2 Hz), 4) very strong interaction between -OH and -F in the ^{19}F-^{1}H HOESY spectrum (suggestive of a through-space coupling and intramolecular hydrogen bonding -OH---F-), 5) C10 methyl at the ring junction has a drastic shift to 1.23 ppm and coupling of 1.7 Hz, and 6) identification of distinguishable peaks (quaternary, C-F, etc.) in the ^{13}C NMR spectrum are in agreement with the regioisomer assignment ($^{2}J_{CF}$- and $^{3}J_{CF}$-coupling and chemical shifts).

^{1}H NMR (400 MHz, $CDCl_3$): δ 4.83 (dm, J = 56.44 Hz, 1H), 3.41 (dd, J = 6.70, 3.90 Hz, 1H), 2.39-2.30 (m, 1H), 1.78 (d, J = 4.13 Hz, 1H), 1.45-1.44 (m, 2H), 1.32-1.30 (m, 1H), 1.23 (d, J = 1.70 Hz, 3H), 1.05 (s, 3H), 0.92-0.89 (m, 2H), 0.86 (s, 3H). ^{13}C{^{1}H} NMR (100 MHz, $CDCl_3$): δ93.73, 91.94, 84.16, 84.08, 46.69. 39.26, 36.85, 36.28, 36.08, 30.36, 19.99, 14.37, 14.32. ^{19}F

NMR (282 MHz, $CDCl_3$): □ m (-183.85 - -184.27). TOF MS (Cl) m/z $C_{11}H_{13}OF$: calc 172.1270, observed 172.1263

(1R,2R,4S,5S,9s)-9-(4-chlorophenyl)-2,4-difluorobicyclo[3.3.1]nonan-9-ol (compound **23**)

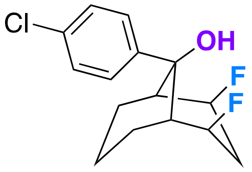

The reaction was run according to the general procedure (with the exception: 3.0 equiv. of Selectfluor used), and the major diastereomer was isolated. The crude material was subjected to gradient column chromatography on silica gel eluting with hexanes and EtOAc, followed by HPLC purification.

Regiochemical and stereochemical assignments were made on the basis of 1) chemical shift in the ^{19}F NMR signal, indicative of a secondary fluoride, 2) the ^{1}H signal at 5.05 ppm has a diagnostic geminal fluoride coupling ($^{2}J_{HF}$ = 48.06 Hz), 3) considering the symmetry of the molecule—the ^{13}C and ^{1}H spectra agree with the proposed structure (e.g. integrations, # of peaks, and coupling) 4) diagnostic -OH is a triplet due to through-space coupling with the two fluorines, 5) ^{1}H signal alpha to the alcohol is shifted downfield (3.28 ppm), suggestive of intramolecular hydrogen bonding, and 6) identification of distinguishable peaks (quaternary, C-F, etc.) in the ^{13}C NMR spectrum are in agreement with the regioisomer assignment ($^{2}J_{CF}$- and $^{3}J_{CF}$-coupling and chemical shifts).

^{1}H NMR (400 MHz, $CDCl_3$): δ 7.49-7.47 (m, 2H), 7.40-7.38 (m, 2H), 5.06 (dd, J = 48.06, 5.81 Hz, 2H), 3.28 (t, J = 19.20, 9.60 Hz, 1H), 3.08 (d, J = 17.07 Hz, 2H), 2.78-2.37 (m, 2H), 1.83-1.71 (m, 2H), 1.62-1.56 (m, 2H), 1.37-1.30 (m, 1H), 1.27-1.13 (m, 1H). $^{13}C\{^{1}H\}$ NMR (100 MHz, $CDCl_3$): δ 141.47, 133.39, 129.02, 127.23, 93.56 (d, J = 170.74 Hz) 73.38, 40.58 (d, J = 8.02 Hz) 34.81 (t, J = 46.50, 23.25 Hz), 29.72, 25.58 (d, J = 10.35 Hz), 17.56. ^{19}F NMR (282 MHz, $CDCl_3$): □ -152.62 – (-153.00) (m). FTMS (ESI) m/z $C_{15}H_{17}OF_2$: calc 286.0986, observed 269.0900 (corresponds to loss of -OH).

Spectral Data for Starting Materials

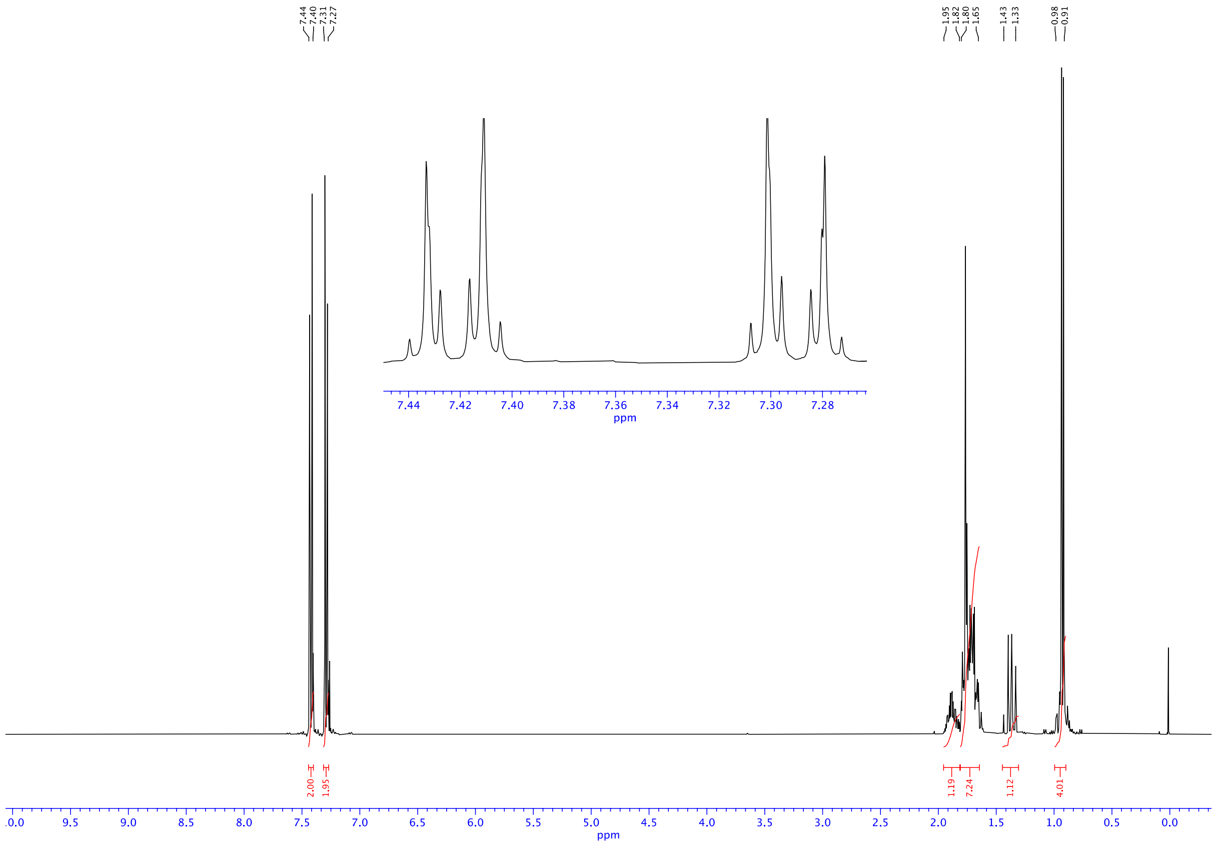

Fig. S1. ^{1}H NMR spectrum ($CDCl_3$, 400 MHz) of compound **5.**

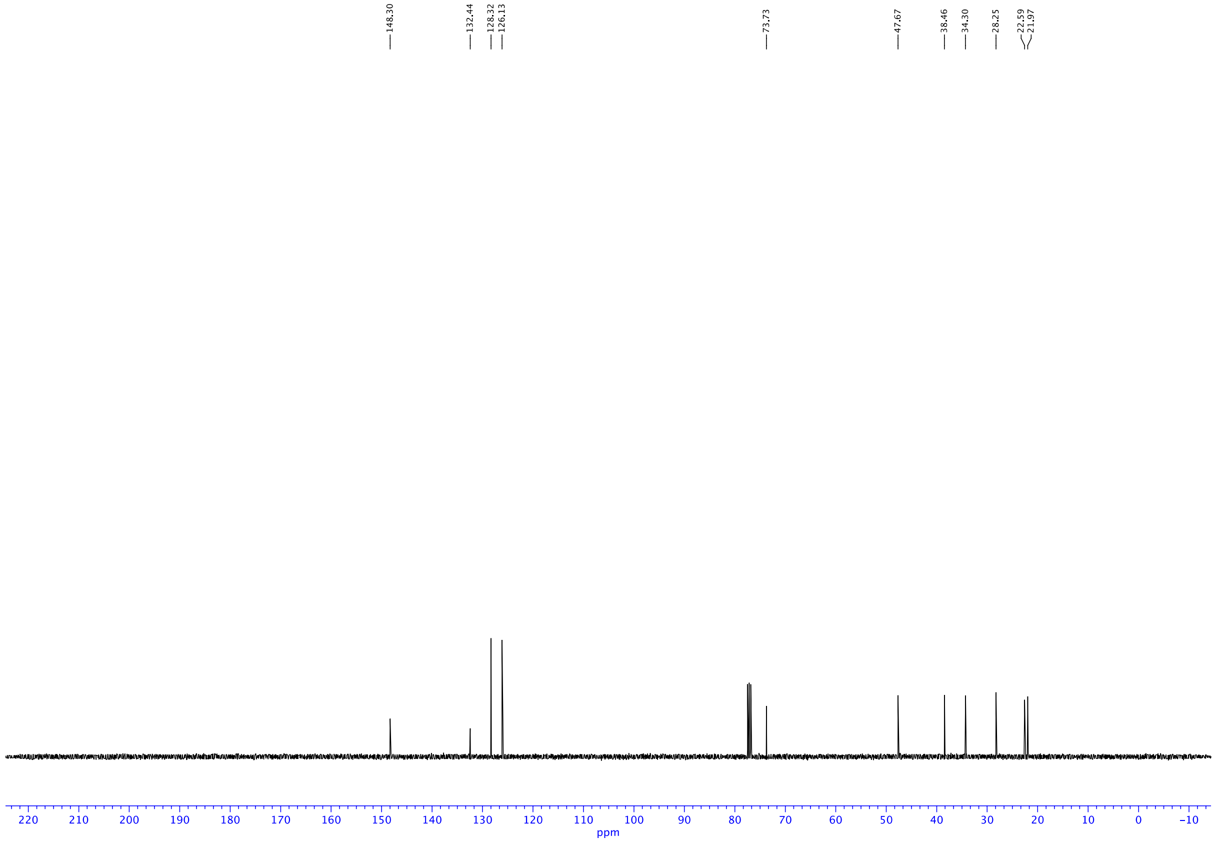

Fig. S2. $^{13}C\{^{1}H\}$ NMR spectrum ($CDCl_3$, 100 MHz) of compound **5.**

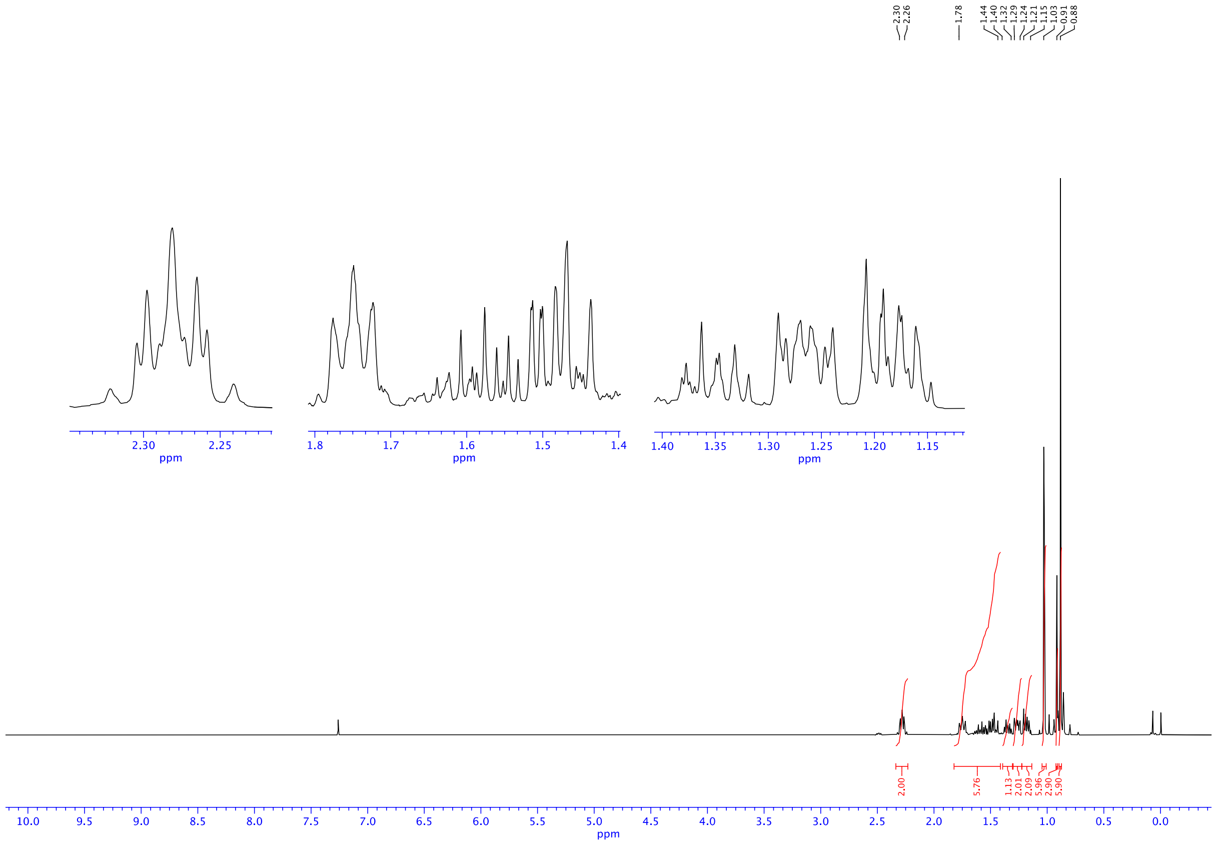

Fig. S3. ^{1}H NMR spectrum ($CDCl_3$, 400 MHz) of starting material for compound **8.**

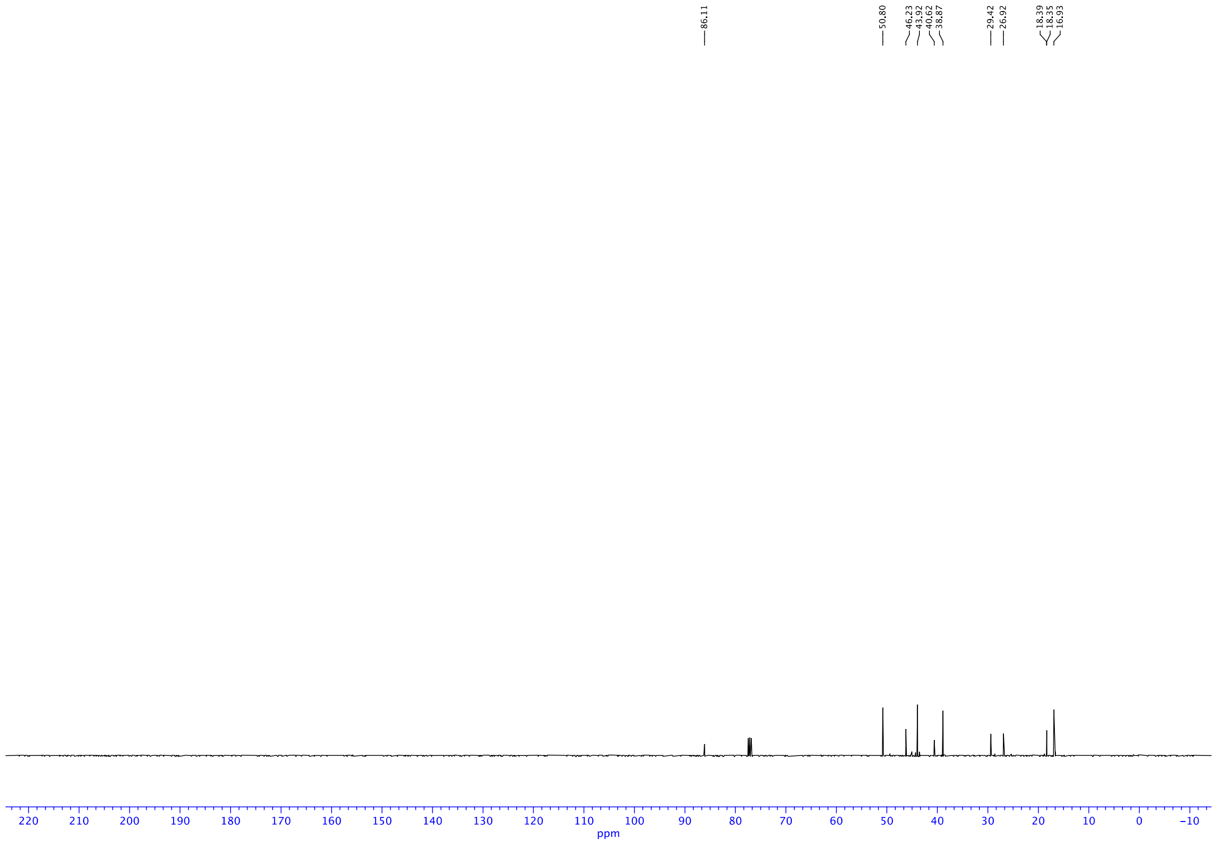

Fig. S4. $^{13}C\{^1H\}$ NMR spectrum ($CDCl_3$, 100 MHz) of starting material for compound **8.**

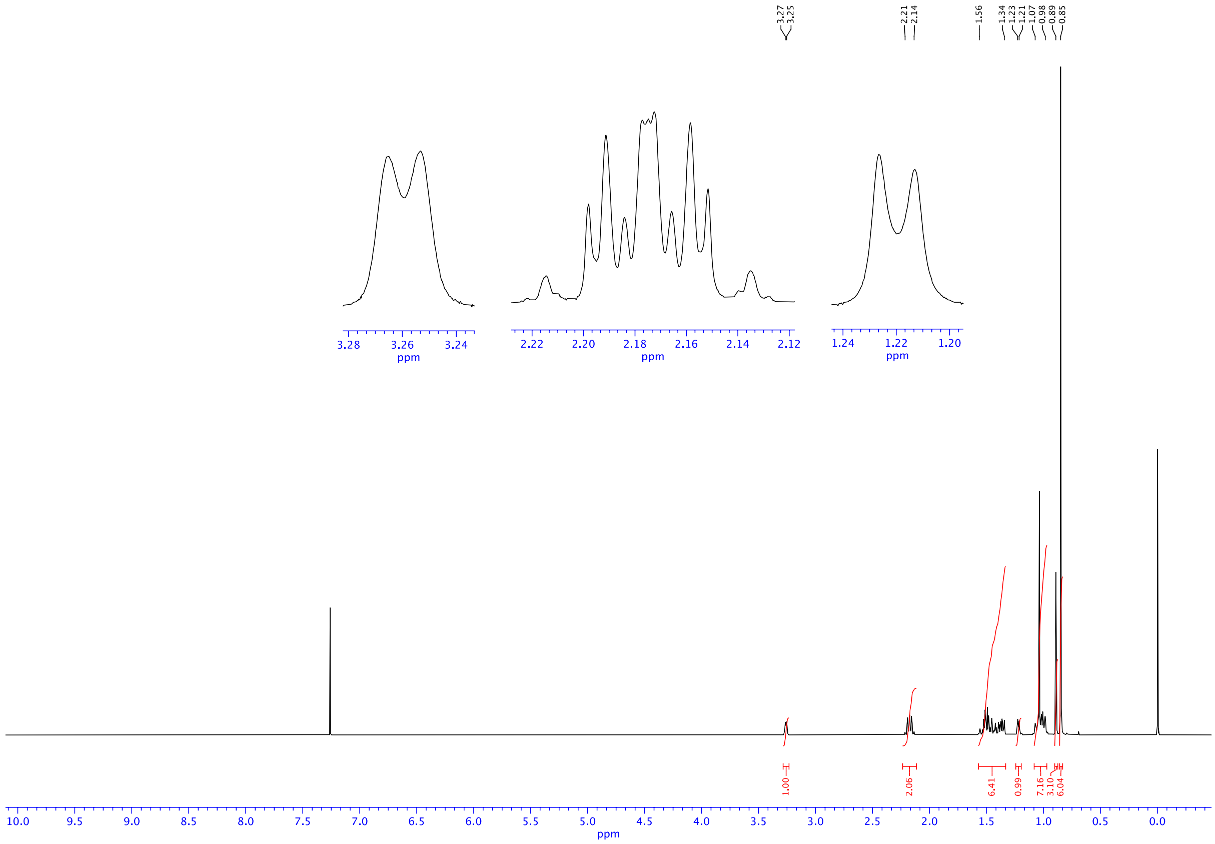

Fig. S5. ^{1}H NMR spectrum ($CDCl_3$, 400 MHz) of starting material for compound **9.**

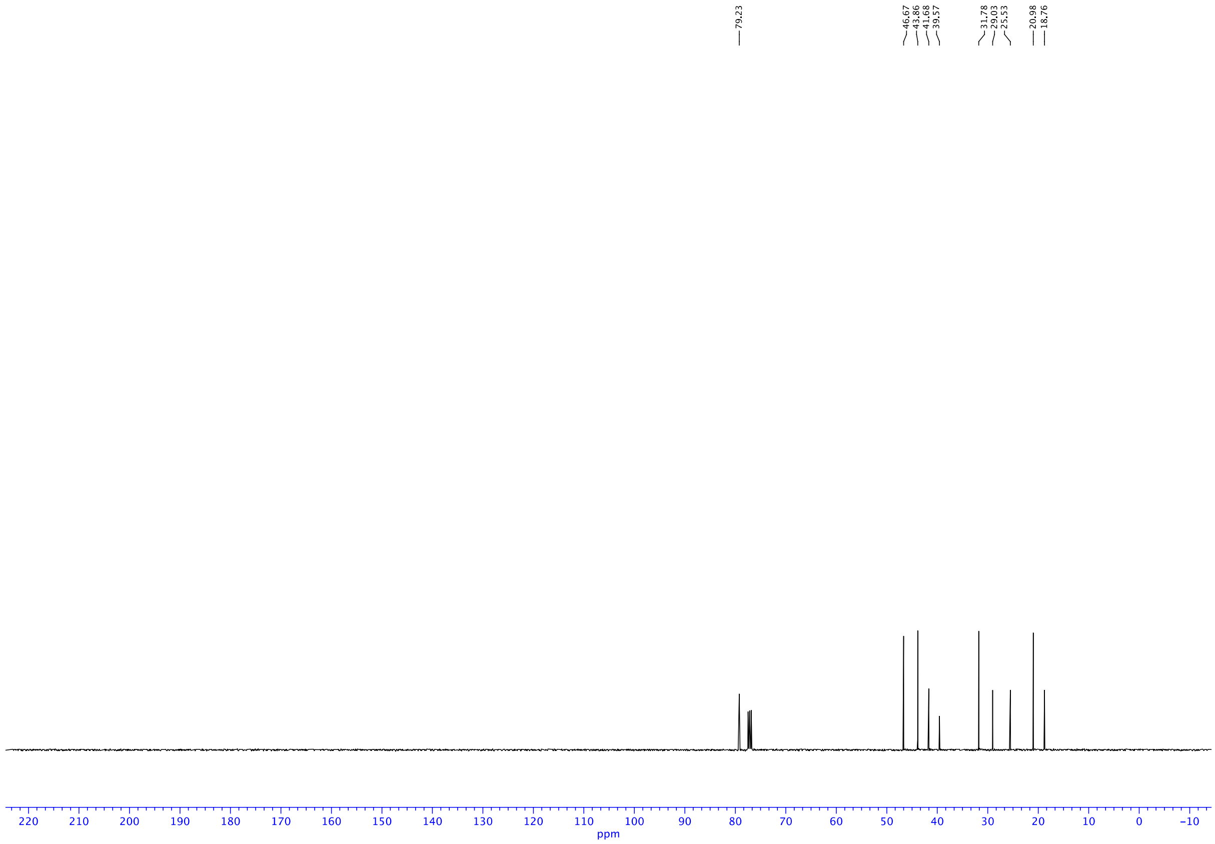

Fig. S6. $^{13}C\{^1H\}$ NMR spectrum ($CDCl_3$, 100 MHz) of starting material for compound **9.**

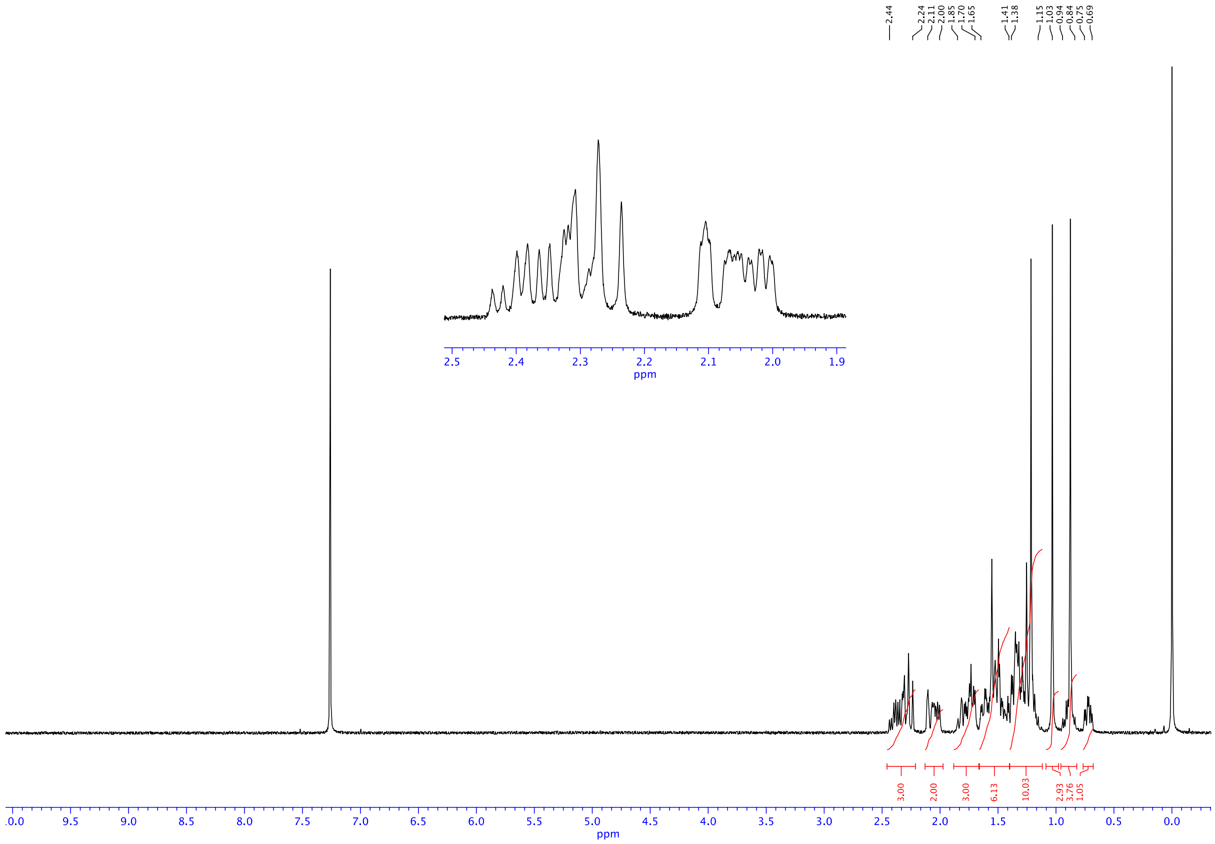

Fig. S7. ^{1}H NMR spectrum ($CDCl_3$, 400 MHz) of starting material for compound **10.**

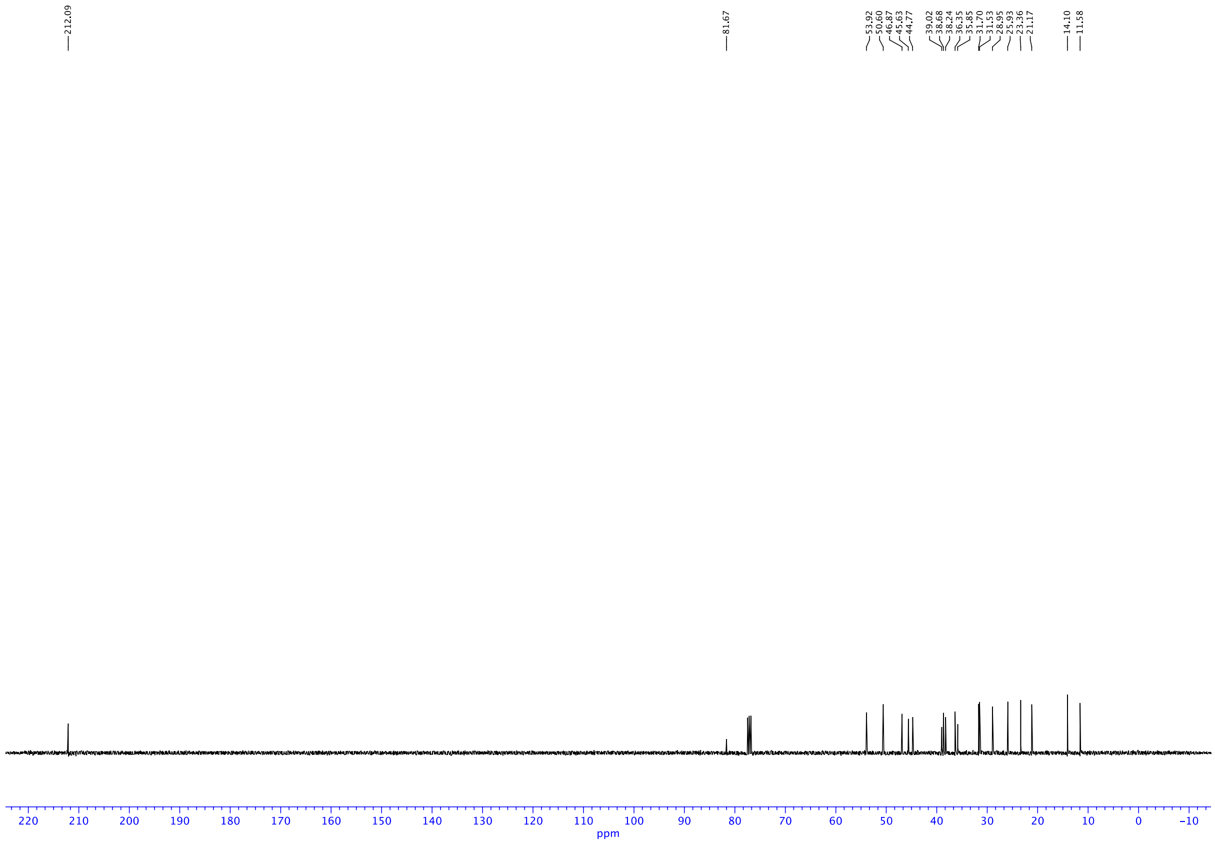

Fig. S8. $^{13}C\{^{1}H\}$ NMR spectrum ($CDCl_3$, 100 MHz) of starting material for compound **10.**

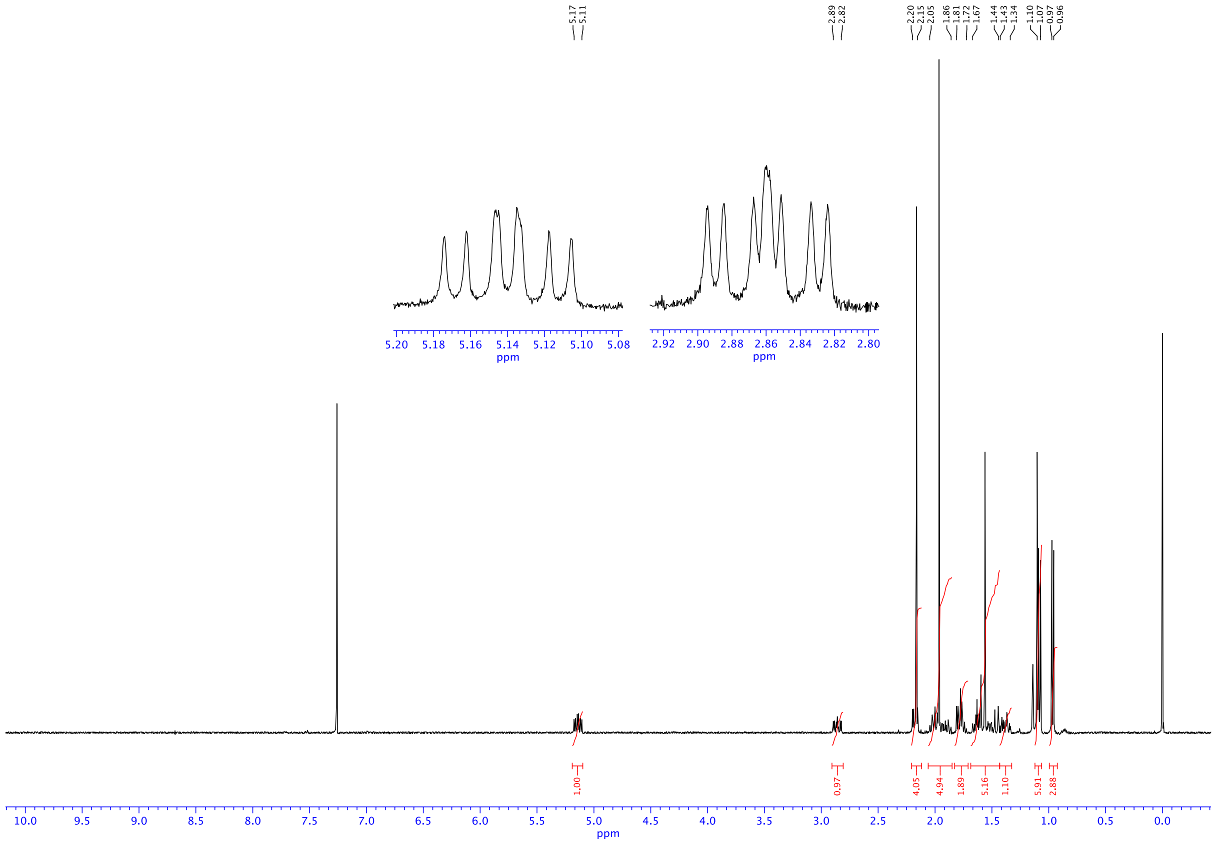

Fig. S9. ^{1}H NMR spectrum ($CDCl_3$, 400 MHz) of starting material for compound **14.**

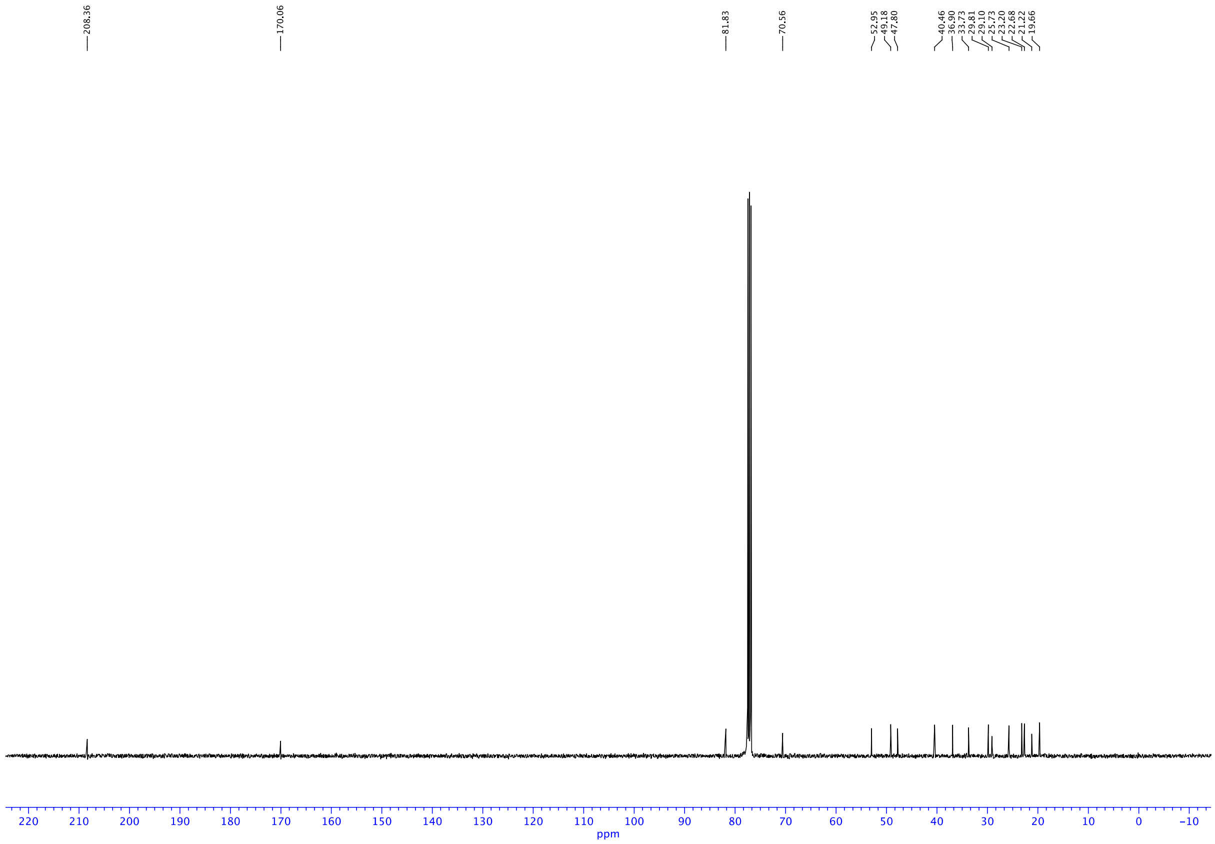

Fig. S10. $^{13}C\{^{1}H\}$ NMR spectrum ($CDCl_3$, 100 MHz) of starting material for compound **14.**

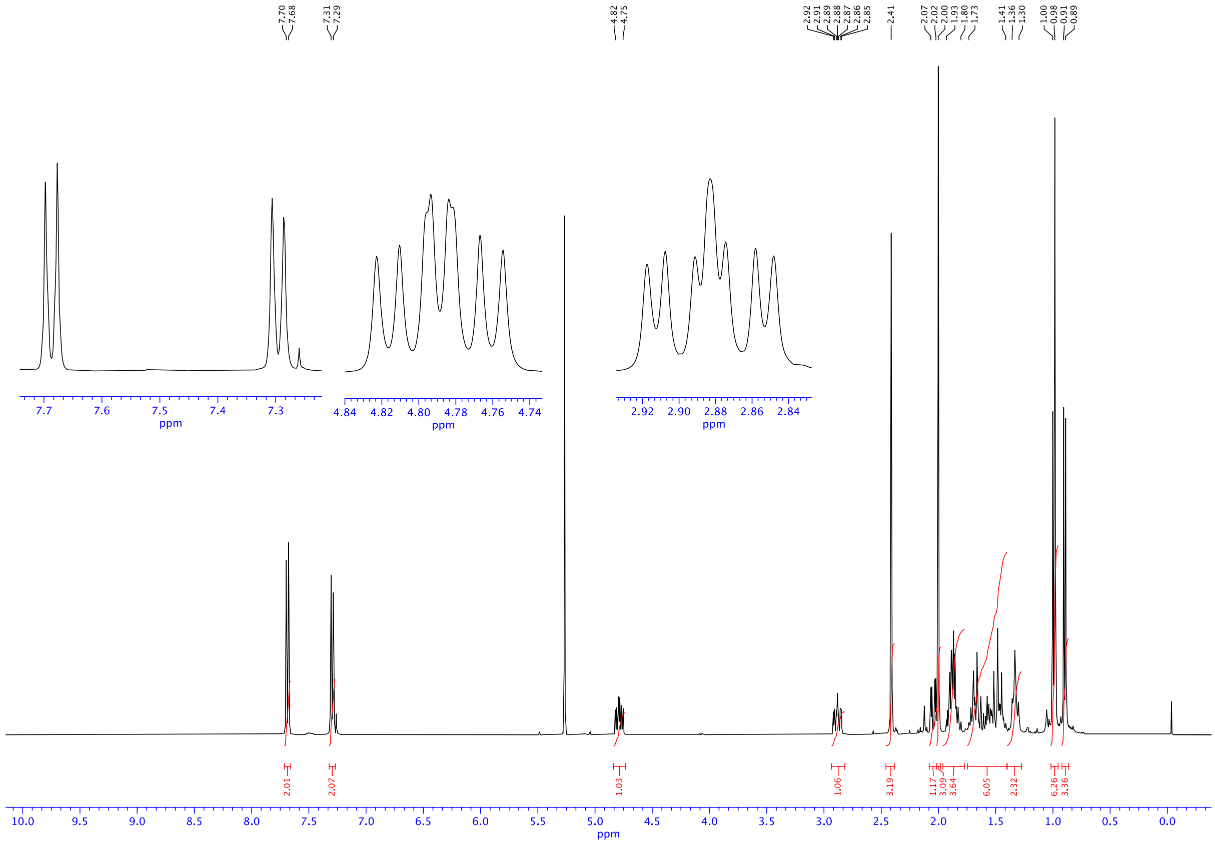

Fig. S11. ^{1}H NMR spectrum ($CDCl_3$, 400 MHz) of starting material for compound **15.**

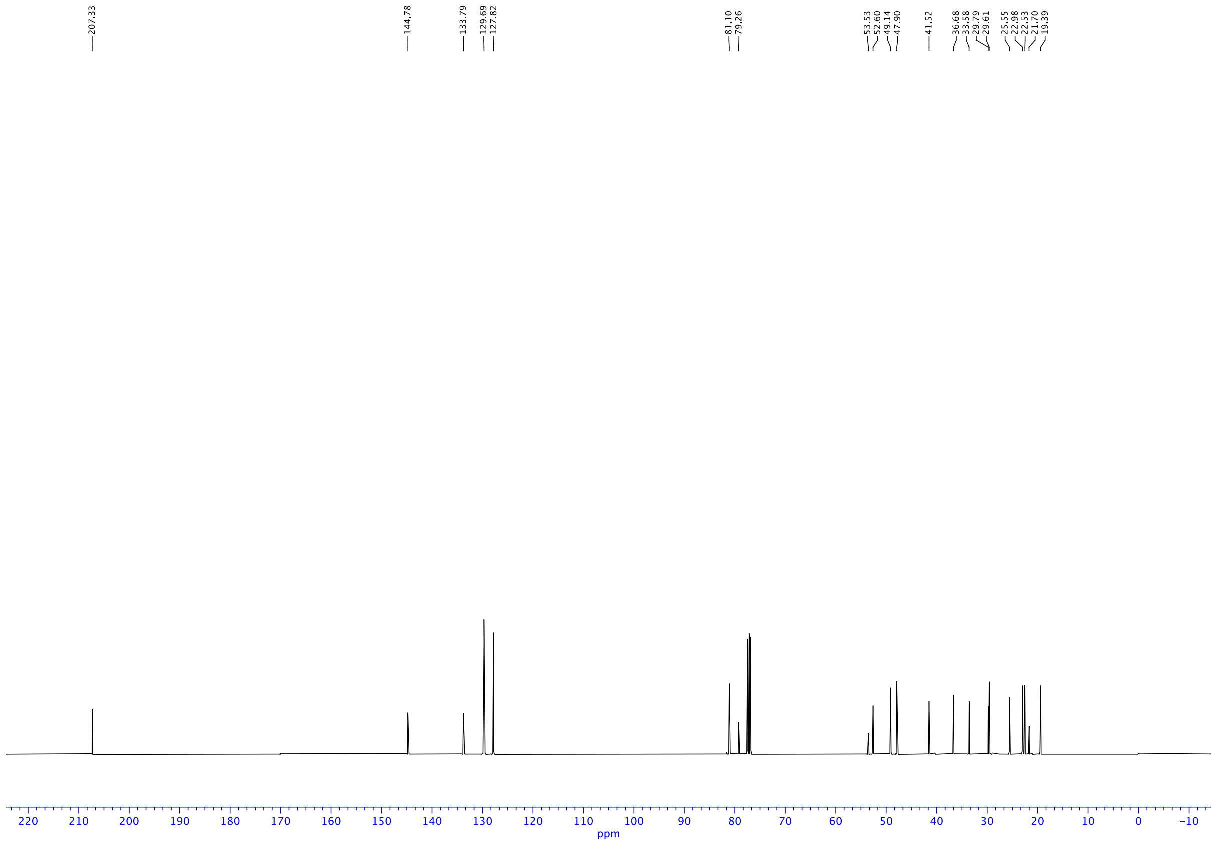

Fig. S12. $^{13}C\{^1H\}$ NMR spectrum ($CDCl_3$, 100 MHz) of starting material for compound **15.**

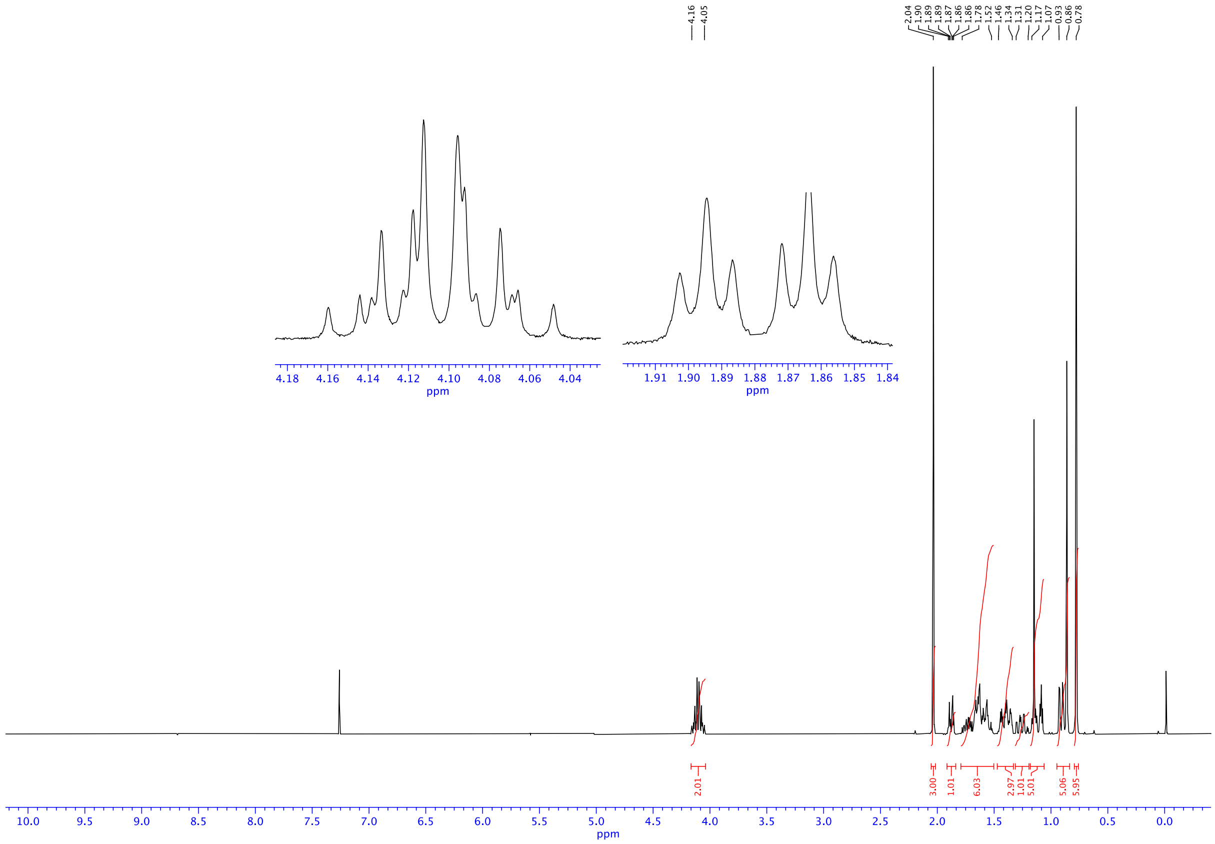

Fig. S13. ^{1}H NMR spectrum ($CDCl_3$, 400 MHz) of starting material for compound **16.**

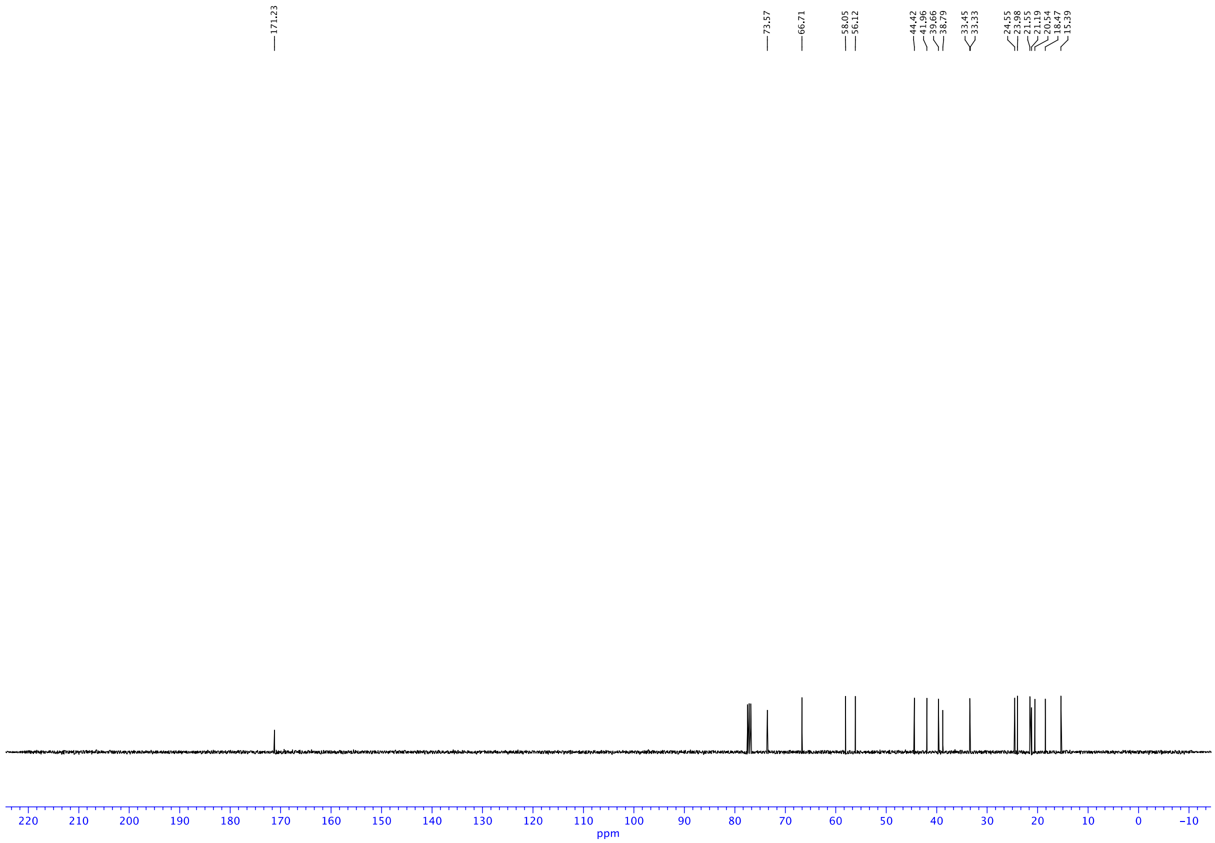

Fig. S14. $^{13}C\{^{1}H\}$ NMR spectrum ($CDCl_3$, 100 MHz) of starting material for compound **16.**

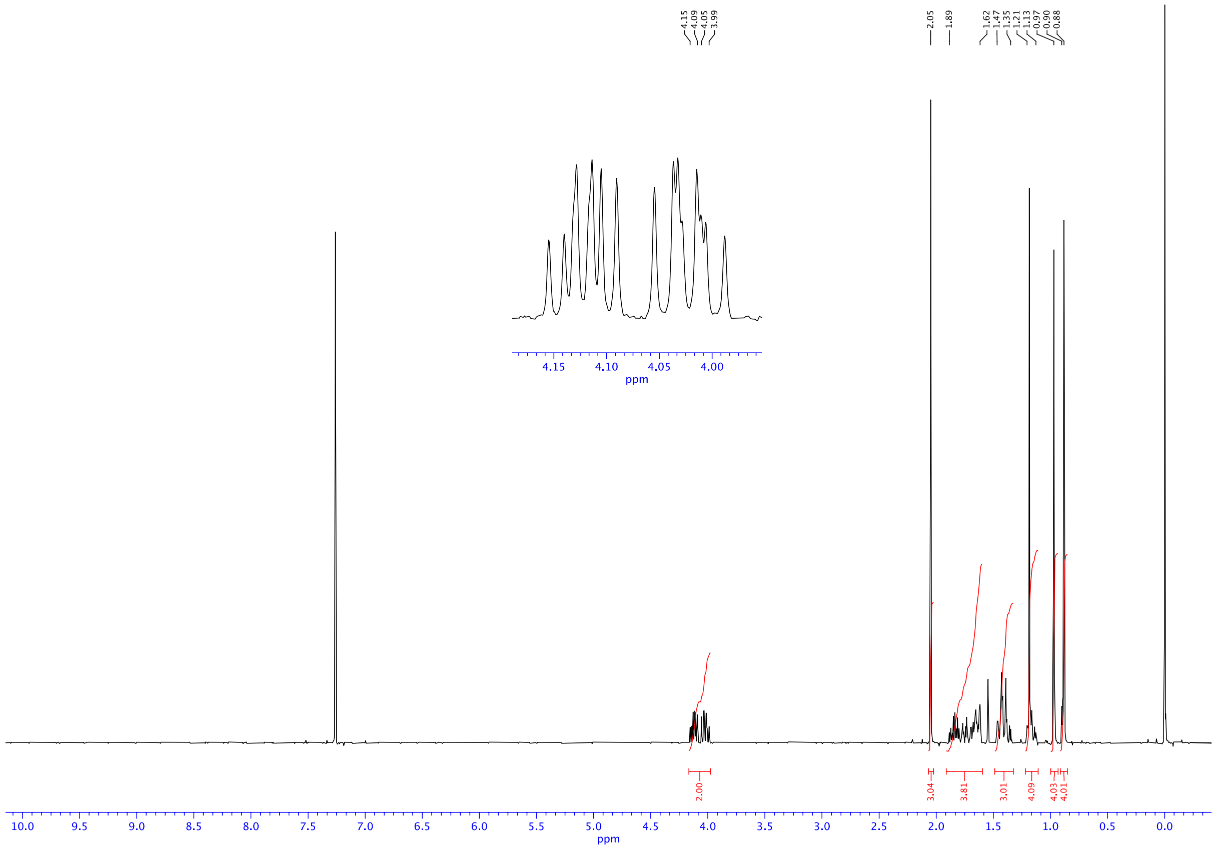

Fig. S15. ^{1}H NMR spectrum ($CDCl_3$, 400 MHz) of starting material for compound **17.**

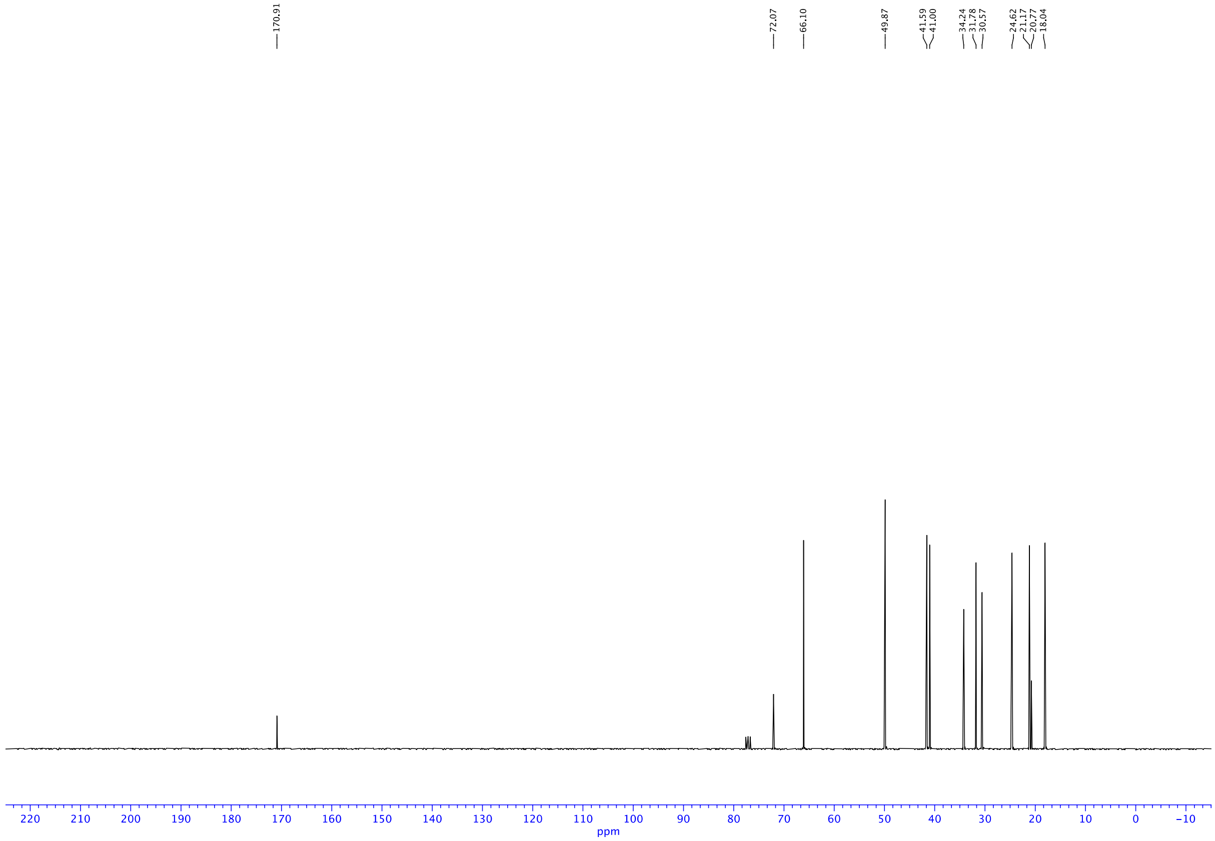

Fig. S16. $^{13}C\{^{1}H\}$ NMR spectrum ($CDCl_3$, 100 MHz) of starting material for compound **17.**

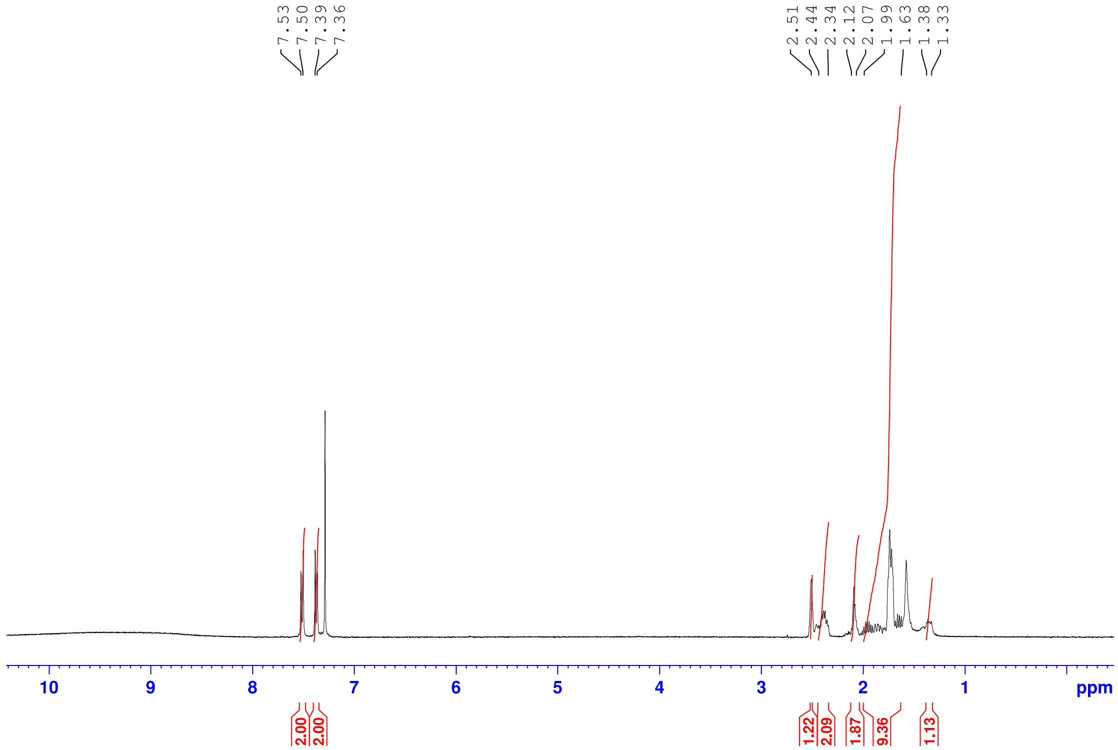

Fig. S17. ^{1}H NMR spectrum ($CDCl_3$, 400 MHz) of starting material for compound **23.**

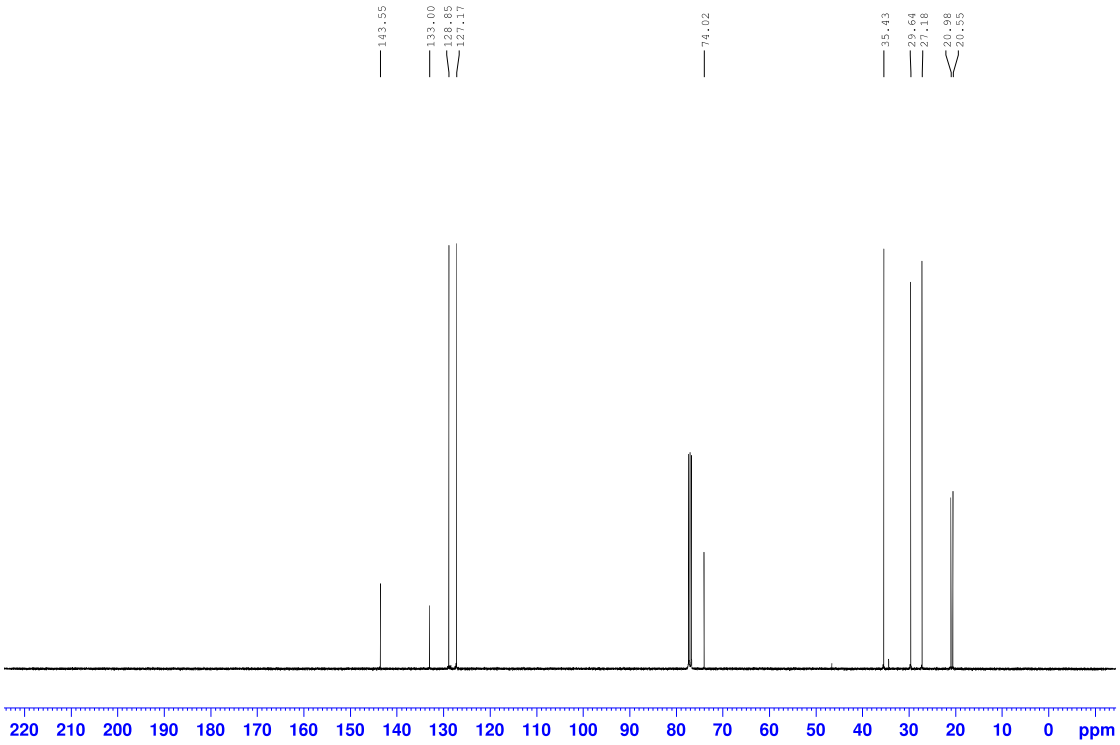

Fig. S18. $^{13}C\{^1H\}$ NMR spectrum ($CDCl_3$, 100 MHz) of starting material for compound **23.**

Spectral Data for Fluorinated Materials

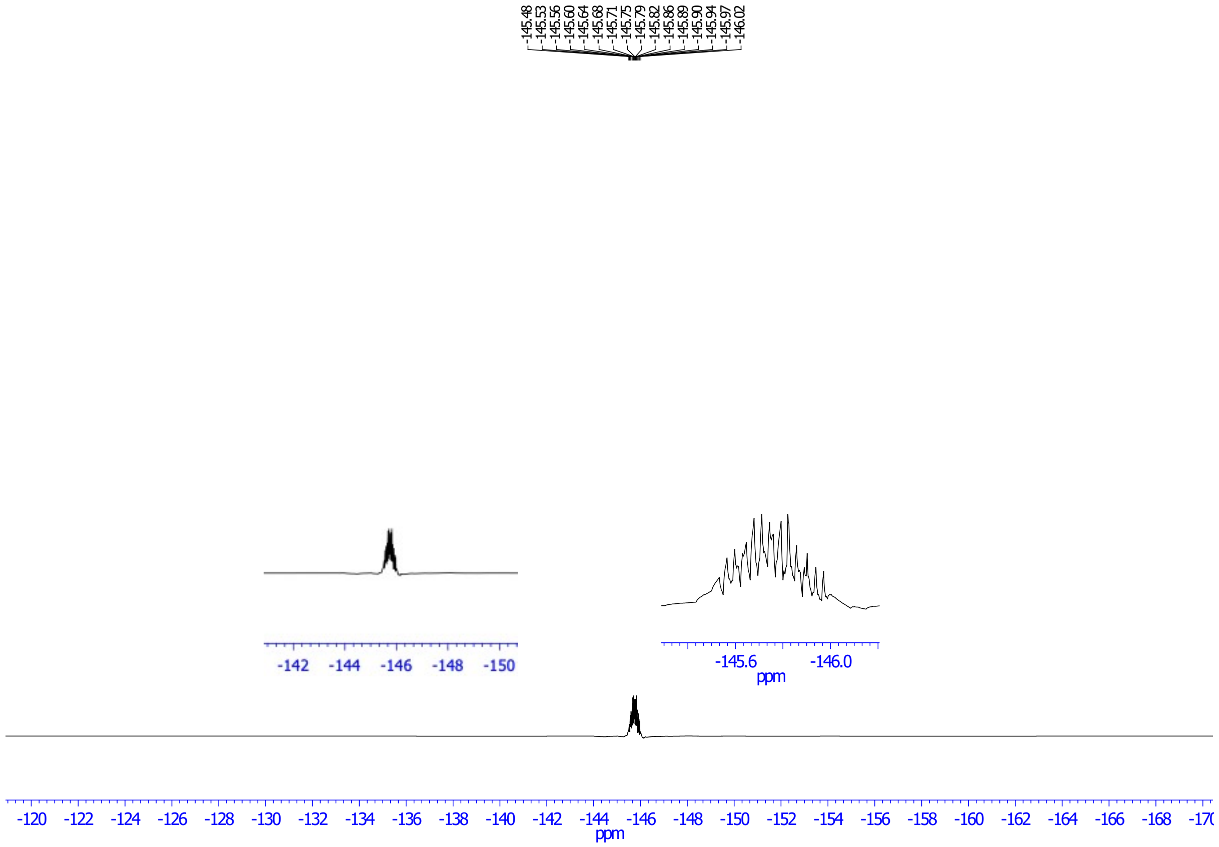

Fig. S19. ^{19}F NMR spectrum ($CDCl_3$, 282 MHz) of compound **6**.

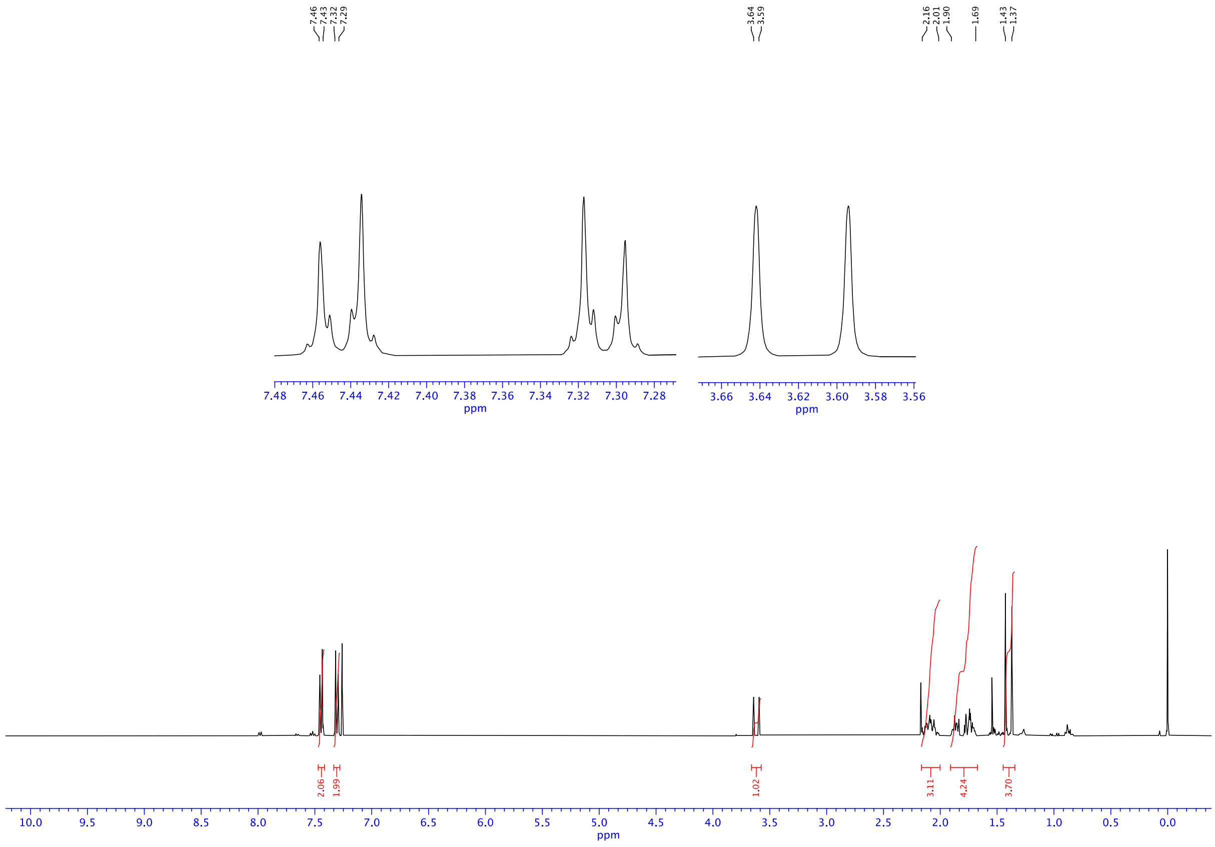

Fig. S20. ^{1}H NMR spectrum ($CDCl_3$, 400 MHz) of compound **6**.

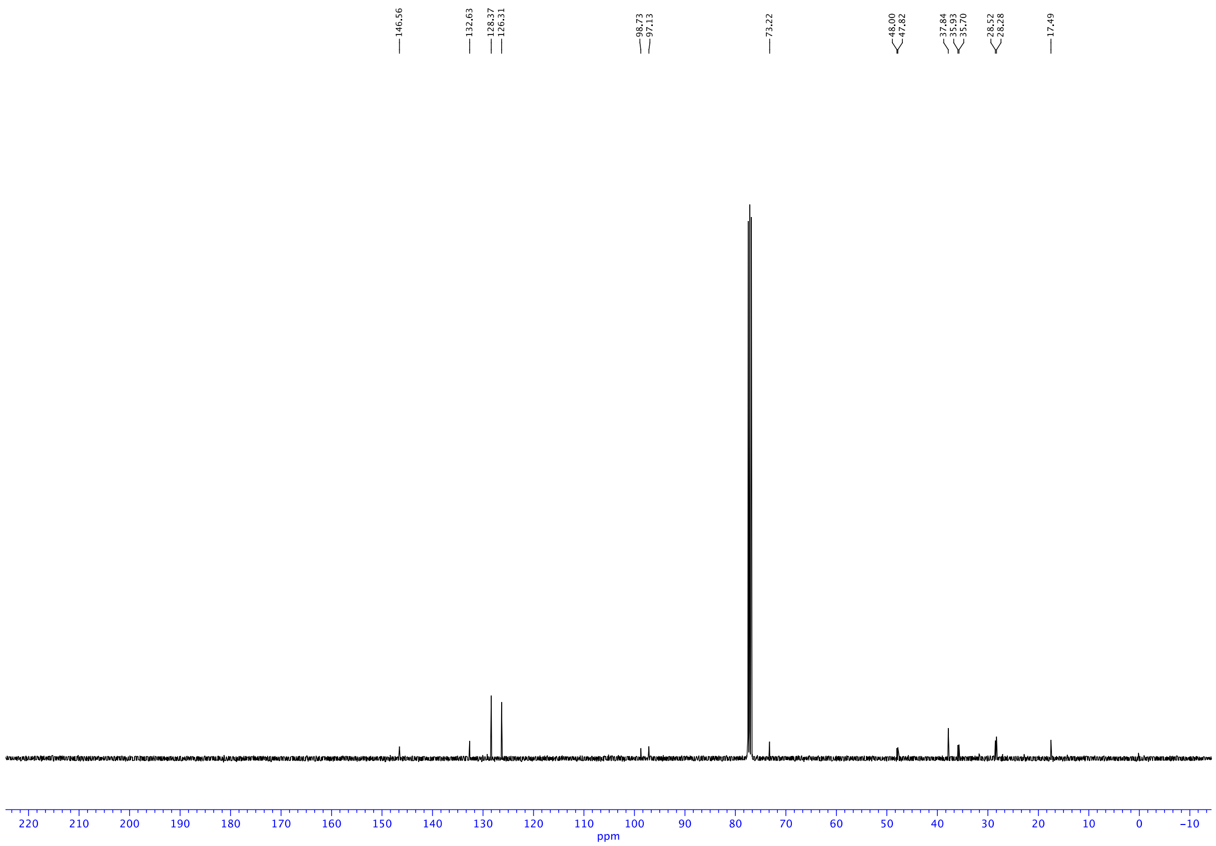

Fig. S21. $^{13}C\{^1H\}$ NMR spectrum ($CDCl_3$, 100 MHz) of compound **6**.

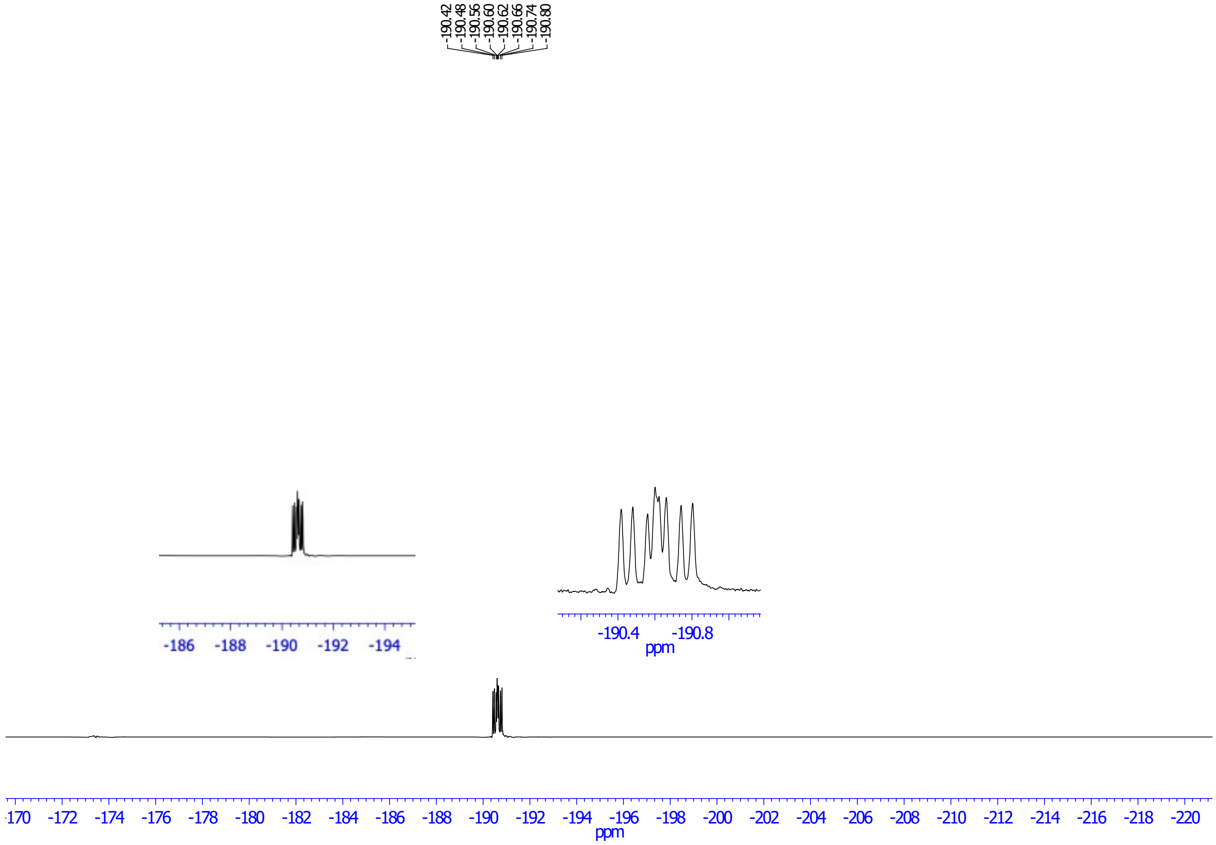

Fig. S22. ^{19}F NMR spectrum ($CDCl_3$, 282 MHz) of compound **7**.

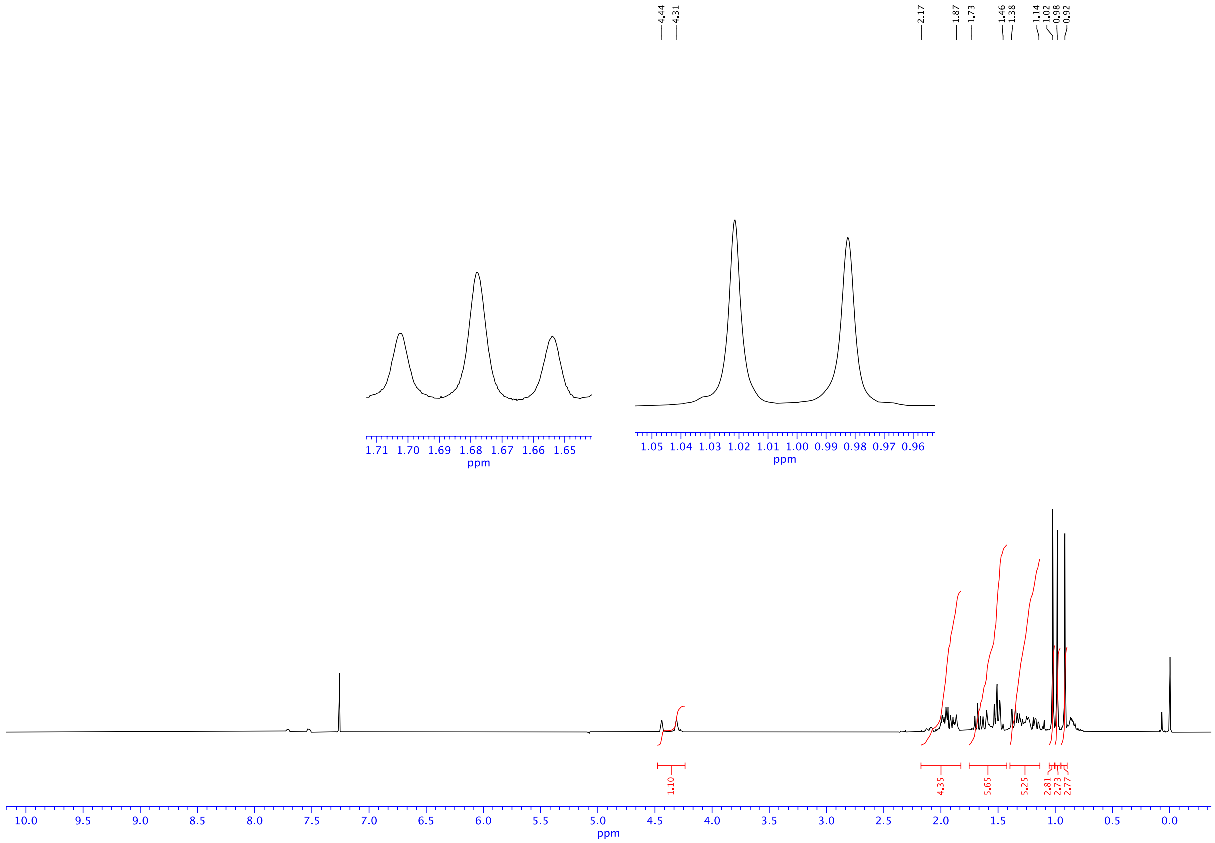

Fig. S23. ^{1}H NMR spectrum ($CDCl_3$, 400 MHz) of compound **7**.

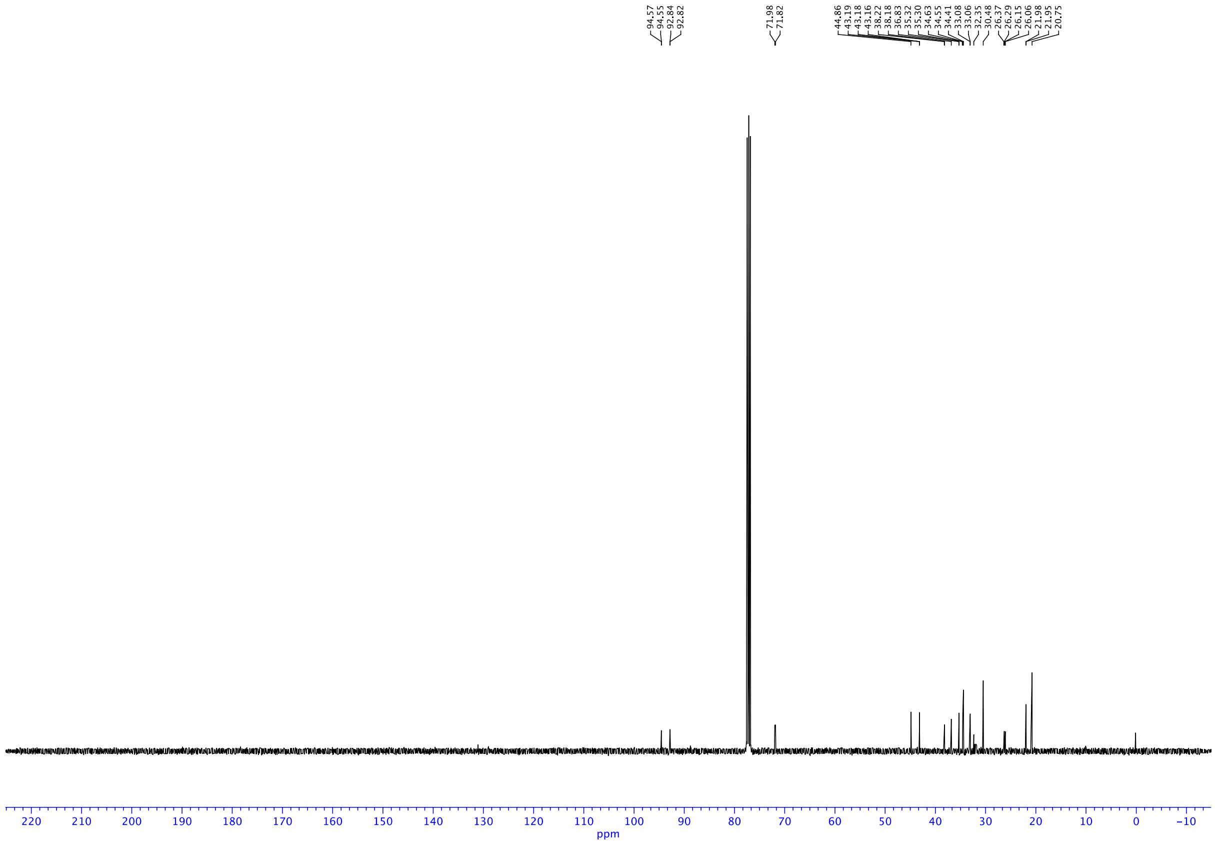

Fig. S24. $^{13}C\{^{1}H\}$ NMR spectrum ($CDCl_3$, 100 MHz) of compound **7**.

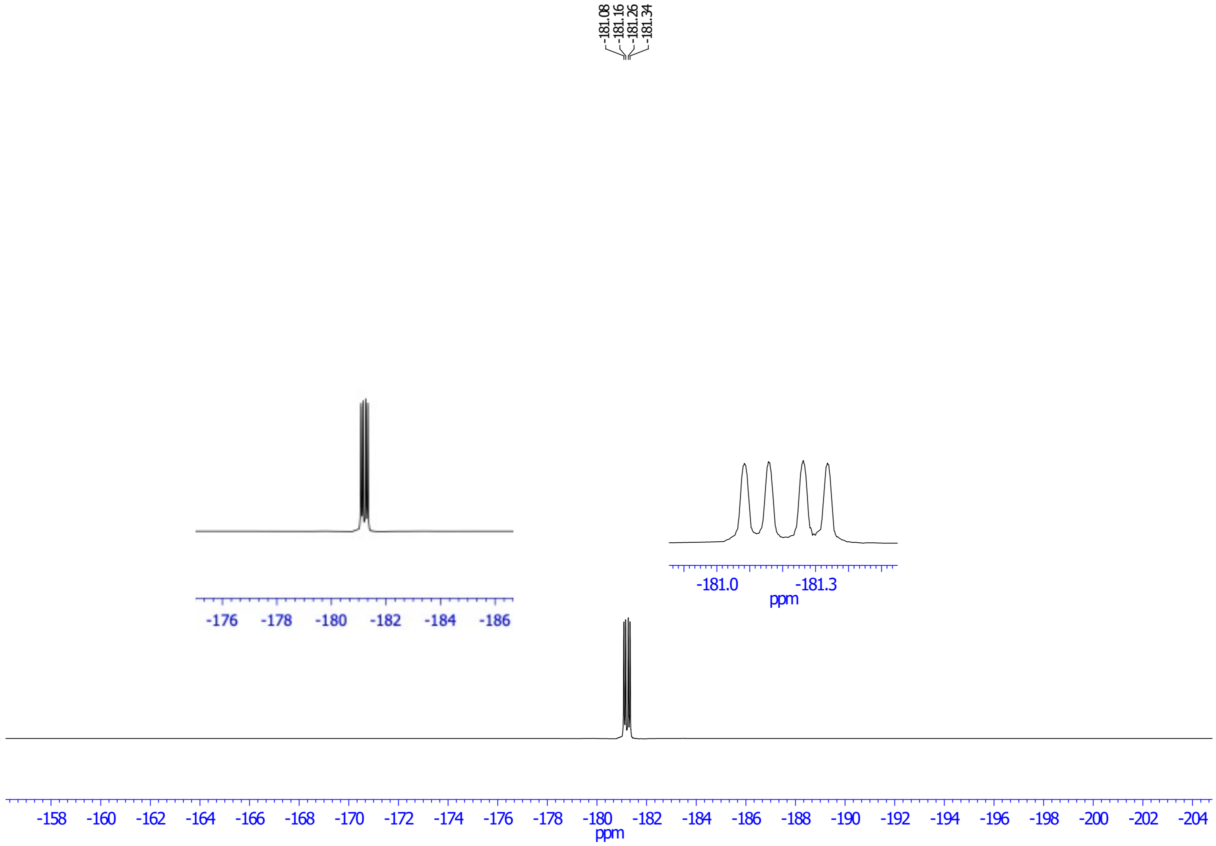

Fig. S25. ^{19}F NMR spectrum ($CDCl_3$, 282 MHz) of compound **8**.

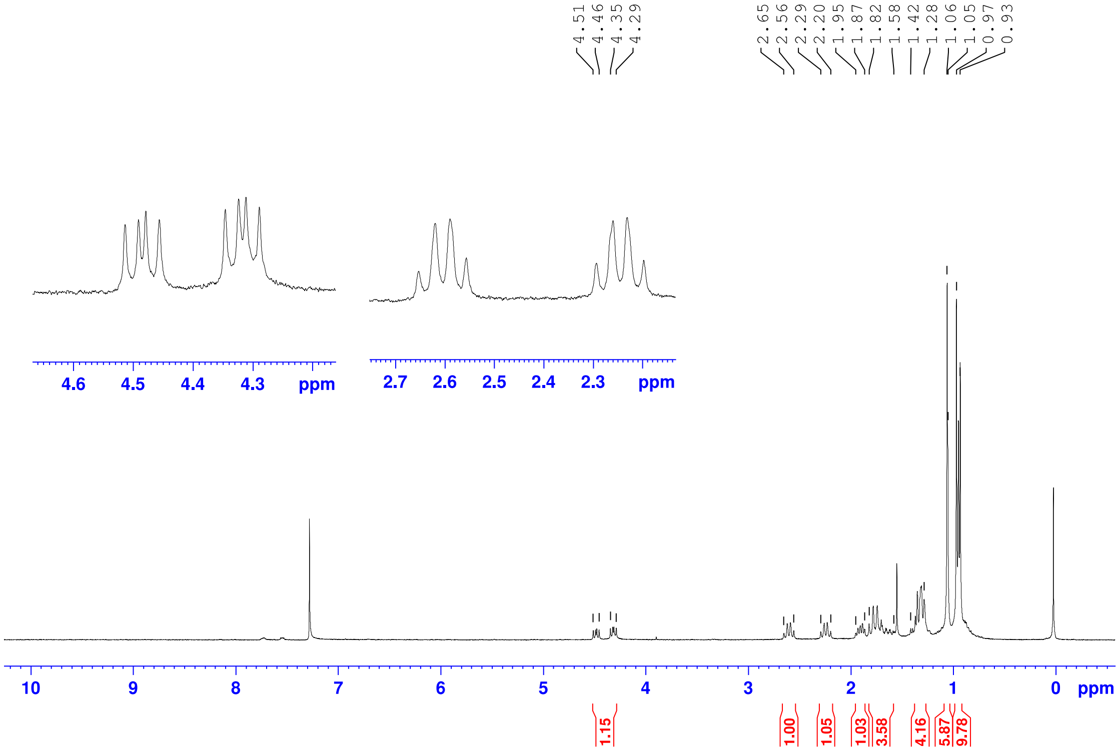

Fig. S26. ^{1}H NMR spectrum ($CDCl_3$, 400 MHz) of compound **8**.

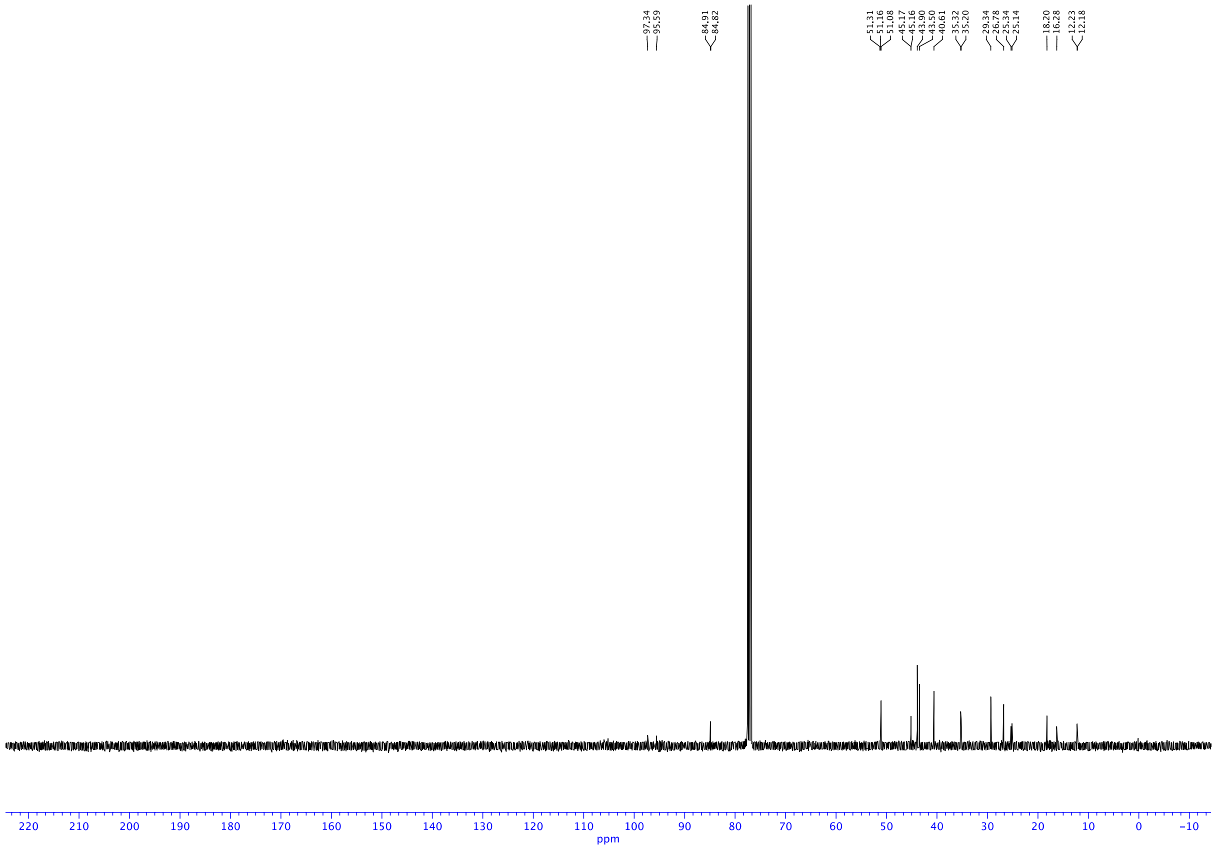

Fig. S27. $^{13}C\{^{1}H\}$ NMR spectrum ($CDCl_3$, 100 MHz) of compound **8**.

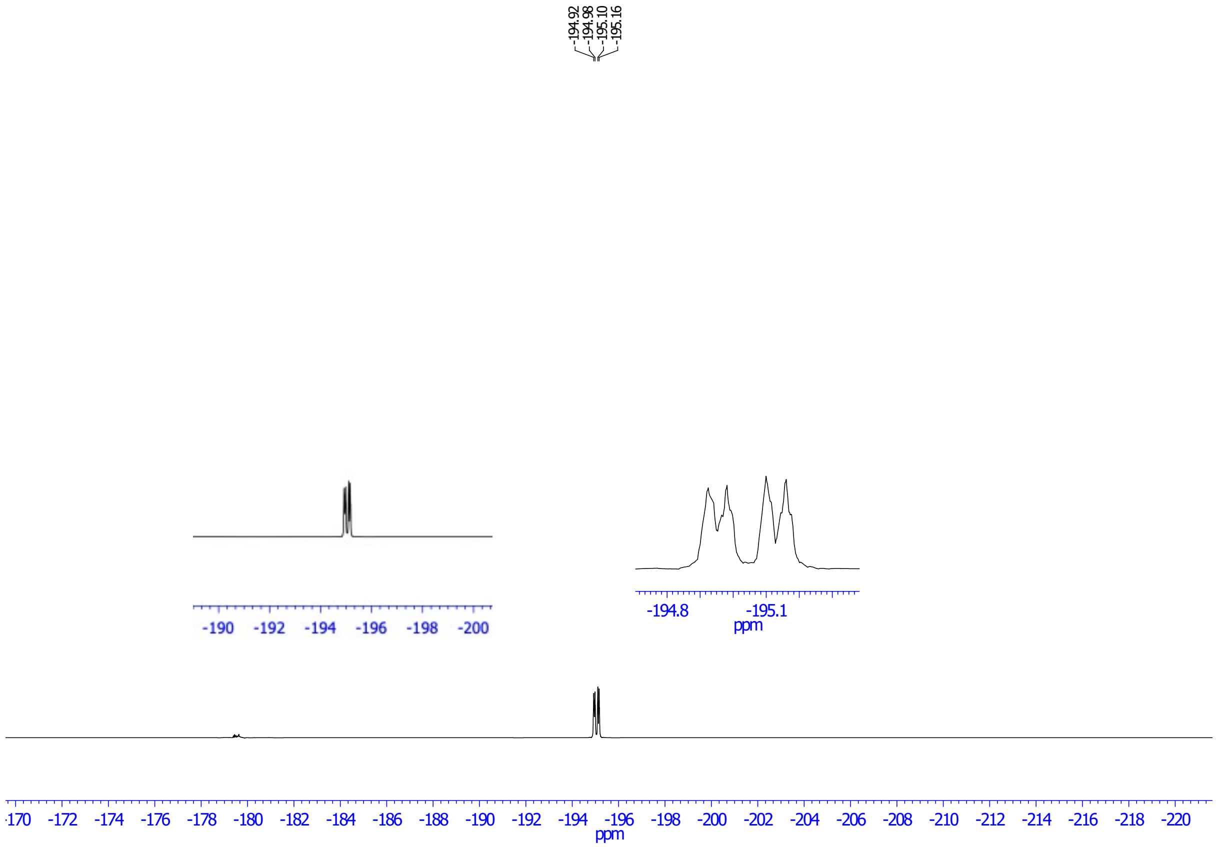

Fig. S28. ^{19}F NMR spectrum ($CDCl_3$, 282 MHz) of compound **9**.

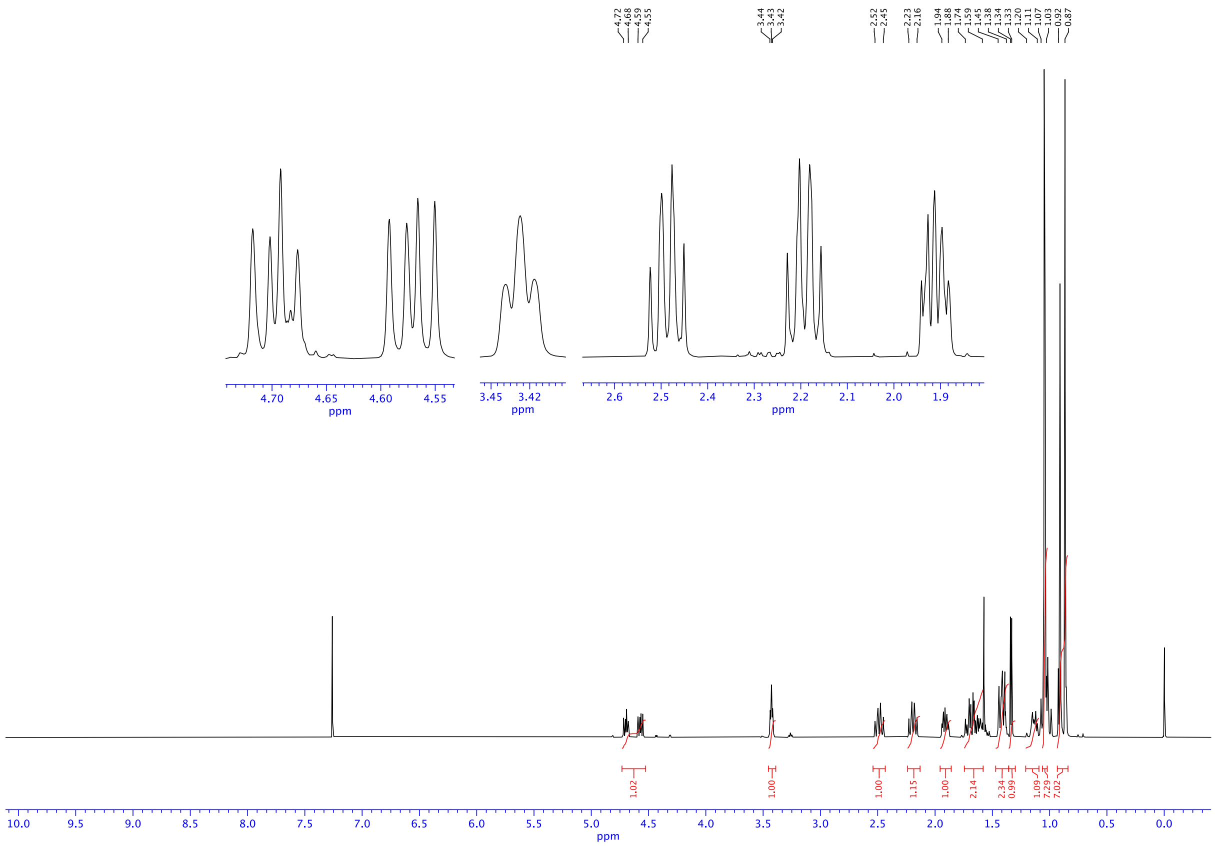

Fig. S29. ^{1}H NMR spectrum ($CDCl_3$, 400 MHz) of compound **9**.

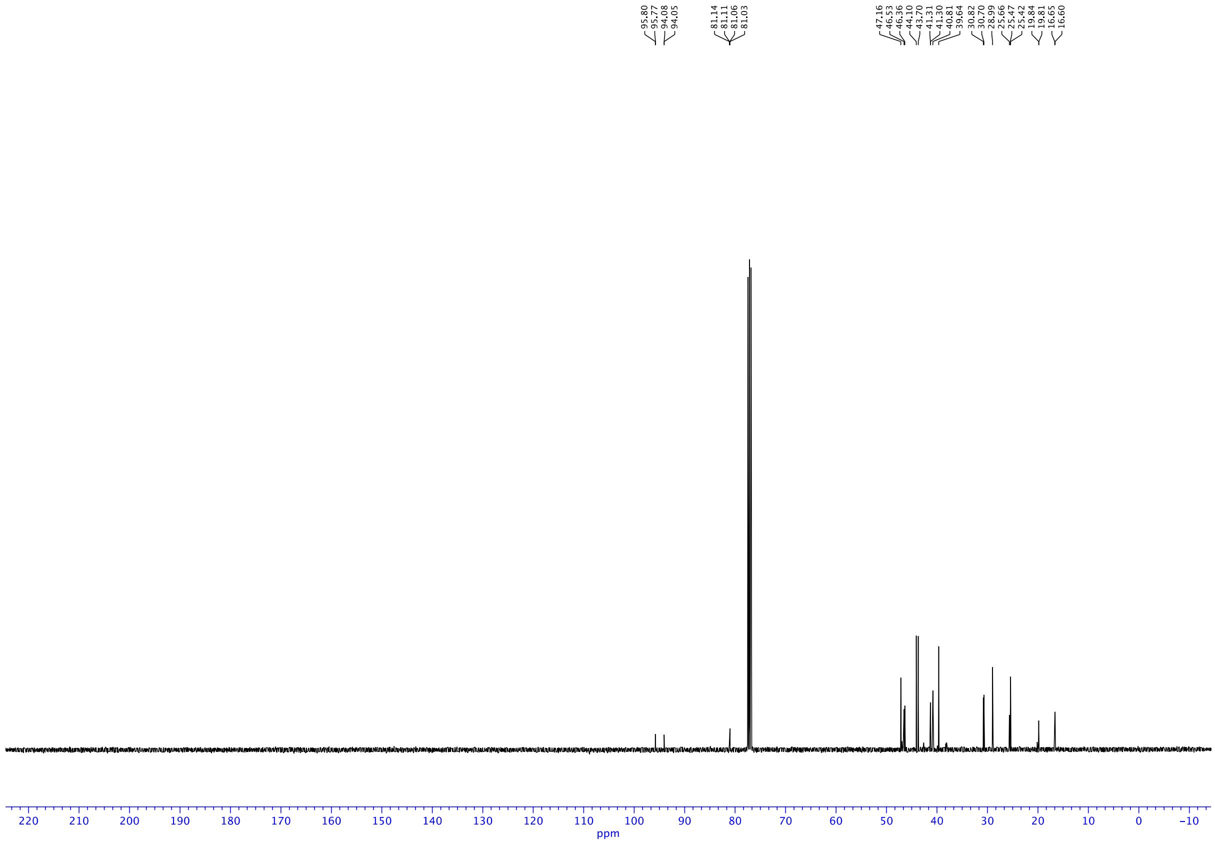

Fig. S30. $^{13}C\{^{1}H\}$ NMR spectrum ($CDCl_3$, 100 MHz) of compound **9**.

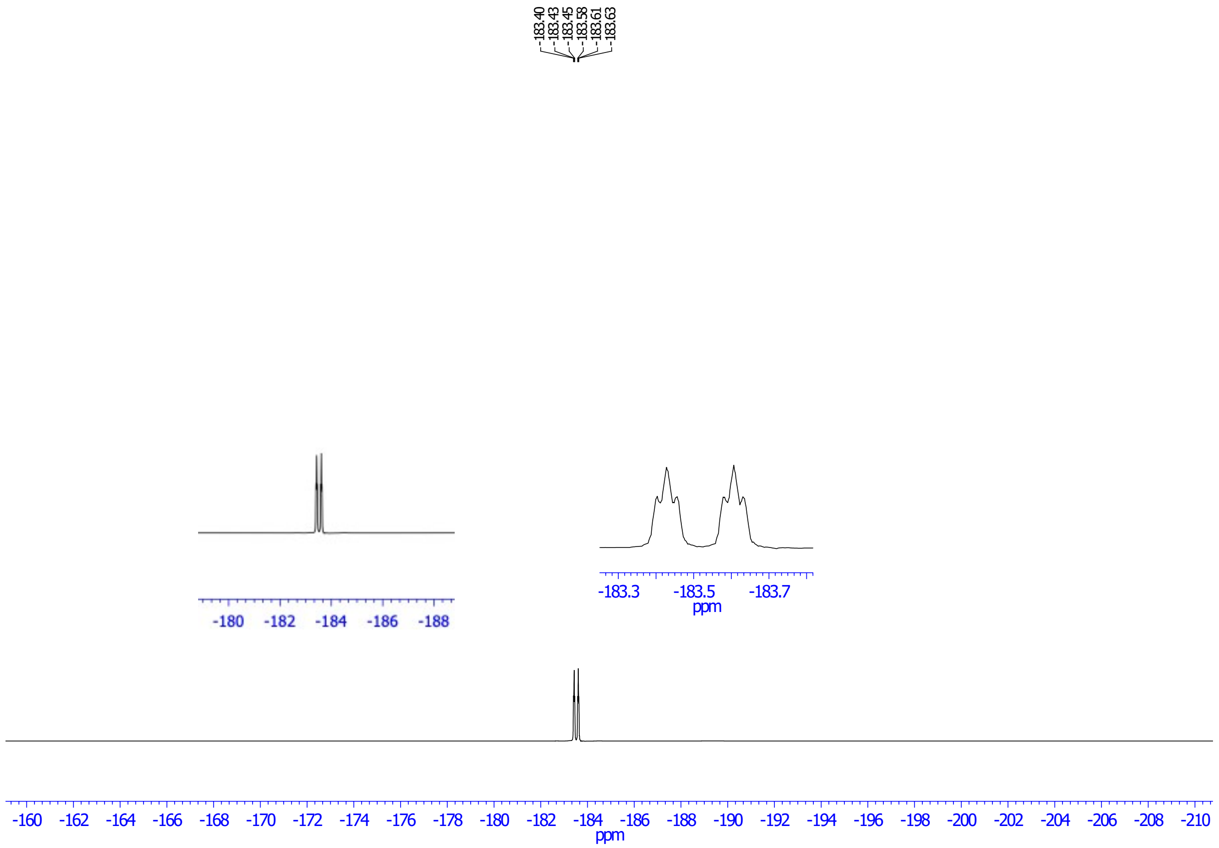

Fig. S31. ^{19}F NMR spectrum ($CDCl_3$, 282 MHz) of compound **10**.

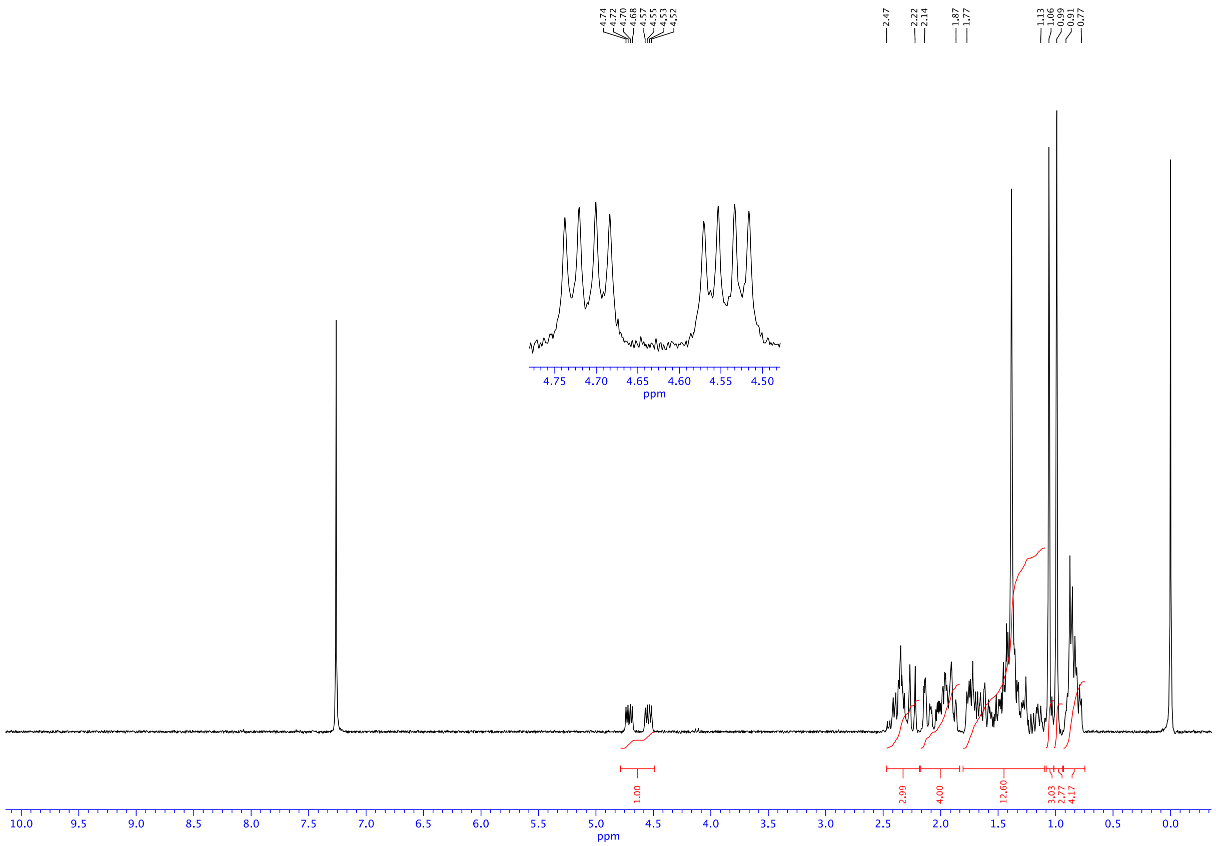

Fig. S32. ^{1}H NMR spectrum ($CDCl_3$, 400 MHz) of compound **10**.

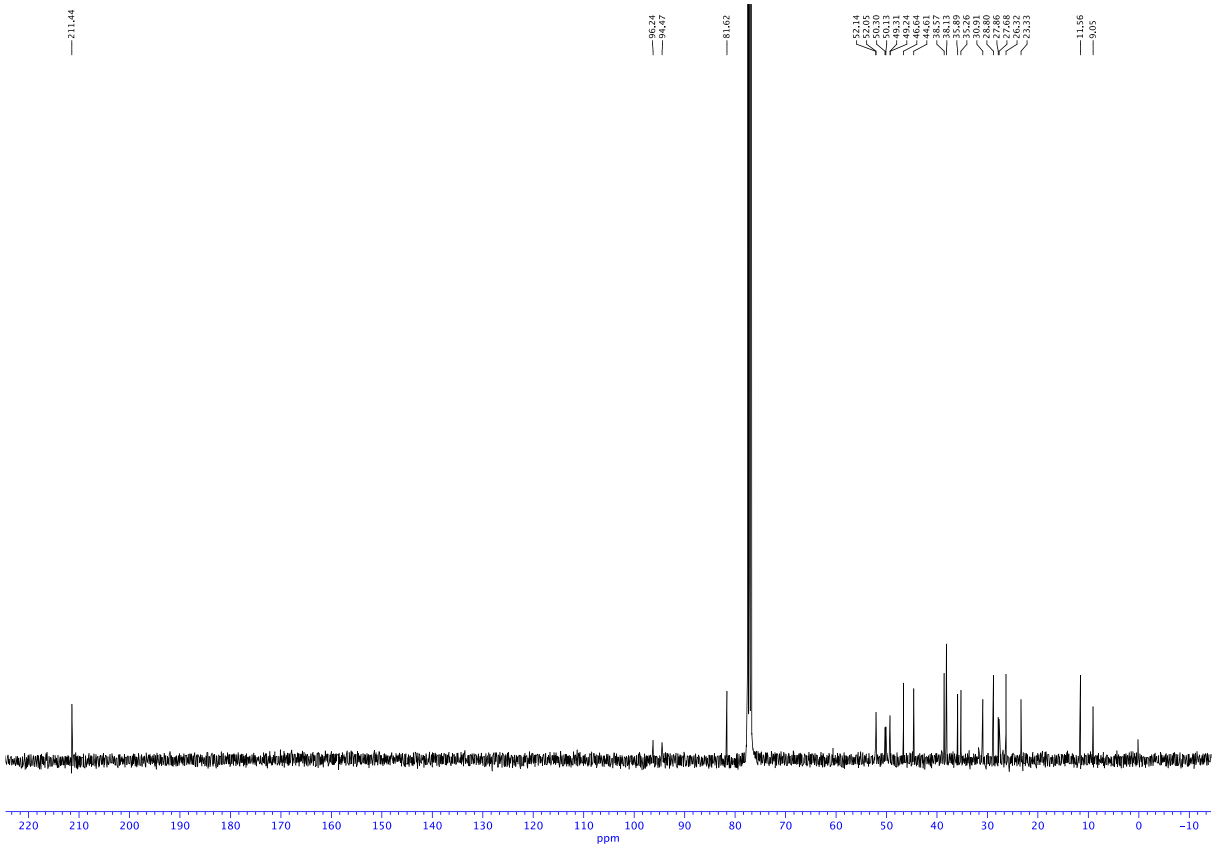

Fig. S33. $^{13}C\{^1H\}$ NMR spectrum ($CDCl_3$, 100 MHz) of compound **10**.

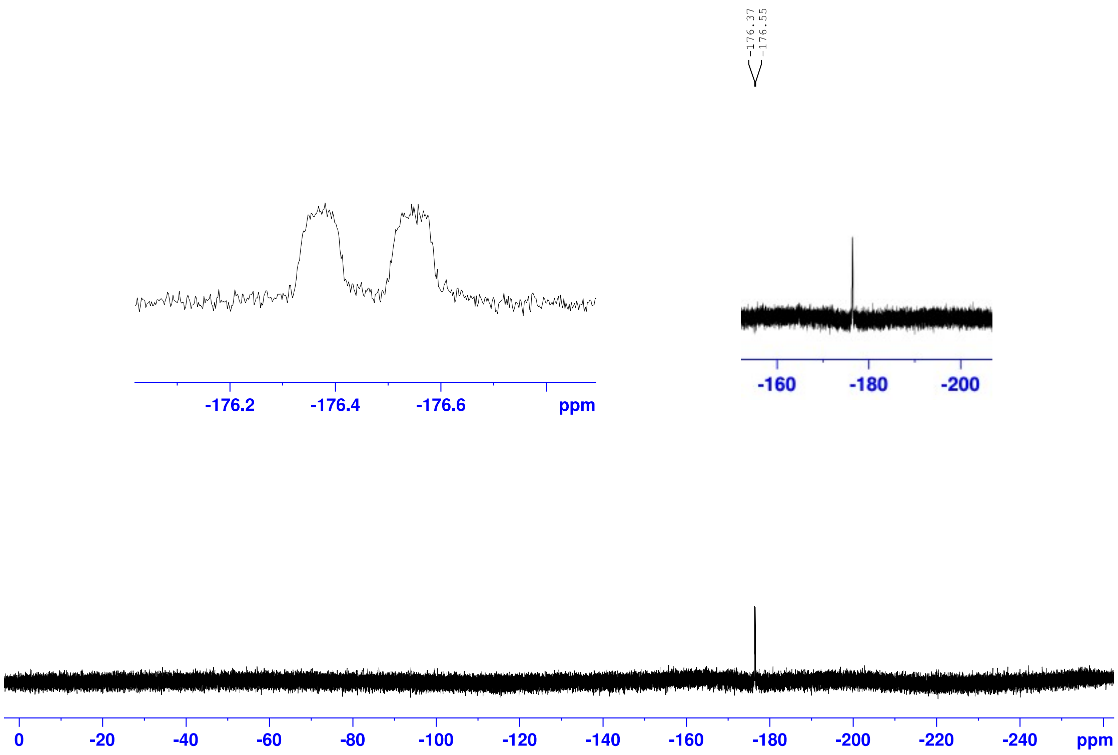

Fig. S34. ^{19}F NMR spectrum ($CDCl_3$, 282 MHz) of compound **11**.

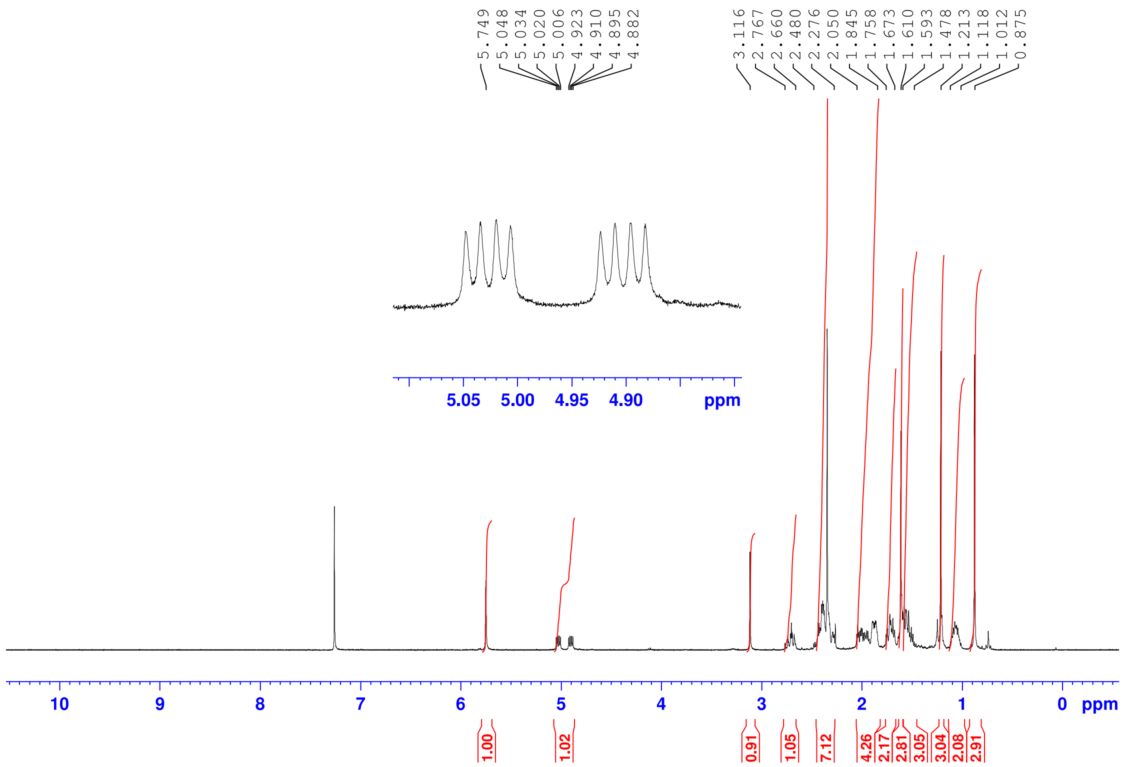

Fig. S35. ^{1}H NMR spectrum ($CDCl_3$, 400 MHz) of compound **11**.

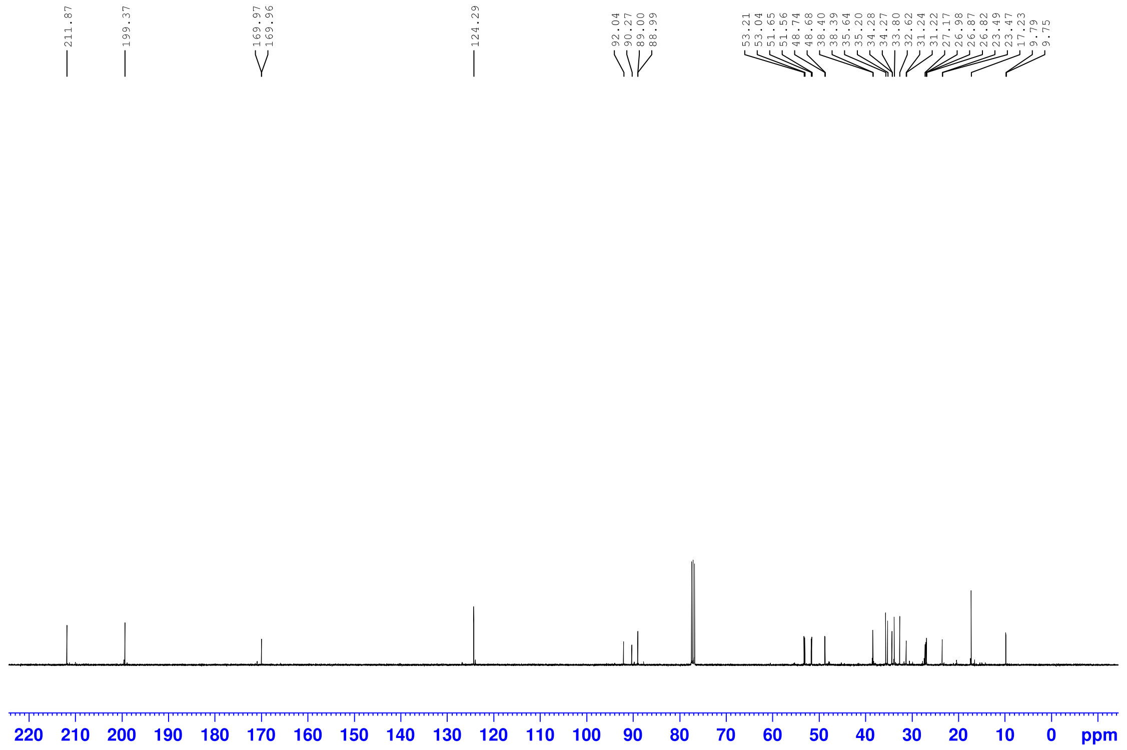

Fig. S36. $^{13}C\{^{1}H\}$ NMR spectrum ($CDCl_3$, 100 MHz) of compound **11**.

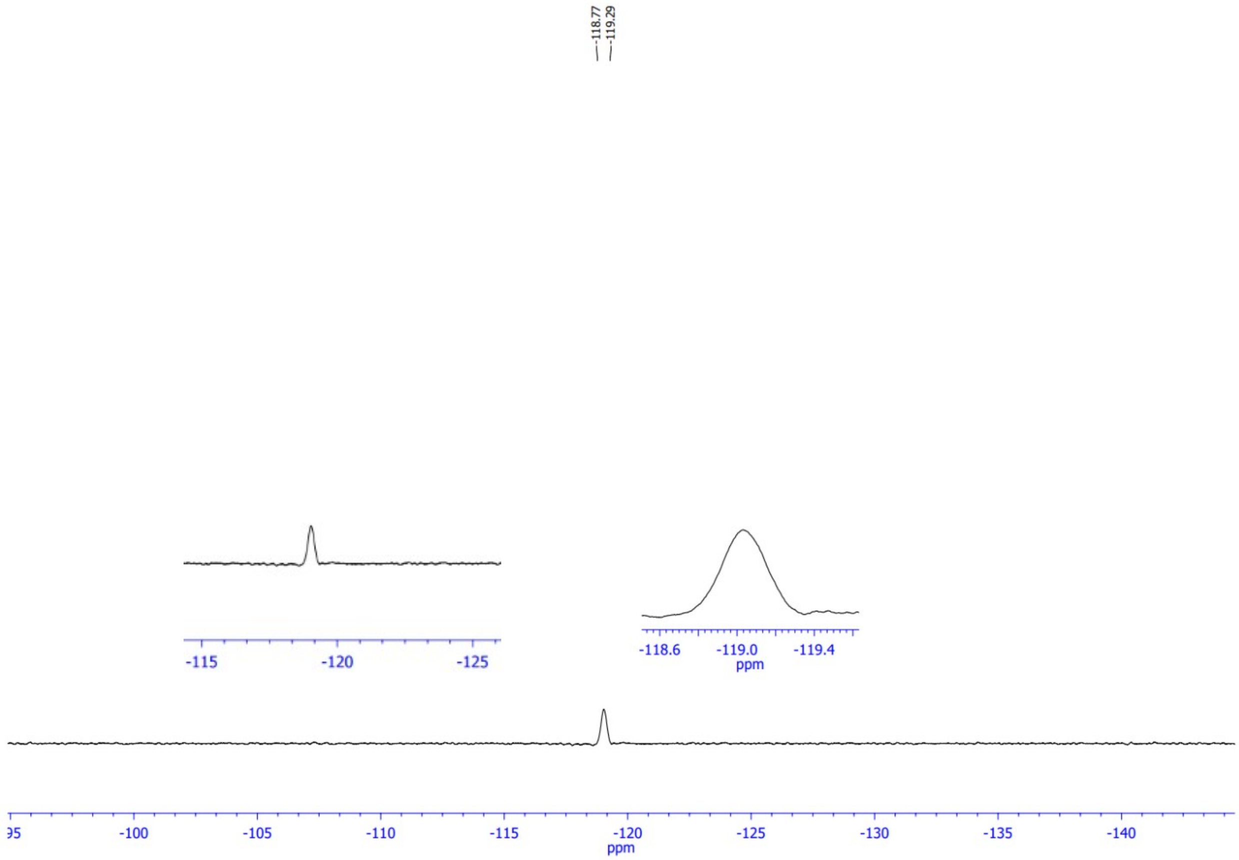

Fig. S37. ^{19}F NMR spectrum ($CDCl_3$, 282 MHz) of compound **13**.

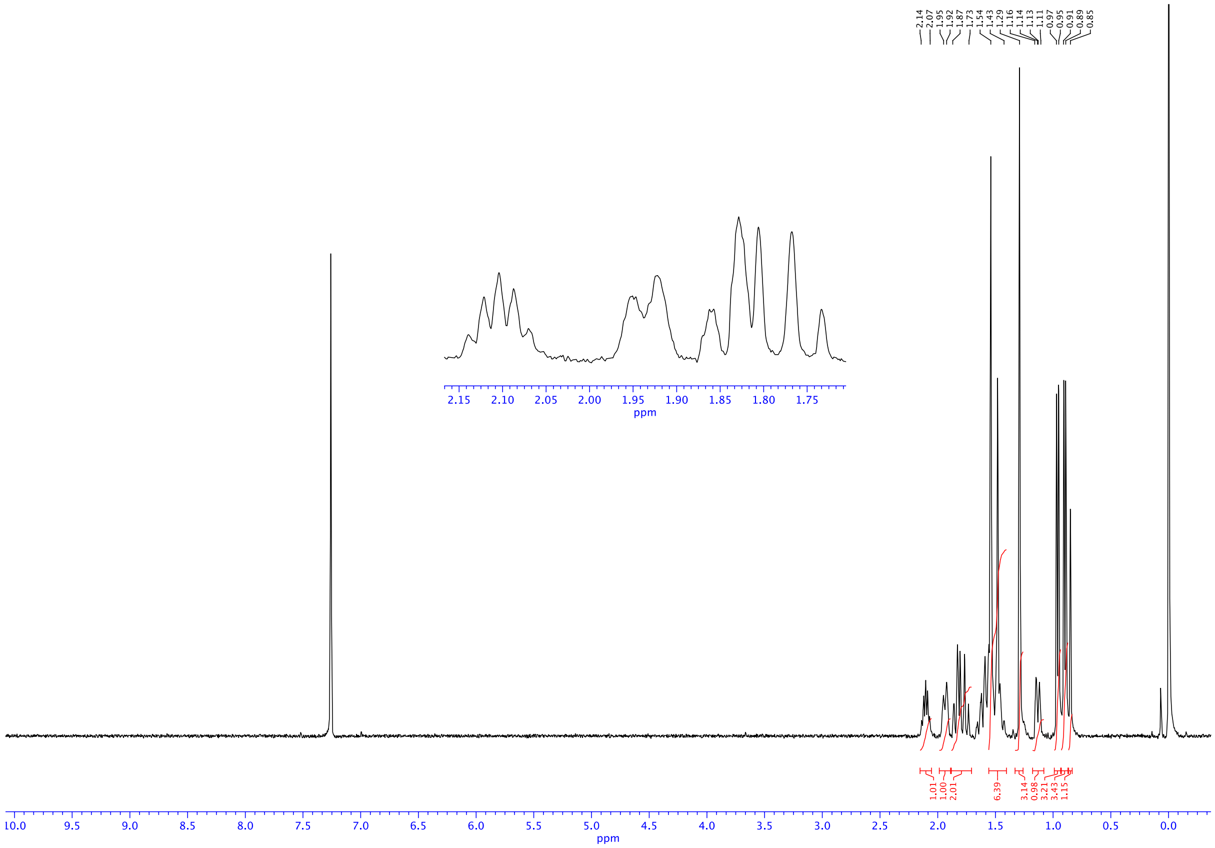

Fig. S38. ^{1}H NMR spectrum ($CDCl_3$, 400 MHz) of compound **13**.

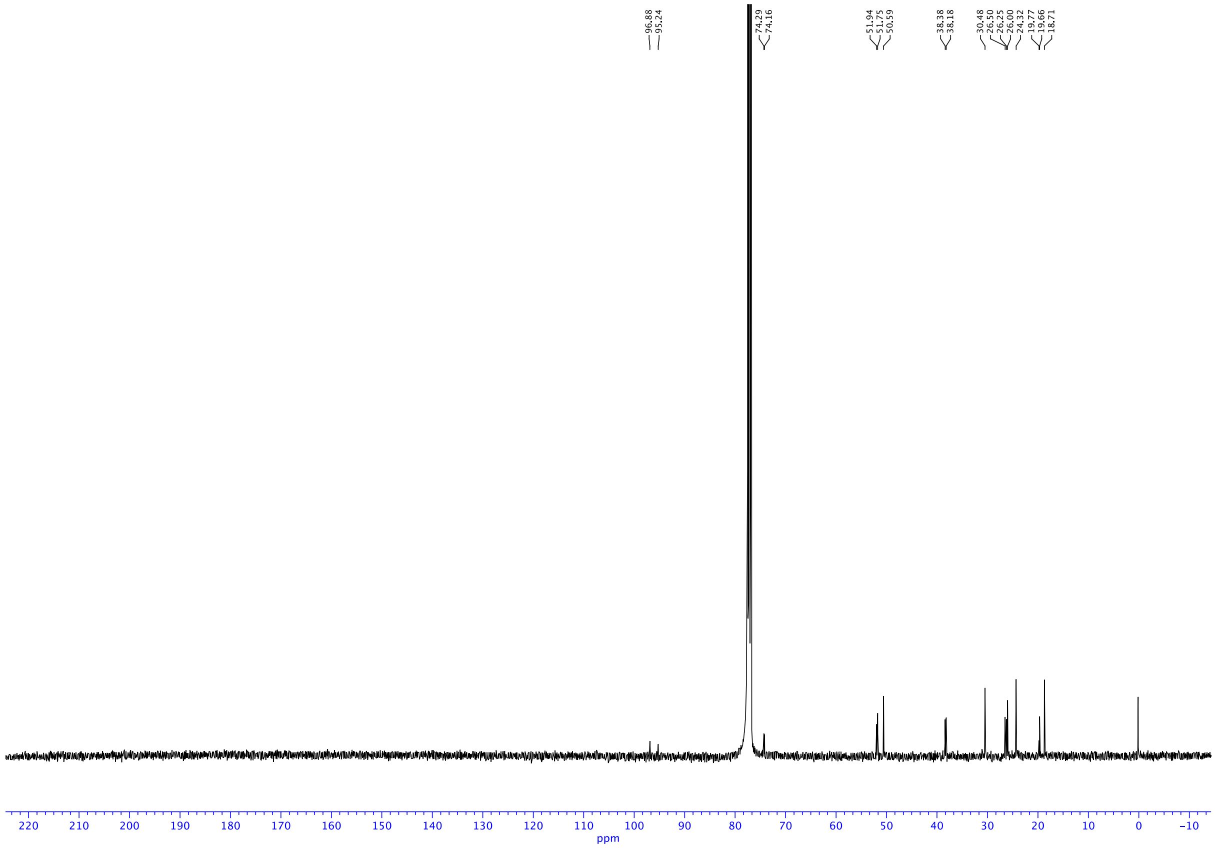

Fig. S39. $^{13}C\{^1H\}$ NMR spectrum ($CDCl_3$, 100 MHz) of compound **13**.

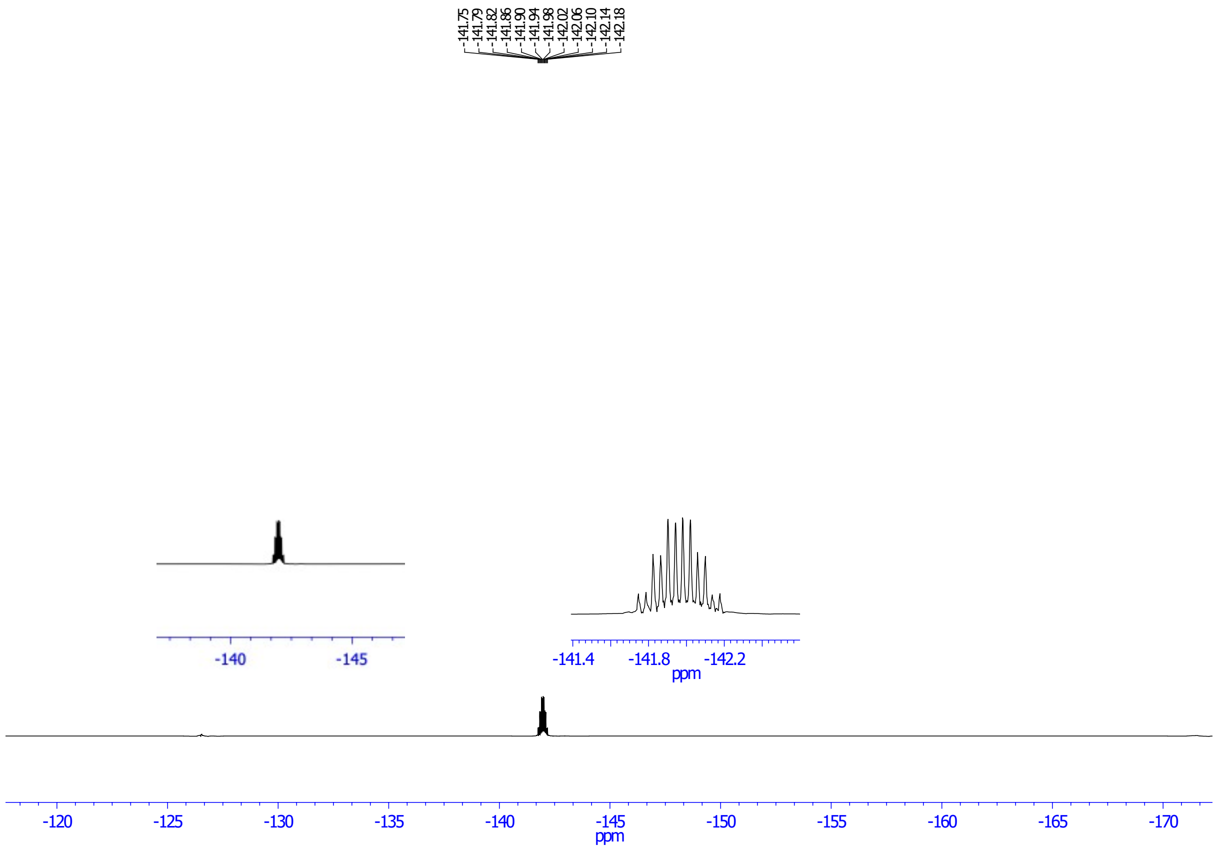

Fig. S40. ^{19}F NMR spectrum ($CDCl_3$, 282 MHz) of compound **14**.

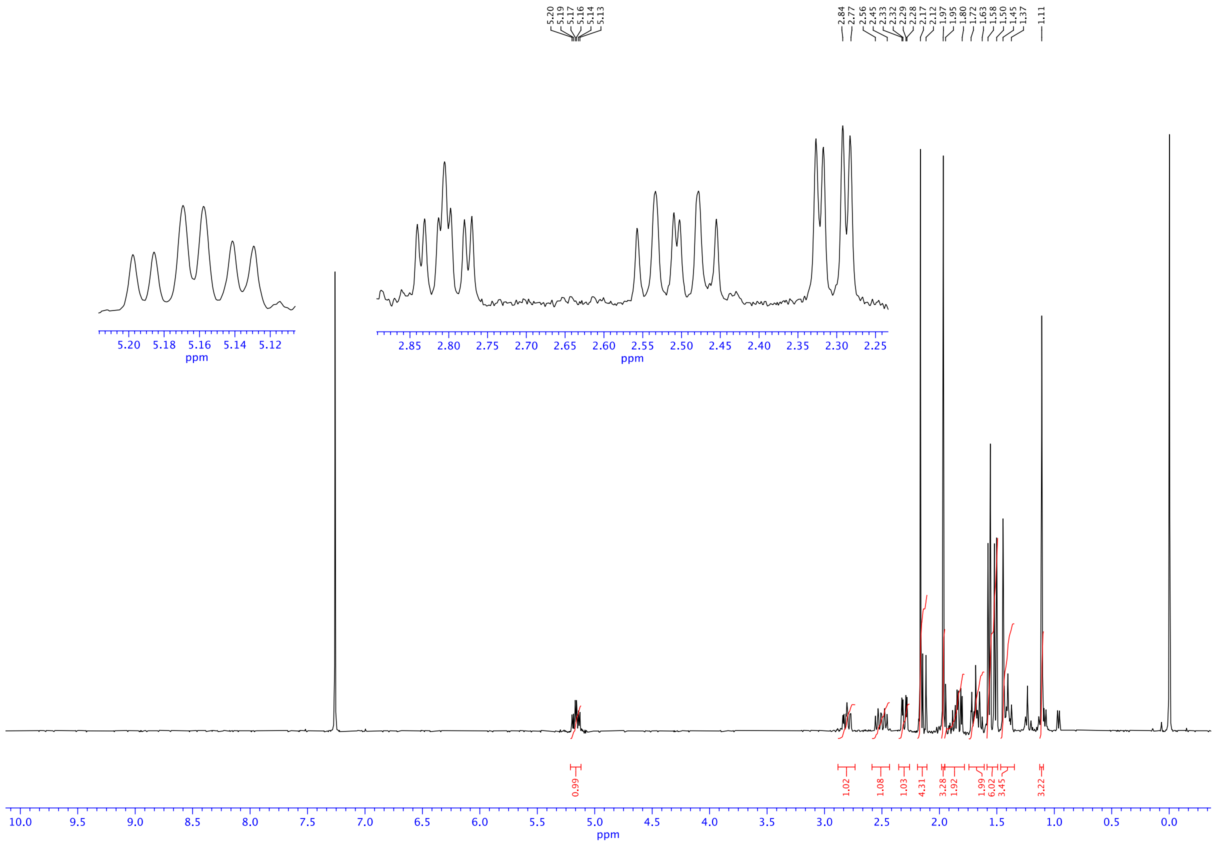

Fig. S41. ^1H NMR spectrum ($CDCl_3$, 400 MHz) of compound **14**.

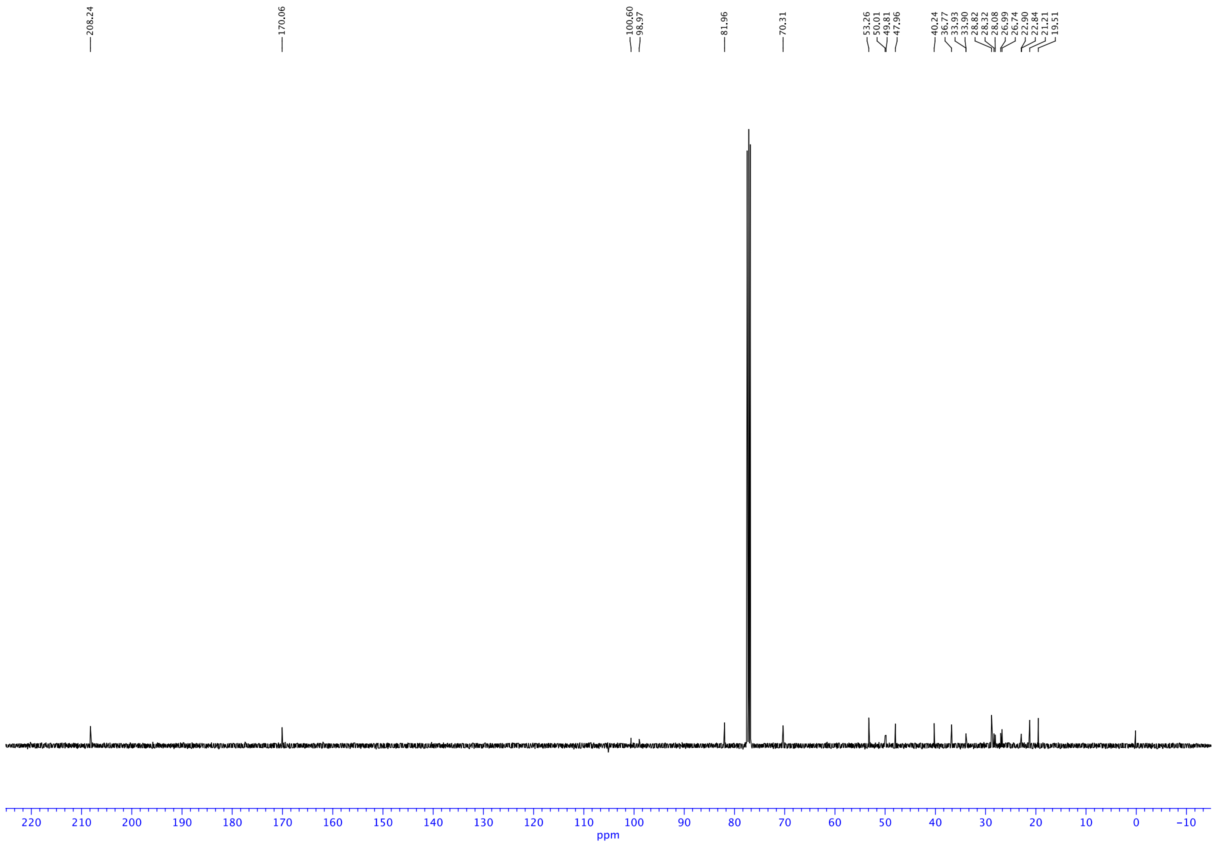

Fig. S42. $^{13}C\{^{1}H\}$ NMR spectrum ($CDCl_3$, 100 MHz) of compound **14**.

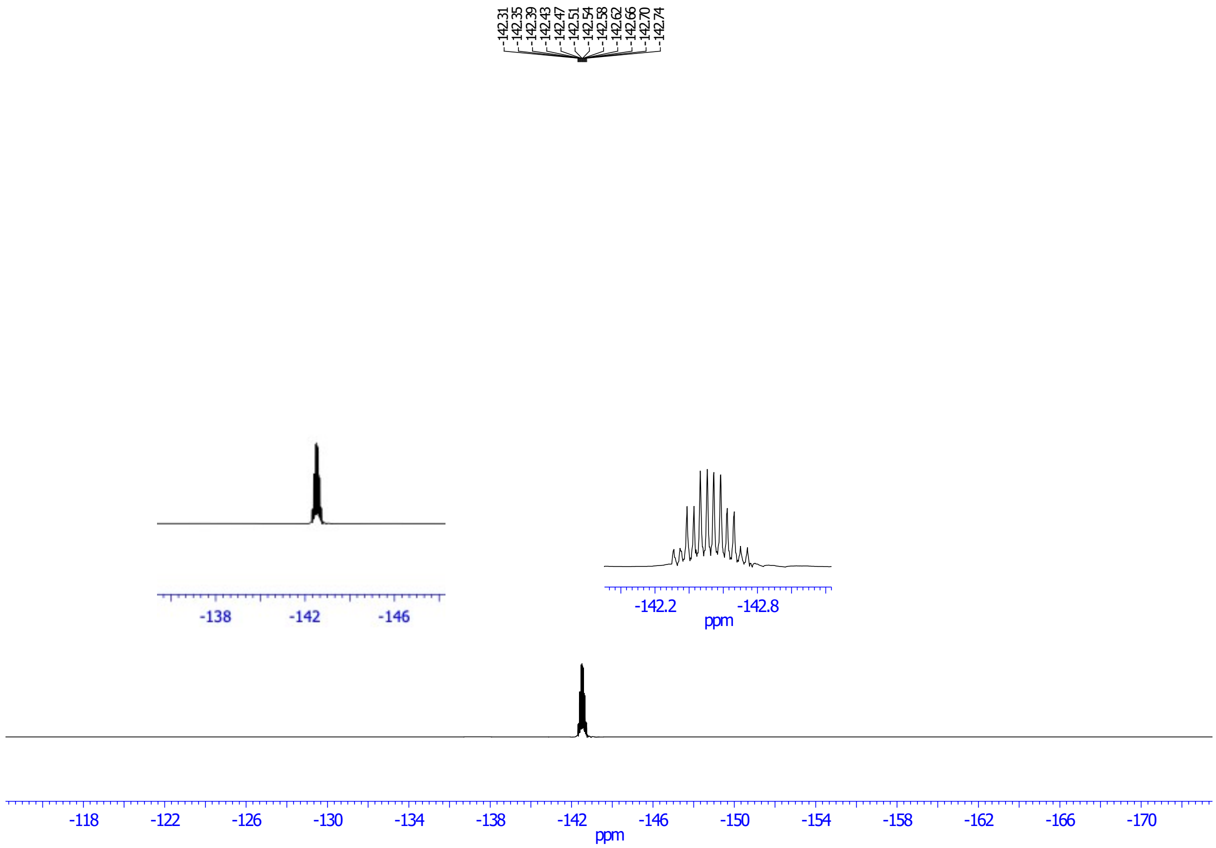

Fig. S43. ^{19}F NMR spectrum ($CDCl_3$, 282 MHz) of compound **15**.

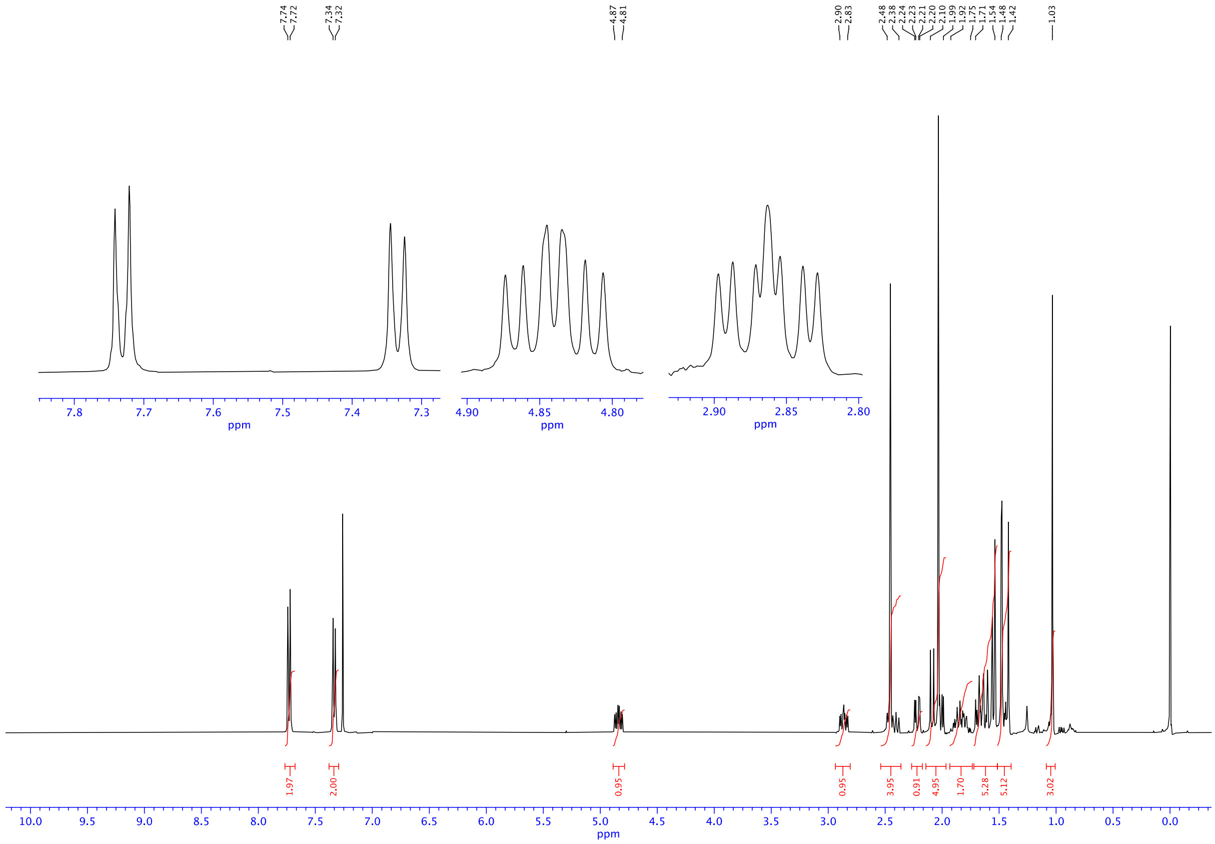

Fig. S44. ^{1}H NMR spectrum ($CDCl_3$, 400 MHz) of compound **15**.

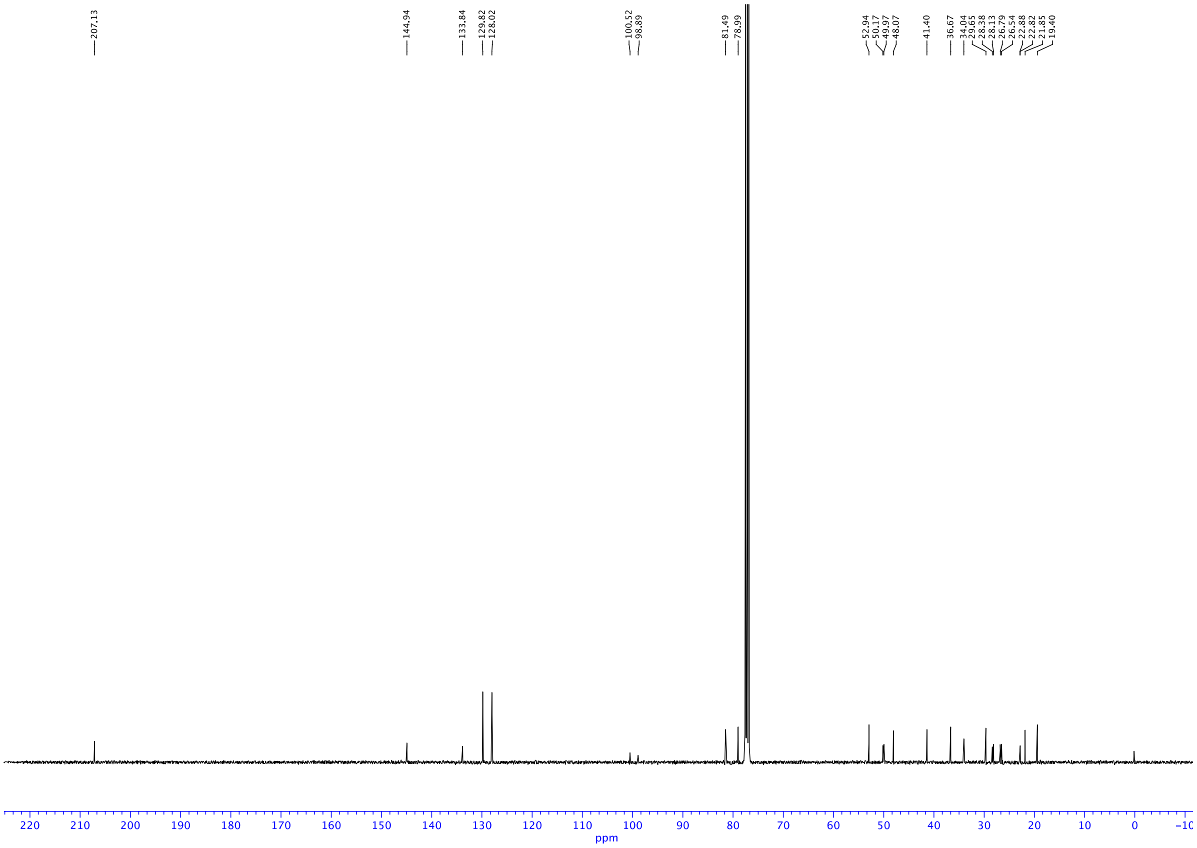

Fig. S45. $^{13}C\{^1H\}$ NMR spectrum ($CDCl_3$, 100 MHz) of compound **15**.

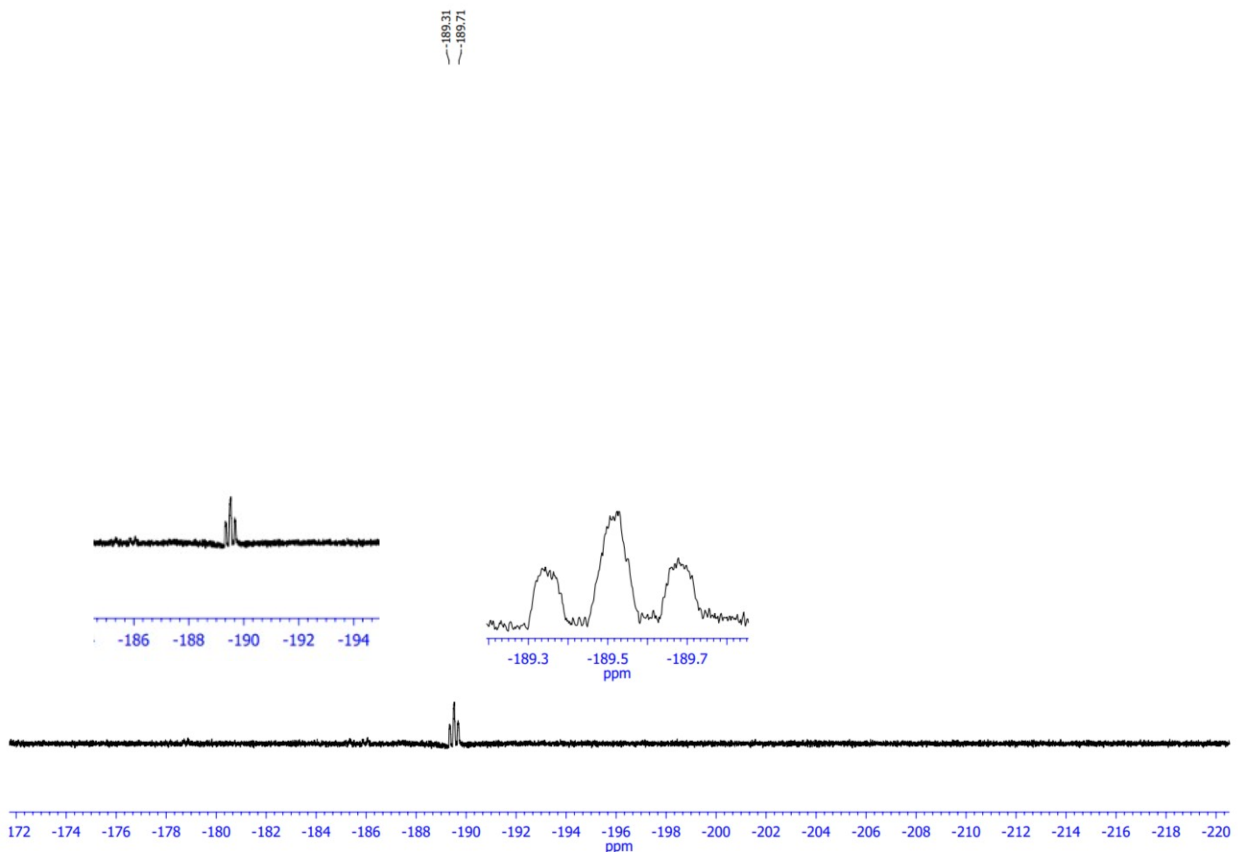

Fig. S46. ^{19}F NMR spectrum ($CDCl_3$, 282 MHz) of compound **16**.

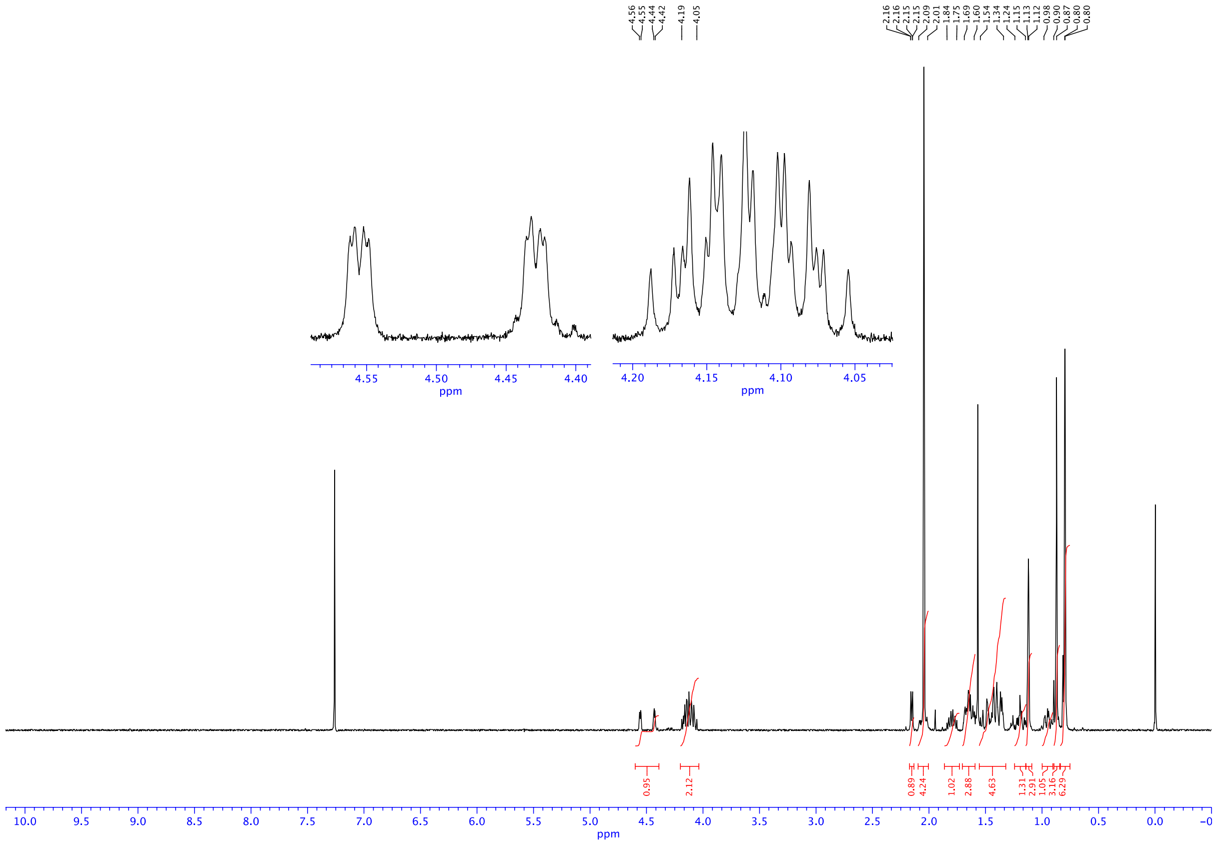

Fig. S47. ^{1}H NMR spectrum ($CDCl_3$, 400 MHz) of compound **16**.

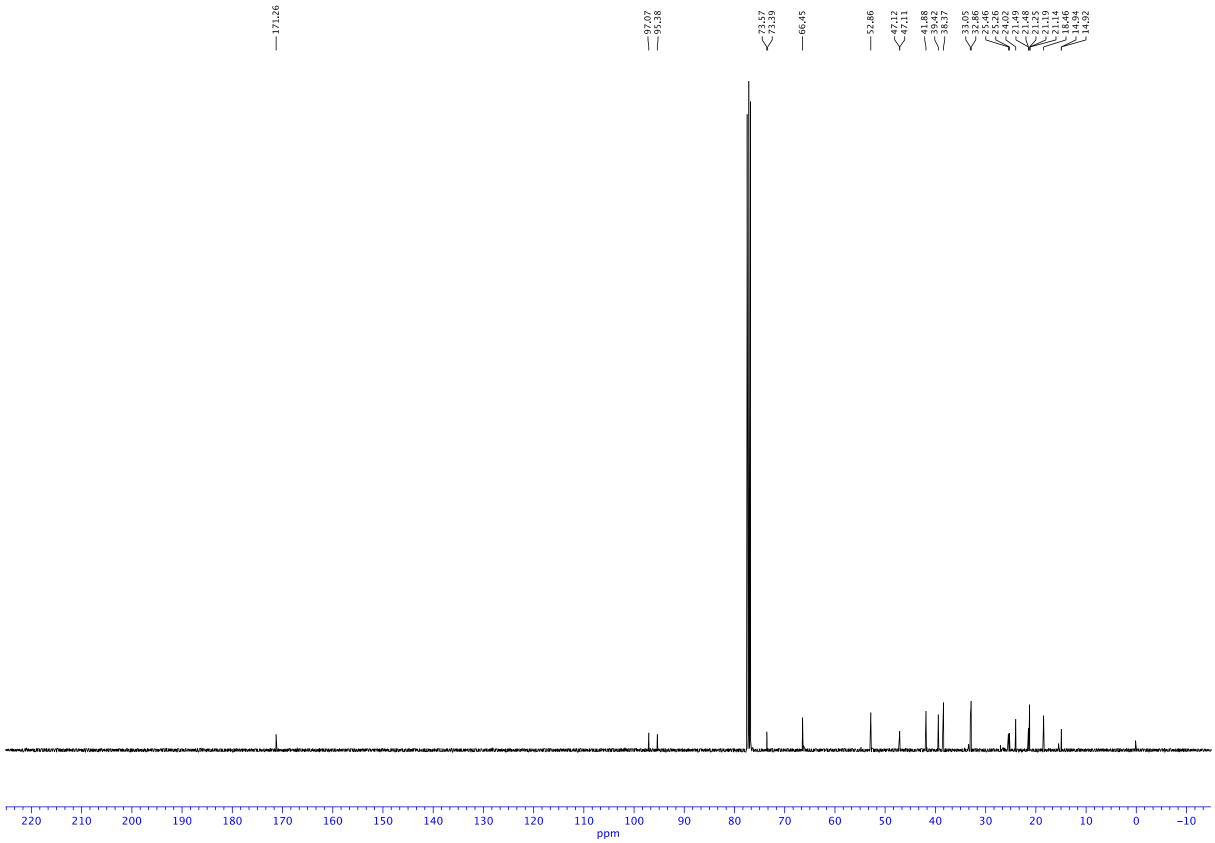

Fig. S48. $^{13}C\{^{1}H\}$ NMR spectrum ($CDCl_3$, 100 MHz) of compound **16**.

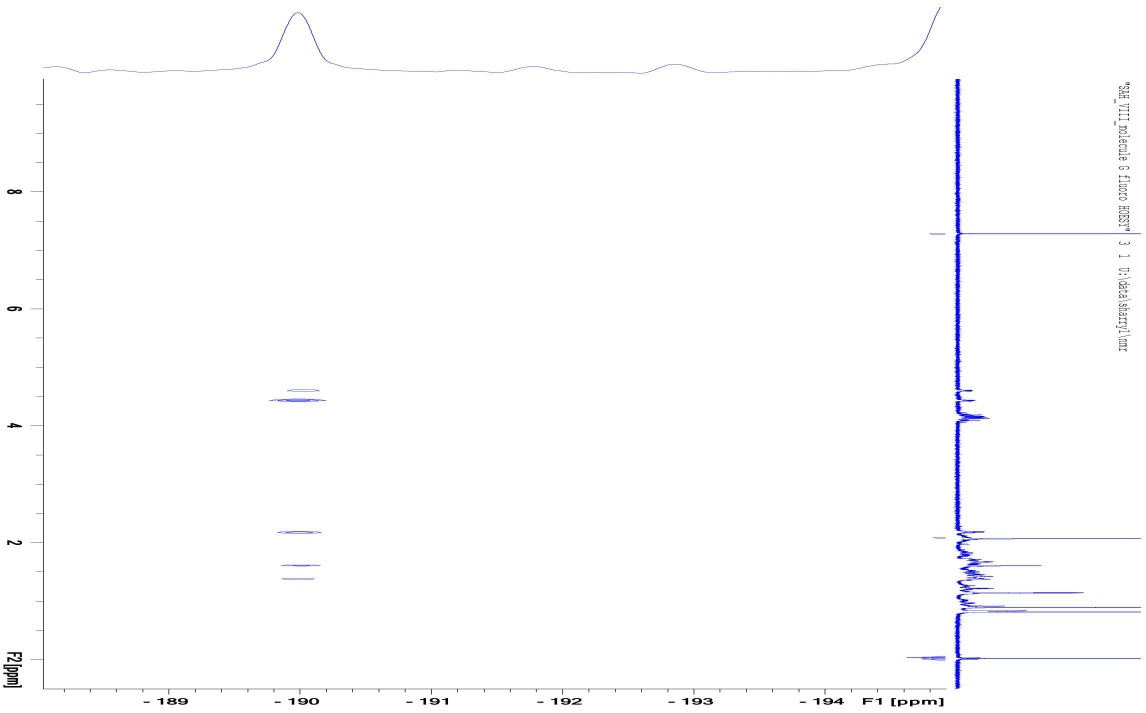

Fig. S49. HOESY spectrum ($CDCl_3$, 300 MHz) of compound **16**.

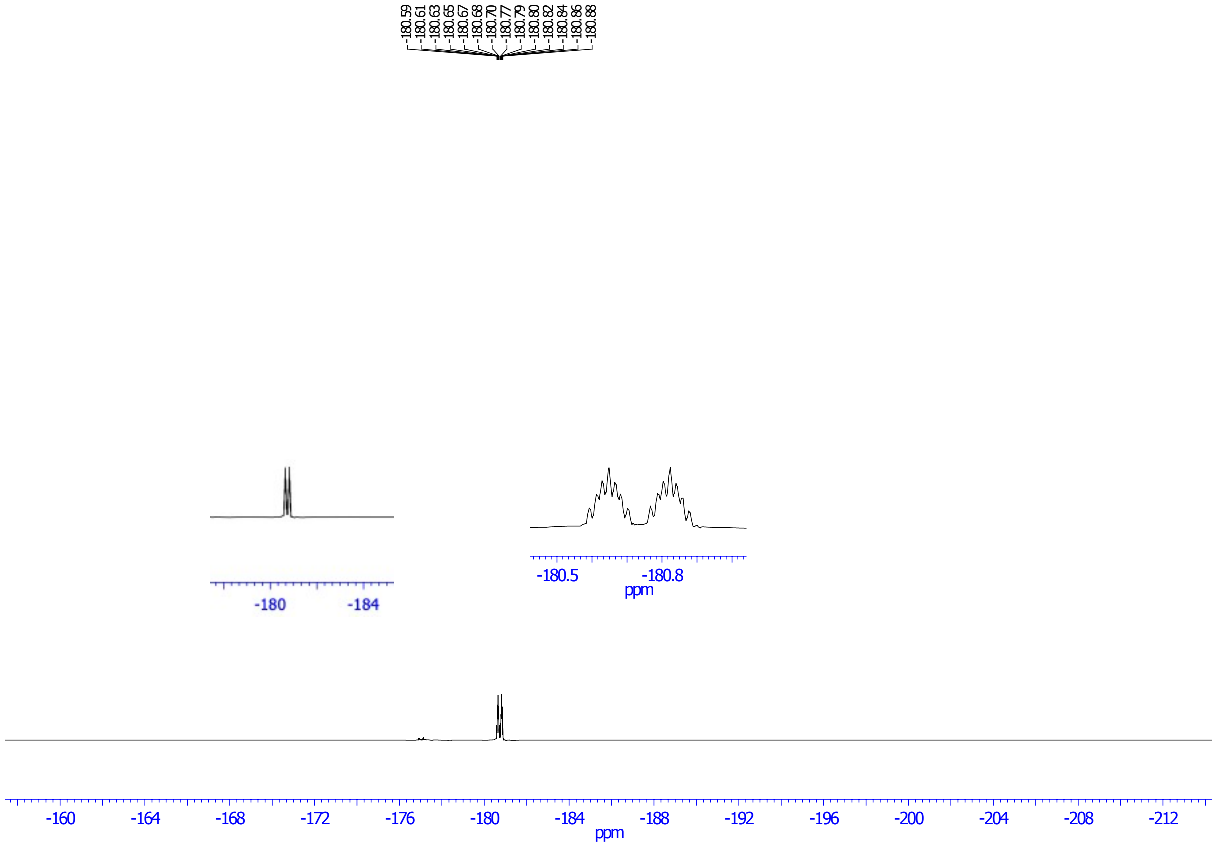

Fig. S50. ^{19}F NMR spectrum ($CDCl_3$, 282 MHz) of compound **17**.

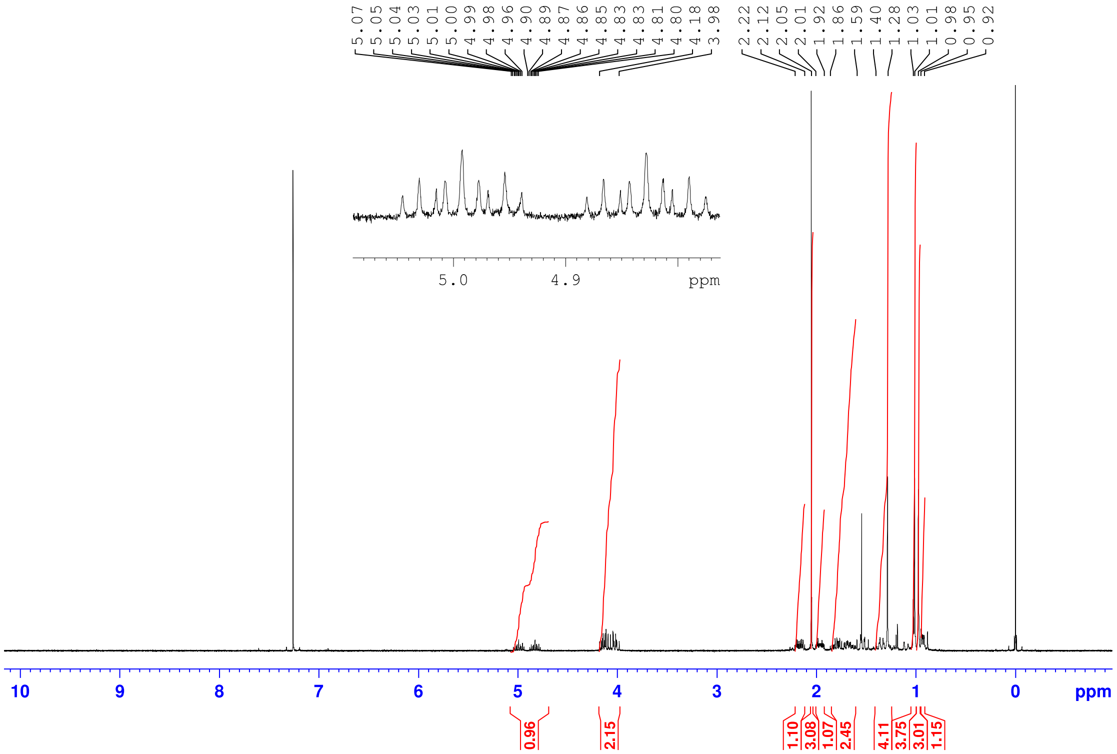

Fig. S51. ^{1}H NMR spectrum ($CDCl_3$, 400 MHz) of compound **17**.

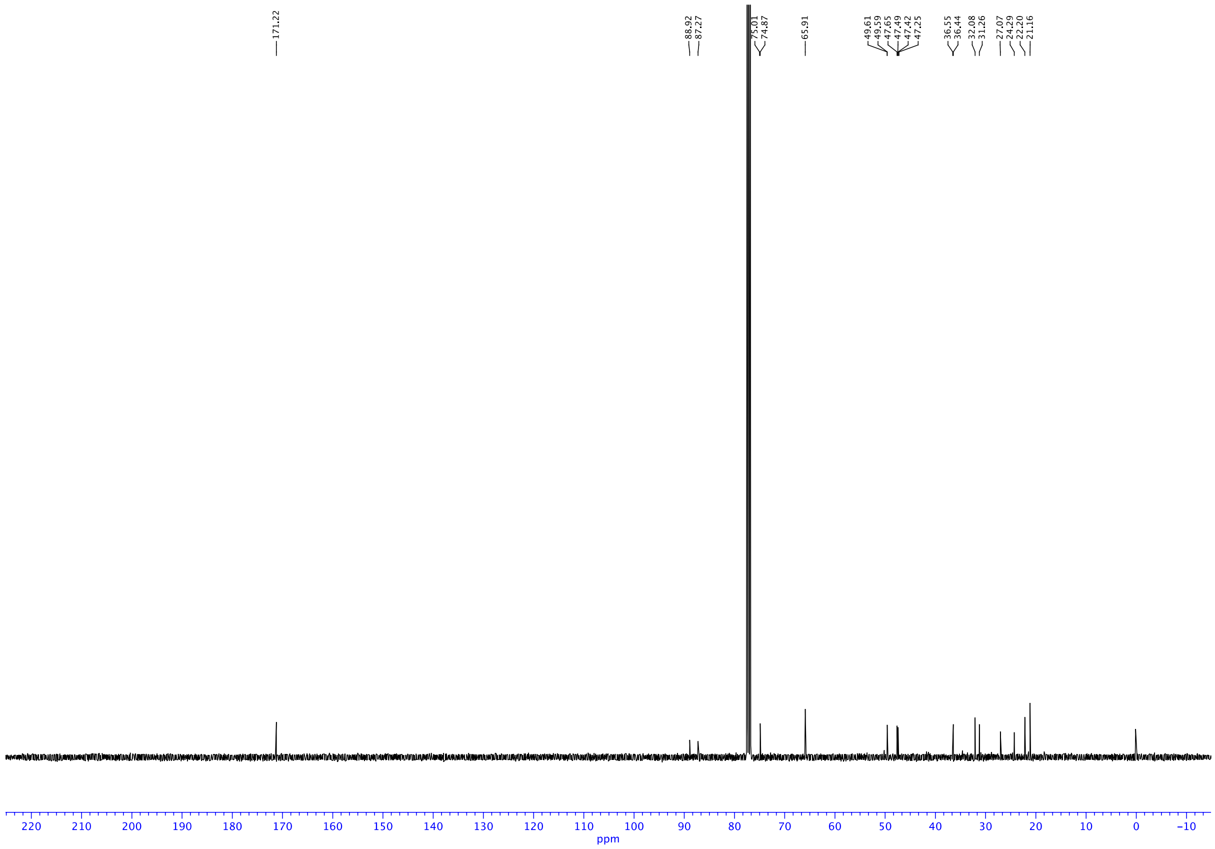

Fig. S52. $^{13}C\{^{1}H\}$ NMR spectrum ($CDCl_3$, 100 MHz) of compound **17**.

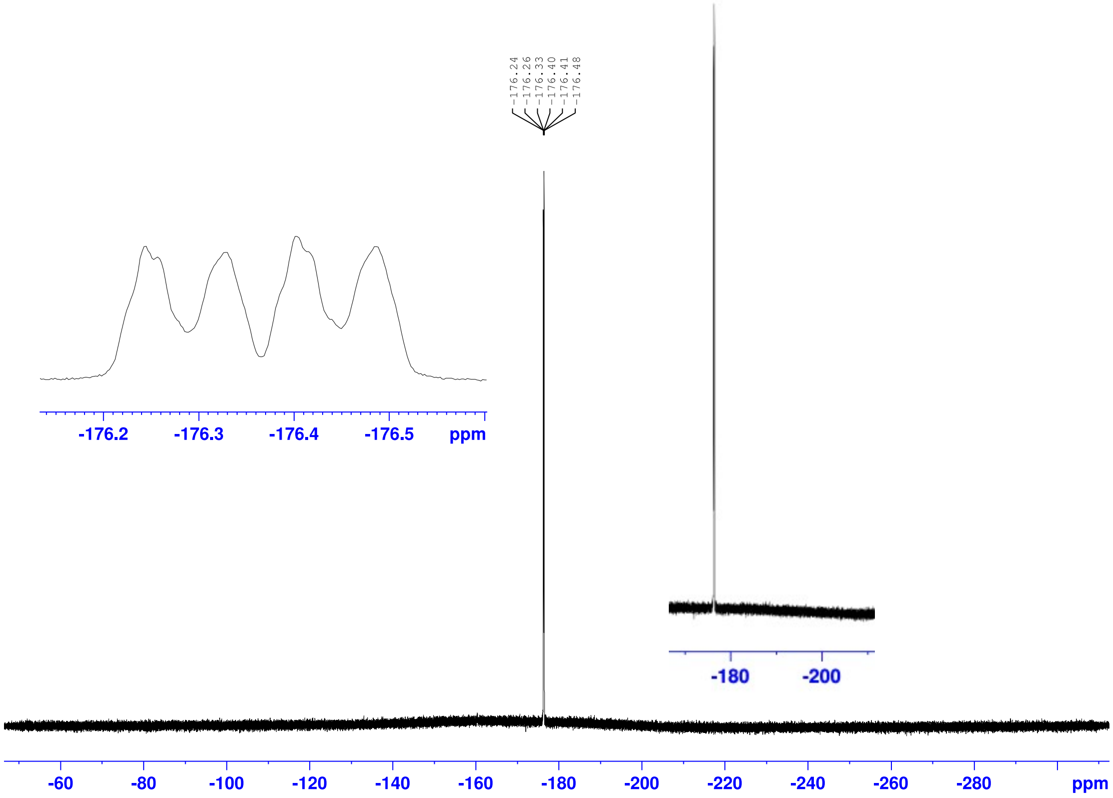

Fig. S53. ^{19}F NMR spectrum ($CDCl_3$, 282 MHz) of compound **18**.

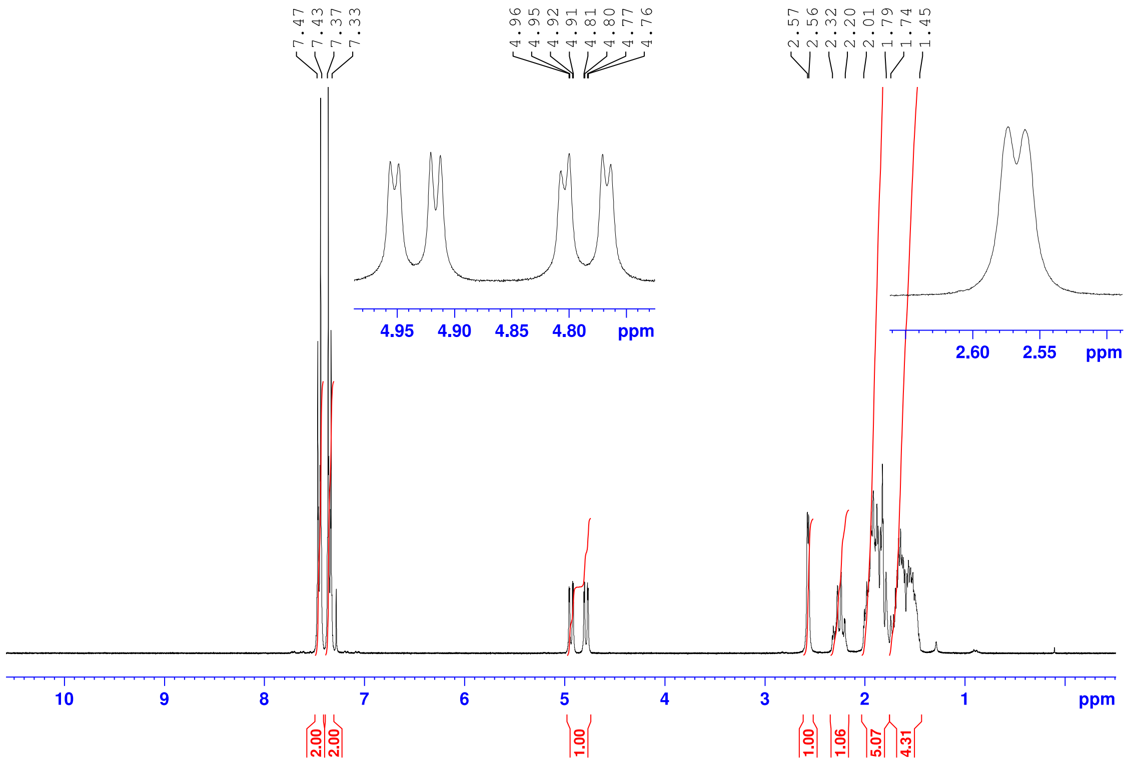

Fig. S54. ^{1}H NMR spectrum ($CDCl_3$, 400 MHz) of compound **18**.

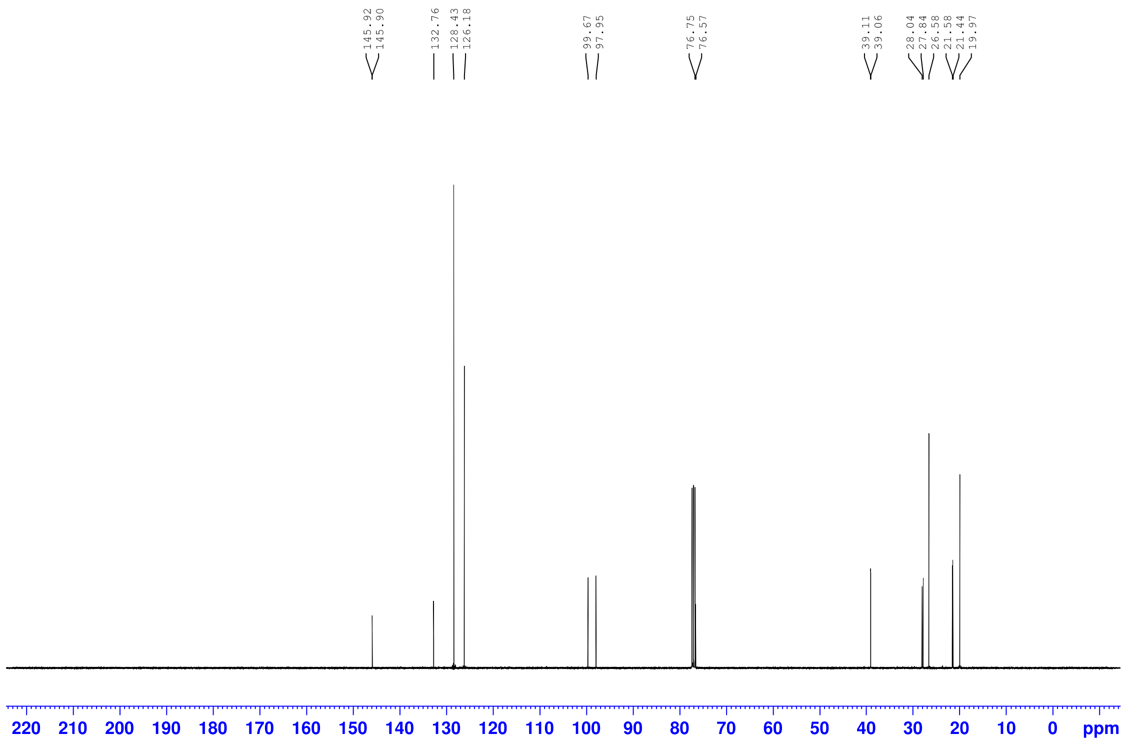

Fig. S55. $^{13}C\{^{1}H\}$ NMR spectrum ($CDCl_3$, 100 MHz) of compound **18**.

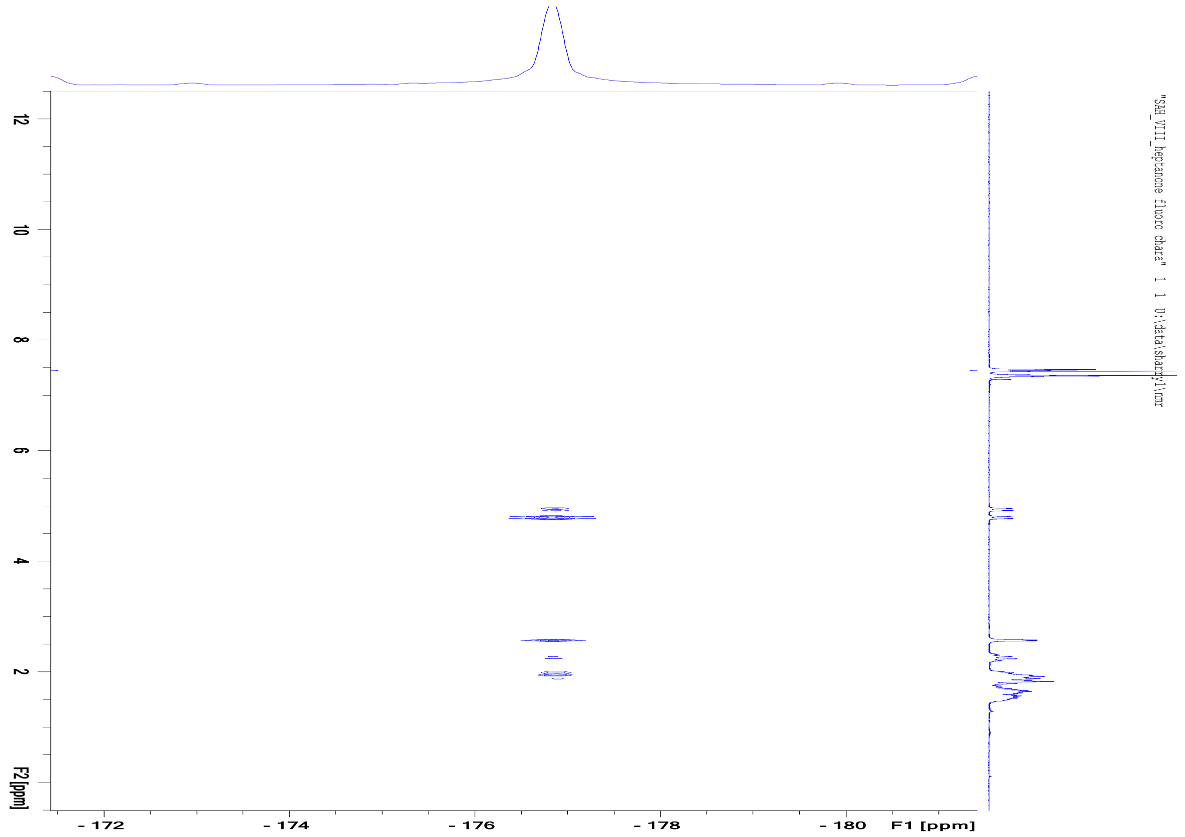

Fig. S56. HOESY ($CDCl_3$, 300 MHz) of compound **18**.

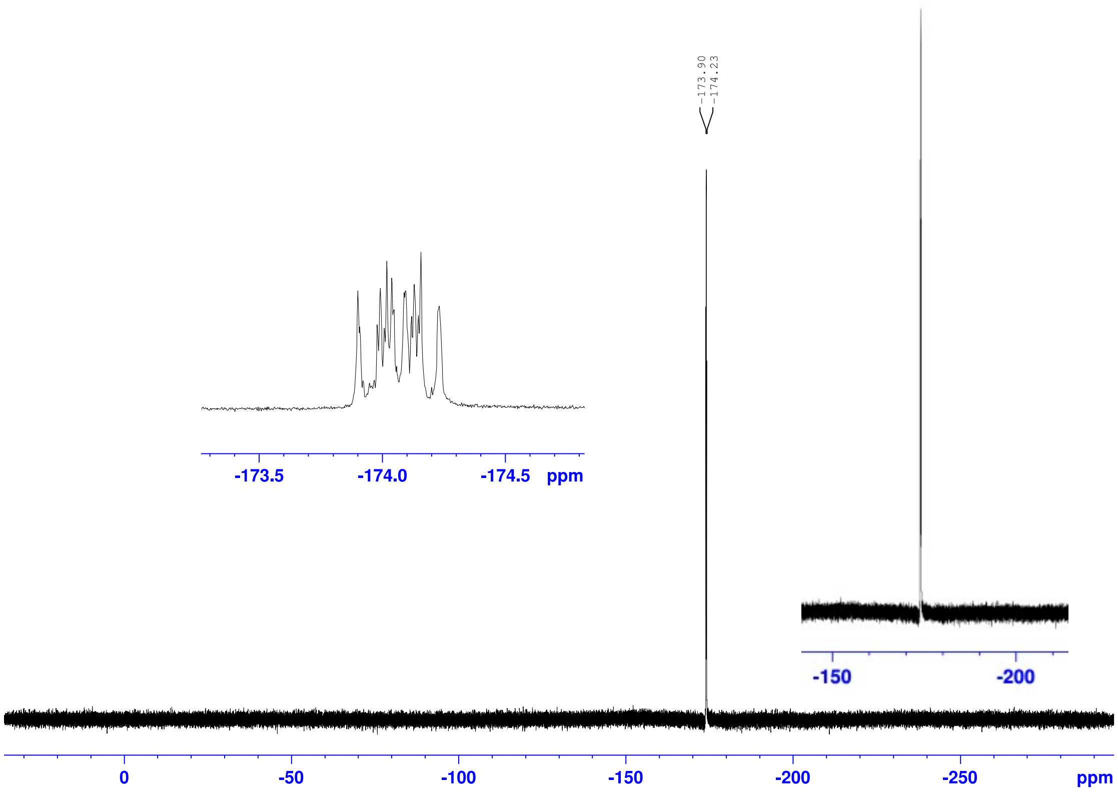

Fig. S57. ^{19}F NMR spectrum ($CDCl_3$, 282 MHz) of compound **19**.

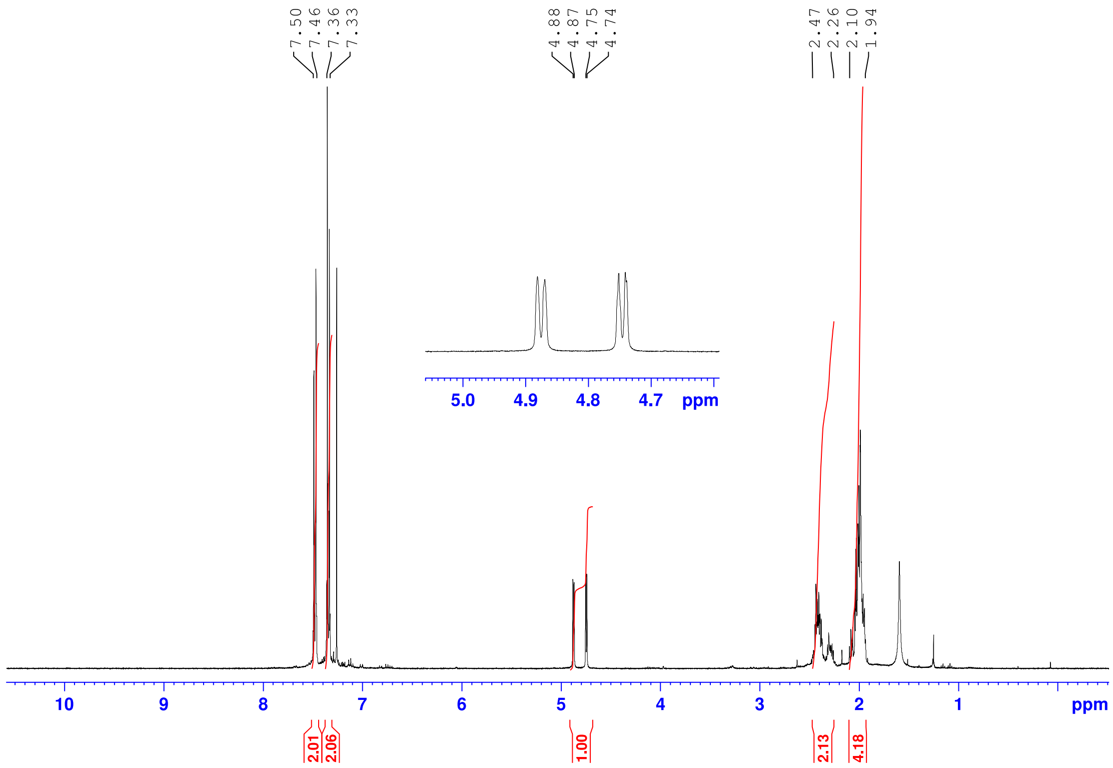

Fig. S58. ^{1}H NMR spectrum ($CDCl_3$, 400 MHz) of compound **19**.

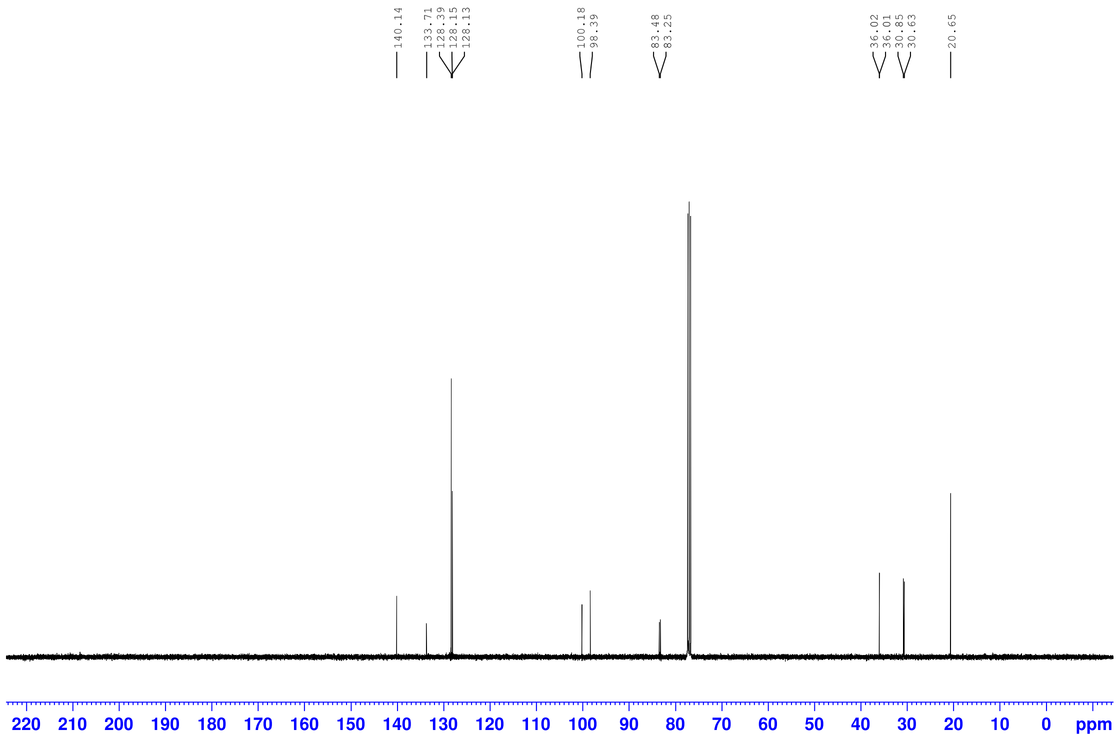

Fig. S59. $^{13}C\{^{1}H\}$ NMR spectrum ($CDCl_3$, 100 MHz) of compound **19**.

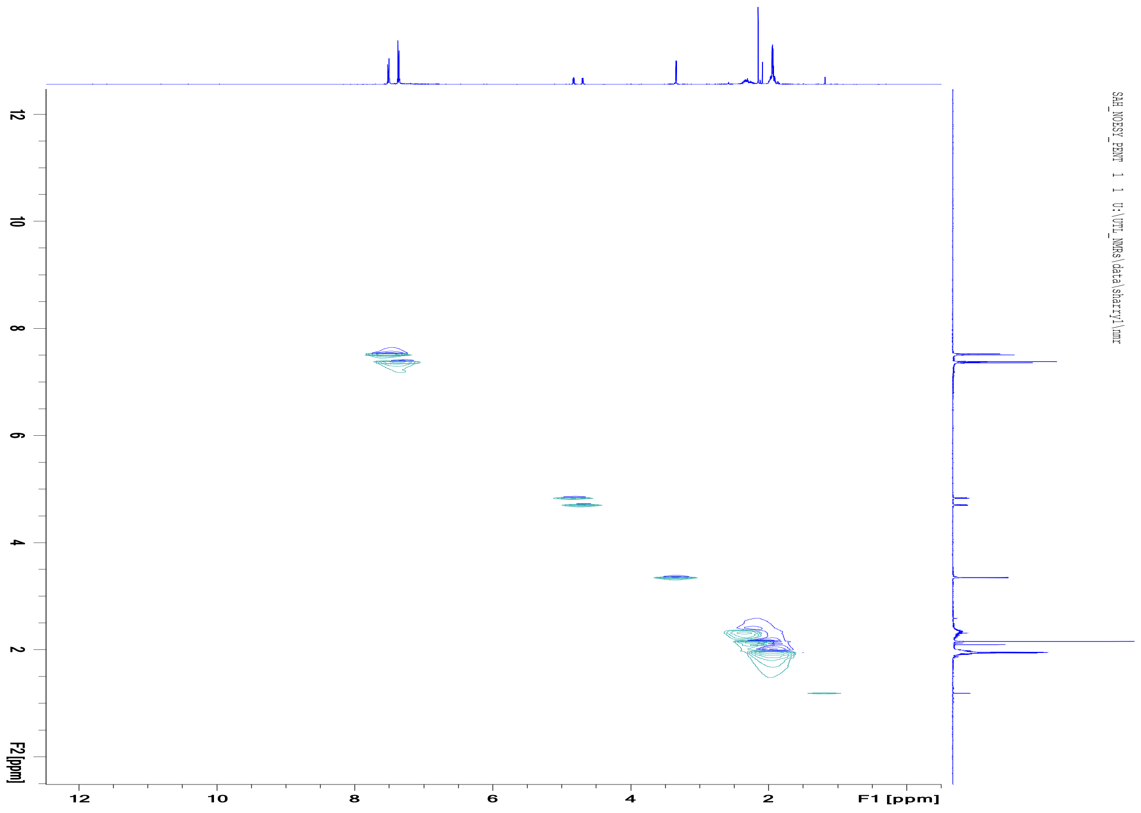

Fig. S60. NOESY ($CDCl_3$, 300 MHz) of compound **19**.

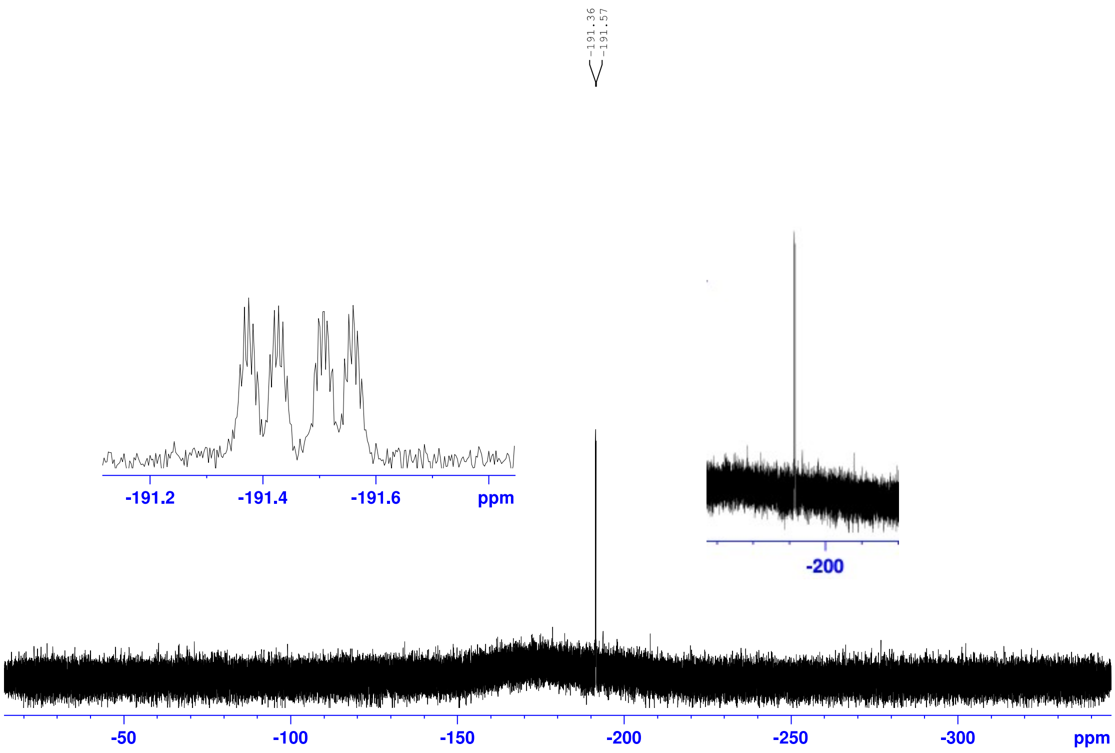

Fig. S61. ^{19}F NMR spectrum ($CDCl_3$, 282 MHz) of compound **20**.

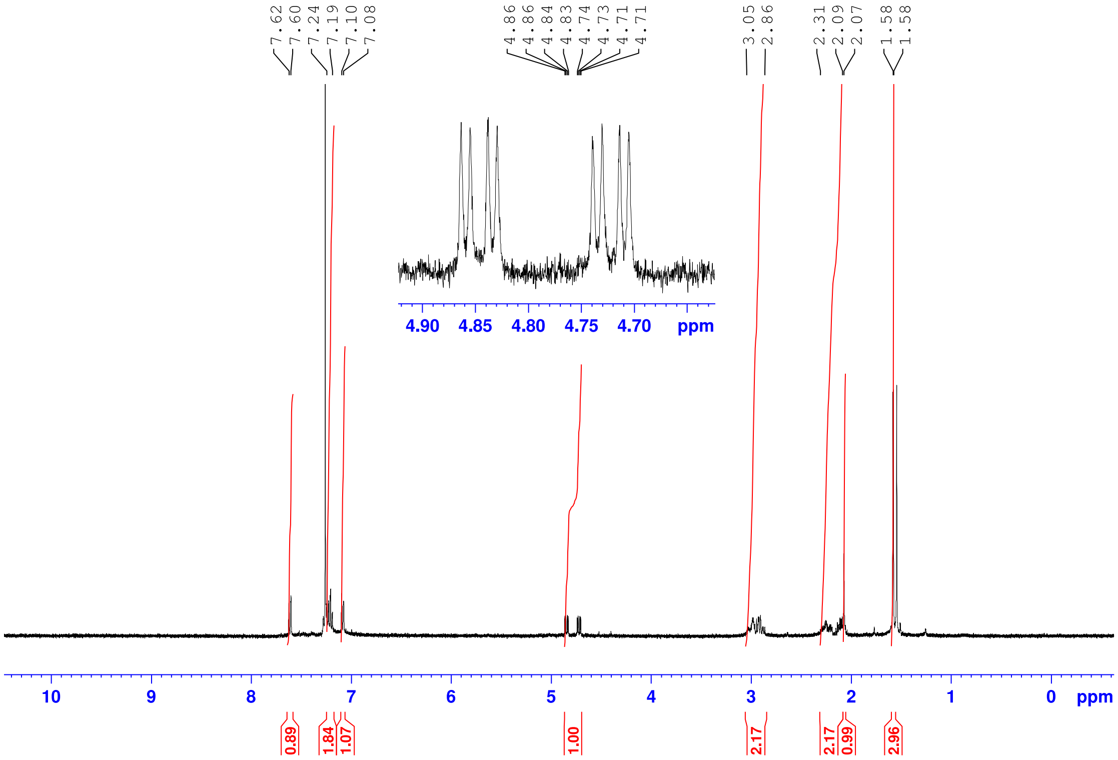

Fig. S62. ^{1}H NMR spectrum ($CDCl_3$, 400 MHz) of compound **20**.

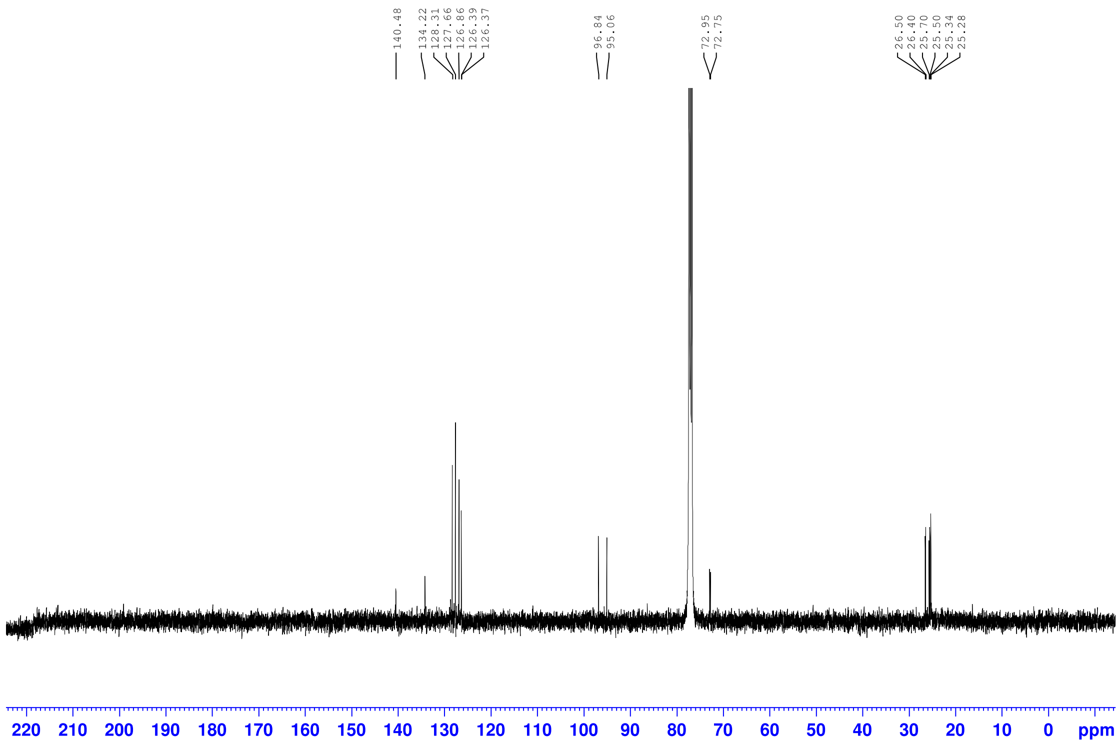

Fig. S63. $^{13}C\{^{1}H\}$ NMR spectrum ($CDCl_3$, 100 MHz) of compound **20**.

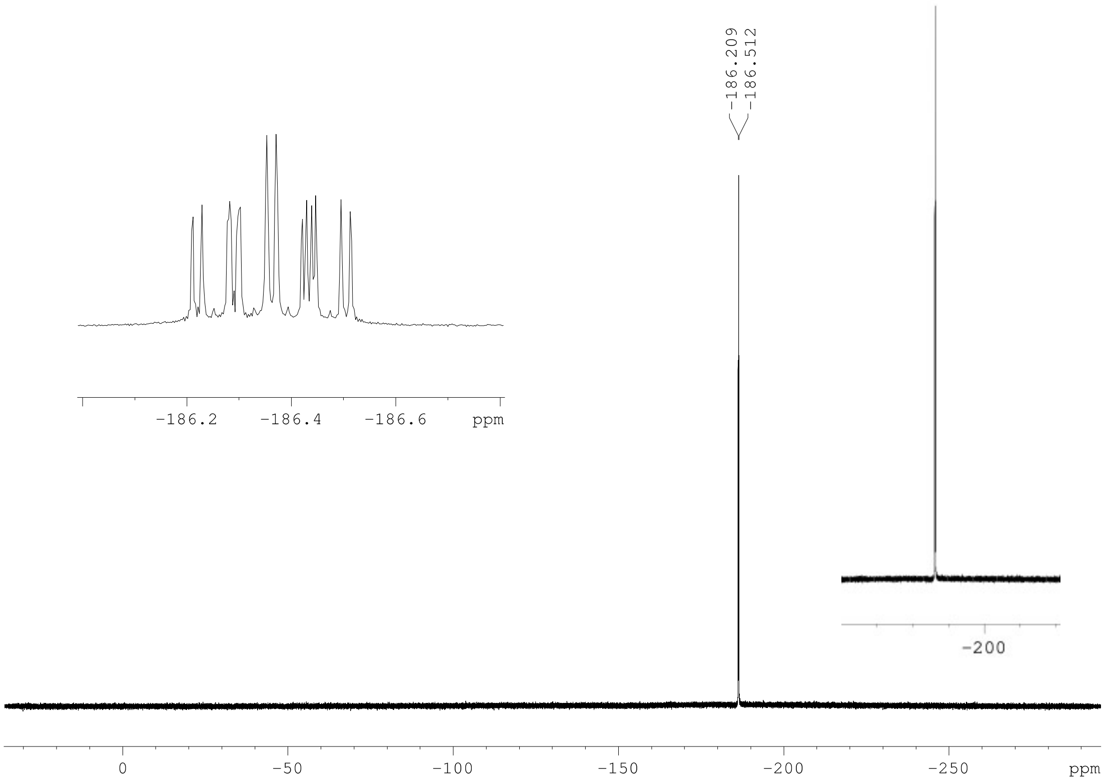

Fig. S64. ^{19}F NMR spectrum ($CDCl_3$, 282 MHz) of compound **21**.

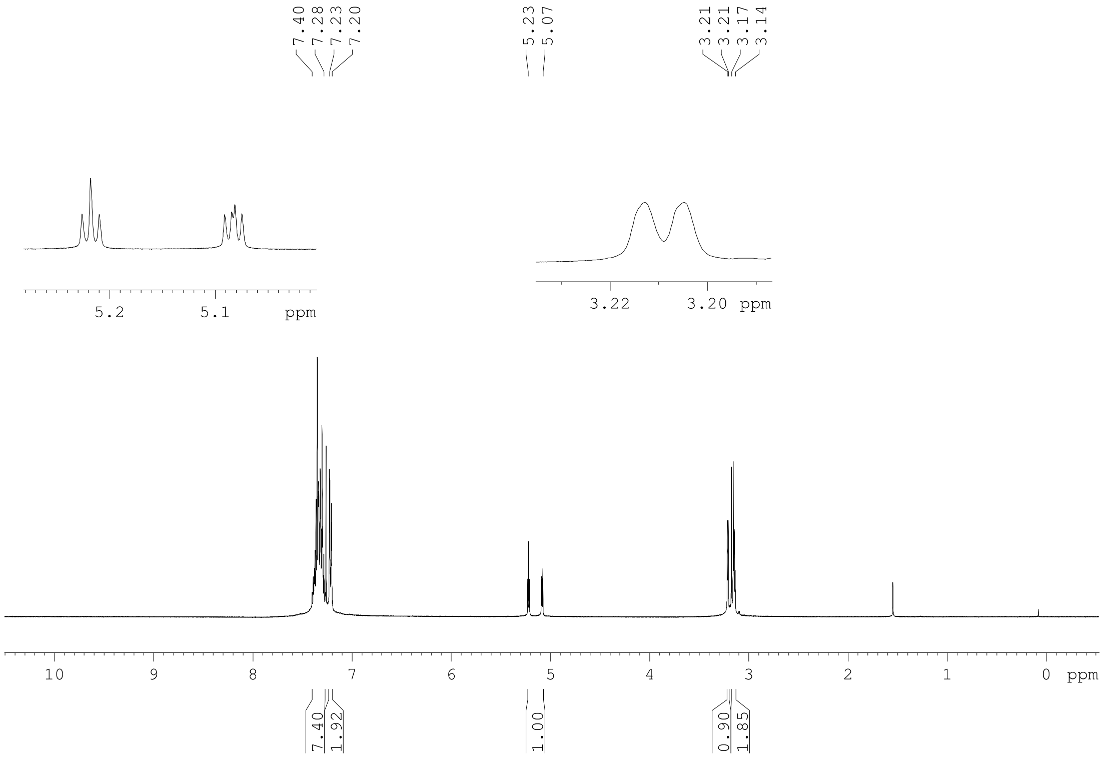

Fig. S65. ^{1}H NMR spectrum ($CDCl_3$, 400 MHz) of compound **21**.

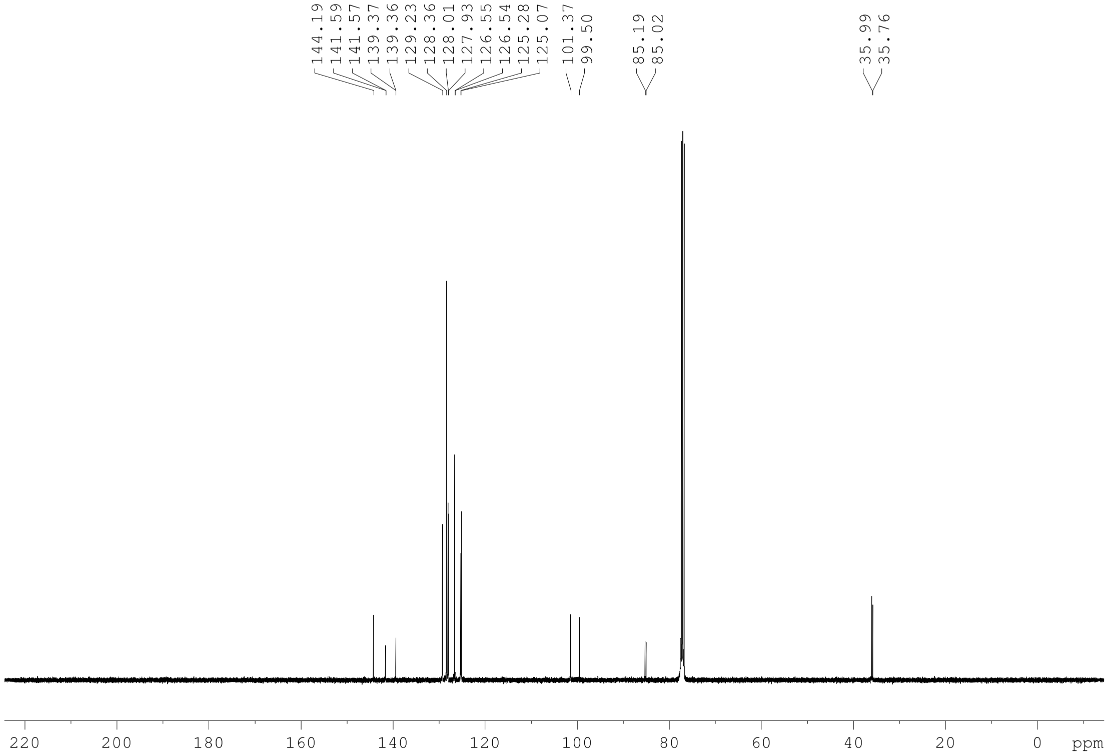

Fig. S66. $^{13}C\{^{1}H\}$ NMR spectrum ($CDCl_3$, 100 MHz) of compound **21**.

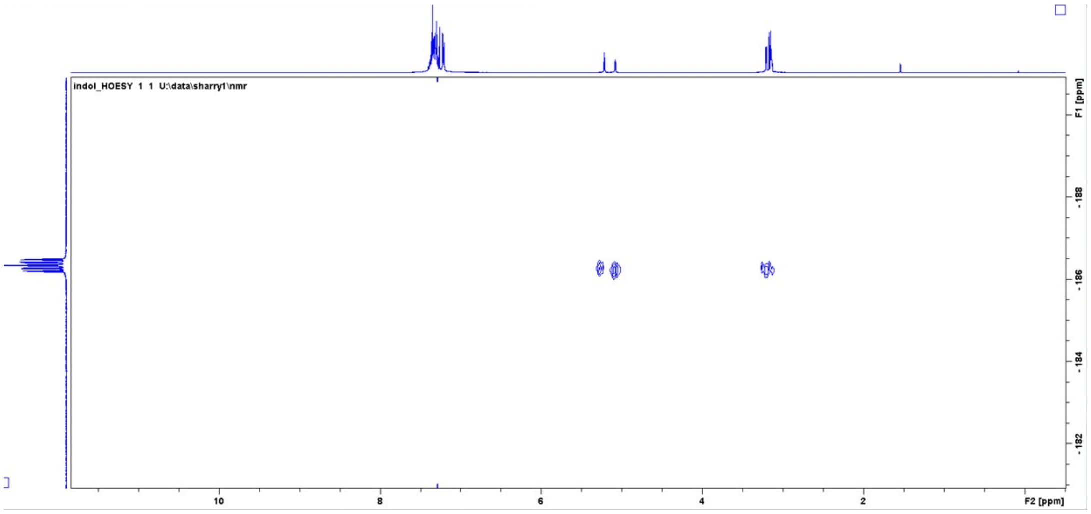

Fig. S67. ^{19}F-^{1}H HOESY ($CDCl_3$, 300 MHz) of compound **21**.

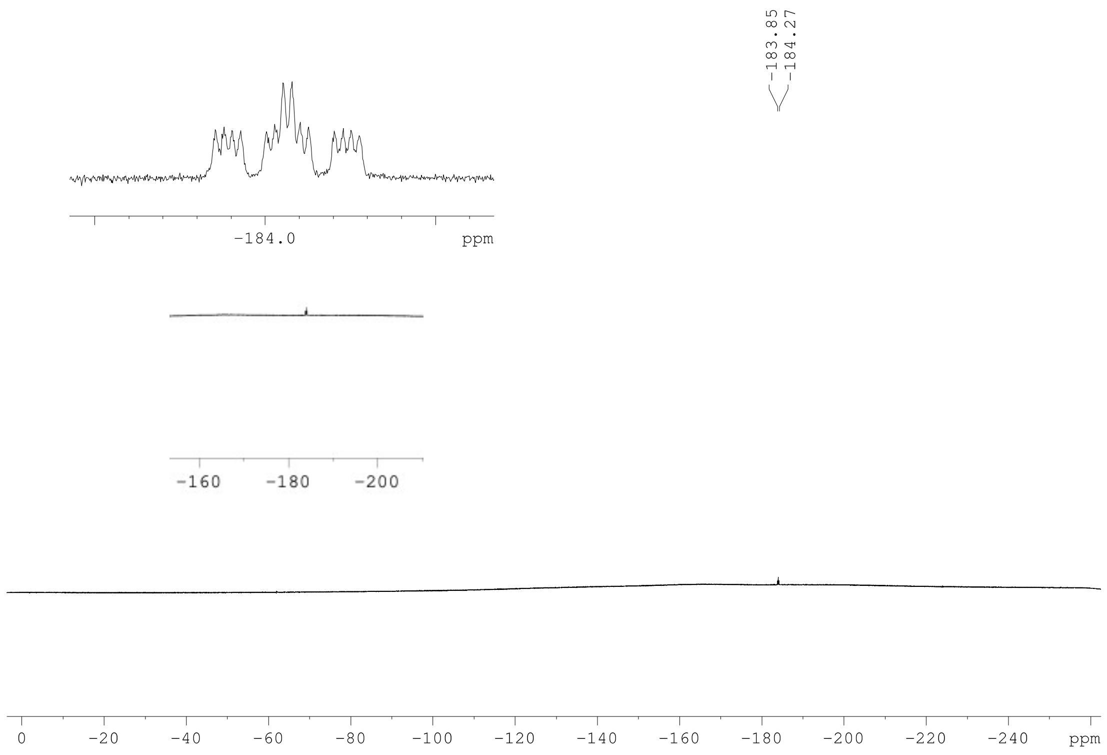

Fig. S68. ^{19}F NMR spectrum ($CDCl_3$, 282 MHz) of compound **22**.

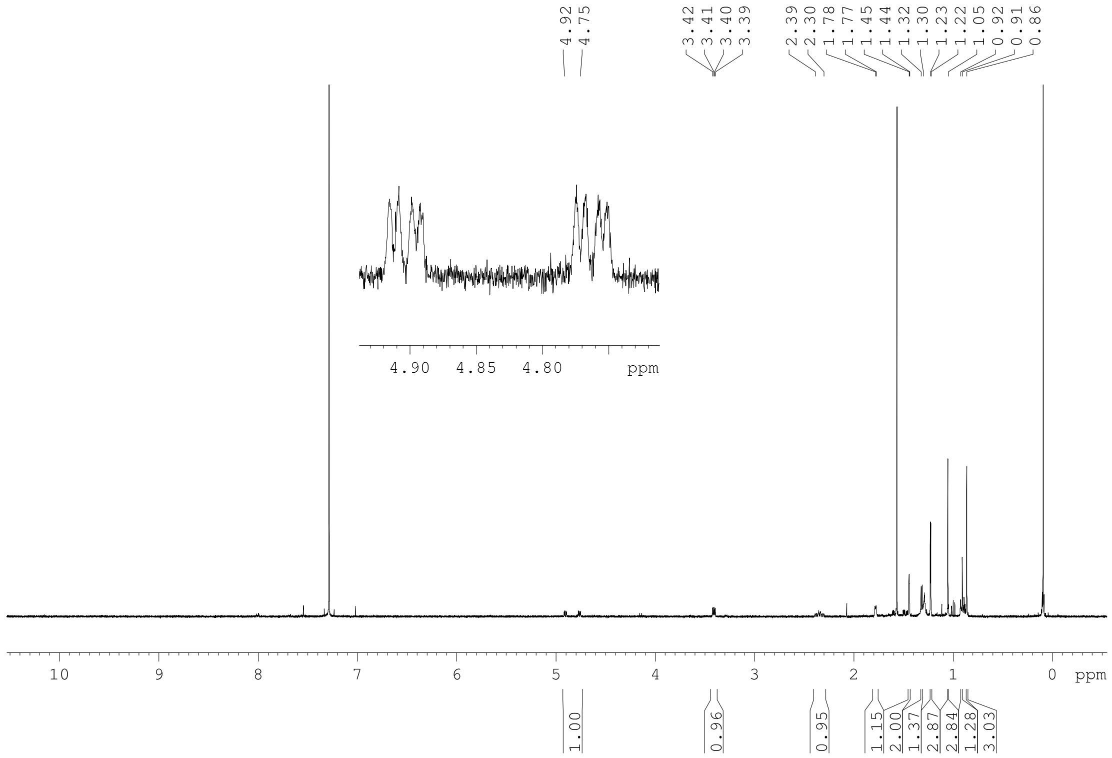

Fig. S69. ^{1}H NMR spectrum ($CDCl_3$, 400 MHz) of compound **22**.

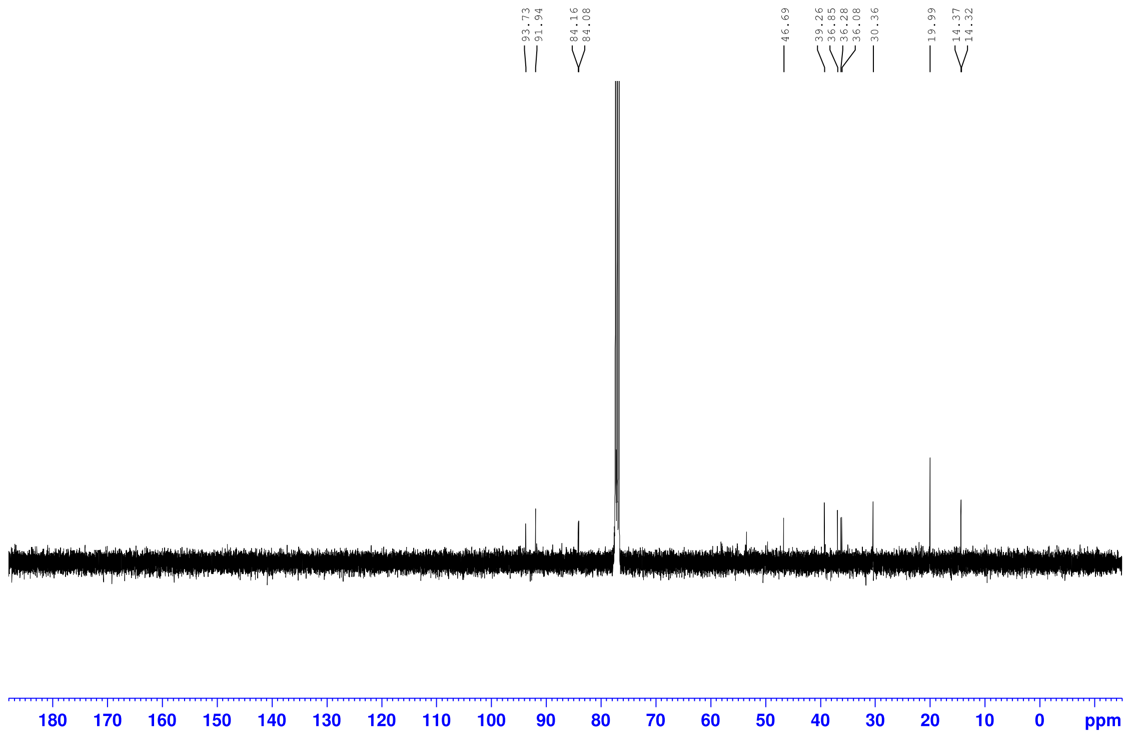

Fig. S70. $^{13}C\{^{1}H\}$ NMR spectrum ($CDCl_3$, 100 MHz) of compound **22**.

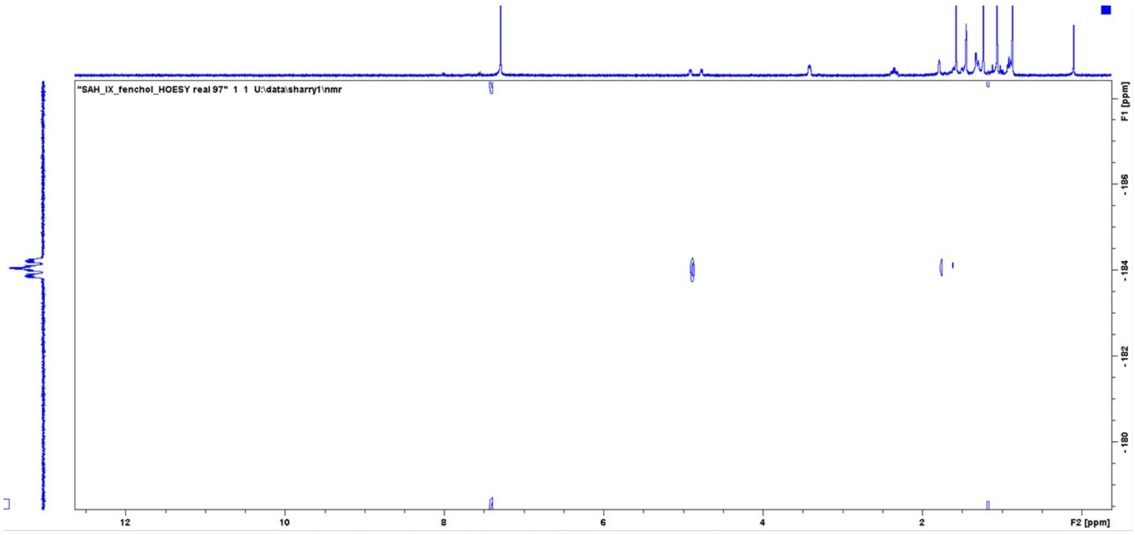

Fig. S71. ^{19}F-^{1}H HOESY ($CDCl_3$, 300 MHz) of compound **22**.

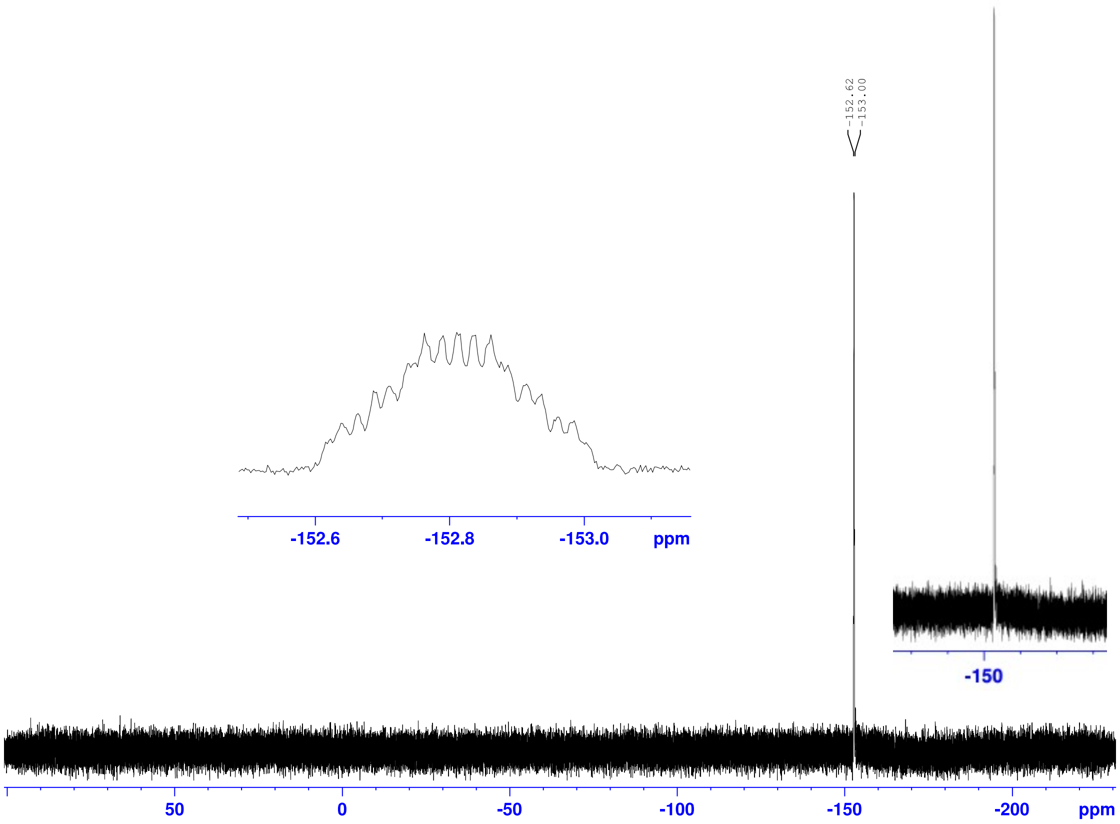

Fig. S72. ^{19}F NMR spectrum ($CDCl_3$, 282 MHz) of compound **23**.

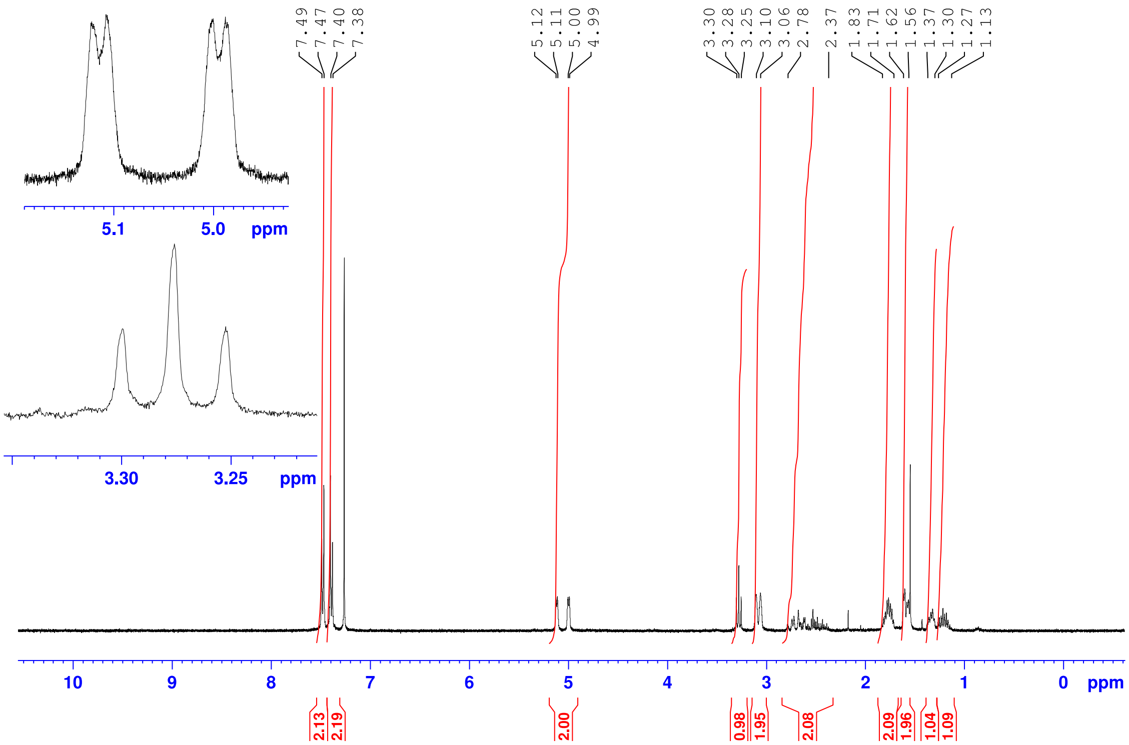

Fig. S73. ^{1}H NMR spectrum ($CDCl_3$, 400 MHz) of compound **23**.

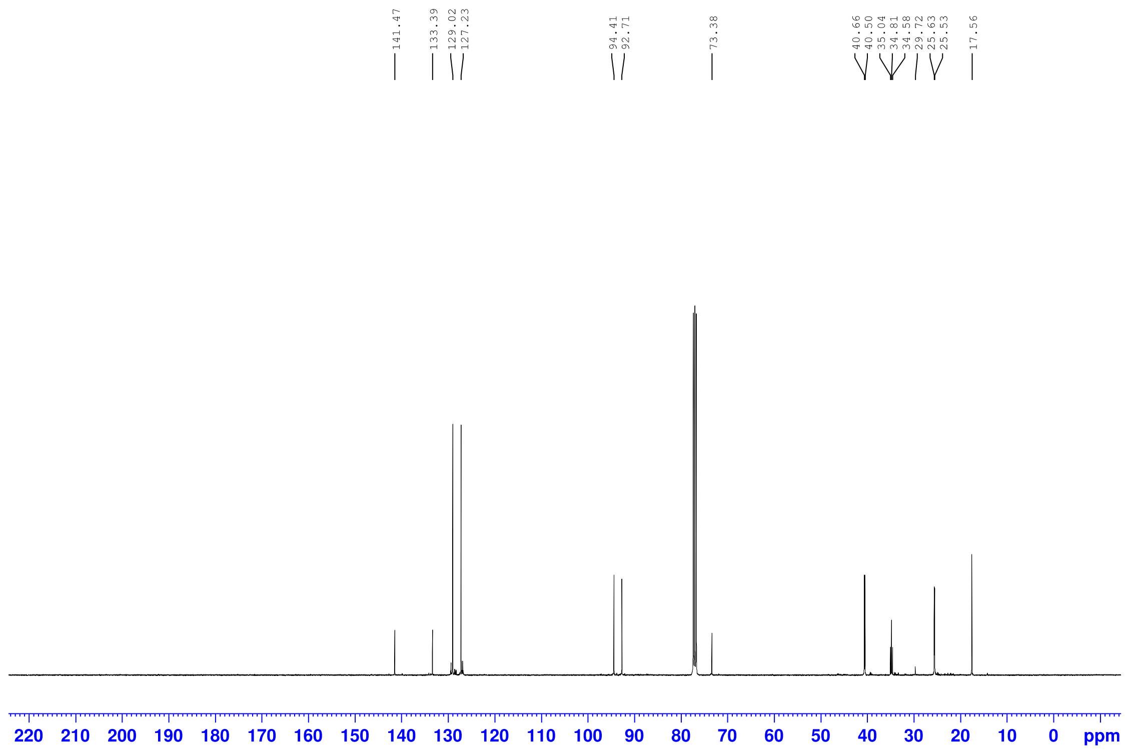

Fig. S74. $^{13}C\{^1H\}$ NMR spectrum ($CDCl_3$, 100 MHz) of compound **23**.

Computational Data

Table S1. Starting material for compound **6** geometry optimization (B3LYP/6-311++G**).

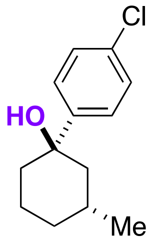

Center Number	Atomic Number	Forces (Hartrees/Bohr) X	Y	Z
1	6	-0.000009341	0.000000377	0.000012144
2	6	0.000000854	-0.000000990	-0.000007473
3	6	-0.000007527	-0.000009624	0.000010466
4	6	-0.000014961	-0.000017677	-0.000007326
5	6	0.000017283	-0.000009083	-0.000006966
6	6	-0.000008860	-0.000002709	-0.000002734
7	1	-0.000001510	-0.000005680	-0.000005619
8	1	-0.000004347	-0.000006844	0.000006262
9	1	-0.000004010	-0.000008615	0.000004479
10	1	-0.000006474	-0.000003711	-0.000000239
11	1	0.000003761	0.000006116	-0.000004803
12	1	0.000006084	0.000002111	-0.000008124
13	1	0.000004872	0.000003918	0.000001061
14	1	-0.000002289	-0.000001834	0.000006822
15	1	-0.000004100	-0.000006920	-0.000006956
16	6	-0.000010918	-0.000003133	-0.000000168
17	1	-0.000004020	-0.000000252	0.000009077
18	1	-0.000008054	-0.000001836	0.000009709
19	1	-0.000006429	-0.000001783	0.000007144
20	6	-0.000006470	0.000004663	0.000009046
21	6	-0.000001790	0.000010177	-0.000001368
22	6	0.000007896	0.000001423	0.000001021
23	6	0.000010579	0.000001835	-0.000007293
24	1	0.000003216	0.000004702	-0.000000211
25	6	-0.000002399	0.000012871	-0.000004477
26	1	0.000003377	0.000005353	-0.000004769
27	6	0.000014631	0.000002098	0.000001349
28	1	0.000007074	0.000008526	-0.000003306
29	1	0.000008550	0.000007838	-0.000001745
30	8	0.000005412	-0.000000231	-0.000000471
31	1	0.000000998	-0.000003436	-0.000001488

32	17	0.000008914	0.000012346	-0.000003044

Table S2. Starting material for compound **7** geometry optimization (B3LYP/6-311++G**).

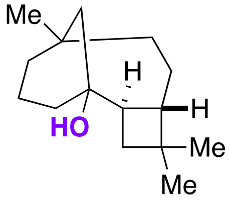

Center Number	Atomic Number	Forces (Hartrees/Bohr) X	Y	Z
1	6	0.000010683	0.000020438	0.000004752
2	1	0.000000998	-0.000011461	-0.000006563
3	6	-0.000007114	-0.000007830	-0.000017815
4	1	0.000012294	0.000001940	-0.000006995
5	6	-0.000008699	-0.000000038	0.000012674
6	1	0.000004839	-0.000001928	0.000001341
7	6	0.000006974	-0.000015725	-0.000008584
8	1	0.000000923	-0.000007320	-0.000004840
9	6	0.000002307	-0.000007369	0.000004029
10	1	-0.000001810	-0.000009747	0.000003303
11	1	-0.000003419	-0.000007043	0.000010201
12	1	0.000001025	-0.000010243	0.000004526
13	6	-0.000005180	0.000003840	0.000003192
14	1	0.000000585	0.000003146	0.000001738
15	1	-0.000001206	-0.000000649	0.000007647
16	1	0.000000851	0.000000764	0.000000534
17	6	0.000004510	0.000012685	0.000000782
18	6	-0.000007801	-0.000002875	0.000005841
19	1	-0.000001594	0.000001711	0.000007540
20	1	-0.000000858	0.000006243	0.000003008
21	6	0.000001984	0.000007384	0.000008517
22	1	-0.000006197	0.000002048	0.000006418
23	1	-0.000001996	-0.000005199	0.000004911
24	6	-0.000006919	-0.000004798	0.000007826
25	6	0.000005444	0.000000311	0.000007791
26	1	-0.000002239	0.000002999	0.000005812
27	1	0.000000540	0.000000027	-0.000002776
28	1	-0.000001158	-0.000005998	0.000001921
29	6	-0.000001920	0.000001041	-0.000002958
30	1	-0.000000468	0.000011643	0.000003795
31	1	0.000000367	0.000008030	-0.000002664

32	6	-0.000008582	0.000003000	0.000006510
33	1	-0.000001488	0.000008087	-0.000003758
34	1	-0.000008231	0.000011793	-0.000004266
35	6	0.000004375	0.000009366	-0.000015622
36	1	0.000004741	0.000002293	-0.000007582
37	1	0.000002766	0.000007253	-0.000006436
38	6	-0.000002410	-0.000005427	-0.000004856
39	1	0.000005337	-0.000002213	-0.000009433
40	1	0.000003366	-0.000002762	0.000001567
41	8	-0.000000303	-0.000019048	-0.000016443
42	1	0.000004683	0.000001632	-0.000004587

Table S3. Starting material for compound **8** geometry optimization (B3LYP/6-311++G**).

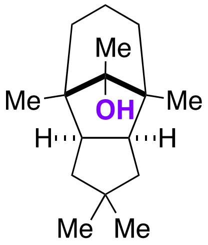

Center Number	Atomic Number	Forces (Hartrees/Bohr) X	Y	Z
1	6	0.000006709	0.000005101	0.000009759
2	6	0.000005703	-0.000004947	0.000010110
3	6	-0.000002891	-0.000010948	-0.000016994
4	6	-0.000003300	0.000011207	-0.000016986
5	6	-0.000002030	0.000003207	0.000010487
6	6	-0.000002120	-0.000002974	0.000010006
7	6	-0.000005953	-0.000000092	0.000001312
8	1	-0.000000772	-0.000001733	0.000005196
9	1	-0.000000706	0.000001346	0.000001091
10	1	-0.000000499	-0.000001128	0.000000613
11	1	-0.000000626	0.000001737	0.000004865
12	1	-0.000003210	0.000002851	0.000001301
13	1	-0.000003304	-0.000002913	0.000001163
14	1	0.000001291	-0.000000055	0.000003856
15	1	-0.000008918	-0.000000063	0.000003913
16	6	0.000005936	0.000000141	0.000003421
17	6	0.000002892	-0.000006023	-0.000002548
18	1	0.000000654	-0.000002354	0.000004355
19	1	-0.000001000	-0.000004134	-0.000000130
20	1	-0.000000306	0.000001074	0.000003414

21	6	0.000002957	0.000005926	-0.000002862
22	1	0.000000655	0.000002290	0.000004325
23	1	0.000000016	-0.000000997	0.000003448
24	1	-0.000000973	0.000004114	-0.000000111
25	6	0.000001563	-0.000000873	-0.000010428
26	1	-0.000006064	-0.000001056	0.000002532
27	1	0.000000859	-0.000004205	-0.000003444
28	6	0.000001730	0.000000217	-0.000010740
29	1	-0.000006039	0.000000959	0.000002408
30	1	0.000000632	0.000004257	-0.000003901
31	6	-0.000002192	0.000000010	-0.000009193
32	6	0.000001030	0.000000003	-0.000013631
33	1	0.000001633	0.000001474	-0.000001564
34	1	0.000001655	-0.000001463	-0.000001569
35	1	0.000004538	-0.000000036	0.000000584
36	6	0.000002026	0.000000084	0.000004154
37	1	-0.000000064	-0.000000669	-0.000004078
38	1	-0.000000033	0.000000651	-0.000004096
39	1	-0.000005378	0.000000043	-0.000004208
40	8	0.000012179	0.000000167	0.000016024
41	1	0.000002634	0.000000127	0.000001819
42	6	0.000002855	-0.000000248	-0.000006074
43	1	0.000002814	0.000007556	0.000002695
44	1	0.000002855	-0.000007453	0.000002708
45	1	-0.000009439	-0.000000180	-0.000003002

Table S4. Starting material for compound **9** geometry optimization (B3LYP/6-311++G**).

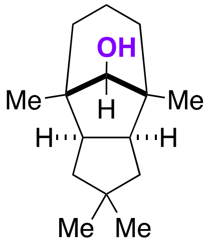

Center Number	Atomic Number	Forces (Hartrees/Bohr) X	Y	Z
1	6	0.000003550	-0.000025675	-0.000007088
2	6	0.000003377	0.000025600	-0.000006942
3	6	-0.000003112	-0.000013770	0.000009305
4	6	-0.000003313	0.000013324	0.000009312
5	6	0.000002192	-0.000007665	0.000003675
6	6	0.000002725	0.000007319	0.000003487

7	6	0.000019956	-0.000000089	0.000003998
8	1	-0.000004367	0.000003106	-0.000007465
9	1	-0.000003008	-0.000006243	0.000008566
10	1	-0.000003406	0.000006497	0.000008411
11	1	-0.000004339	-0.000003077	-0.000007478
12	1	-0.000000939	0.000004167	0.000003378
13	1	-0.000001100	-0.000004054	0.000003517
14	1	-0.000002765	0.000000005	0.000008422
15	1	0.000006593	0.000000045	-0.000008458
16	6	0.000024659	0.000000552	-0.000017521
17	1	-0.000010598	0.000000299	0.000013189
18	6	-0.000007938	0.000006565	-0.000003544
19	1	0.000000990	-0.000004920	0.000005217
20	1	-0.000000958	-0.000001109	0.000002538
21	1	0.000003148	-0.000000180	-0.000005155
22	6	-0.000008098	-0.000006603	-0.000003426
23	1	0.000001003	0.000005118	0.000005176
24	1	0.000003242	0.000000174	-0.000005150
25	1	-0.000000914	0.000001102	0.000002560
26	6	0.000016675	0.000008513	-0.000008099
27	1	-0.000007513	-0.000000540	-0.000001154
28	1	0.000000562	-0.000005448	0.000007326
29	6	0.000016716	-0.000008415	-0.000008145
30	1	-0.000007525	0.000000527	-0.000001142
31	1	0.000000548	0.000005422	0.000007335
32	6	-0.000028355	-0.000000035	-0.000007758
33	6	0.000000346	0.000000014	-0.000008699
34	1	0.000000348	-0.000000147	-0.000004437
35	1	0.000000344	0.000000152	-0.000004436
36	1	0.000004162	0.000000000	-0.000003282
37	6	0.000000049	0.000000008	-0.000000938
38	1	0.000000464	-0.000002259	-0.000002437
39	1	0.000000469	0.000002250	-0.000002440
40	1	-0.000009225	-0.000000006	-0.000001279
41	8	-0.000008597	-0.000001638	0.000015790
42	1	0.000003954	0.000001114	0.000005273

Table S5. Starting material for compound **10** geometry optimization (B3LYP/6-311++G**).

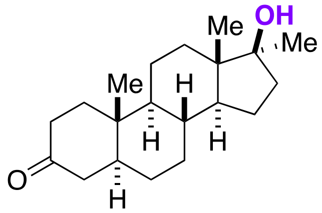

Center Number	Atomic Number	Atomic Type	Coordinates (Angstroms) X	Y	Z
1	6	0	1.936887	-0.389950	0.314988
2	6	0	2.706365	-1.558553	-0.352904
3	6	0	4.232623	-1.521053	-0.133180
4	1	0	2.331393	-2.518486	0.010116
5	1	0	4.460486	-1.724203	0.921050
6	1	0	4.738113	-2.288043	-0.723156
7	1	0	2.508076	-1.535514	-1.431496
8	6	0	0.437784	-0.405359	-0.151776
9	1	0	0.473715	-0.346936	-1.251411
10	6	0	-0.348701	0.856852	0.311673
11	1	0	-0.391315	0.869149	1.407495
12	6	0	-0.317081	-1.710259	0.193101
13	1	0	0.198710	-2.562690	-0.256934
14	1	0	-0.296003	-1.881161	1.272891
15	6	0	-1.783133	0.778566	-0.227147
16	1	0	-1.684625	0.718852	-1.320792
17	6	0	-2.551276	-0.492801	0.211941
18	6	0	-1.779269	-1.719838	-0.298448
19	1	0	-1.773562	-1.724251	-1.393428
20	6	0	2.042702	-0.517664	1.851005
21	1	0	3.073101	-0.428138	2.200370
22	1	0	1.461655	0.244269	2.372523
23	1	0	1.678640	-1.492462	2.183553
24	6	0	-2.714633	-0.583743	1.747530
25	1	0	-3.272160	0.260701	2.156056
26	1	0	-3.268226	-1.486891	2.006835
27	1	0	-1.750969	-0.618110	2.256499
28	6	0	4.844423	-0.173390	-0.469609
29	6	0	4.089699	1.029705	0.065172
30	1	0	4.513221	1.929086	-0.387688
31	1	0	4.275272	1.093935	1.145263
32	8	0	5.861374	-0.065721	-1.122690
33	6	0	0.356351	2.144974	-0.141600
34	1	0	0.292980	2.218693	-1.236308
35	1	0	-0.168388	3.018586	0.257593
36	6	0	1.829474	2.184721	0.274619
37	1	0	1.912279	2.272641	1.364542
38	1	0	2.310015	3.077637	-0.140356
39	6	0	2.574423	0.935665	-0.208603
40	1	0	2.459694	0.906629	-1.302621
41	6	0	-2.761729	1.931084	0.068105
42	1	0	-2.658239	2.270172	1.103246

43	1	0	-2.582748	2.799907	-0.568849
44	1	0	-2.269512	-2.646380	0.019115
45	6	0	-4.166300	1.308997	-0.169126
46	1	0	-4.810973	1.440005	0.702075
47	1	0	-4.676893	1.773051	-1.017667
48	6	0	-3.961547	-0.223100	-0.415835
49	8	0	-4.934803	-1.016946	0.286246
50	1	0	-5.783447	-0.921714	-0.158341
51	6	0	-4.065083	-0.562269	-1.908540
52	1	0	-3.941870	-1.633916	-2.074751
53	1	0	-5.054435	-0.271878	-2.280248
54	1	0	-3.328437	-0.026714	-2.511833

Table S6. Starting material for compound **12** geometry optimization (B3LYP/6-311++G**).

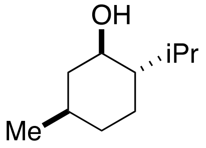

Center Number	Atomic Number	Forces (Hartrees/Bohr) X	Y	Z
1	6	0.000004327	0.000005308	0.000007415
2	6	0.000000291	-0.000004518	0.000007822
3	6	0.000001374	-0.000007737	0.000006029
4	6	0.000006992	0.000012907	-0.000003059
5	6	-0.000009071	0.000015155	0.000014155
6	6	0.000000500	-0.000005745	-0.000002070
7	1	-0.000000312	-0.000002317	0.000002563
8	1	0.000000307	0.000001182	0.000011441
9	1	0.000003103	-0.000008102	0.000012764
10	1	0.000002525	-0.000009550	0.000004835
11	1	-0.000002984	0.000003723	0.000002261
12	1	-0.000003023	0.000008262	0.000005329
13	1	-0.000000928	0.000003646	-0.000000082
14	1	0.000001605	-0.000007362	0.000000140
15	6	-0.000003072	-0.000008796	-0.000002542
16	1	-0.000002735	0.000008168	-0.000008280
17	6	0.000001651	0.000002014	-0.000017398
18	1	-0.000000671	-0.000006084	-0.000011290
19	1	0.000000502	-0.000002681	-0.000016532
20	1	0.000002503	-0.000008688	-0.000010642
21	6	0.000002781	0.000006150	-0.000004742

22	1	-0.000004005	0.000005171	-0.000009104
23	1	-0.000003297	0.000006896	-0.000002276
24	1	0.000000116	-0.000001681	-0.000003820
25	6	-0.000000399	-0.000008807	0.000014637
26	1	0.000000681	-0.000001334	0.000011459
27	1	0.000003855	-0.000006336	0.000014830
28	1	0.000001197	0.000002187	0.000016619
29	1	-0.000001614	-0.000000382	-0.000010778
30	8	0.000000390	0.000001002	-0.000020801
31	1	-0.000002588	0.000008348	-0.000008885

Table S7. Starting material for compound **13** geometry optimization (B3LYP/6-311++G**).

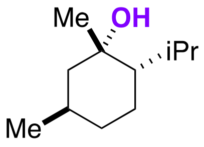

Center Number	Atomic Number	Forces (Hartrees/Bohr) X	Y	Z
1	6	0.000011992	0.000012963	-0.000001122
2	6	-0.000001525	-0.000003847	-0.000001569
3	6	0.000004784	-0.000007121	0.000017894
4	6	0.000006770	0.000023229	-0.000014700
5	6	-0.000007417	-0.000026299	0.000021266
6	6	0.000012887	0.000023159	-0.000003637
7	1	-0.000002423	0.000000167	-0.000000838
8	1	-0.000000198	0.000001491	0.000005895
9	1	0.000006193	-0.000003918	0.000012502
10	1	0.000002961	-0.000011317	0.000005542
11	1	-0.000005498	0.000002719	0.000007325
12	1	-0.000009404	-0.000000045	0.000000437
13	1	-0.000001150	0.000002342	-0.000004005
14	1	0.000003933	-0.000003210	0.000002465
15	6	0.000001337	0.000007508	-0.000002845
16	1	-0.000004879	0.000005965	-0.000008418
17	1	-0.000009455	0.000002354	-0.000005547
18	1	-0.000004819	0.000004461	-0.000001701
19	6	0.000000421	-0.000008897	-0.000010678
20	1	-0.000004352	0.000005043	-0.000001116
21	6	0.000014814	-0.000005521	-0.000016891
22	1	-0.000004508	-0.000003122	-0.000001521
23	1	-0.000000239	0.000004579	-0.000004145
24	1	0.000003820	-0.000003397	0.000002776

25	6	-0.000000862	0.000001674	-0.000005226
26	1	-0.000004420	0.000006735	-0.000000333
27	1	-0.000004352	0.000002926	0.000003314
28	1	0.000004350	-0.000002565	0.000001100
29	6	-0.000002357	-0.000017456	0.000010515
30	1	-0.000000583	-0.000000623	0.000004708
31	1	0.000004923	-0.000004432	0.000005411
32	1	0.000003051	0.000001967	0.000010290
33	8	-0.000012685	-0.000012223	-0.000024884
34	1	-0.000001107	0.000004711	-0.000002264

Table S8. Starting material for compound **14** geometry optimization (B3LYP/6-311++G**).

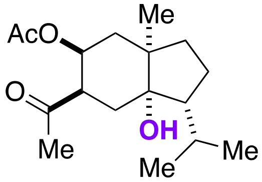

Center Number	Atomic Number	Forces (Hartrees/Bohr) X	Y	Z
1	6	0.000004895	0.000001375	-0.000000843
2	6	-0.000002462	-0.000000015	0.000001846
3	6	0.000002234	0.000002055	0.000000218
4	6	-0.000007764	-0.000003800	-0.000001398
5	6	0.000002140	0.000003595	-0.000005180
6	6	0.000000300	-0.000002166	0.000005644
7	1	0.000000645	-0.000000196	0.000001042
8	1	0.000003091	-0.000002414	0.000000096
9	1	0.000000615	0.000000300	-0.000002663
10	1	-0.000003357	0.000002534	-0.000002593
11	1	0.000000317	0.000001775	-0.000004036
12	1	-0.000001121	0.000001982	-0.000004807
13	6	-0.000001584	-0.000005286	0.000004607
14	1	-0.000002903	0.000002703	0.000000358
15	1	-0.000002932	0.000002458	0.000002321
16	6	0.000000287	-0.000002796	-0.000007356
17	1	-0.000000454	-0.000002625	0.000002474
18	6	0.000000414	0.000002835	-0.000003406
19	1	-0.000002506	-0.000000452	0.000003620
20	1	-0.000002411	0.000002873	0.000003267
21	6	0.000000656	0.000010785	0.000006527
22	1	-0.000000569	0.000000180	-0.000003574

23	1	-0.000000594	0.000000050	-0.000003489
24	1	-0.000006168	-0.000001531	0.000002741
25	8	0.000002170	-0.000003895	0.000000273
26	1	0.000002373	-0.000001348	-0.000000624
27	6	0.000000045	-0.000002252	0.000010980
28	1	0.000009682	-0.000002021	0.000001264
29	6	0.000004166	0.000002888	0.000001901
30	1	0.000001765	-0.000003284	0.000004255
31	1	-0.000000116	-0.000002772	0.000001549
32	1	-0.000000408	-0.000002903	0.000004679
33	6	0.000006362	-0.000010311	-0.000006519
34	1	0.000006165	-0.000004096	0.000003117
35	1	0.000004124	-0.000002486	0.000005907
36	1	0.000004302	0.000002221	0.000006160
37	6	-0.000003127	-0.000003422	0.000001110
38	8	-0.000006824	-0.000000210	-0.000011050
39	6	-0.000003283	0.000005554	0.000005307
40	8	-0.000004403	0.000004547	0.000001132
41	6	-0.000004887	-0.000004119	-0.000012951
42	1	-0.000002855	0.000005570	-0.000001158
43	1	-0.000003932	0.000004042	-0.000000640
44	1	-0.000005950	0.000004656	-0.000001778
45	8	0.000007003	0.000003671	-0.000006644
46	6	-0.000003507	0.000001842	-0.000003995
47	1	0.000002617	-0.000000765	0.000000149
48	1	0.000004654	-0.000003108	-0.000000090
49	1	0.000003094	-0.000002218	0.000002250

Table S9. Compound **8** geometry optimization (B3LYP/6-311++G**) shows intramolecular hydrogen bonding.

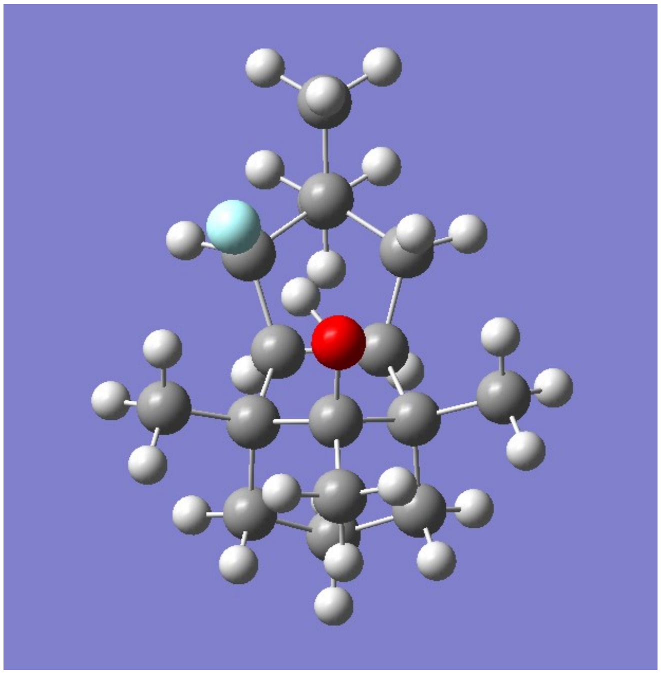

Center Number	Atomic Number	Atomic Type	Coordinates (Angstroms) X	Y	Z
1	6	0	-0.276126	-1.075286	0.307629
2	6	0	-0.357963	0.380759	0.888756
3	6	0	0.995542	1.075427	0.498373
4	6	0	1.117563	-1.180387	-0.392191
5	6	0	2.018846	0.740730	1.622135
6	6	0	2.146910	-1.636559	0.675882
7	6	0	2.131071	-0.760023	1.939931
8	1	0	-0.303455	-1.804368	1.122043
9	1	0	2.994504	1.139084	1.329687
10	1	0	3.145757	-1.644308	0.230807
11	1	0	-0.414325	0.358423	1.981091
12	1	0	1.729910	1.286629	2.527725
13	1	0	1.931613	-2.675976	0.949685
14	1	0	3.027696	-0.946388	2.539526
15	1	0	1.288799	-1.057611	2.572979
16	6	0	1.427689	0.305770	-0.800123
17	6	0	1.148071	-2.155576	-1.571719
18	1	0	2.166476	-2.273306	-1.954081
19	1	0	0.800834	-3.146571	-1.261553
20	1	0	0.523652	-1.810185	-2.395395

21	6	0	0.939832	2.603034	0.355612
22	1	0	1.948688	3.009506	0.242875
23	1	0	0.348003	2.934008	-0.495246
24	1	0	0.509584	3.052271	1.256723
25	6	0	-1.568966	-1.250265	-0.520469
26	1	0	-1.920054	-2.285689	-0.536433
27	1	0	-1.416979	-0.942803	-1.557099
28	6	0	-1.726742	0.934837	0.441199
29	1	0	-2.172876	1.625365	1.159368
30	6	0	-2.600126	-0.302687	0.144369
31	6	0	-3.795825	0.011807	-0.763640
32	1	0	-4.359973	-0.903441	-0.965877
33	1	0	-4.478745	0.724752	-0.290598
34	1	0	-3.475765	0.432756	-1.717229
35	6	0	-3.120910	-0.883731	1.475875
36	1	0	-3.786306	-0.173978	1.977182
37	1	0	-3.694136	-1.794805	1.282869
38	1	0	-2.319885	-1.141085	2.172601
39	8	0	0.633277	0.674820	-1.940166
40	1	0	-0.182370	1.108174	-1.663199
41	6	0	2.860364	0.569000	-1.274140
42	1	0	3.028447	0.029847	-2.209204
43	1	0	2.981700	1.632600	-1.490917
44	1	0	3.630061	0.270524	-0.564452
45	9	0	-1.625827	1.704102	-0.765270

Table S10. Compound **9** geometry optimization (B3LYP/6-311++G**) shows intramolecular hydrogen bonding.

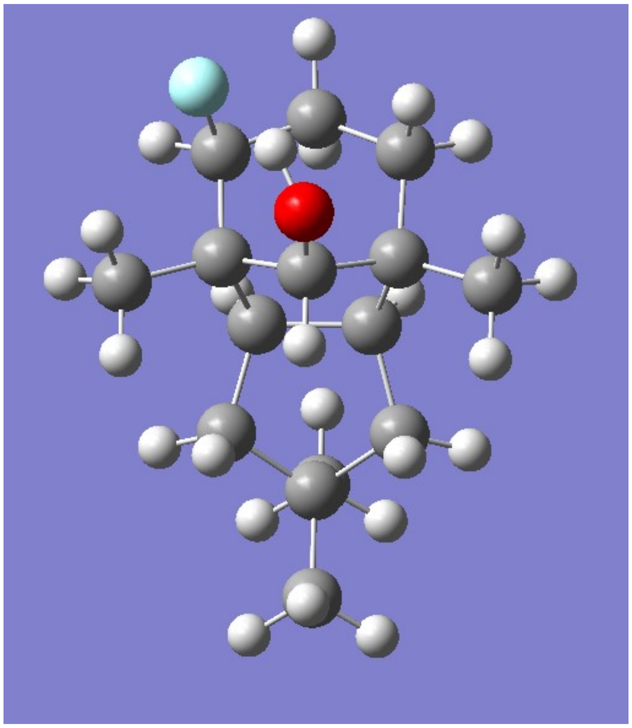

Center Number	Atomic Number	Atomic Type	Coordinates (Angstroms) X	Y	Z
1	6	0	-0.485260	-0.769434	0.495654
2	6	0	-0.639707	0.803363	0.575741
3	6	0	0.648879	1.389535	-0.080360
4	6	0	0.884741	-1.028947	-0.210126
5	6	0	1.771556	1.501077	0.977780
6	6	0	1.965687	-1.033643	0.891322
7	6	0	1.971658	0.204395	1.775797
8	1	0	-0.468376	-1.209019	1.497378
9	1	0	2.701756	1.775605	0.470189
10	1	0	-0.695612	1.131475	1.617358
11	1	0	1.542819	2.321293	1.666338
12	1	0	1.884669	-1.945303	1.490184
13	1	0	2.909907	0.225557	2.337155
14	1	0	1.175551	0.090144	2.517573
15	6	0	1.017029	0.254672	-1.074192
16	1	0	0.243650	0.217354	-1.849957
17	6	0	0.973673	-2.326329	-1.018916
18	1	0	1.966594	-2.439083	-1.457758
19	1	0	0.784520	-3.199305	-0.385535

20	1	0	0.250290	-2.338671	-1.835899
21	6	0	0.481366	2.743152	-0.773188
22	1	0	1.428879	3.048945	-1.224289
23	1	0	-0.263238	2.710235	-1.571680
24	1	0	0.178713	3.517759	-0.061020
25	6	0	-1.754643	-1.264092	-0.233554
26	1	0	-2.064524	-2.262180	0.089589
27	1	0	-1.584228	-1.316373	-1.313118
28	6	0	-1.992214	1.102717	-0.109255
29	1	0	-2.491330	1.980128	0.312345
30	1	0	-1.849943	1.300487	-1.176138
31	6	0	-2.832944	-0.187636	0.033305
32	6	0	-3.977166	-0.249980	-0.985627
33	1	0	-4.521898	-1.196758	-0.911090
34	1	0	-4.697177	0.558130	-0.820893
35	1	0	-3.600674	-0.159430	-2.009239
36	6	0	-3.409818	-0.320463	1.455646
37	1	0	-4.118322	0.487705	1.662760
38	1	0	-3.944059	-1.268944	1.570398
39	1	0	-2.633052	-0.283179	2.223954
40	8	0	2.230959	0.427350	-1.788675
41	1	0	2.956838	0.065256	-1.267680
42	9	0	3.258172	-1.126771	0.268702

Table S11. Compound **13** geometry optimization (B3LYP/6-311++G**) shows intramolecular hydrogen bonding.

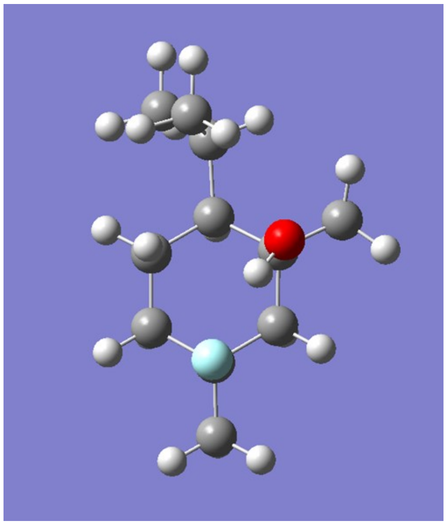

Center Number	Atomic Number	Atomic Type	Coordinates (Angstroms) X	Y	Z
1	6	0	2.084107	-0.354771	-0.076989
2	6	0	1.234710	-1.605653	-0.278073
3	6	0	-0.205341	-1.413449	0.208727
4	6	0	-0.885750	-0.200998	-0.453843
5	6	0	-0.068277	1.102345	-0.173830
6	6	0	1.395835	0.891935	-0.632009
7	1	0	-0.774017	-2.323664	0.000408
8	1	0	1.238705	-1.844554	-1.348503
9	1	0	1.713032	-2.443691	0.238233
10	1	0	-0.806089	-0.348335	-1.541548
11	1	0	1.416488	0.821000	-1.725091
12	1	0	1.979435	1.775756	-0.355780
13	1	0	-0.204388	-1.294831	1.295712
14	6	0	-0.639007	2.315641	-0.913125
15	1	0	-1.585261	2.626194	-0.468983
16	1	0	0.053385	3.157044	-0.831504
17	1	0	-0.802614	2.098267	-1.972325
18	6	0	-2.410668	-0.116340	-0.153112
19	1	0	-2.754609	0.857607	-0.515160
20	6	0	-2.781696	-0.203309	1.336614
21	1	0	-2.260251	0.552436	1.923715
22	1	0	-3.859183	-0.056291	1.460662
23	1	0	-2.540813	-1.186710	1.752549
24	6	0	-3.187526	-1.173465	-0.957900
25	1	0	-4.264428	-1.056653	-0.804628
26	1	0	-2.993016	-1.085594	-2.031459
27	1	0	-2.927180	-2.191721	-0.652450
28	6	0	3.512350	-0.509583	-0.570521
29	1	0	4.095357	0.385734	-0.343412
30	1	0	3.988864	-1.366815	-0.089387
31	1	0	3.526993	-0.667685	-1.651960
32	8	0	-0.096817	1.466222	1.217786
33	1	0	0.574473	0.955068	1.685471
34	9	0	2.185247	-0.165161	1.357501

Table S12. Starting material for compound **21** geometry optimization (B3LYP/6-311++G**) shows hydroxy group in a suboptimal position.

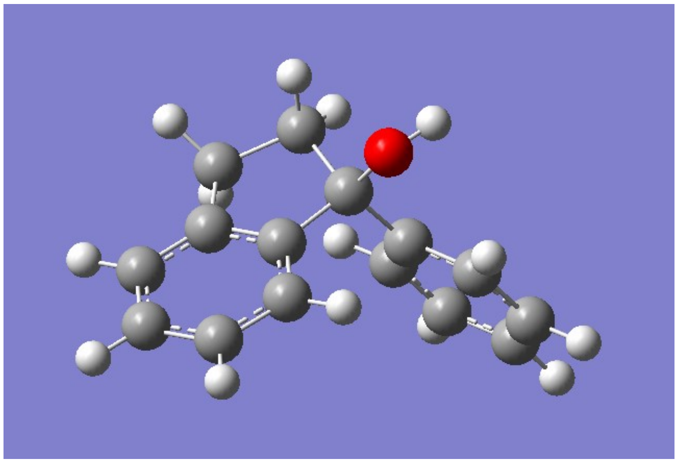

Center Number	Atomic Number	Forces (Hartrees/Bohr) X	Y	Z
1	6	0.000008198	-0.000004749	0.000000703
2	6	0.000003891	0.000020685	-0.000006828
3	6	0.000000177	-0.000000302	-0.000000384
4	6	0.000005237	-0.000001930	0.000007244
5	6	-0.000006581	0.000002370	0.000003846
6	6	-0.000003810	0.000008267	0.000004048
7	6	0.000022641	-0.000028546	0.000007367
8	6	-0.000056203	0.000036975	-0.000009946
9	6	0.000031122	-0.000022348	-0.000011698
10	1	-0.000000441	0.000001069	0.000002475
11	1	0.000002956	0.000002410	0.000004628
12	1	0.000007379	0.000002944	0.000004397
13	1	0.000001670	-0.000000493	0.000001093
14	1	0.000001883	0.000001055	-0.000011985
15	1	-0.000002165	-0.000000092	0.000001811
16	1	0.000013350	-0.000005497	-0.000003986
17	1	-0.000000796	0.000009882	-0.000006496
18	8	-0.000000202	-0.000007348	0.000015984
19	1	-0.000008388	0.000011194	-0.000005039
20	6	-0.000036392	0.000007843	-0.000000202
21	6	0.000029886	-0.000011690	0.000030391
22	6	0.000005806	0.000004093	-0.000010757
23	6	-0.000001728	0.000004829	-0.000039702
24	1	-0.000004864	0.000008212	-0.000003036
25	6	0.000019429	-0.000007030	0.000013865
26	1	-0.000002502	-0.000003240	0.000003513
27	6	-0.000028676	-0.000011139	0.000013716
28	1	-0.000000668	-0.000010023	0.000004473
29	1	-0.000004843	-0.000004203	-0.000005050

30	1	0.000004635	-0.000003197	-0.000004447

Table S13. Isodesmic equation data for Scheme 3 within manuscript.

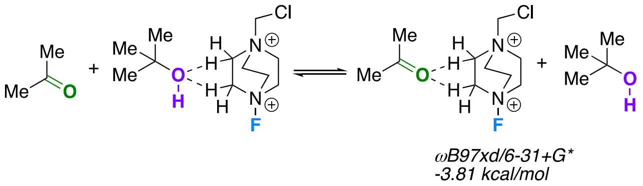

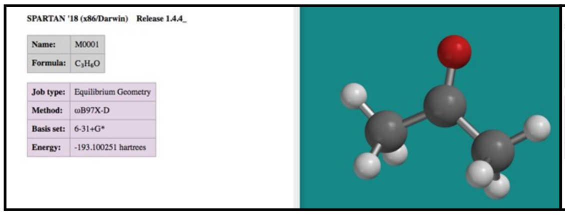

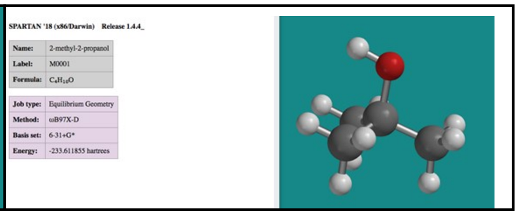

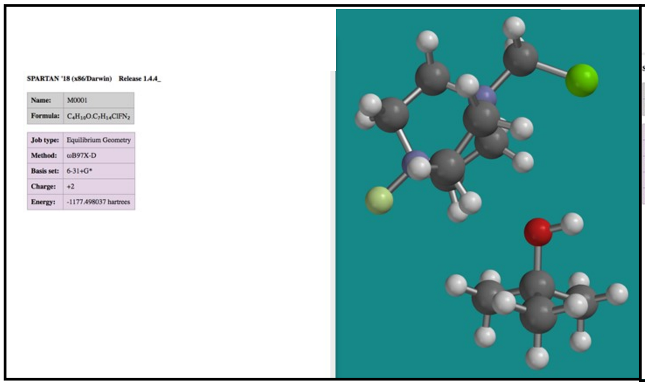

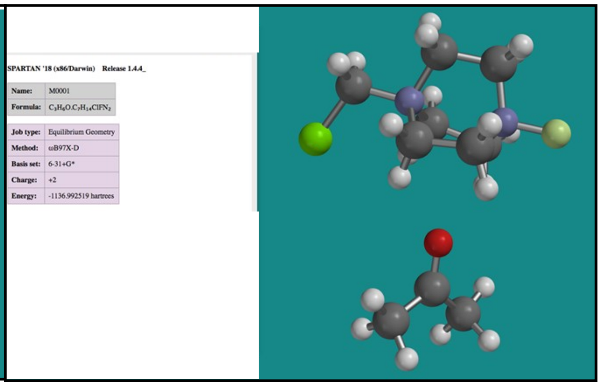

Mass Spectral Data

Elemental Composition Report **Page 1**

Single Mass Analysis
Tolerance = 100.0 PPM / DBE: min = -1.5, max = 50.0
Element prediction: Off

Monoisotopic Mass, Odd and Even Electron Ions
1 formula(e) evaluated with 1 results within limits (up to 50 best isotopic matches for each mass)
Elements Used:
C: 13-13 H: 14-76 O: 1-1 Cl: 1-1
Lectka_Stefan_SAH-K-SM_06232021_LIFDI 190 (3.167)
TOF MS FD+

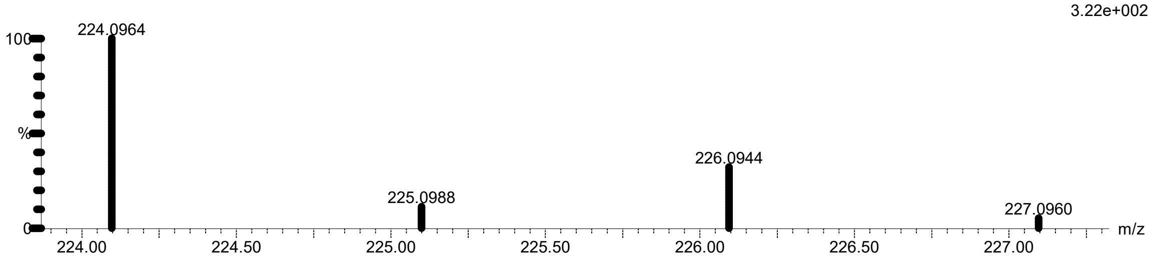

Minimum:				-1.5		
Maximum:		5.0	100.0	50.0		
Mass	Calc. Mass	mDa	PPM	DBE	i-FIT	Formula
224.0964	224.0968	-0.4	-1.8	5.0	1.4	C13 H17 O Cl

Fig. S75. High-resolution mass spectrum of compound **5**.

Elemental Composition Report

Page 1

Single Mass Analysis

Tolerance = 100.0 PPM / DBE: min = -1.5, max = 50.0
Element prediction: Off

Monoisotopic Mass, Odd and Even Electron Ions
2 formula(e) evaluated with 1 results within limits (up to 50 best isotopic matches for each mass)
Elements Used:
C: 13-17 H: 10-76 O: 1-1 F: 1-1 Cl: 1-2
Lectka_Stefan_SAH_K_Fluoro_06232021-GCMS 488 (10.194)
TOF MS EI+

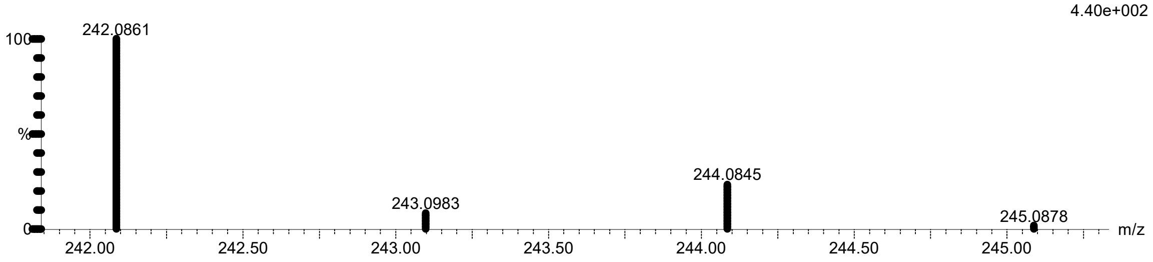

Minimum:				-1.5		
Maximum:		5.0	100.0	50.0		
Mass	Calc. Mass	mDa	PPM	DBE	i-FIT	Formula
242.0861	242.0874	-1.3	-5.4	5.0	10.8	C13 H16 O F Cl

Fig. S76. High-resolution mass spectrum of compound **6**.

Elemental Composition Report

Page 1

Single Mass Analysis

Tolerance = 100.0 PPM / DBE: min = -1.5, max = 50.0
Element prediction: Off

Monoisotopic Mass, Odd and Even Electron Ions
3 formula(e) evaluated with 1 results within limits (up to 50 best isotopic matches for each mass)
Elements Used:
C: 13-17 H: 10-76 O: 1-1 F: 1-1
Lectka_Stefan_SAH_D_Fluoro_06232021-GCMS 457 (9.678)
TOF MS EI+

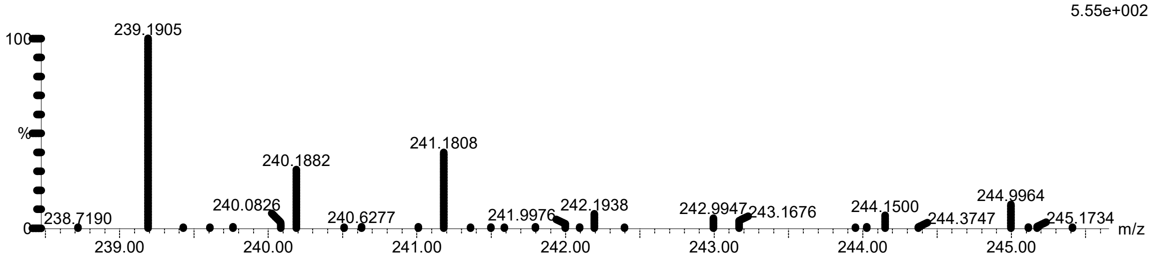

Minimum:				-1.5		
Maximum:		5.0	100.0	50.0		
Mass	Calc. Mass	mDa	PPM	DBE	i-FIT	Formula
240.1882	240.1889	-0.7	-2.9	3.0	101.8	C15 H25 O F

Fig. S77. High-resolution mass spectrum of compound **7**.

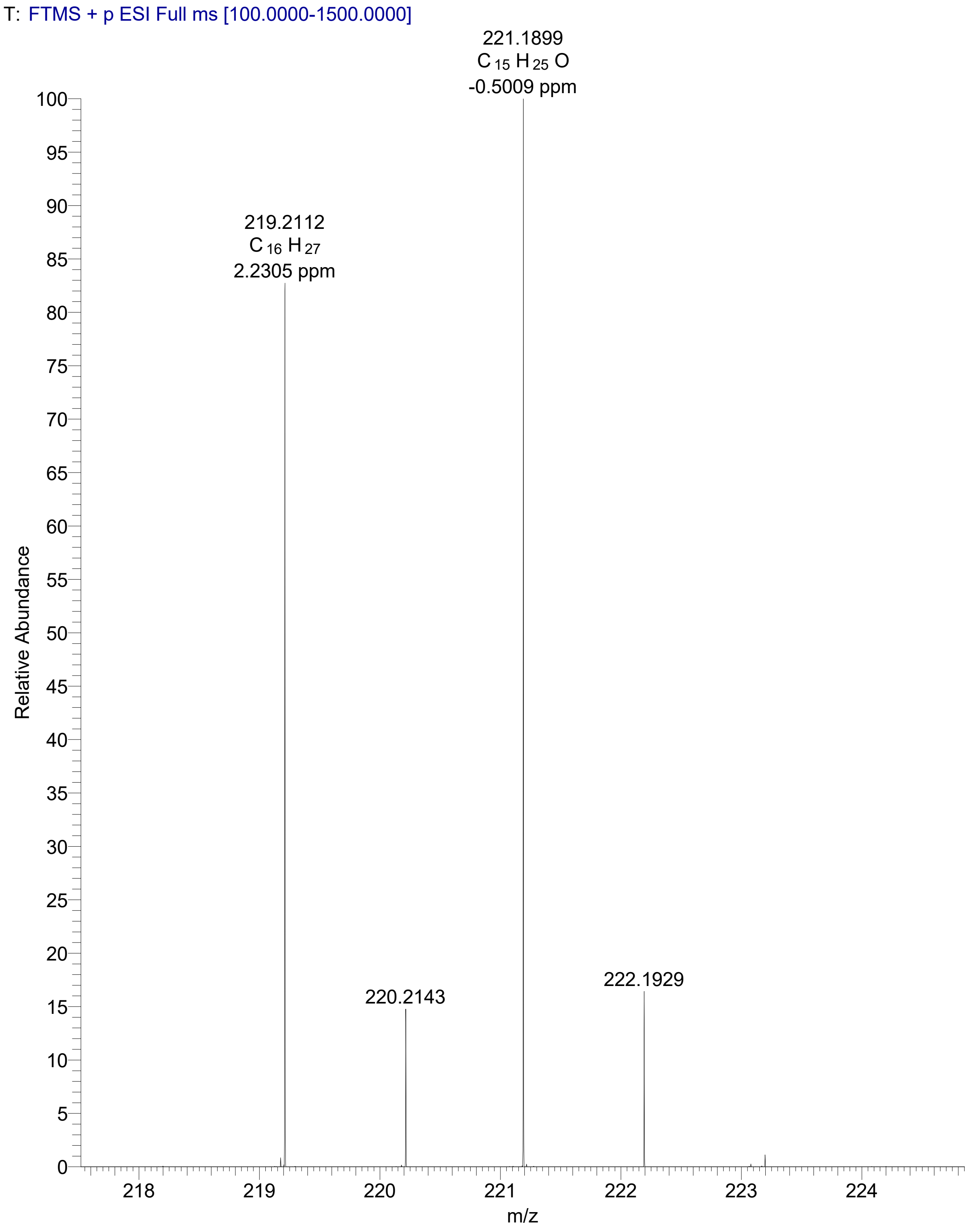

Fig. S78. High-resolution mass spectrum of the starting material for compound **8**.

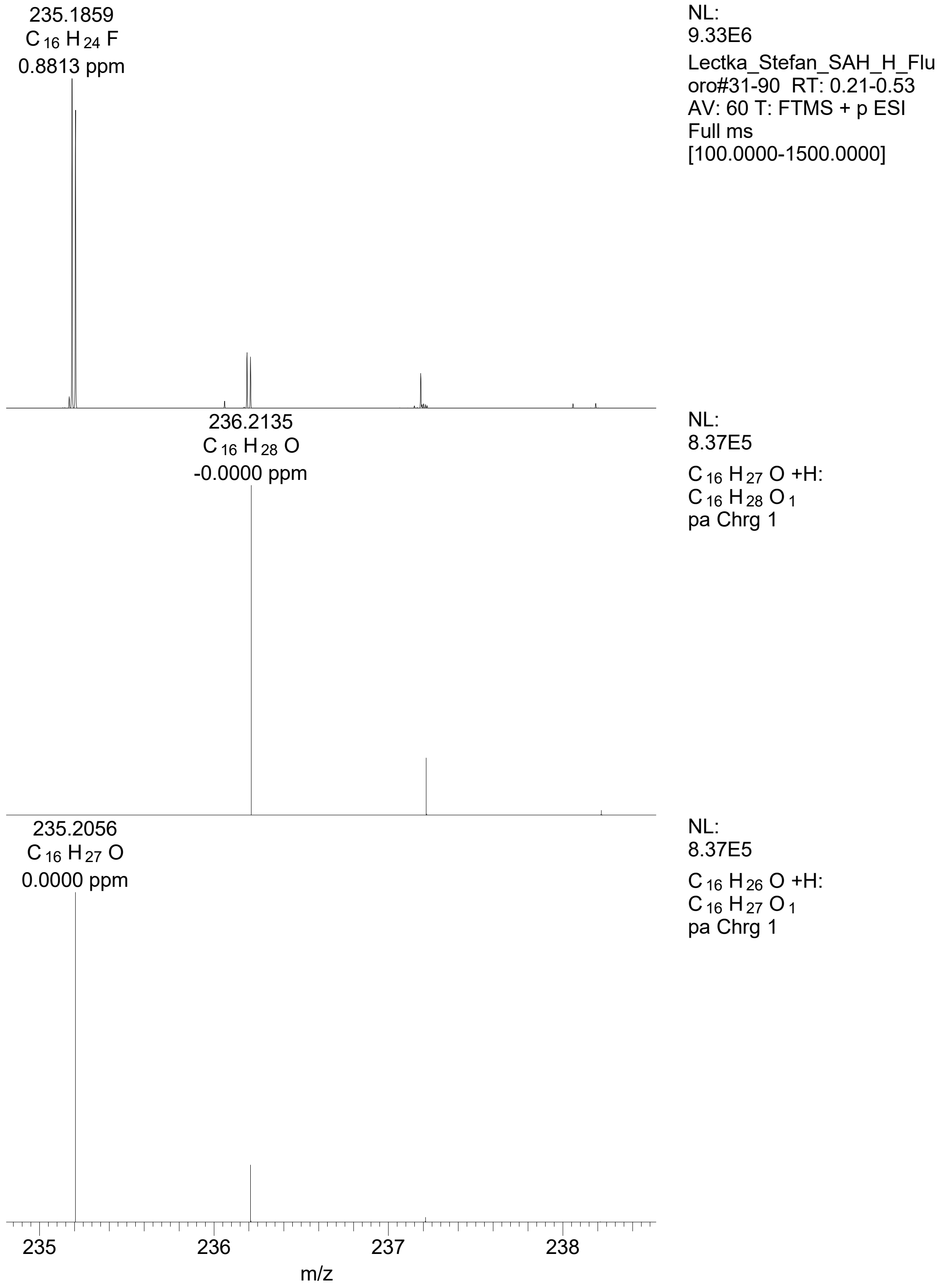

Fig. S79. High-resolution mass spectrum of compound **8**.

Elemental Composition Report

Single Mass Analysis
Tolerance = 100.0 PPM / DBE: min = -1.5, max = 50.0
Element prediction: Off

Monoisotopic Mass, Odd and Even Electron Ions
3 formula(e) evaluated with 1 results within limits (up to 50 best isotopic matches for each mass)
Elements Used:
C: 13-17 H: 10-76 O: 1-1
Lectka_Stefan_SAH_E_SM_06232021-GCMS 447 (9.509)
TOF MS EI+

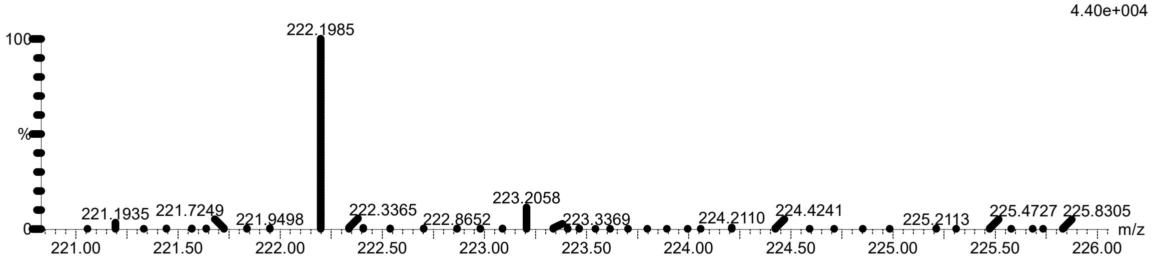

Minimum:				-1.5		
Maximum:		5.0	100.0	50.0		
Mass	Calc. Mass	mDa	PPM	DBE	i-FIT	Formula
222.1985	222.1984	0.1	0.5	3.0	482.0	C15 H26 O

Fig. S80. High-resolution mass spectrum of the starting material for compound **9**.

Elemental Composition Report

Single Mass Analysis
Tolerance = 100.0 PPM / DBE: min = -1.5, max = 50.0
Element prediction: Off

Monoisotopic Mass, Odd and Even Electron Ions
3 formula(e) evaluated with 1 results within limits (up to 50 best isotopic matches for each mass)
Elements Used:
C: 13-17 H: 10-76 O: 1-1 F: 1-1
Lectka_Stefan_SAH_E_Fluoro_06232021-GCMS 438 (9.359)
TOF MS EI+

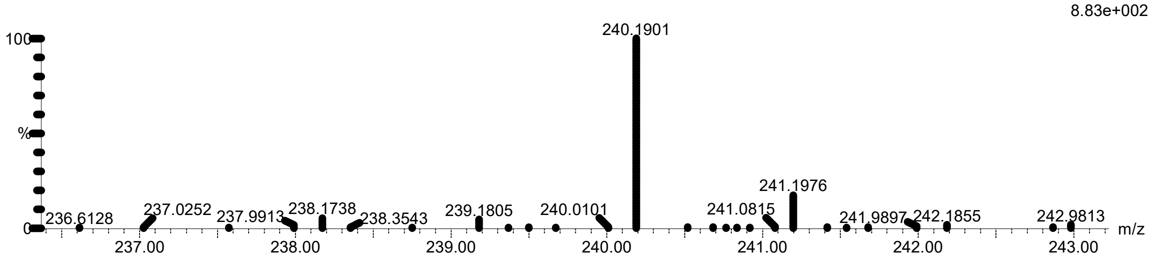

Minimum:				-1.5		
Maximum:		5.0	100.0	50.0		
Mass	Calc. Mass	mDa	PPM	DBE	i-FIT	Formula
240.1901	240.1889	1.2	5.0	3.0	0.5	C15 H25 O F

Fig. S81. High-resolution mass spectrum of compound **9**.

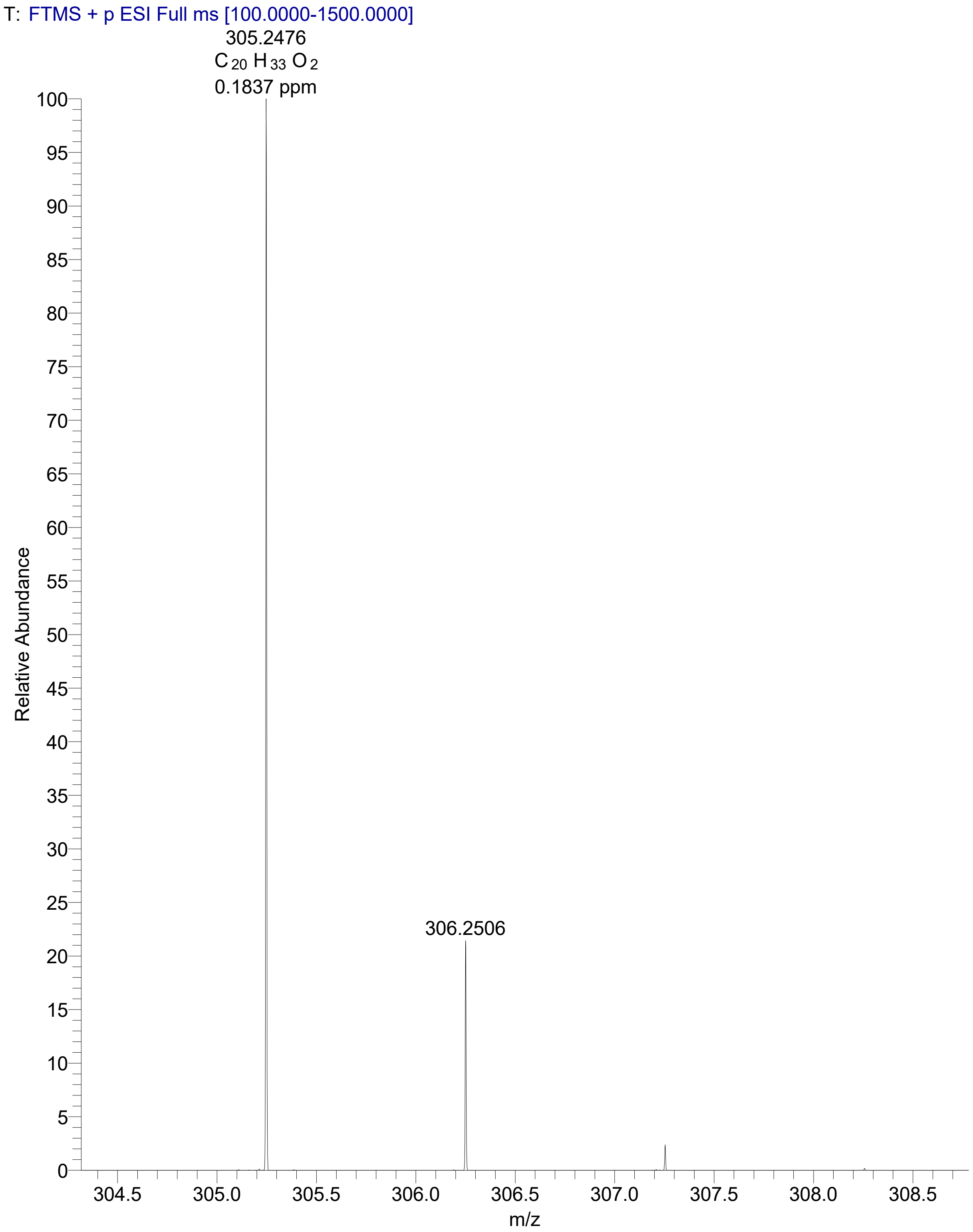

Fig. S82. High resolution mass spectrum of the starting material for compound **10**.

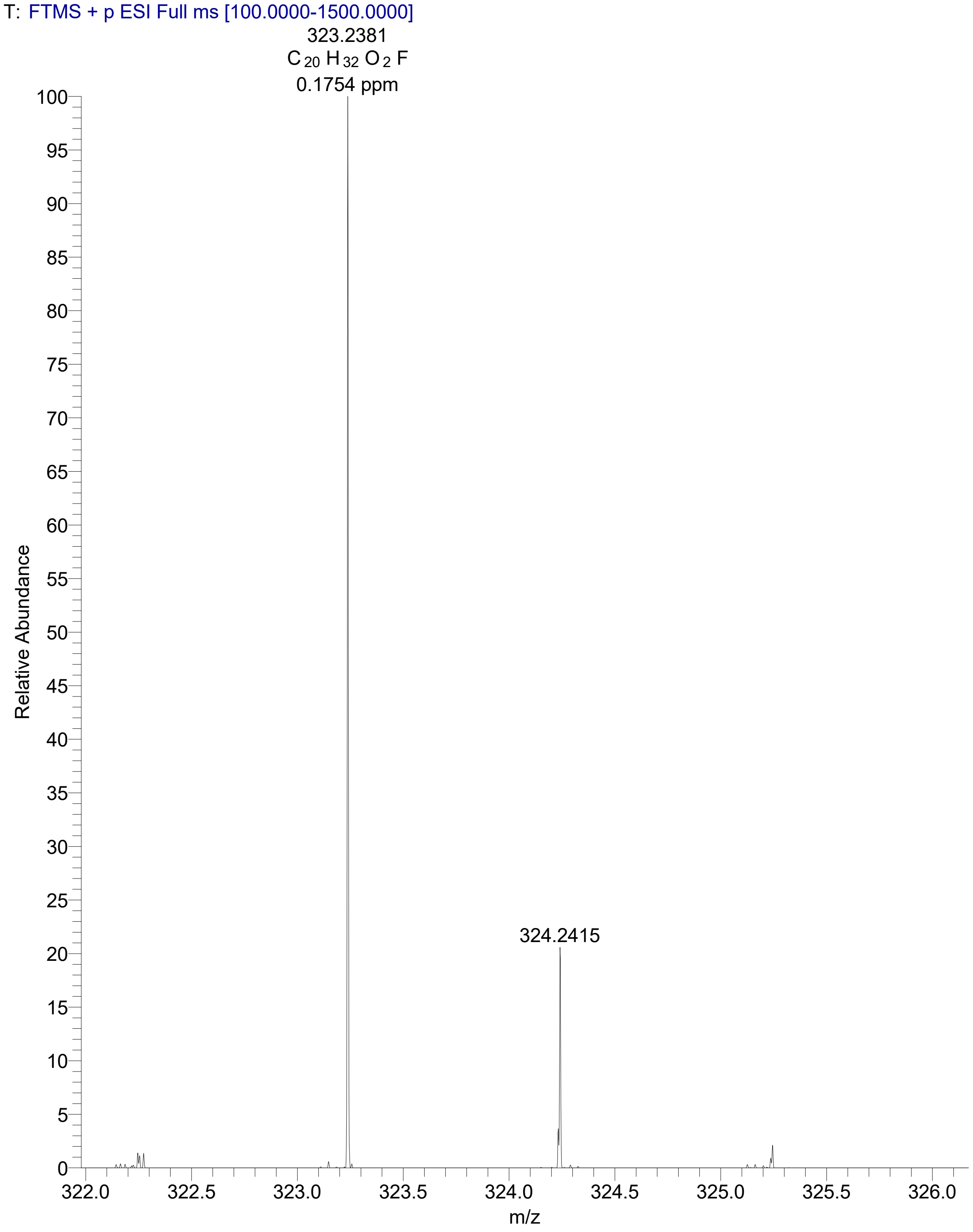

Fig. S83. High-resolution mass spectrum of compound **10**.

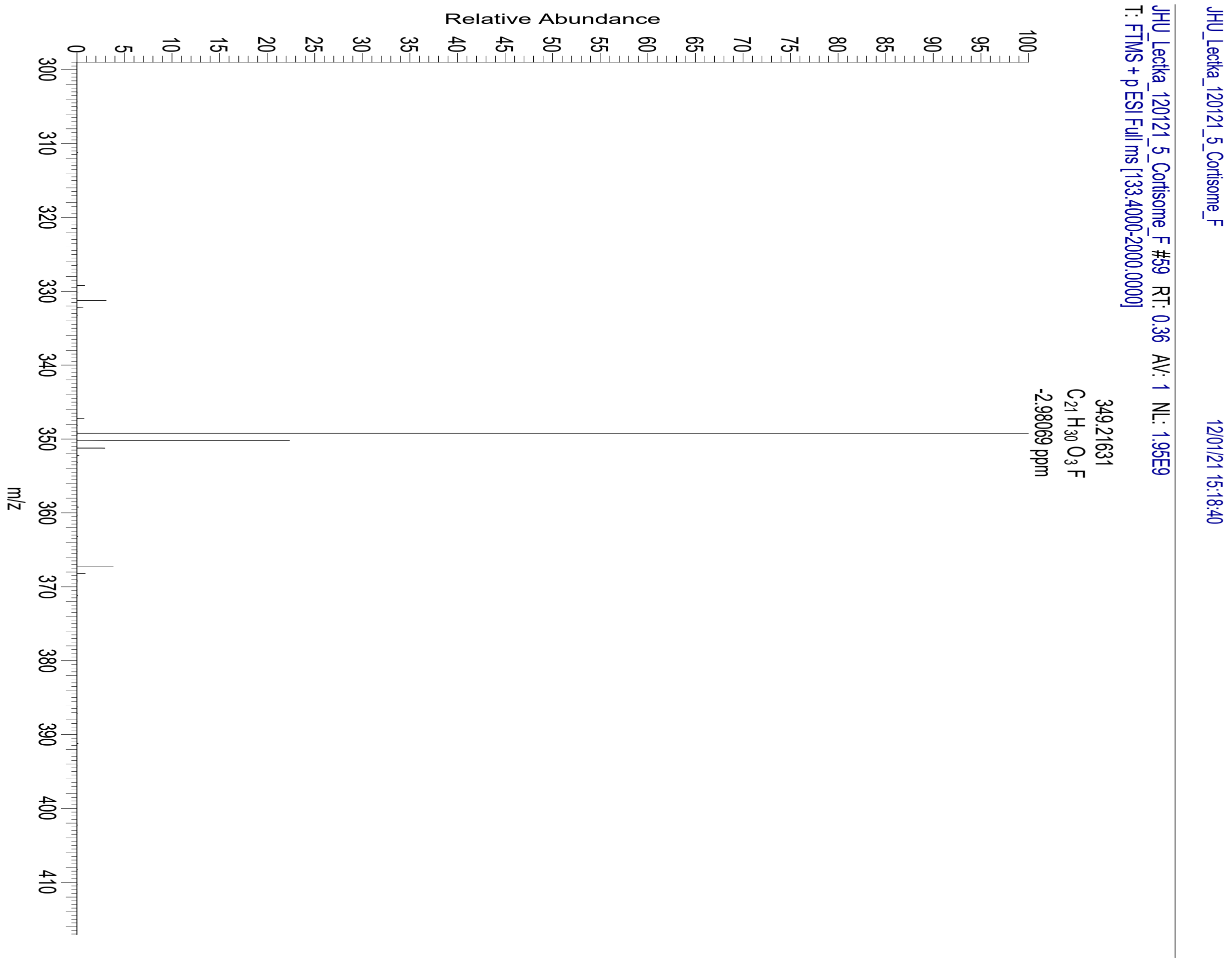

Fig. S84. High-resolution mass spectrum of compound **11**.

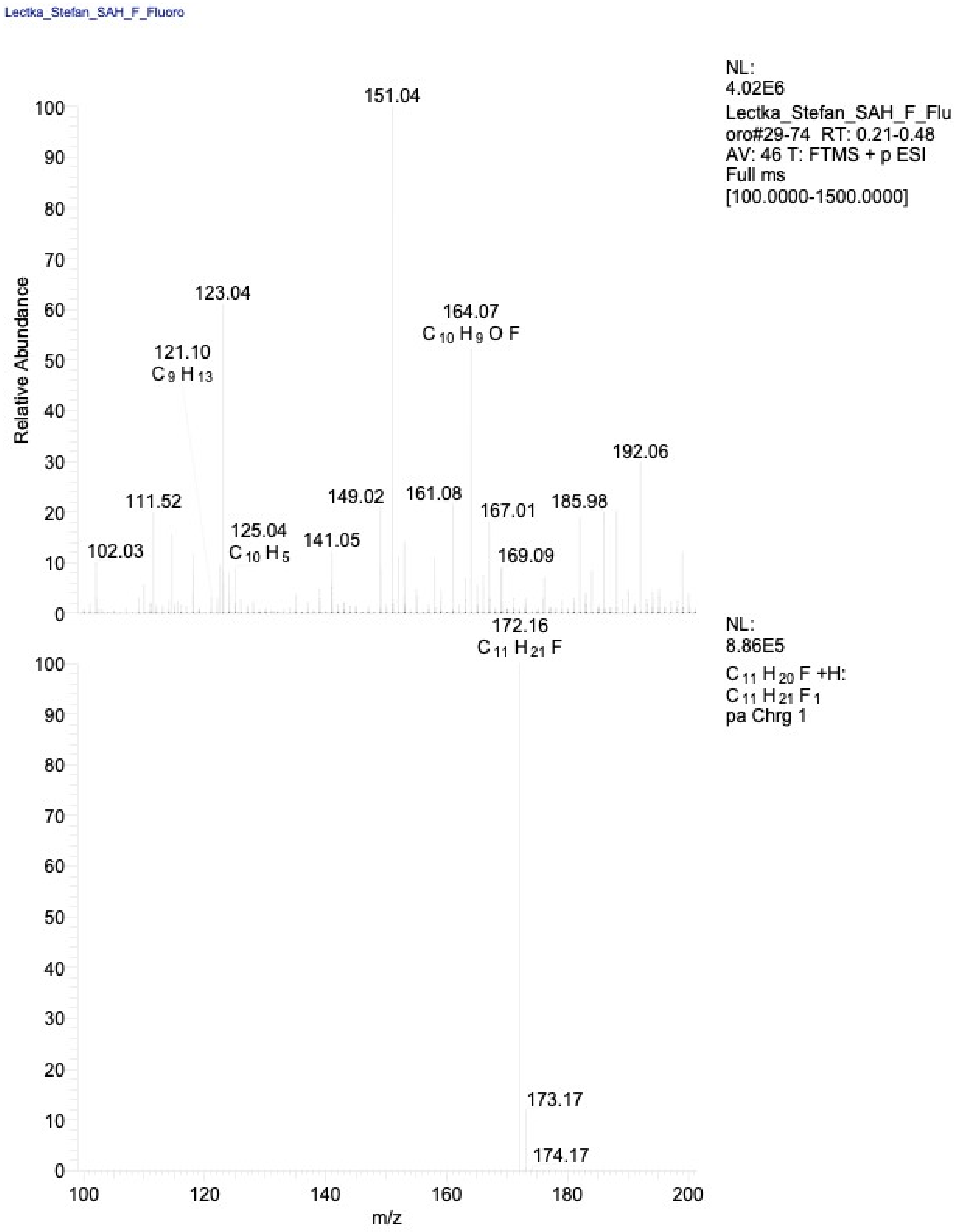

Fig. S85. Mass spectrum of compound **13**.

Elemental Composition Report **Page 1**

Single Mass Analysis
Tolerance = 100.0 PPM / DBE: min = -1.5, max = 50.0
Element prediction: Off

Monoisotopic Mass, Odd and Even Electron Ions
2 formula(e) evaluated with 1 results within limits (up to 50 best isotopic matches for each mass)
Elements Used:
C: 13-17 H: 14-76 O: 4-4
Lectka_Stefan_SAH-M-SM_06232021_LIFDI_2 62 (1.034)
TOF MS FD+

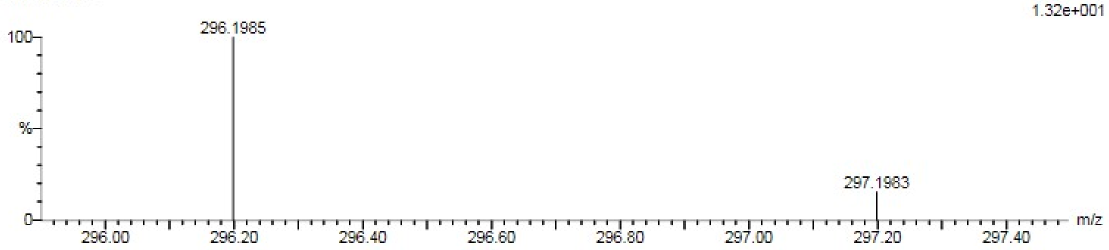

Minimum:				-1.5		
Maximum:		5.0	100.0	50.0		
Mass	Calc. Mass	mDa	PPM	DBE	i-FIT	Formula
296.1985	296.1988	-0.3	-1.0	4.0	2773012.8	C17 H28 O4

Fig. S86. High-resolution mass spectrum of starting material for compound **14**.

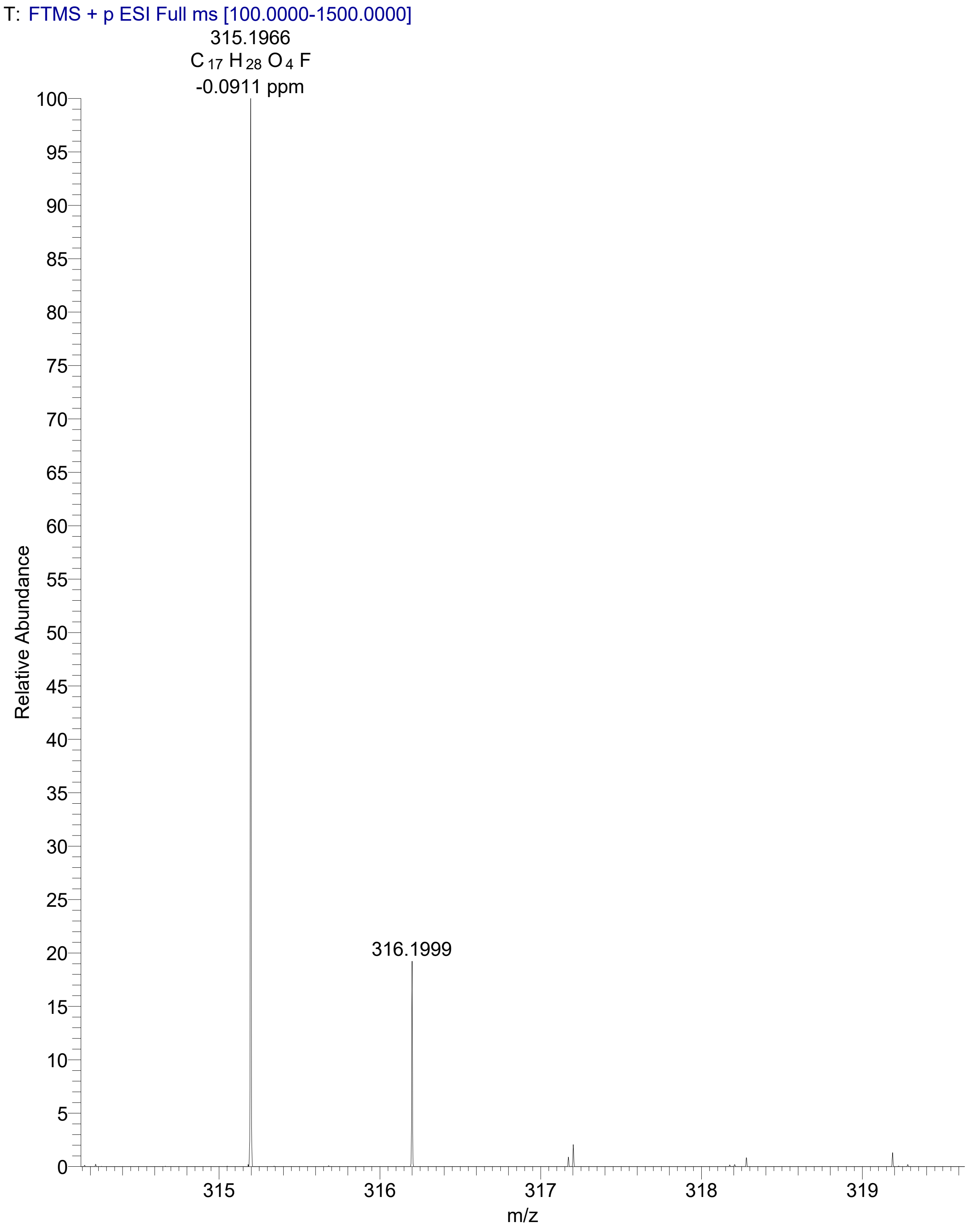

Fig. S87. High-resolution mass spectrum of compound **14**.

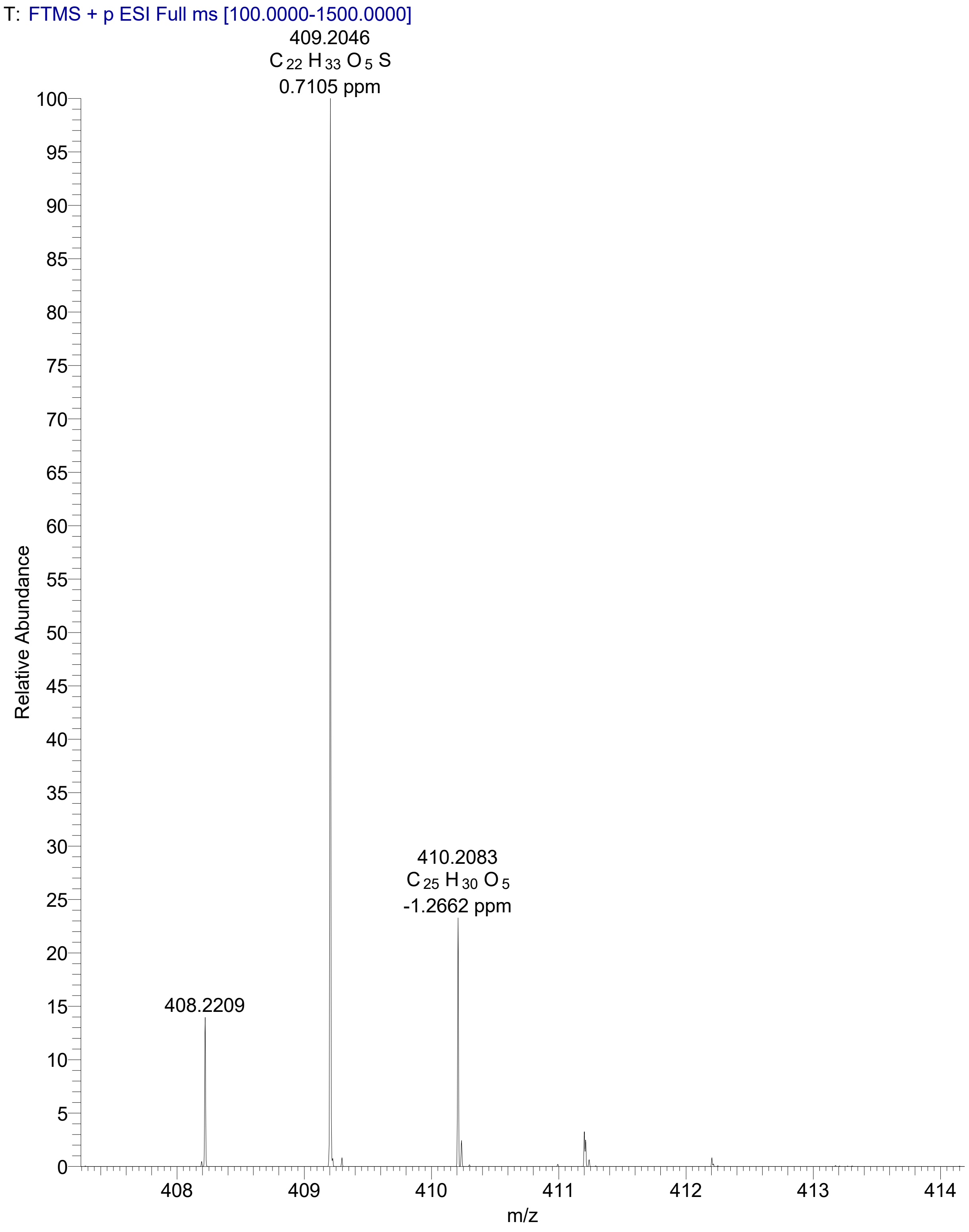

Fig. S88. High resolution mass spectrum of the starting material for compound **15**.

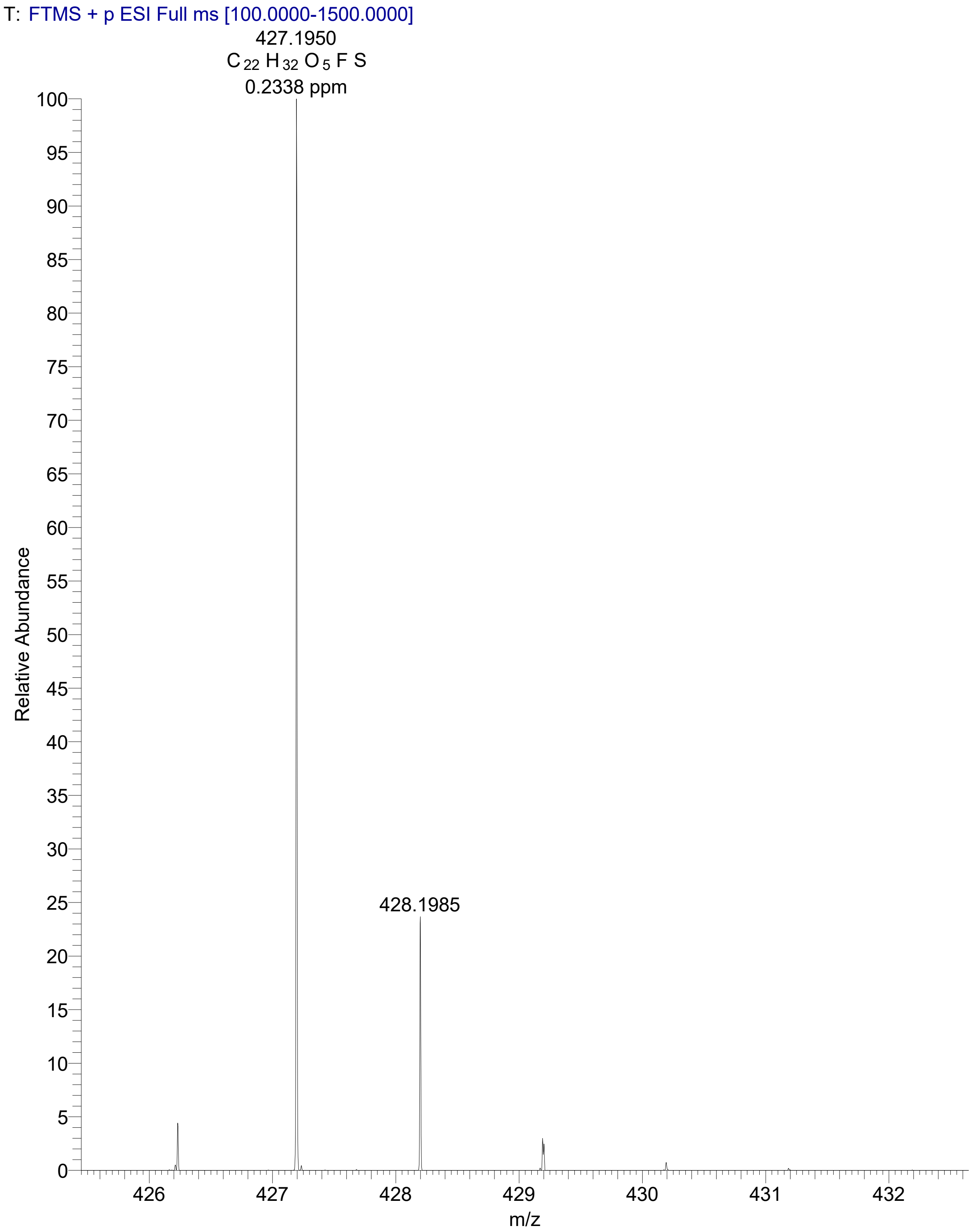

Fig. S89. High resolution mass spectrum of compound **15**.

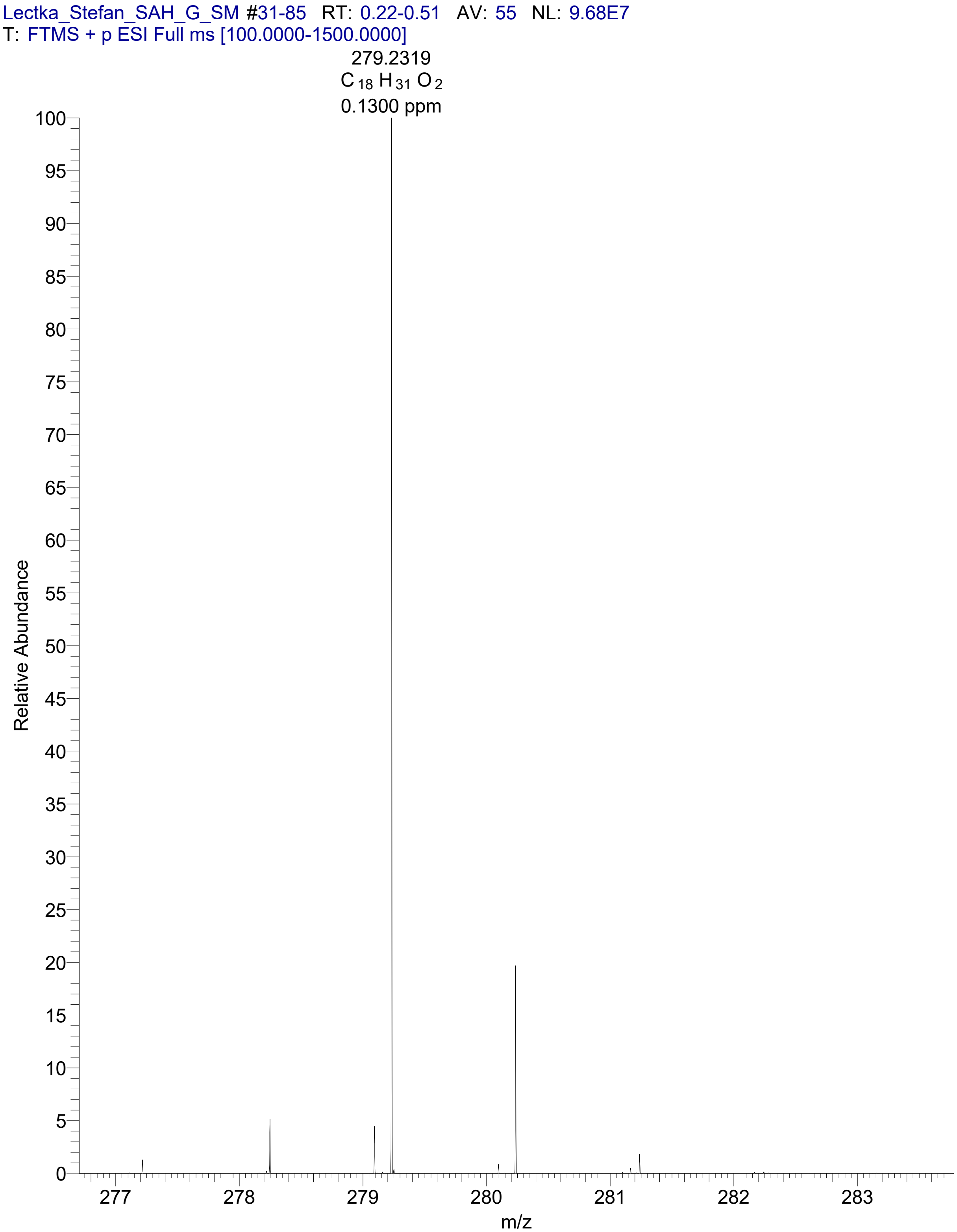

Fig. S90. High-resolution mass spectrum of the starting material for compound **16**.

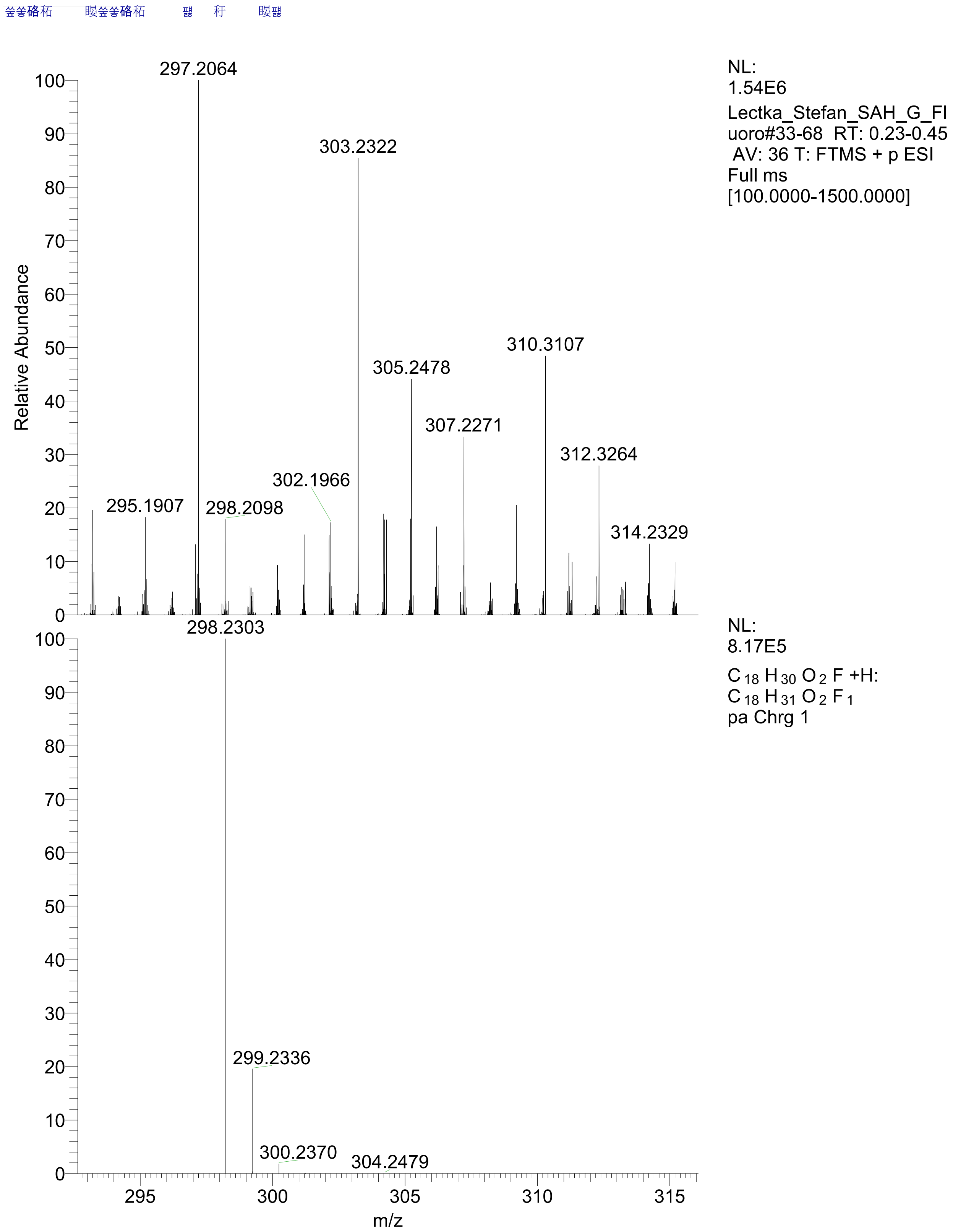

Fig. S91. High-resolution mass spectrum of compound **16**.

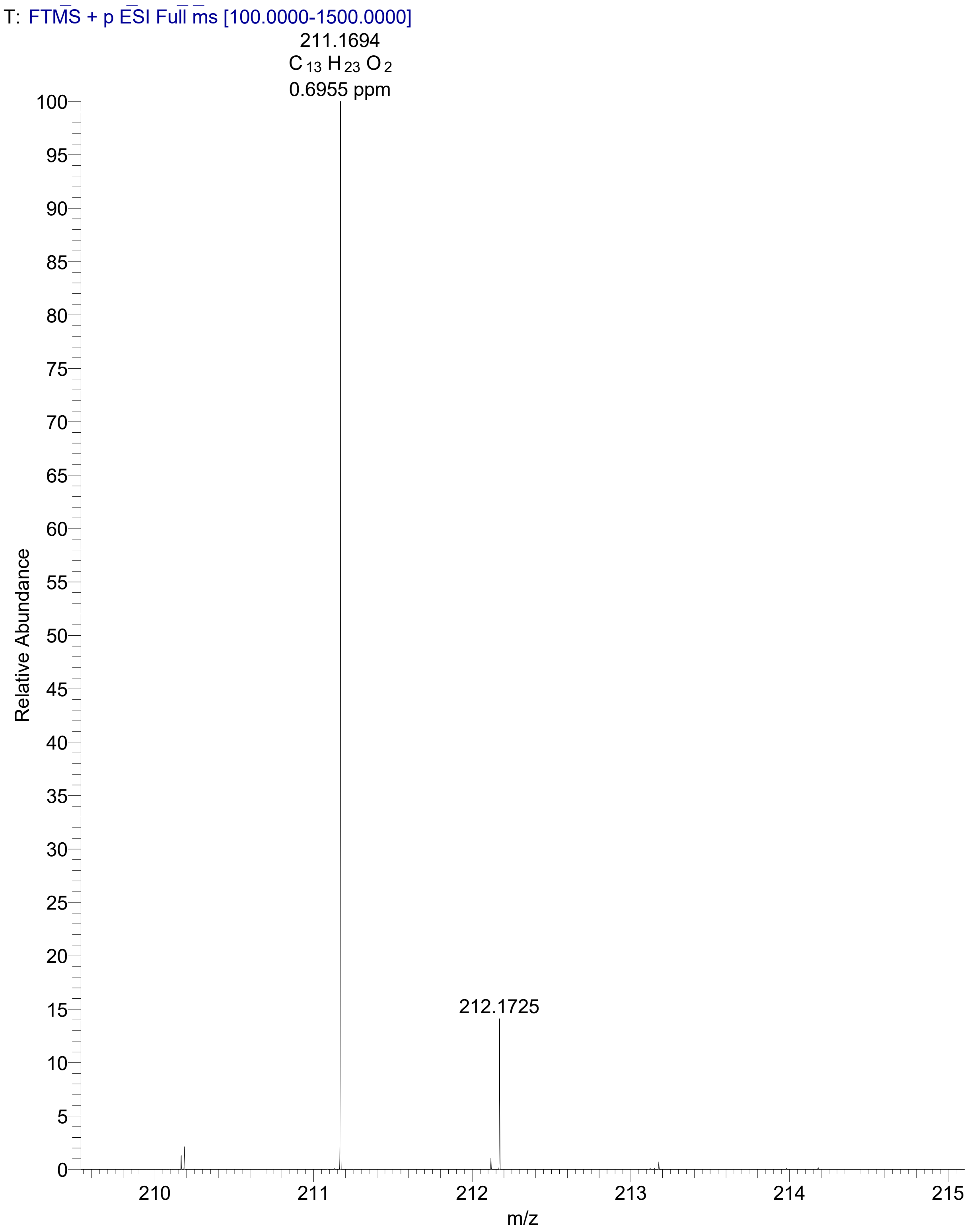

Fig. S92. High resolution mass spectrum of the starting material for compound **17**.

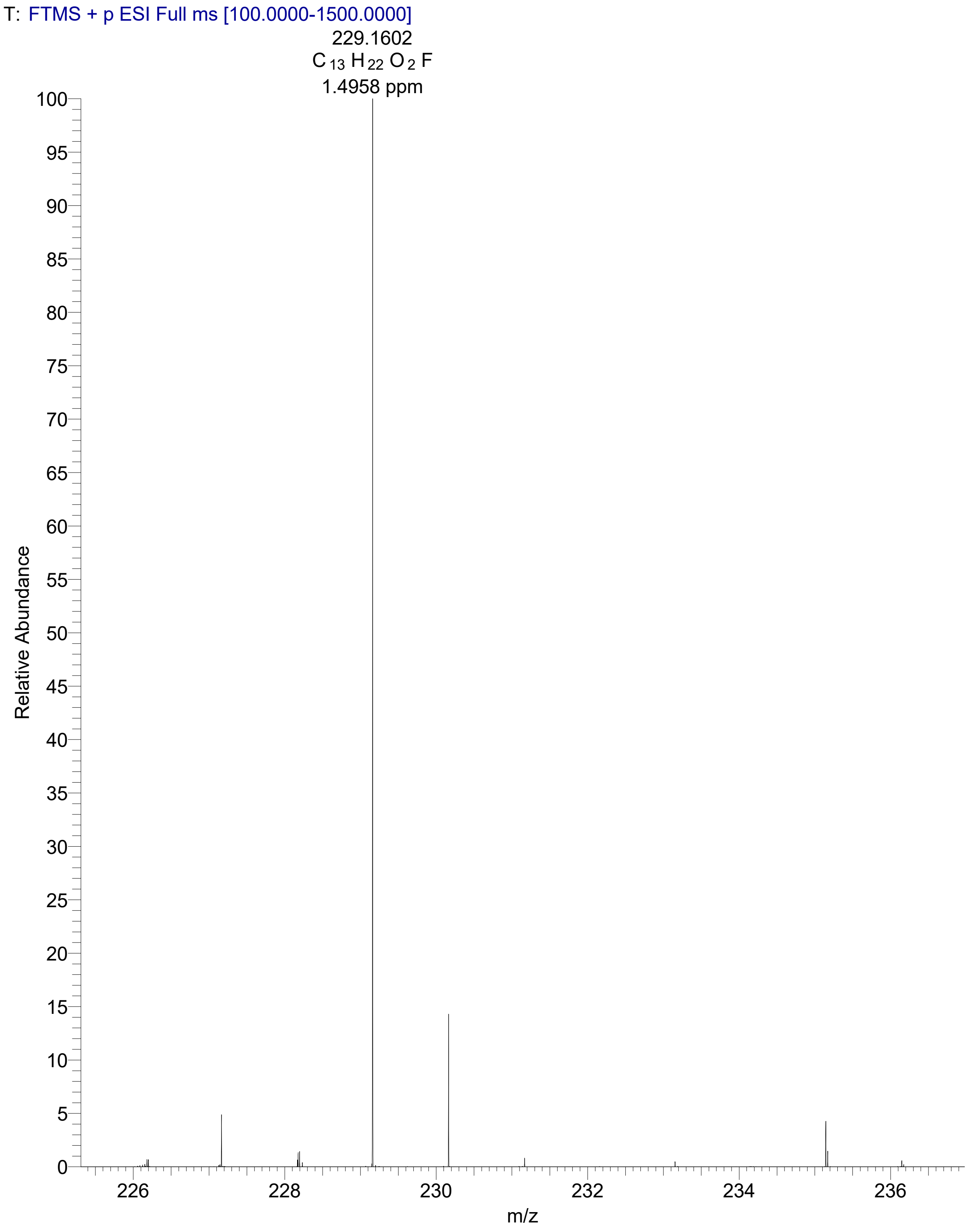

Fig. S93. High-resolution mass spectrum of compound **17**.

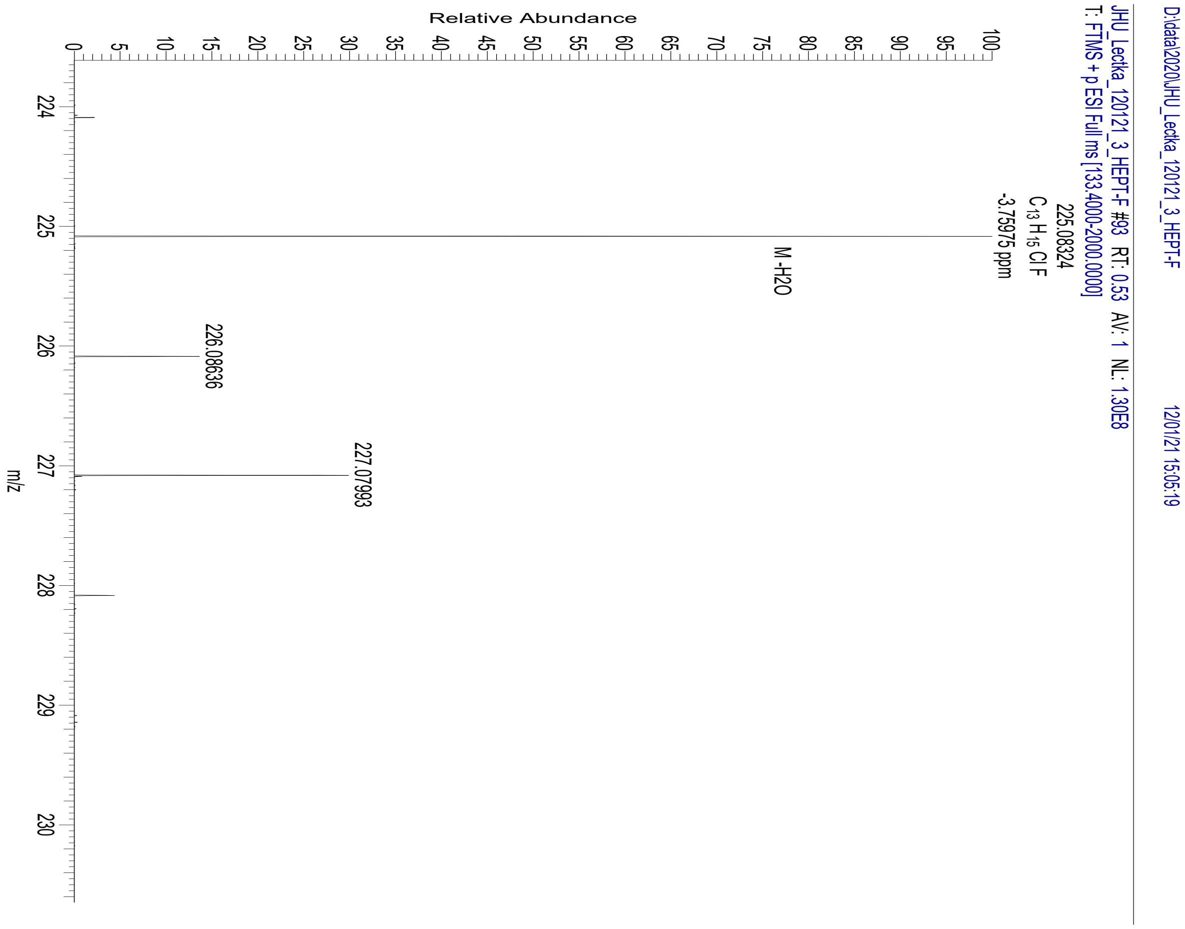

Fig. S94. High-resolution mass spectrum of compound **18**.

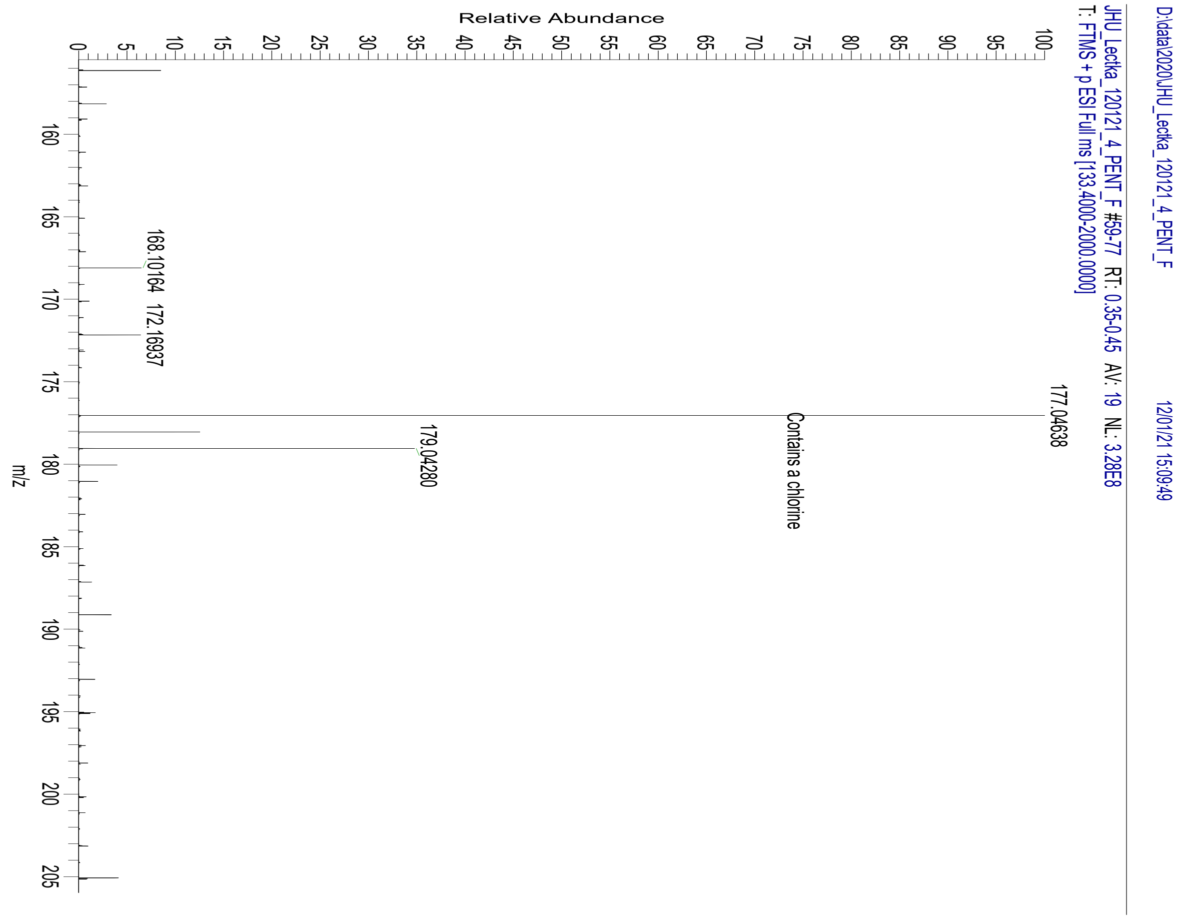

Fig. S95. High-resolution mass spectrum of compound **19**.

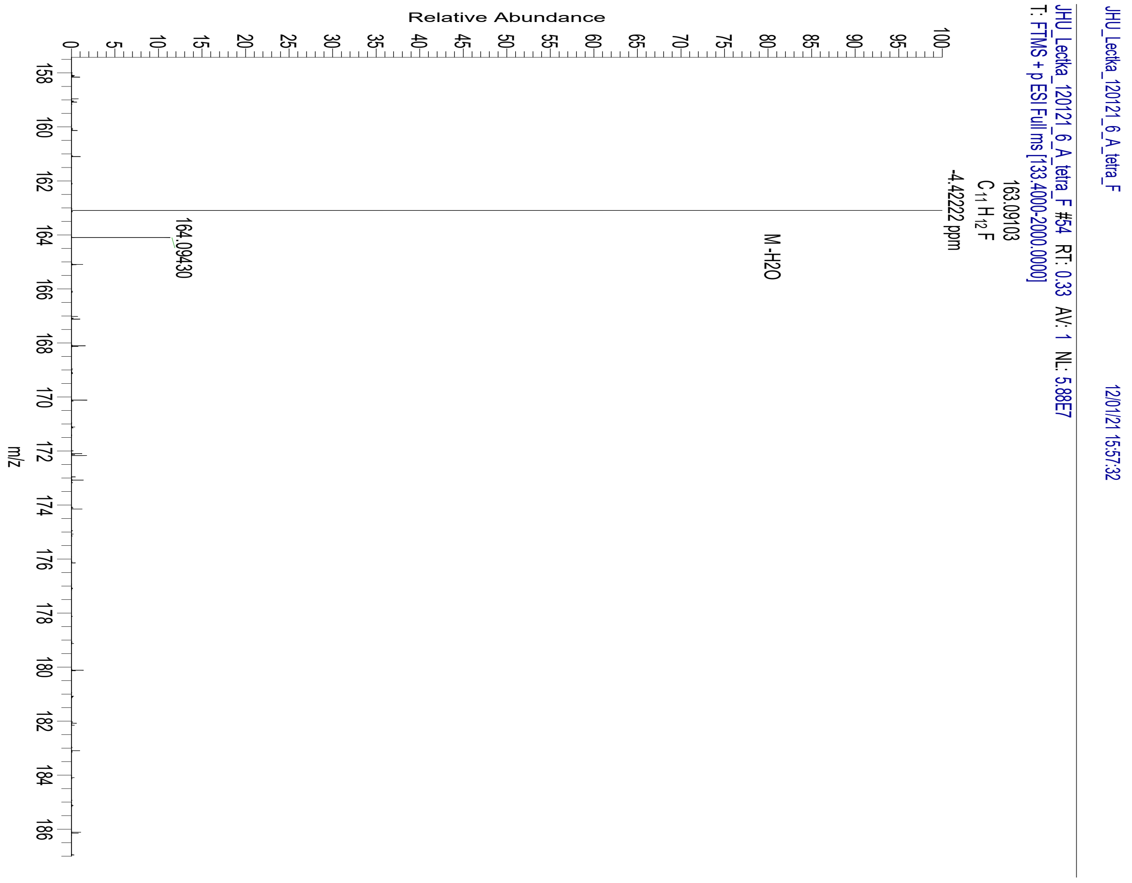

Fig. S96. High-resolution mass spectrum of compound **20**.

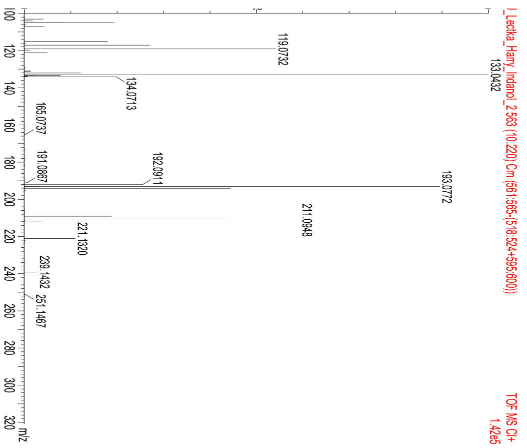

Fig. S97. High-resolution mass spectrum for compound **21**.

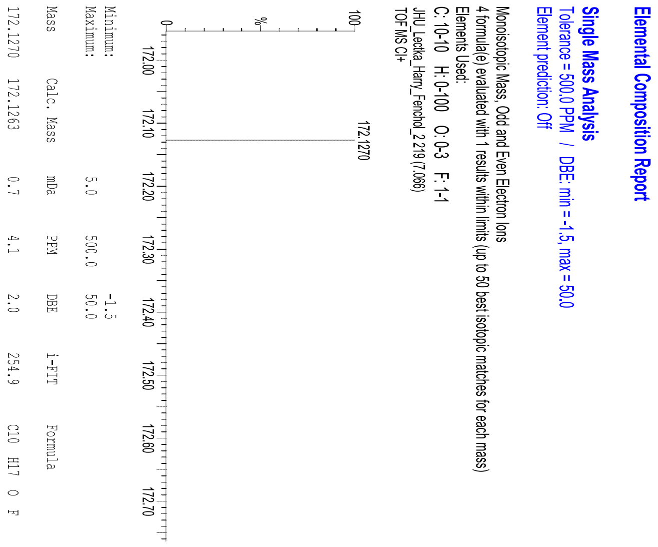

Fig. S98. High-resolution mass spectrum for compound **22**.

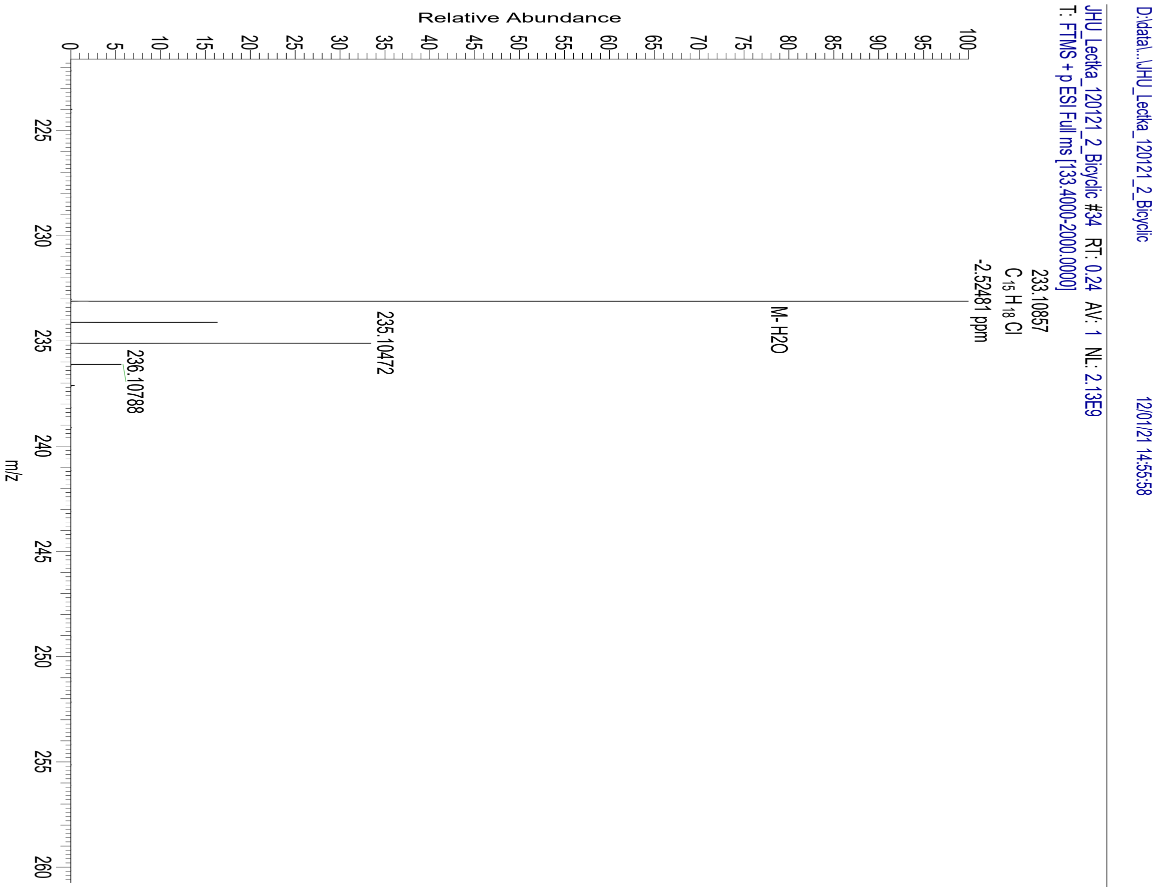

Fig. S99. High-resolution mass spectrum of the starting material for compound **23**.

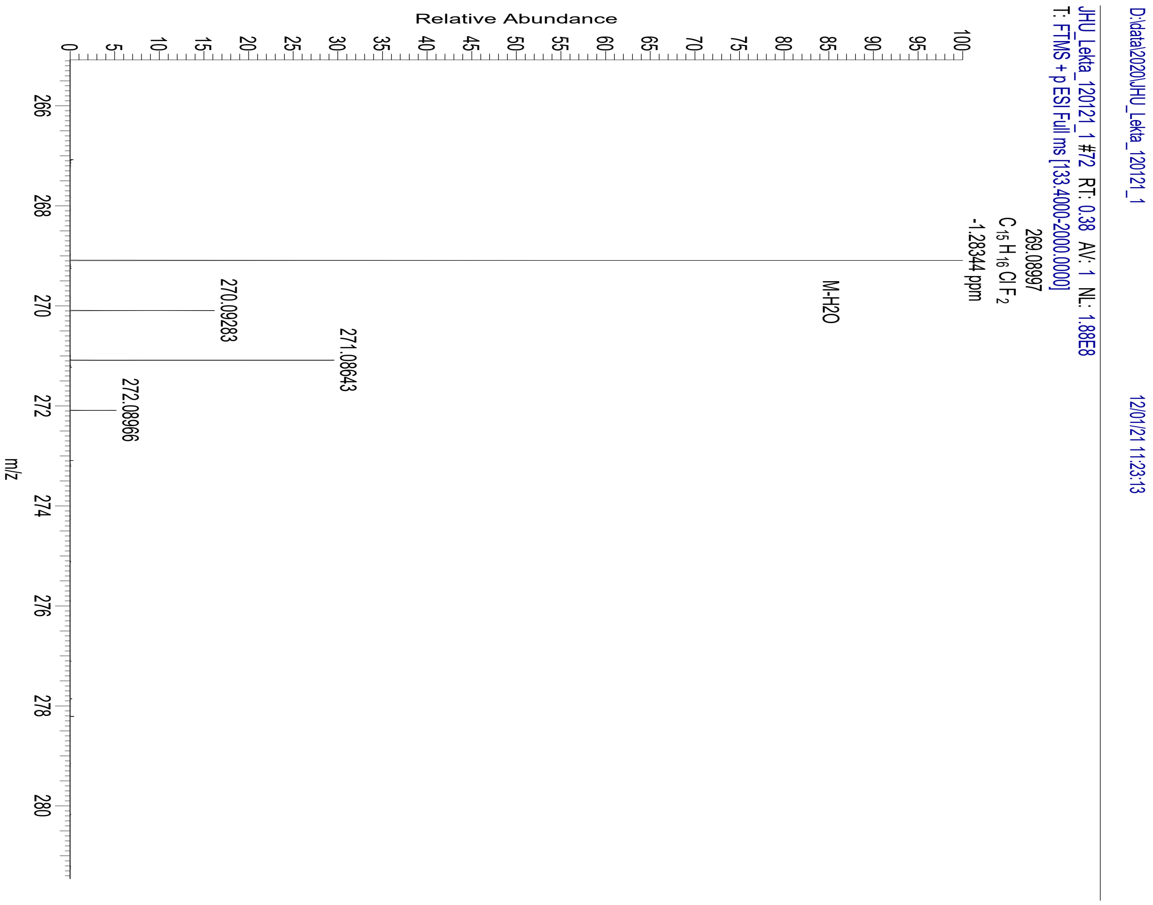

Fig. S100. High-resolution mass spectrum of compound **23**.

References

[1] A. Huczynski, J. Rutkowski, B. Brzezinski, *Struct. Chem.* **2011**, *22*, 627-634.

[2] M. T. Reetz, S. Stanchev, *J. Chem. Soc. Chem. Commun.* **1993**, *3*, 328-330.

[3] C. Oberhauser, V. Harms, K. Seidel, B. Schröder, K. Ekramzadeh, S. Beutel, S. Winkler, L. Lauterbach, J. Dickschat, A. Kirschning, *Angew. Chem.* **2018**, *57*, 11802-11806.

[4] R. Fernandes, D. Chaudhari, *Eur. J. Org. Chem.* **2012**, *10*, 1945-1952.

[5] C. Karmel, B. Li, J. Hartwig, *J. Am. Chem. Soc.* **2018**, *140*, 1460-1470.

[6] R. Deslauriers, F. Hasan, B. Lodge, I. Smith, Ian, *J. Pharm. Sci.* **1978**, *67*, 1187-1189.

[7] R. Fernandes, D. Chaudhari, *Eur. J. Org. Chem.* **2012**, *10*, 1945-1952.

[8] E. Bratoeff, P. García, Y. Heuze, J. Soriano, A. Mejía, A. Labastida, N. Valencia, M. Cabeza, *Steroids*, **2010**, *75*, 499-505.

[9] P. Kathe, A. Berkefeld, I. Fleischer, *Synlett* **2021**, *32*, 1629-1632.

[10] M. Magre, E. Paffenholz, B. Maity, L. Cavallo, M. Rueping, *J. Am. Chem. Soc*. **2020**, *142*, 14286-14294.

[11] J. Kula, R. Bonikowski, M. Staniszewska, A. Krakowiak, M. Wieczorek, W. Majzner, G. Bujacz, *Eur. J. Org. Chem*. **2002,** *11*, 1826-1829.

[12] R. Saleem, J. Meinwald, *J. Chem. Soc*. **2000**, *3*, 391-394.

[13] M. Jackson, C. Moody, P. Shah, *J. Am. Chem. Soc.* **1990**, *11*, 2909-2918.

[14] T. Duvold, M. Sorensen, F. Bjorkling, A. Henriksen, N. Rastrup-Anderson, *J. Med. Chem.* **2001**, *44*, 3125-3131.

[15] G. Lee, H. Lee, W. Wang, C. Cherng, *Tetrahedron* **2014**, *70*, 2956-2961.

[16] D. Özdemirhan, *Synth. Commun*. **2017**, *47*, 629-645.

[17] C. Nielsen, A.White, D. Sale, J. Bures, A. Spivey, *J. Org. Chem.* **2019**, *84*, 14965-14973.

[18] F. Bohlmann, R. Zeisberg, *Org. Mag. Reson.* **1975**, *7*, 426.

[19] E. Hayashi, S. Hara, H. Shirato, T. Hatekeyama, T. Fukuhara, N. Yoneda, *Chem*. *Lett*. **1995**, *3*, 205-206.
